# Comparative Proteomics Reveals Novel Components at the Plasma Membrane of Differentiated HepaRG Cells and Different Distribution in Hepatocyte- and Biliary-Like Cells

**DOI:** 10.1371/journal.pone.0071859

**Published:** 2013-08-20

**Authors:** Catalina Petrareanu, Alina Macovei, Izabela Sokolowska, Alisa G. Woods, Catalin Lazar, Gabriel L. Radu, Costel C. Darie, Norica Branza-Nichita

**Affiliations:** 1 Department of Viral Glycoproteins, Institute of Biochemistry of the Romanian Academy, Bucharest, Romania; 2 Department of Analytical Chemistry and Enviromental Engineering, Faculty of Applied Chemistry and Materials Science, Politehnica University of Bucharest, Bucharest, Romania; 3 Biochemistry and Proteomics Group, Department of Chemistry and Biomolecular Science, Clarkson University, Potsdam, New York, United States of America; Yonsei University, Republic of Korea

## Abstract

Hepatitis B virus (HBV) is a human pathogen causing severe liver disease and eventually death. Despite important progress in deciphering HBV internalization, the early virus-cell interactions leading to infection are not known. HepaRG is a human bipotent liver cell line bearing the unique ability to differentiate towards a mixture of hepatocyte- and biliary-like cells. In addition to expressing metabolic functions normally found in liver, differentiated HepaRG cells support HBV infection *in vitro,* thus resembling cultured primary hepatocytes more than other hepatoma cells. Therefore, extensive characterization of the plasma membrane proteome from HepaRG cells would allow the identification of new cellular factors potentially involved in infection. Here we analyzed the plasma membranes of non-differentiated and differentiated HepaRG cells using nanoliquid chromatography-tandem mass spectrometry to identify the differences between the proteomes and the changes that lead to differentiation of these cells. We followed up on differentially-regulated proteins in hepatocytes- and biliary-like cells, focusing on Cathepsins D and K, Cyclophilin A, Annexin 1/A1, PDI and PDI A4/ERp72. Major differences between the two proteomes were found, including differentially regulated proteins, protein-protein interactions and intracellular localizations following differentiation. The results advance our current understanding of HepaRG differentiation and the unique properties of these cells.

## Introduction

Of the human hepatoma-derived cell lines isolated so far, the HepaRG cells originating from a hepatic tumor of a HCV-infected patient have unique properties [1], [2]. The cells have bipotent progenitor features, being able to synchronously develop both, hepatocyte-like and biliary-like epithelial phenotypes when differentiated [3]. Optimal differentiation of HepaRG cells, in the presence of dimethylsulfoxide (DMSO), promotes expression of high levels of adult hepatocyte-specific genes, such as albumin and aldolase B, several phase I and II biotransformation enzymes with a function in drug metabolism, proteins involved in oxidative stress relief, membrane transporters and hepatocyte nuclear transcription factors [4], [5]. Owing to these singular features, HepaRG cells are a valuable alternative to primary human hepatocytes and have become an excellent *in vitro* liver cell model to investigate xenobiotic metabolism and genotoxic compounds [2], [6]. Recent studies have shown that the HepaRG cell line is also a good model to study the innate immunity, as differentiation is accompanied by increased expression of interferon-regulatory factors [7]. Moreover, the discovery of HepaRG cells has provided a ground-breaking tool to address the mechanisms of hepatic viral infections, being the first human liver-derived cell line permissive for infection with hepatitis B virus (HBV) *in vitro.* The cells were also successfully employed to study infection with other human pathogens with hepatic tropism, such as Hepatitis C and D viruses [8], [9].

HBV infection is a leading cause of liver disease, [10] more than 500,000 patients dying annually of serious complications, such as cirrhosis and carcinoma. Studies of HBV infection have been limited by the lack of a cell culture system able to support productive entry *in vitro*, in an efficient and reproducible manner [11]. Human and tupaia primary hepatocytes are susceptible to HBV infection, however, the variability of the origin and the low viability in culture significantly hamper their use *in vitro* [12]. The development of the HepaRG cell line has overcome many of these disadvantages, allowing for more systematic studies on viral-host cell interactions leading to the discovery of key cellular factors involved in HBV infection [13]–[16]. However, many aspects of the life cycle, particularly the early steps, remained unexplored.

Evidencing subtle changes of the expression pattern of a set of proteins from a particular cell, tissue, organ or organism is now possible with the tremendous development of the proteomics technology [17], [18]. Classical proteomics aims at identifying proteins from two samples and detect the expression differences [18]–[20]. Therefore, comparing the proteomes in proliferating and quiescent HepaRG cells can provide insights on the function of some proteins that are differentially synthesized, regulated or degraded and identify signaling mechanisms associated with differentiation. So far, two proteomics studies have been published on HepaRG cells. The first analysis compared the protein pattern between naive and HBV-infected HepaRG cells [21]. The second study performed by our group, intended to identify plasma membrane (PM) proteins in non-differentiated (ND) and differentiated (D) cells; however only a limited number of differentially expressed proteins were found in this preliminary approach [22].

Performing proteomics on subcellular fractions and organelles rather than whole cells is particularly relevant, as low abundant proteins can be significantly enriched; moreover, proteins trafficked between different compartments, such as the cytoplasm and the PM, can be identified. Here, we continued our proteomics studies of the PMs of (ND) and (D) HepaRG cells, aiming not only to improve the list of differentially regulated proteins, but importantly, to further investigate potential alterations of their intracellular distribution within hepatocyte and biliary cell populations. Following purification, the PM fraction was significantly enriched compared to the total cell lysates, allowing the identification of a new series of proteins, unreported previously. Several proteins with unexpected PM localization, undergoing most significant changes of expression, such as Cathepsins D and K, Cyclophilin A, Annexin A1 and several disulfide isomerases with a potential role in cell-pathogen interaction were further investigated by Western blotting. Moreover, the distribution of these proteins into hepatocyte and biliary cells was monitored by confocal microscopy. Overall, we found important differences between the proteomes of the PMs of (ND) and (D) cells and profound changes of the intracellular localization of proteins upon differentiation. Taken together, these results advance our current understanding of the biology of HepaRG cells, the molecular changes underlying their differentiation and the unique susceptibility for hepatic viruses infection. Similar to primary hepatocytes, HepaRG cells are difficult to manipulate genetically once differentiated, making functional investigations very laborious. In this respect, the panel of differentially regulated plasma membrane proteins provided by this work becomes an important tool for future analysis of selected targets.

## Materials and Methods

### Chemicals

All chemicals were purchased from Sigma-Aldrich, unless mentioned otherwise.

### Cell Culture and Differentiation

HepaRG cells (kind gift from Dr. David Durantel, INSERM U871, Lyon, France, [23]) were grown in T75 flasks, maintained and differentiated as previously described [22]. The typical cell morphology associated with differentiation was constantly monitored under the microscope and the up-regulation of albumin and aldolase B mRNAs was confirmed at the end of the differentiation process [1]. Cells were differentiated in collagen-coated 6-well plates and infected with HBV as described [14], [16]. When indicated, Pepstatin A (36 µM) was added to the cells during infection, or following removal of the viral inoculum. HBV transcripts were quantified in infected cells by reverse transcription (RT)-real time PCR, as published previously [14],[16].

### Preparation of PM from HepaRG Cells

PMs were obtained from an equal number of (D) and (ND) HepaRG cells using an optimized cell fractionation protocol described previously in detail [22], with a minor modification. Briefly, only two thirds of the PM ring obtained after the subcellular fractionation was collected, to ensure a higher purity of the preparation and a 3 KDa cut-off membrane was employed to concentrate the sample, in order to retain as many proteins as possible. Two independent experiments run in duplicates were performed. In some experiments, PM was also purified form (D) HepaRG cells infected with HBV, as described [14], [16]. Purification of the PM fractions was further validated following comparative analysis of expression of different organelle markers, in purified samples and total cell lysates (TL), by Western blotting.

### SDS-PAGE and Western Blotting

The protein content was quantified in TL and PM samples using the BCA method (Pierce). Sample volumes containing equal amounts of proteins were boiled for 5 min in Laemmli buffer and loaded on SDS-PAGE. The proteins were stained with Coomassie blue or transferred to PVDF membranes (GE Healthcare) using a semi-dry blotter (Millipore). The blots were incubated with either Ponceau S Red or specific antibodies (Abs): rabbit anti-Rab7 (1∶1000), from Cell Signaling Technology; goat anti-CD71 (1∶1000), goat anti-Calnexin (1∶1000), mouse anti-Lamp1 (1∶200), mouse anti-Ezrin (1∶200), rabbit anti-Annexin A1 (1∶100) and goat anti-Grp78 (1∶500), all from Santa Cruz Biotechnology; mouse anti-PDI (1∶1000), mouse anti-Cathepsin D (1∶1000), rabbit anti-Cathepsin K (1∶1000), rabbit anti-eyes absent (Eya) phosphatase (1∶1000) and rabbit anti-cyclophilin A (1∶1000), all from Abcam; rabbit anti-PDI A4 (1∶4000), from Antibodies-online. The secondary Abs used were goat anti-mouse, goat anti-rabbit horseradish peroxidase (HRP) (Pierce, dilution 1∶1000) or donkey anti-goat HRP (Santa Cruz Biotechnology, dilution 1∶5000). Proteins were visualized using the ECL (GE Healthcare) detection system.

### Protein Digestion and Peptide Extraction

The gels were cut in three pieces containing high (80–200 kDa), middle (35–80 kDa) and low (0–35 kDa) molecular mass proteins. Two sets of proteins per condition (ND and D) were analyzed for each of the two independent PM isolation experiments and three LC-MS/MS runs were applied per set of proteins. The gel pieces were digested according to published procedures [22], [24]. Briefly, the gel pieces were washed several times in HPLC grade water for 10–15 min, then cut into very small fragments and dehydrated by incubation in 50 mM ammonium bicarbonate (ABC), 50 mM ABC/50% acetonitrile (ACN), and 100% ACN. After the last incubation step, the fragments were dried in a Speed-vac concentrator and washed again in 50 mM ABC. The dried samples were then rehydrated in 50 mM ABC containing 10 mM DTT, for 45 minutes at 56°C then incubated in 50 mM ABC containing 100 mM iodoacetamide, for 45 minutes in the dark. The initial washing procedure was repeated once, followed by Speed-vac drying and overnight incubation in10 ng/µL trypsin in 50 mM ABC, at 37°C. The resulting peptides were extracted by incubation in 5% formic acid (FA)/50 mM ABC/50% ACN then 100% ACN. The peptides mixtures were combined, dried as above and solubilized in 20 µL of 0.1% FA/2% ACN in HPLC water.

### Nanoliquid Chroµatography-tandem Mass Spectrometry (LC-MS/MS)

The samples were analyzed by LC-MS/MS using a NanoAcuity UPLC (Micromass/Waters, Milford, MA) coupled to a Q-TOF Micro MS (Micromass/Waters, Milford, MA) [22], [25], [26]. The peptides were loaded onto a 100 µm×10 mm nanoAquity BEH130 C18 1.7 µm UPLC column (Waters, Milford, MA) and eluted over a 150 min gradient of 2–80% organic solvent (ACN containing 0.1% FA) at a flow rate of 400 nL/min. The aqueous solvent was 0.1% FA in HPLC water. The column was coupled to a Picotip Emitter Silicatip nano-electrospray needle (New Objective, Woburn, MA). MS data acquisition involved survey MS scans and automatic data dependent analysis (DDA) of the highest intensity ions with the charge of 2+, 3+ or 4+. The MS/MS was triggered when the MS signal intensity exceeded 10 counts/second. In survey MS scans, the three most intense peaks were selected for CID and fragmented until the total MS/MS ion counts reached 10,000 or for up to 6 seconds each. Calibration was performed for both precursor and product ions using 1 pmol GluFib (Glu1-Fibrinopeptide B) standard and the monoisotopic doubly-charged peak with m/z of 785.84.

### Data Processing and Protein Identification

The raw data were processed using ProteinLynx Global Server (PLGS, version 2.4) software as previously described [22]. The following parameters were used: background subtraction of polynomial order 5 adaptive with a threshold of 30%, two smoothings with a window of three channels in Savitzky-Golay mode and centroid calculation of top 80% of peaks based on a minimum peak width of 4 channels at half height. The resulting pkl files were submitted to the public Mascot database search (www.matrixscience.com, Matrix Science, London, UK; version 2.4.1) for protein identification, using the following parameters: human databases from SwissProt_2013_05 containing 20256 entries, parent mass error of 1.3 Da, product ion error of 0.8 Da, enzyme used: trypsin, one missed cleavage, carbamidomethyl-Cysteine as fixed modification and Methionine oxidized as variable modification. In addition, the pkl files were searched against in-house PLGS database version 2.4 (www.waters.com) using similar parameters as for the Mascot search. Only the peptides that were identified with a Mascot score higher than 25 were considered. A second Mascot database search was further performed as above, using more stringent parameters (a fragment ion mass tolerance of 0.6 Da and a parent ion tolerance of 1.3 Da). The Mascot results were then exported as.dat files and built into a Scaffold file using Scaffold software version 4.0.4 (Proteome Software Inc., Portland, OR) and the proteins were further classified according to their molecular function, subcellular localization and involvement in different biological processes. Peptide identifications were accepted when established with more than 79.0% probability by the Scaffold Local FDR algorithm. Protein identifications were accepted when established with more than 95.0% probability (FDR less than 1.0% and containing at least 1 identified peptide).

### Immunocytochemistry and Confocal Microscopy

Cells were differentiated on cover glass, fixed with 4% paraformaldehyde and washed with PBS. Following permeabilization with 0.2% Triton-X100, samples were incubated for 30 min with specific Abs: mouse anti-albumin, rabbit anti-Annexin A1 (both from Santa Cruz Biotechnology, dilution 1∶50 and 1∶100, respectively), mouse anti-PDI, mouse anti-Cathepsin D, rabbit anti-Cathepsin K, rabbit anti-Cyclophilin A (all from Abcam, dilution 1∶1000) and rabbit anti-PDI A4 (Antibodies-online, dilution: 1∶200). Cells were washed with PBS and further incubated for 30 min with secondary Alexa Fluor 488 goat anti-mouse or Alexa Fluor 594 goat-anti rabbit Abs (Invitrogen, dilution1∶400). The samples were mounted with Vectashield Mounting Medium (Invitrogen) containing DAPI (4, 6-diamidino-2-phenylindole) to visualize the nuclei. Images were taken with a Zeiss LSM 710 laser scanning confocal microscope using a 63x objective and further processed using the ZEN software.

## Results and Discussion

### Purification of PM from HepaRG Cells

Despite their crucial functions in intra- and extracellular signaling pathways, ion transport, cell-cell and cell-environment interactions, PM proteins remain significantly under-represented in proteomics studies, mainly because of their low abundance. To ensure a comprehensive MS analysis of the differentially expressed PM proteins in HepaRG cells, enriched fractions isolated from (ND) and (D) cells were used.

The procedures for purification of the PMs was previously described by our laboratory [22] and further optimized in this study. The PM fraction was purified from an equal number of (ND) and (D) cells in two independent experiments run in duplicate samples. The proteins were extracted and separated by SDS-PAGE and the gels were stained with Coomassie dye (Fig. S1, A) or transferred on PVDV membranes and stained with Ponceau S (Fig. S1, B). Consistent with our previous observation [22], a clear difference between the protein patterns of the PMs from (ND) and (D) cells was noticeable. Overall, more proteins were extracted from the PMs of (D) cells (Fig. S1). This behavior was observed before and explained by an increased PM fluidity following the prolonged treatment of HepaRG cells with 1.8% DMSO required for differentiation [22].

**Figure 1:**
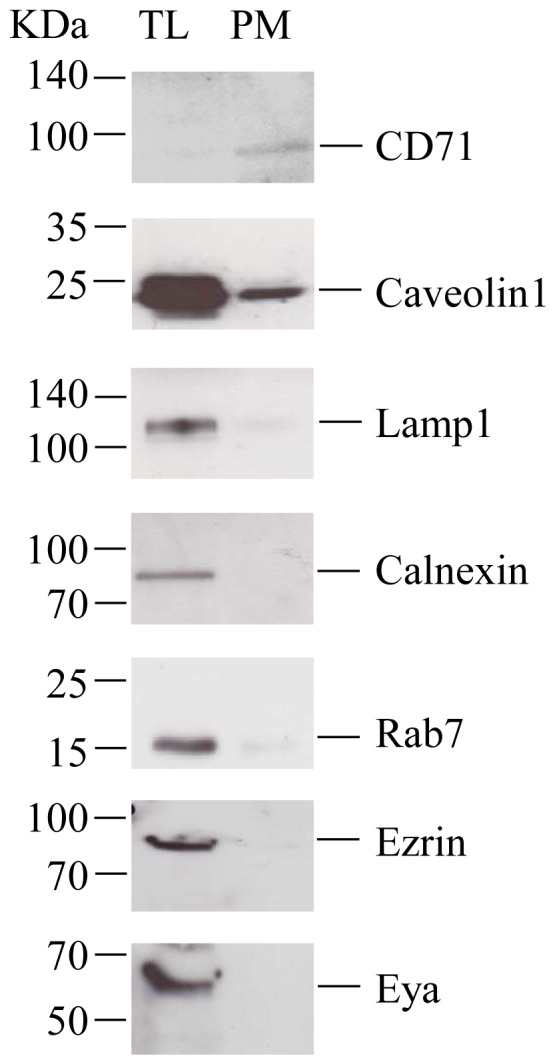
Validation of the plasma membrane (PM) purification. Equal amounts of proteins from total HepaRG cell lysate (TL) and purified PM fraction were loaded on SDS-PAGE and analyzed by Western blotting using organelle-specific Abs. The molecular weight markers are indicated. The representative blot of three independent experiments is shown.

To determine the purity of the PM fractions, a series of well-established organelle markers was identified by Western blotting in both, PM and TL samples. As shown in Fig. 1, CD71 (also known as the human transferrin receptor-1), an integral membrane protein that mediates the uptake of transferrin-iron complexes, was almost exclusively detected in the PM sample, in agreement with its previously reported localization on the surface of particular cells, including the hepatocytes [27]. This suggests an important enrichment of the PM-characteristic proteins in this fraction, compared to the total cell content. Unlike CD71, Caveolin1 was present in both, PM and TL cells. This behavior is expected, since this scaffolding protein is associated with many intracellular membranes but also with specific PM domains, the lipid rafts, and is highly expressed in HepaRG cells [14]. In contrast, the endoplasmic reticulum (ER) marker, Calnexin, was absent in the PM fraction while readily detectable in TL (Fig. 1). Similarly, the lysosomal marker Lamp1 and the cytoplasmic/endosome-associated Rab7 protein, were present in TL and only in trace amounts in PM fractions. The purity of the PM fractions was further checked by determining the expression of the Eyes absent (Eya) protein, a cytoplasmic phosphatase also functioning as a transcription factor, when translocated to the cell nucleus [28] and that of Ezrin, an actin-binding protein of the ezrin/radixin/moesin (ERM) family [29]. Both proteins were detected in TL, but not in the PM fraction (Fig. 1). Together, the results show the lack of significant contamination of the PM preparations and their suitability for further MS analysis.

### LC-MS/MS Analysis

**Figure 2:**
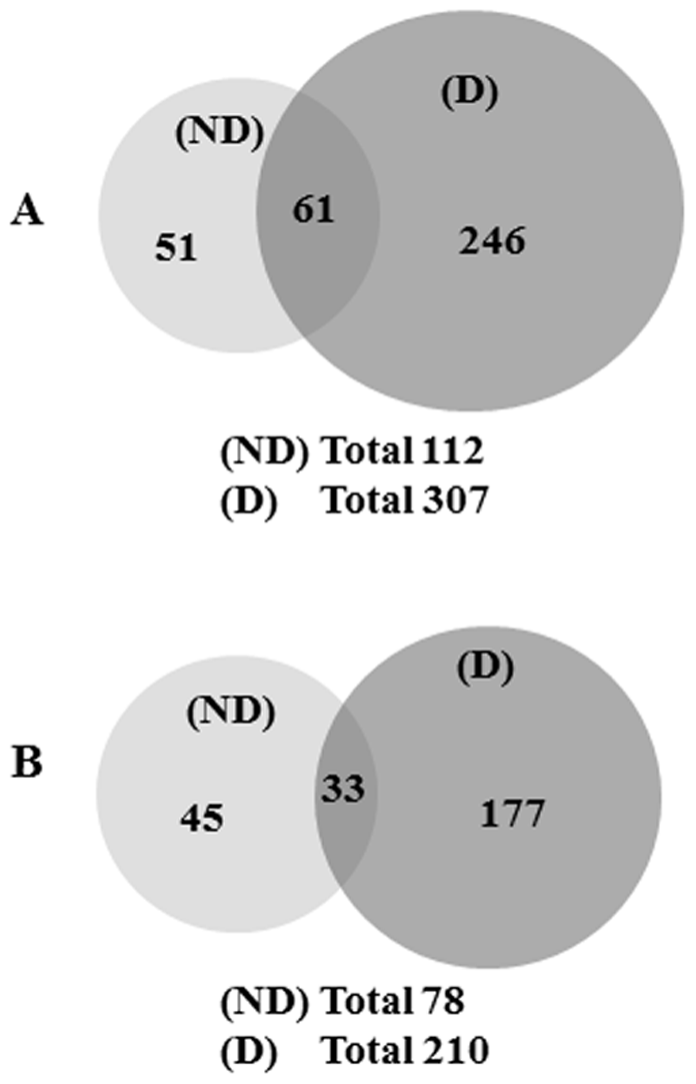
Summary of the proteomic analysis of the PM from undifferentiated (ND) and differentiated (D) HepaRG cells. (A and B) SDS-PAGE resolved proteins were analyzed by LC-MS/MS as described. Venn diagrams show the number of proteins identified in PM from (ND) and (D) HepaRG cells in experiments 1 (A) and 2 (B).

To identify the proteins from the PMs of (ND) and (D) cells, the gels were cut in three small pieces per condition, digested with trypsin and analyzed independently by LC/MS/MS, resulting three Mascot scores. The indication of one Mascot score shows that the corresponding protein was identified in only one of the three gel bands analyzed. An outcome of these experiments is summarized in Fig. 2 (A and B). Specifically, 307 and 112 proteins were identified in the PMs of (D) and (ND) cells, respectively, in experiment 1. Of these, 61 proteins were shared between the two conditions (Fig. 2A). In experiment 2, 210 and 78 proteins were identified in the PM of (D) and (ND) cells, respectively, while 33 of them were common between the two conditions (Fig. 2B). The complete lists of proteins identified in the two independent experiments are shown in Tables S1 and S2. As observed in these tables, some proteins were identified by only one peptide. The MS/MS spectra for these peptides are shown in Figs. S2 and S3. The second Mascot database search performed using more stringent parameters (a fragment ion mass tolerance of 0.6 Da and a parent ion tolerance of 1.3 Da) resulted in a similar protein distribution profile in (D) and (ND) cells, except that a lower number of proteins were identified (data not shown).

**Figure 3:**
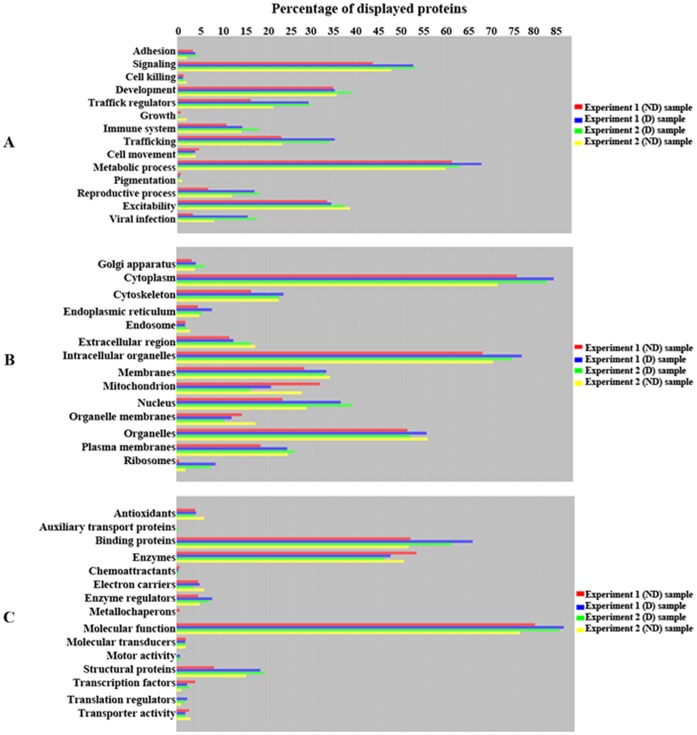
Classification of the proteins identified at the PM of (ND) and (D) HepaRG cells. The mascot search results from experiments 1 and 2 were exported as.dat files and processed with the license-based Scaffold 4.0 software (www. proteomescience.com). The proteins identified in (ND) and (D) cells were classified according to their involvement in a biological process (A), subcellular localization (B) and molecular function (C).

The proteins were further classified using the Scaffold 4.0 software, according to their biological role (Fig. 3A), subcellular localization (Fig. 3B) and molecular function (Fig. 3C). Interestingly, overall, (D) cells expressed more proteins involved in intracellular trafficking and transport regulation, a consistent result between the two independent experiments (Fig. 3A). Similarly, proteins reported to have a role in viral infections, as well as signaling molecules, were reproducibly increased in (D) samples (Fig. 3A). The list of proteins with a role in viral reproduction, as resulted from this analysis, is shown in Table S3. Cytoplasmic, nuclear and ribosomal proteins appear up-regulated in (D) cells in both experiments (Fig. 3B), which could reflect an enhanced rate of protein synthesis, coupled with increased expression of translational regulators, binding proteins and molecular chaperones (Fig. 3C). An important fraction of proteins identified in PM preparations were cytoplasmic and organelle-associated. Although an unwanted contamination of the PM with these fractions cannot be completely excluded, these proteins may well arise from subcellular vesicles or cytoplasmic proteins that are in close contact with the PM, or are shuttled between different compartments and the PM, as shown in an independent study [30].

Since PM is the first barrier encountered by pathogens before infection of the host cell, we next focused on proteins that are possible interaction partners with viruses [31]–[33] that will be discussed below.

### Proteins that are Significantly Up-regulated in (D) Cells

Strong differences of the Mascot scores between PM proteins from (D) and (ND) cells were obtained for Cathepsin D, Cyclophilins A and B, HSP 90-beta (HSP90AB1 or HSP90B), Endoplasmin (HSP90B1), HSP 90-alpha 2 or HSP alpha 2 (90 kDa HSP alpha 2 or HSP90AA1 or HSP90A), Annexins A1, A2 and A5, Protein Disulfide-Isomerases (PDI), Protein Disulfide-Isomerase A4 (PDI A4 or ERp72) or Carboxylesterase.

For example, in experiment 1, Annexin A1 was identified with Mascot scores of 360 and 397 in the PMs of (D) cells and was absent in (ND); in experiment 2, the Mascot scores of the protein were 222 in (D) and 109 in (ND) cells. A similar behavior was observed for Annexin A2, A4 and A5. It has been shown that Annexins expression is regulated both temporally and spatially during rat hepatocyte differentiation, significant changes being observed in Annexin A1 and A2 levels [34]. Owing to their specific expression pattern and localization in differentiating hepatocytes, Annexins were suggested to play unique roles at different stages of liver ontogeny. Annexins were also involved in the life-cycle of many viruses. For example, Annexin A2 interacts with various viruses [35], [36], while Annexin A6 was recently reported as a novel, negative regulator of influenza A virus infection, by directly interacting with the viral M2 protein [37]. The liver Annexin A5 was linked to an increased susceptibility to HBV infection [38]; however, this function was not confirmed in more recent infectivity models.

**Figure 4:**
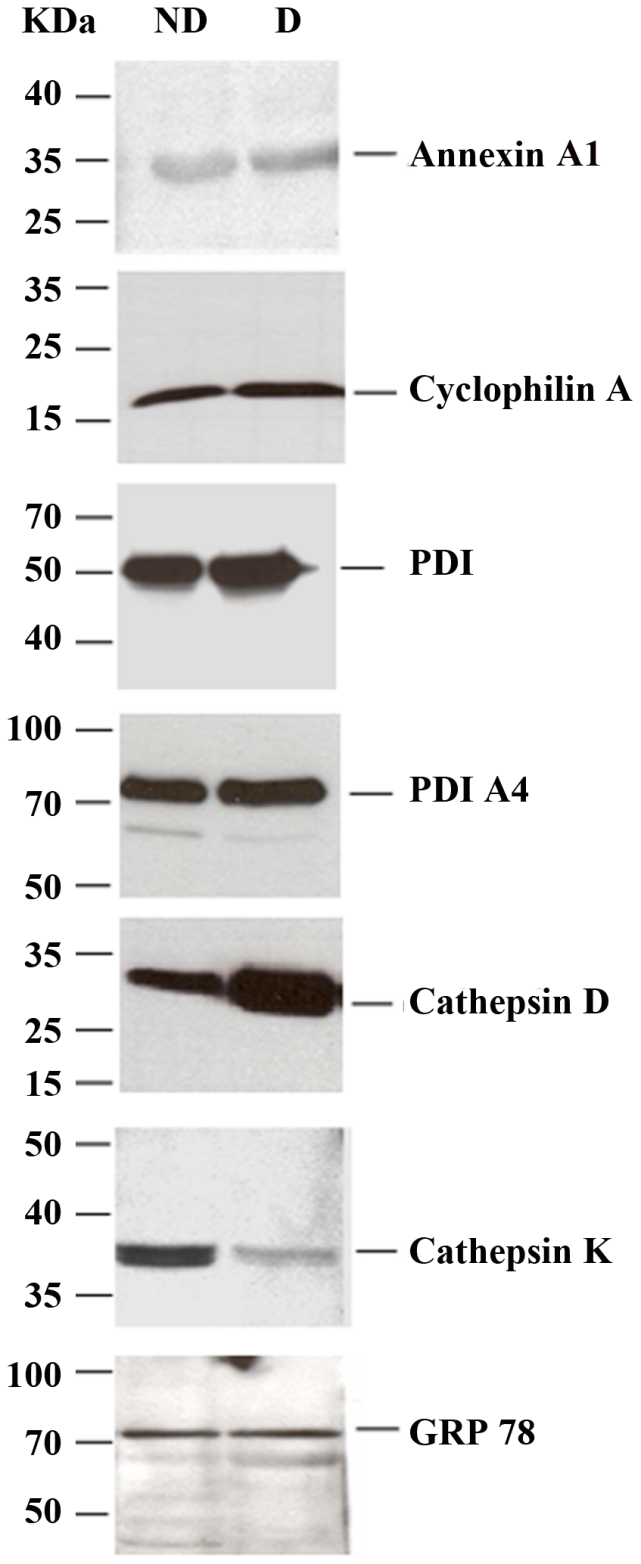
Analysis of differentially expressed proteins identified at the PM of (ND) and (D) HepaRG cells. The PM fractions of (ND) and (D) cells were analyzed by Western blotting using Abs specific for proteins with significantly altered levels of expression following differentiation. The molecular weight markers are indicated.

Interestingly, using the Search Tool for the Retrieval of Interacting Genes (STRING) Annexins are also predicted to interact with each other [39], [40]. Although identification of high amounts of these proteins in differentiated HepaRG cells may not be surprising, it is important to note that their PM localization has not been reported before. Moreover, in a previous proteomics study performed on whole HepaRG cells, Annexin A1 was found in HBV-infected, but not naïve cells [21]; this observation strongly supports the notion that organelle proteomics is a powerful tool to identify scarce proteins or those having multiple subcellular localizations. PM localization of Annexin A1 was further confirmed by Western blotting using specific Abs in both naïve (Fig. 4) and HBV-infected HepaRG cells (Fig. S4). Collectively, these data suggest that Annexins are prime candidates for pathogen-host cell interaction and might be relevant for the susceptibility of HepaRG cells to viral infection.

Cyclophilins had an expression pattern similar to Annexins. In experiment 1, Cyclophilin B was identified with Mascot scores of 312 and 129 in the PMs from (D) and (ND) cells, respectively, but was absent in experiment 2. Unlike Cyclophilin B, Cyclophilin A was identified in the PM of (D) cells, with very high Mascot scores (897 in experiment 1 and 765 in experiment 2). This increased expression was also demonstrated by Western blotting (Fig. 4). Cyclophilins are members of the immunophilin superfamily with peptidyl-prolyl cis-trans isomerase activity. The proteins were involved in protein folding, cell signaling and replication of several viruses, according to their specific localization in cellular compartments including the cytosol, ER, mitochondria and nucleus. It was shown recently that Cyclophilin A inhibits replication of influenza virus by interacting with the M1 protein. Moreover, the protein was found into purified virions [41]. Cyclophilin A was also involved in the HBV life-cycle, expression of the small surface antigen (SHBs) promoting its secretion, which in turn, may contribute to the pathogenesis of HBV infection [42]. However, this is the first report demonstrating that important amounts of cyclophilins are present at the PM of (D) HepaRG cells, suggesting a potential role in virus internalization, as well as secretion.

Other examples of up-regulated proteins at PM of (D) cells are PDI and PDI A4. In experiment 1, PDI was identified with Mascot scores of 584, 208 and 88 in (D), versus 114 in the (ND) cells. In experiment 2, PDI was identified with Mascot scores of 200 and 107 in (D) and (ND) cells, respectively. The mascot scores for PDI A4 were 103 and 116 (D) cells in experiments 1 and 2 and only 89 in (ND) cells. This enhanced expression of the PDIs was confirmed by Western blotting in both, naïve (Fig. 4) and HBV-infected HepaRG cells (Fig. S4). PDIs belong to a family of structurally related enzymes involved in disulfide bonds formation, reduction, or isomerization during protein folding within the ER. However, they have been shown to play important roles in biological processes at unusual intracellular locations, such as PM, cytosol and nucleus [43]. For instance, the PDI reducing activity at the cell surface was required for internalization of certain viruses, such as Dengue, as recently reported [44]. Interestingly, we have shown that HBV productive infection of HepaRG cells also depends on the reducing milieu provided by the endocytic pathway, likely involving the processing of the disulfide-linked envelope proteins in a catalytic process which remains to be identified [16].

Similar to PDI, Cathepsin D was identified in experiment 1 with Mascot scores of 276, 159 and 76 in (D), versus a Mascot score of 99 in (ND) cells. In experiment 2, the protein was identified with Mascot scores of 200 and 107 in (D) and (ND) cells, respectively. High levels of Cathepsin D at PM of (D) cells were also detected by Western blotting (Fig. 4). Traditionally localized in the endo-lysosomal compartment, the presence of Cathepsin D at the PM of HepaRG cells may appear highly unusual. However, PM binding has been previously shown for Cathepsin B, another member of the Cathepsin family of proteins, occurring via interaction with alpha 2-macroglobulin [45]. To rule out a potential contamination of the PM with the endo-lysosomal compartment, the presence of Cathepsin K, a lysosomal-resident cysteine protease that was not identified in our MS experiments, was also investigated by Western blotting. Unlike Cathepsin D, the relative levels of this protein decreased in the PMs of the (D) compared with (ND) cells (Fig. 4). In addition, the other lysosomal-associated proteins, Lamp1 and Rab7, were also absent from the PM (Fig. 1), strongly suggesting that Cathepsin D transport to PM is not artifactual, but rather a requirement to fulfill a specific function in (D) cells. A potential role in HBV infection was addressed in a preliminary study using Pepstatin A, an inhibitor specifically interacting with Cathepsin D [46]. Interestingly, the drug inhibited HBV infection by about 40% when added to the cells during viral inoculation, but not at later points (Fig. S5). This suggests a possible role for Cathepsin D at very early steps of the viral life-cycle, which deserves more comprehensive, future investigations.

### Proteins that are Present Exclusively in the PM of the (D) Cells

Within this category, HSP 90-beta (HSP90AB1 or HSP90B) was identified with Mascot scores of 477 (experiment 1) and 523 (experiment 2) in the PMs of (D) cells, but not in the (ND) cells. Similarly, Endoplasmin (HSP90B1) was identified with Mascot scores of 452 (experiment 1) and 305 (experiment 2) only in the PMs of (D) cells. HSP alpha 2 (90 kDa HSP alpha 2 or HSP90AA1 or HSP90A) was identified with Mascot scores of 362 and 112 (experiment 1) and 478 (experiment 2) in the PMs of (D), but not (ND) cells. These proteins are members of the 90 kDa HSP family, involved in a variety of functions [47]–[49], including a specific interaction with HBV [48]; importantly, they are also key players in building stable and transient protein-protein interactions, either as homo- or heterodimers [47], [49]–[51]. The significant localization of these proteins at PM of (D) but not (ND) cells makes them promising candidates for functions restricted to differentiated HepaRG cells.

### Proteins with Similar Expression in (ND) and (D) Cells

For some proteins the results were more difficult to interpret. For example, the Glucose Regulated Protein 78 kDa HSP (GRP78 or BiP) was identified with a Mascot score of 502 and 57 at PMs of (D) cells, versus a Mascot score of 113 in (ND) cells. In addition, in experiment 2, the protein was identified with a Mascot score of 109 in (D) versus 127 and 110 in (ND) cells. The Western blotting analysis also revealed similar amounts of GRP78 at the PM of HepaRG cells, in both conditions (Fig. 4). Identification of GRP78 in our experiments may be the result of its known interactions with other members of the HSP70 and HSP90 families of proteins and other proteins, as well [49], [52]. In the light of recent literature [53], [54], these data may be interpreted as GRP78 being a member of a large multimeric complex at the PM of (D) cells.

Taken together, these data validate the LC-MS/MS experiments and further reinforce the hypothesis of a potential role played by the differentially expressed proteins in virus-cell interaction.

### Relative Quantitation of the Proteins from PMs of (ND) and (D) Cells

**Figure 5:**
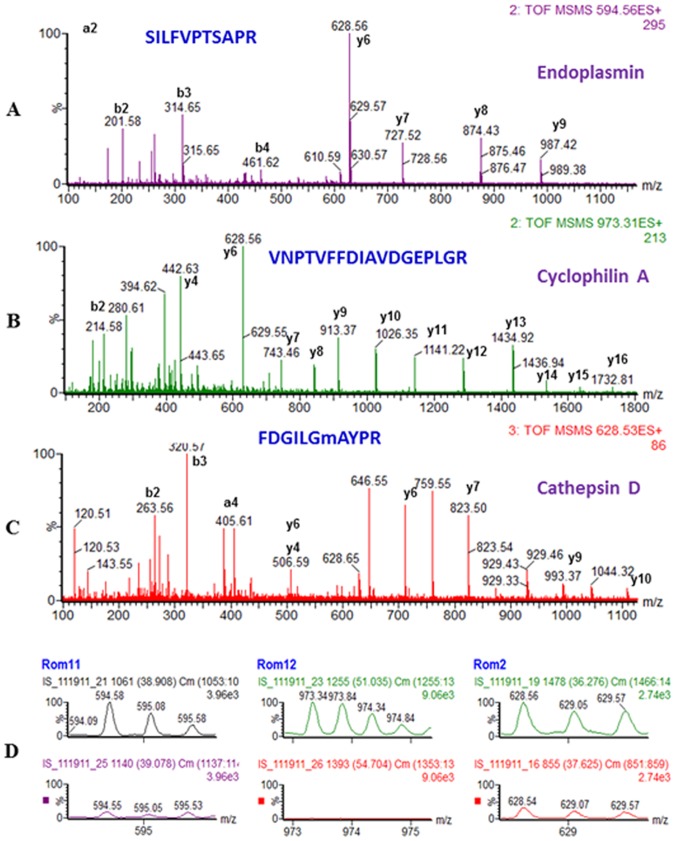
Identification and relative quantification of Endoplasmin, Cyclophilin A and Cathepsin D. (A) A doubly-charged peak at m/z of 594.56 (D) was fragmented by MS/MS and produced a series of b and y peaks shown in the MS/MS spectrum leading to identification of the Endoplasmin-derived SILFVPTSAPR peptide. (B) A double-charged peak at m/z of 973.31 (D) was fragmented by MS/MS and produced a series of b and y peaks shown in the MS/MS spectrum leading to identification of the Cyclophilin A-derived VNPTVFFDIAVDGEPLGR peptide. (C) A double-charged peak at m/z of 628.53 (D) was fragmented by MS/MS and produced a series of b and y peaks shown in the MS/MS spectrum leading to identification of the Cathepsin D-derived FDGILGmAYPR sequence (m denoted oxidized methionine. (D) Comparison of the intensities of MS spectra for the doubly charged precursor peaks with m/z of 594.58, 973.34 and 628.56 corresponding to peptides derived from Endoplasmin (A), Cyclophilin A (B) and Cathepsin D (C). The intensity scale for the spectra from both (D) (upper panels) and (ND) cells (lower panels), for each individual peptide was identical.

Mascot scores and other MS methods used for protein quantitation, such as PAI or emPAI score [25], [26], [55] are good indicators of the relative abundance of a protein in a sample. Nevertheless, to conclude about the relative quantitation, more appropriate approaches must be employed, such as spectral counting or comparison of the relative intensity of the precursor ions from two samples corresponding to a particular peptide, over a specific amount of time. To confirm the semi-quantitative results determined by the Mascot score, in a more accurate manner, we compared the relative intensity of precursor ions which corresponded to peptides derived from Cyclophilin A, Endoplasmin and Cathepsin D (Fig. 5). We identified Cyclophilin A based on 25 MS/MS spectra and an emPAI score of 5.85 in the PMs of (D) cells and only 3 MS/MS spectra and an emPAI score of 0.47 in (ND) cells. Endoplasmin was identified in the PM of (D) cells based on 21 MS/MS spectra and an emPAI score of 0.60, and only one MS/MS spectrum and an emPAI score of 0.04 in (ND) cells. Finally, Cathepsin D was identified in the PM of (D) cells based on 10 MS/MS spectra and an emPAI score of 1.55 and 6 MS/MS spectra and an emPAI score of 0.49 in (ND) cells. We further compared the intensity of precursor ions for the peptides shown in Fig. 5A–C. As observed in Fig. 2D, there was a higher intensity for the peak with m/z of 594.58 (2+) that corresponds to the Endoplasmin-derived SILFVPTSAPR peptide (Fig. 5A) in the PMs from the (D) cells (upper panel), compared with (ND) cells (lower panel). A similar behavior was observed for the peak with m/z of 973.34 (2+) corresponding to the Cyclophilin A-derived VNPTVFFDIAVDGEPLGR peptide (Fig. 5B) and the peak with m/z of 628.56 (2+) that corresponds to the FDGILGmAYPR peptide (m stands for methionine oxidized) derived from Cathepsin D (Fig. 5C).

The proteins of the Annexin family (Annexin A1, A2, A4 and A5) were also quantitatively analyzed, by comparing the total number of spectra as well as the relative intensity of the precursor ions. As observed in Fig. S6, the relative number of MS/MS spectra corresponding to either Annexins was higher in the PM of (D) compared to (ND) cells. This trend was reproduced in both experiments and further confirmed by the comparison of the relative intensity of the precursor ions (Fig. S6).

### Distribution of Differentially Expressed Proteins in Hepatocyte- and Biliary-like Cells upon HepaRG Differentiation

**Figure 6:**
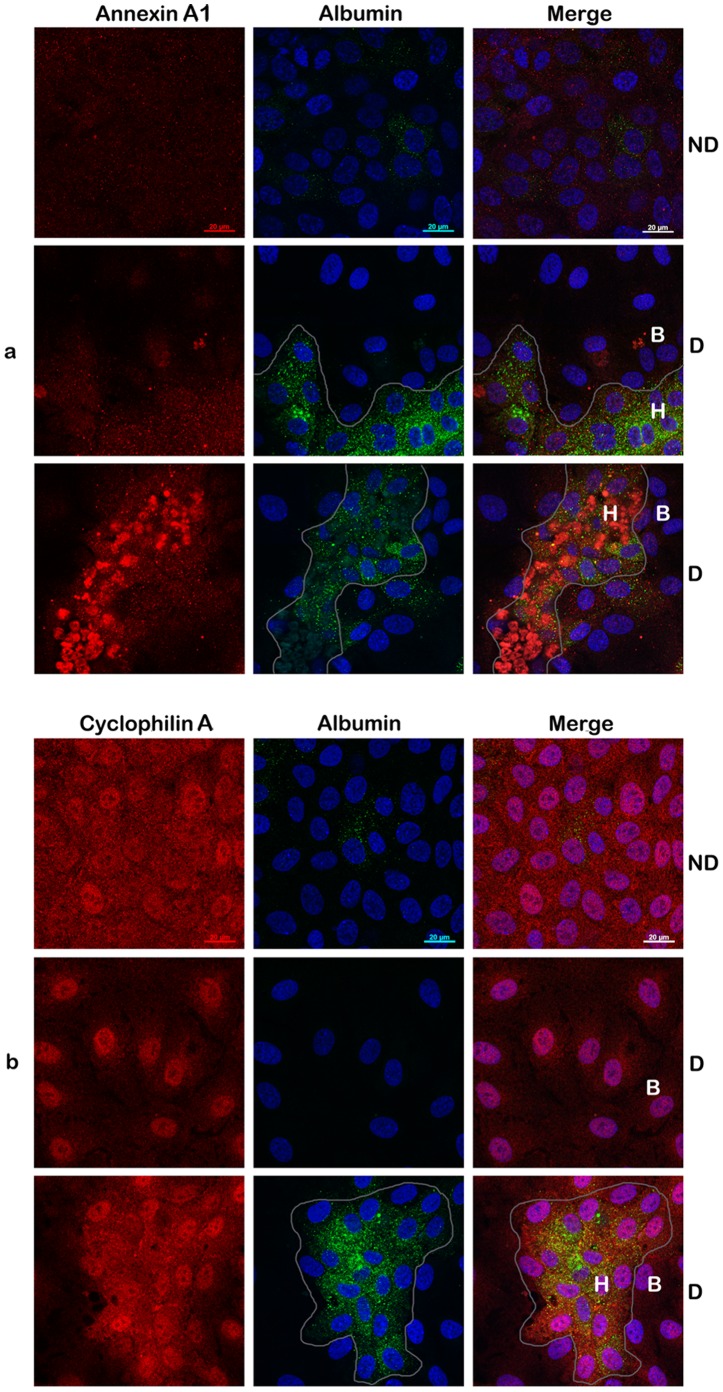
Analysis of differentially expressed proteins in hepatocyte- and biliary-like HepaRG cells (I). Cells were seeded on coverslips in 6-well plates and either differentiated (D) or maintained untreated, as control (ND). Expression of Annexin A1 (panel a) and Cyclophilin A (panel b) in hepatic (H) and biliary (B) cells was evidenced using specific Abs. Albumin expression was also investigated as a marker for differentiation to adult hepatocytes (panels a and b). Samples were analyzed with a Zeiss LSM 710 confocal microscope following mounting with Vectashield Mounting Medium containing DAPI, to visualize the nuclei (blue). Images were taken with the 63x objective and processed with ZEN software. Scale bar is 20 µm.

The PM purification and the LC-MS/MS experiments were performed using the whole population of differentiated HepaRG cells. However, the fraction of hepatocyte-like cells may vary as much as 45–90% between different HepaRG batches and passages [15]. Thus, it was important to determine how these differences in expression of the proteins validated above are reflected at the level of each, hepatocyte and biliary cell populations. To this aim, dual immunolabelling and confocal microscopy was employed. The two cell populations obtained following DMSO treatment were discriminated based on: a) their different morphology; b) the up-regulation of albumin expression in hepatocyte, as opposed to biliary cells [1]. As shown in Fig. 6, Annexin A1 was hardly expressed in (ND) cells. Interestingly, upon differentiation, its level remained low in biliary (B) cells while it considerably increased in hepatocyte (H) islands (panel a). This suggests that the overall increase of the Annexin A1 amount observed in (D) cells, both by LC-MS/MS and Western blotting is exclusively due to its up-regulation in hepatocyte-like cells. An interesting trend was observed for Cyclophilin A, which is readily detectable in (ND) cells throughout the cytoplasm and toward the PM. This distribution profile is maintained in hepatocytes, while the protein expression becomes restricted to the nucleus in biliary cells (panel b). Thus, it is reasonable to assume that the higher amount of PM Cyclophilin A observed in (D) cells originates form hepatocyte, rather than biliary cells. Also, due to its different intracellular localization, it is likely that Cyclophilin A plays specific roles within the two cell types.

**Figure 7:**
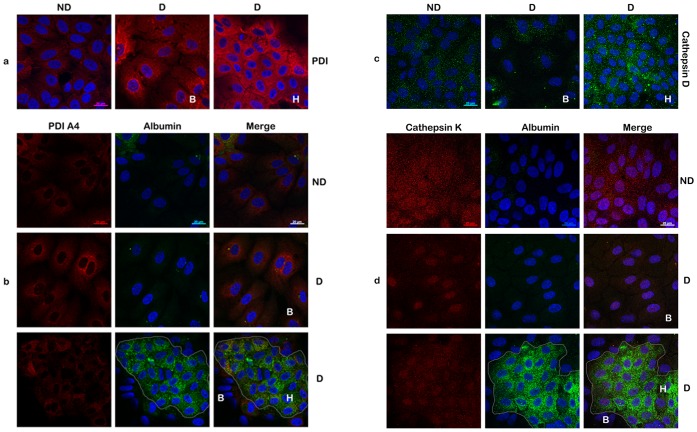
Analysis of differentially expressed proteins in hepatocyte- and biliary-like HepaRG cells (II). HepaRG cells were analyzed as in Figure 4, except that expression of PDI (panel a), PDI A4 (panel b), Cathepsin D (panel C) and Cathepsin K (panel d) were evidenced using specific Abs. Where possible, albumin expression was also evidenced (panels b and d).

A different trend was observed for the two PDIs investigated (Fig. 7), PDI (panel a) and PDIA4 (panel b); the level of both proteins increased significantly following differentiation, however to a similar extent in biliary and hepatic cells. While confirming the up-regulation observed by LC-MS/MS and Western blotting, the results also show that the increased PDI expression is not restricted to hepatocyte-like cells.

As shown by Western blotting, HepaRG differentiation had opposite effects on the expression of two members of the Cathepsin family (Fig. 7), inducing up-regulation of Cathepsin D (panel c) and down-regulation of Cathepsin K (panel d). Moreover, Cathepsin D expression increased in hepatocyte and remained constant in biliary cells (panel c), while Cathepsin K levels decreased in biliary cells and did not change in hepatocytes (panel d). Future experiments will address the significance of these changes and the potential role that these proteases may play in viral uncoating, genome release and initiation of productive HBV infection.

### Conclusions

In this study, we performed proteomics experiments to identify differences between the PMs from (ND) and (D) HepaRG cells. We found a significant alteration of the protein expression profile following differentiation, the PM localization of many of these proteins being reported for the first time. Importantly, the immunocytochemistry and confocal microscopy studies showed that this effect was different in biliary and hepatic cells, some of the proteins analyzed segregating between the two cell populations. Although further work is needeed to understand the observed changes, overall, the results reveal the complexity of the potential protein-protein interaction at the PM of differentiated cells and provide a strong basis for functional studies on selected targets, including identification of specific virus-host cell interactors.

## Supporting Information

Figure S1 **Total protein profile of PM isolated from (ND) and (D) HepaRG cells.** PM fractions were purified from an equal number of (ND) and (D) HepaRG cells. The proteins were extracted and analyzed by SDS-PAGE followed by Coomassie staining (A), or further transferred onto PVDF membrane and stained by Ponceau S (B). The molecular weight markers are indicated.
(TIF)

Figure S2 **MS/MS spectra of proteins identified by one peptide (I).** The proteins were found in PM of (ND) cells, in experiment 1 (Mascot scores higher than 25).
(PDF)

Figure S3 **MS/MS spectra of proteins identified by one peptide (II).** The proteins were found in PM of (D) cells, in experiment 1 (Mascot scores higher than 25).
(PDF)

Figure S4 **Analysis of differentially expressed proteins at PMs of (D) naïve and HBV-infected HepaRG cells.** The PM fractions of (D) cells infected (+) or not (−) with HBV were analyzed by Western blotting using Annexin A1- and PDI-specific Abs. The molecular weight markers are indicated.
(TIF)

Figure S5 **HBV infection in the presence of Cathepsin D inhibitors.** (D) HepaRG cells were treated with Pepstatin A for 24 h either during HBV infection (2) or following removal of the viral inoculum (3). HBV transcripts was quantified by RT-real time PCR at day 14 post-viral inoculation and the results were expressed as percentage from untreated cells (1). The error bars represent the SD between two independent experiments. Statistical analysis showing “p” values was performed using the Student’s unpaired t-test.
(TIF)

Figure S6 **Relative quantification of Annexins.** The number of spectra identified for Annexins A1, A2, A4 and A5 in (ND) and (D) HepaRG cells, in experiments 1 and 2 (A). Direct comparison of the precursor ions corresponding to peptides derived from Annexins (B). Precursor ion 851.92 (2+) and peptide GLGTDEDTLIEILASR, derived from Annexin A1; precursor ion 615.88 (3+) and peptide LSLEGDHSTPPSAYGSVK, derived from Annexin A2; precursor ion 846.95 (2+) and peptide GLGTDEDAIISVLAYR derived from Annexin A4; precursor ion 807.48 (2+) and peptide ETSGNLEQLLLAVVK.S, derived from Annexin A5. The red and green spectra correspond to proteins isolated from (ND) and (D) cells, respectively.
(TIF)

Table S1 **Summary of the LC/MS/MS analysis showing the proteins identified at the PM of (ND) and (D) cells (experiment 1).**
(XLS)

Table S2 **Summary of the LC/MS/MS analysis showing the proteins identified at the PM of (ND) and (D) cells (experiment 2).**
(XLS)

Table S3 **Proteins with a role in viral reproduction.** This classification resulted from the analysis of the Mascot results shown in Tables S1 and S2, using the Scaffold software-version 4.0.4.
(DOCX)
