# Supplementary figures and images for: Comparative Proteomics Reveals Novel Components at the Plasma Membrane of Differentiated HepaRG Cells and Different Distribution in Hepatocyte- and Biliary-Like Cells
Source: PLoS One. 2013 Aug 20;8(8):e71859. doi: 10.1371/journal.pone.0071859 (PMC3748114; doi:10.1371/journal.pone.0071859)

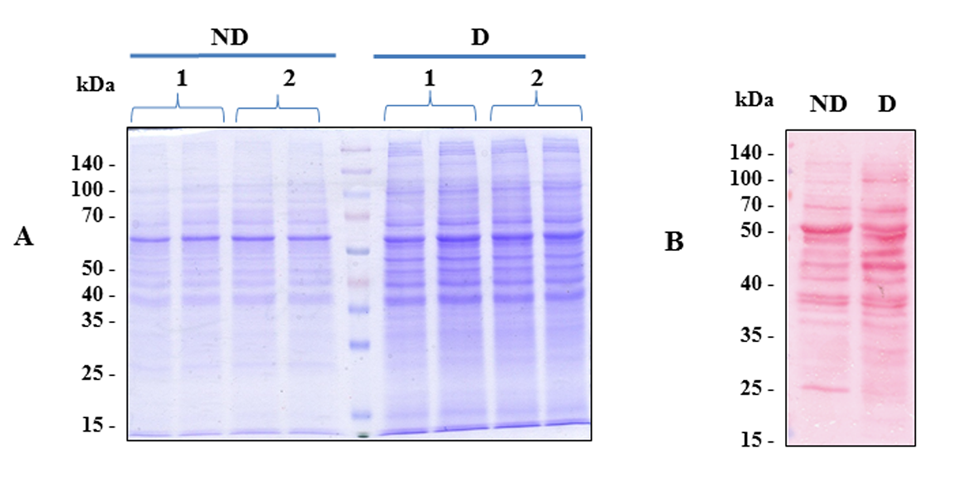

Supplement: Figure S1 — Total protein profile of PM isolated from (ND) and (D) HepaRG cells. PM fractions were purified from an equal number of (ND) and (D) HepaRG cells. The proteins were extracted and analyzed by SDS-PAGE followed by Coomassie staining (A), or further transferred onto PVDF membrane and stained by Ponceau S (B). The molecular weight markers are indicated. (TIF) [file pone.0071859.s001.tif]

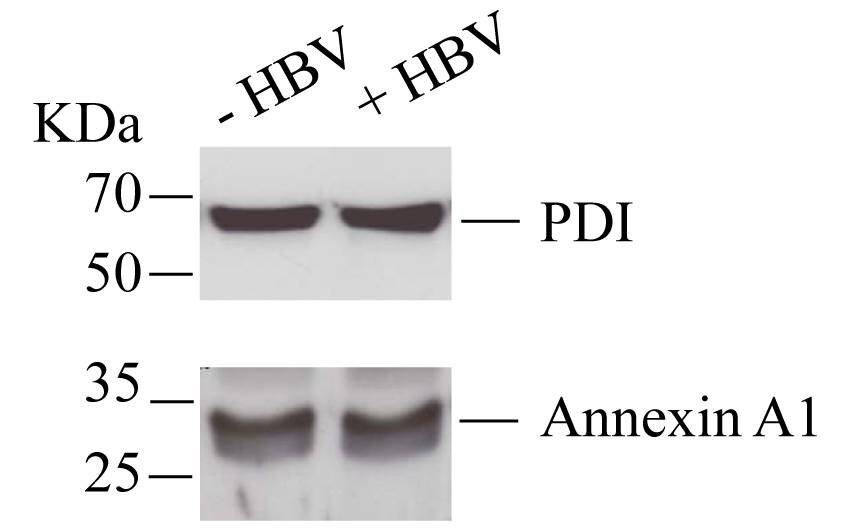

Supplement: Figure S4 — Analysis of differentially expressed proteins at PMs of (D) naïve and HBV-infected HepaRG cells. The PM fractions of (D) cells infected (+) or not (−) with HBV were analyzed by Western blotting using Annexin A1- and PDI-specific Abs. The molecular weight markers are indicated. (TIF) [file pone.0071859.s004.tif]

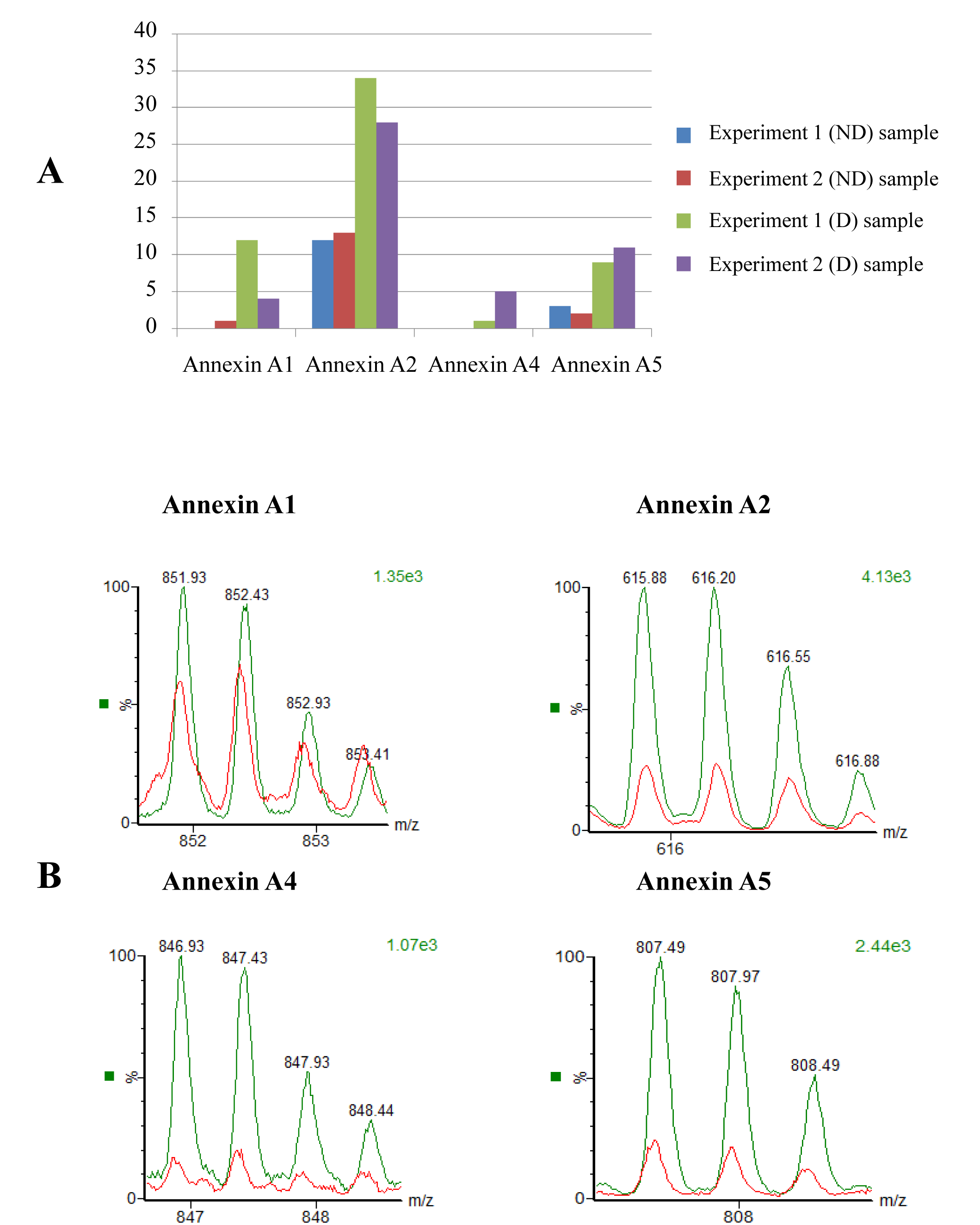

Supplement: Figure S6 — Relative quantification of Annexins. The number of spectra identified for Annexins A1, A2, A4 and A5 in (ND) and (D) HepaRG cells, in experiments 1 and 2 (A). Direct comparison of the precursor ions corresponding to peptides derived from Annexins (B). Precursor ion 851.92 (2+) and peptide GLGTDEDTLIEILASR, derived from Annexin A1; precursor ion 615.88 (3+) and peptide LSLEGDHSTPPSAYGSVK, derived from Annexin A2; precursor ion 846.95 (2+) and peptide GLGTDEDAIISVLAYR derived from Annexin A4; precursor ion 807.48 (2+) and peptide ETSGNLEQLLLAVVK.S, derived from Annexin A5. The red and green spectra correspond to proteins isolated from (ND) and (D) cells, respectively. (TIF) [file pone.0071859.s006.tif]
