# Supplementary material for: Comparative Proteomics Reveals Novel Components at the Plasma Membrane of Differentiated HepaRG Cells and Different Distribution in Hepatocyte- and Biliary-Like Cells
Source: PLoS One. 2013 Aug 20;8(8):e71859. doi: 10.1371/journal.pone.0071859 (PMC3748114; doi:10.1371/journal.pone.0071859)

MS/MS Fragmentation of **NDANPETHAFVTSPEIVTALAIAGTLK**
Found in **ACON_HUMAN** in **SwissProt**, Aconitate hydratase, mitochondrial OS=Homo sapiens GN=ACO2 PE=1 SV=2

Match to Query 595: 2778.994872 from(927.338900,3+) intensity(2264.2000) index(48)
Data file IS_111911_24.pkl

Click mouse within plot area to zoom in by factor of two about that point
Or, Plot from 300 to 1900 Da Full range
Label all possible matches ○ Label matches used for scoring ◉
Show Y-axis ☐

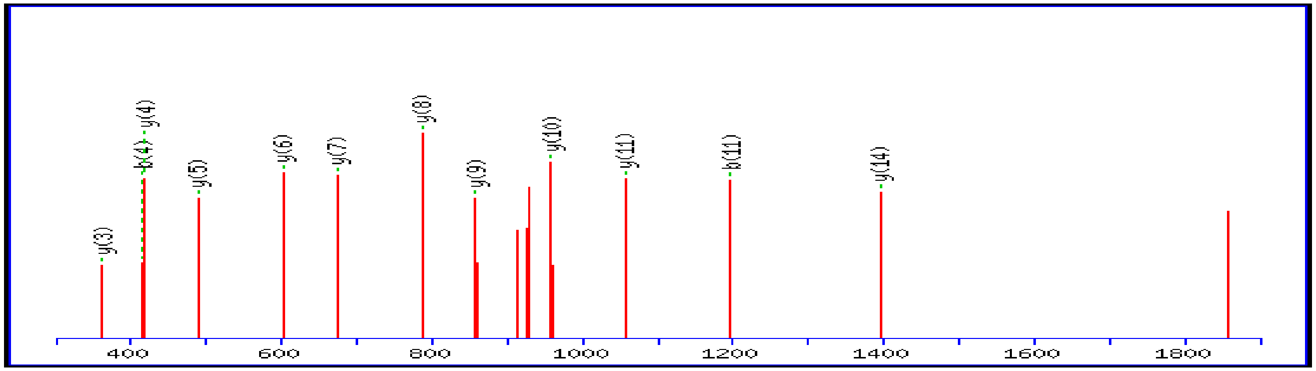


```
Monoisotopic mass of neutral peptide Mr(calc): 2779.4392
Fixed modifications: Carbamidomethyl (C) (apply to specified residues or termini only)
Ions Score: 75  Expect: 2.2e-06
Matches : 12/306 fragment ions using 17 most intense peaks   (help)
```

#	b	b^{++}	b^{*}	b^{*++}	b^{0}	b^{0++}	Seq.	y	y^{++}	y^{*}	y^{*++}	y^{0}	y^{0++}	#
1	115.0502	58.0287	98.0237	49.5155			N							27
2	230.0771	115.5422	213.0506	107.0289	212.0666	106.5369	D	2666.4036	1333.7054	2649.3770	1325.1922	2648.3930	1324.7001	26
3	301.1143	151.0608	284.0877	142.5475	283.1037	142.0555	A	2551.3766	1276.1920	2534.3501	1267.6787	2533.3661	1267.1867	25
4	**415.1572**	208.0822	398.1306	199.5690	397.1466	199.0769	N	2480.3395	1240.6734	2463.3130	1232.1601	2462.3290	1231.6681	24
5	512.2100	256.6086	495.1834	248.0953	494.1994	247.6033	P	2366.2966	1183.6519	2349.2700	1175.1387	2348.2860	1174.6467	23
6	641.2525	321.1299	624.2260	312.6166	623.2420	312.1246	E	2269.2438	1135.1256	2252.2173	1126.6123	2251.2333	1126.1203	22
7	742.3002	371.6537	725.2737	363.1405	724.2897	362.6485	T	2140.2012	1070.6043	2123.1747	1062.0910	2122.1907	1061.5990	21
8	879.3591	440.1832	862.3326	431.6699	861.3486	431.1779	H	2039.1536	1020.0804	2022.1270	1011.5671	2021.1430	1011.0751	20
9	950.3962	475.7018	933.3697	467.1885	932.3857	466.6965	A	1902.0946	951.5510	1885.0681	943.0377	1884.0841	942.5457	19
10	1097.4647	549.2360	1080.4381	540.7227	1079.4541	540.2307	F	1831.0575	916.0324	1814.0310	907.5191	1813.0470	907.0271	18
11	**1196.5331**	598.7702	1179.5065	590.2569	1178.5225	589.7649	V	1683.9891	842.4982	1666.9626	833.9849	1665.9786	833.4929	17
12	1297.5808	649.2940	1280.5542	640.7807	1279.5702	640.2887	T	1584.9207	792.9640	1567.8942	784.4507	1566.9101	783.9587	16
13	1384.6128	692.8100	1367.5862	684.2968	1366.6022	683.8047	S	1483.8730	742.4401	1466.8465	733.9269	1465.8625	733.4349	15
14	1481.6655	741.3364	1464.6390	732.8231	1463.6550	732.3311	P	***1396.8410***	698.9241	1379.8144	690.4109	1378.8304	689.9189	14
15	1610.7081	805.8577	1593.6816	797.3444	1592.6976	796.8524	E	1299.7882	650.3978	1282.7617	641.8845	1281.7777	641.3925	13
16	1723.7922	862.3997	1706.7657	853.8865	1705.7816	853.3945	I	1170.7456	585.8765	1153.7191	577.3632	1152.7351	576.8712	12
17	1822.8606	911.9339	1805.8341	903.4207	1804.8501	902.9287	V	***1057.6616***	529.3344	1040.6350	520.8212	1039.6510	520.3291	11
18	1923.9083	962.4578	1906.8817	953.9445	1905.8977	953.4525	T	***958.5932***	479.8002	941.5666	471.2869	940.5826	470.7949	10
19	1994.9454	997.9763	1977.9189	989.4631	1976.9348	988.9711	A	***857.5455***	429.2764	840.5189	420.7631	839.5349	420.2711	9
20	2108.0295	1054.5184	2091.0029	1046.0051	2090.0189	1045.5131	L	***786.5084***	393.7578	769.4818	385.2445	768.4978	384.7525	8
21	2179.0666	1090.0369	2162.0400	1081.5237	2161.0560	1081.0317	A	***673.4243***	337.2158	656.3978	328.7025	655.4137	328.2105	7
22	2292.1507	1146.5790	2275.1241	1138.0657	2274.1401	1137.5737	I	***602.3872***	301.6972	585.3606	293.1840	584.3766	292.6920	6
23	2363.1878	1182.0975	2346.1612	1173.5842	2345.1772	1173.0922	A	***489.3031***	245.1552	472.2766	236.6419	471.2926	236.1499	5
24	2420.2092	1210.6083	2403.1827	1202.0950	2402.1987	1201.6030	G	***418.2660***	209.6366	401.2395	201.1234	400.2554	200.6314	4
25	2521.2569	1261.1321	2504.2304	1252.6188	2503.2463	1252.1268	T	***361.2445***	181.1259	344.2180	172.6126	343.2340	172.1206	3
26	2634.3410	1317.6741	2617.3144	1309.1609	2616.3304	1308.6688	L	260.1969	130.6021	243.1703	122.0888			2
27							K	147.1128	74.0600	130.0863	65.5468			1

MS/MS Fragmentation of **VGWEQLLTTIAR**
Found in **ACTN1_HUMAN** in **SwissProt**, Alpha-actinin-1 OS=Homo sapiens GN=ACTN1 PE=1 SV=2

Match to Query 498: 1386.002848 from(694.008700,2+) intensity(3436.3000) index(45)
Data file IS_111911_24.pkl

Click mouse within plot area to zoom in by factor of two about that point
Or, [Plot from] 100 to 1400 Da [Full range]
Label all possible matches ○ Label matches used for scoring ◉
Show Y-axis ☐

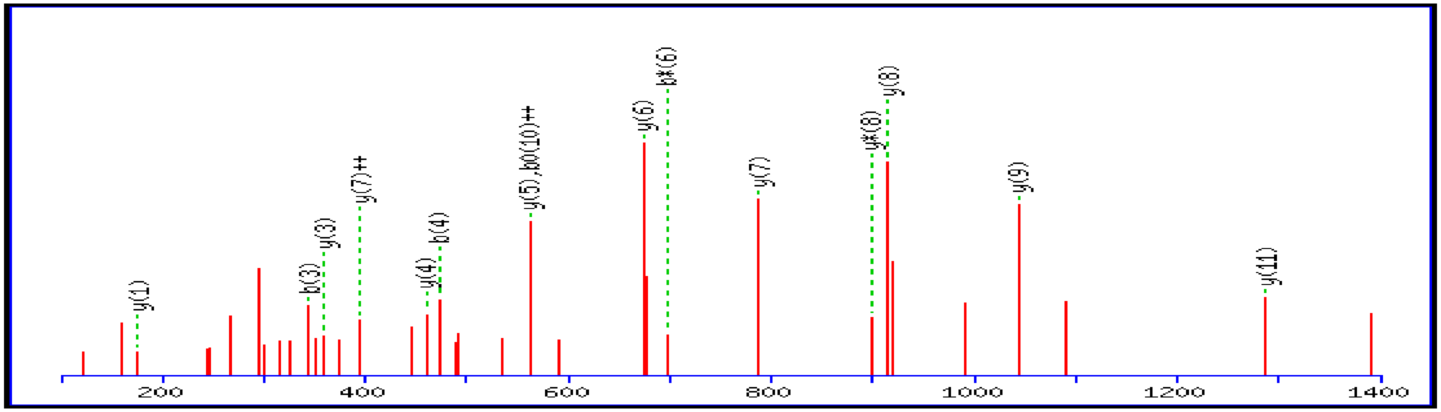

Monoisotopic mass of neutral peptide Mr(calc): 1385.7667
Fixed modifications: Carbamidomethyl (C) (apply to specified residues or termini only)
Ions Score: 62 Expect: 9.9e-05
Matches : 15/112 fragment ions using 26 most intense peaks (help)

#	b	b^{++}	b^{*}	b^{*++}	b^{0}	b^{0++}	Seq.	y	y^{++}	y^{*}	y^{*++}	y^{0}	y^{0++}	#
1	100.0757	50.5415					V							12
2	157.0972	79.0522					G	1287.7056	644.3564	1270.6790	635.8431	1269.6950	635.3511	11
3	343.1765	172.0919					W	1230.6841	615.8457	1213.6576	607.3324	1212.6735	606.8404	10
4	472.2191	236.6132			454.2085	227.6079	E	1044.6048	522.8060	1027.5782	514.2928	1026.5942	513.8007	9
5	600.2776	300.6425	583.2511	292.1292	582.2671	291.6372	Q	915.5622	458.2847	898.5356	449.7715	897.5516	449.2795	8
6	713.3617	357.1845	696.3352	348.6712	695.3511	348.1792	L	787.5036	394.2554	770.4771	385.7422	769.4931	385.2502	7
7	826.4458	413.7265	809.4192	405.2132	808.4352	404.7212	L	674.4196	337.7134	657.3930	329.2001	656.4090	328.7081	6
8	927.4934	464.2504	910.4669	455.7371	909.4829	455.2451	T	561.3355	281.1714	544.3089	272.6581	543.3249	272.1661	5
9	1028.5411	514.7742	1011.5146	506.2609	1010.5306	505.7689	T	460.2878	230.6475	443.2613	222.1343	442.2772	221.6423	4
10	1141.6252	571.3162	1124.5986	562.8030	1123.6146	562.3109	I	359.2401	180.1237	342.2136	171.6104			3
11	1212.6623	606.8348	1195.6358	598.3215	1194.6517	597.8295	A	246.1561	123.5817	229.1295	115.0684			2
12							R	175.1190	88.0631	158.0924	79.5498			1

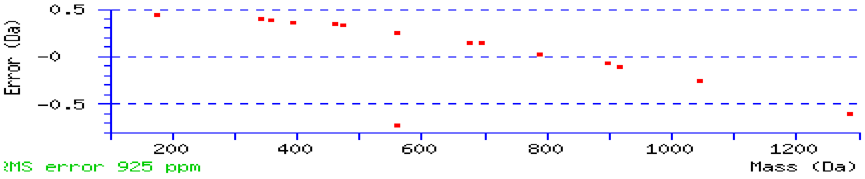

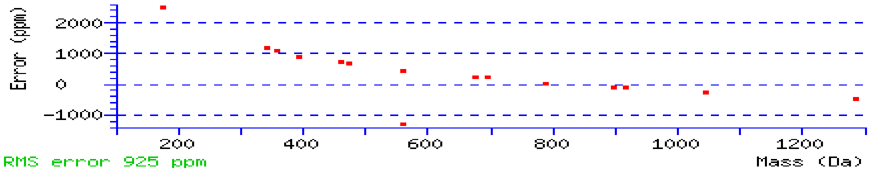

MS/MS Fragmentation of **VNPTVFFDIAVDGEPLGR**
Found in **PPIA_HUMAN** in **SwissProt**, Peptidyl-prolyl cis-trans isomerase A OS=Homo sapiens GN=PPIA PE=1 SV=2

Match to Query 552: 1944.601248 from(973.307900,2+) intensity(2405.4000) index(326)
Data file IS_111911_24.pkl

Click mouse within plot area to zoom in by factor of two about that point
Or, [Plot from] 100 to 1500 Da [Full range]
Label all possible matches ○ Label matches used for scoring ◉
Show Y-axis ☐

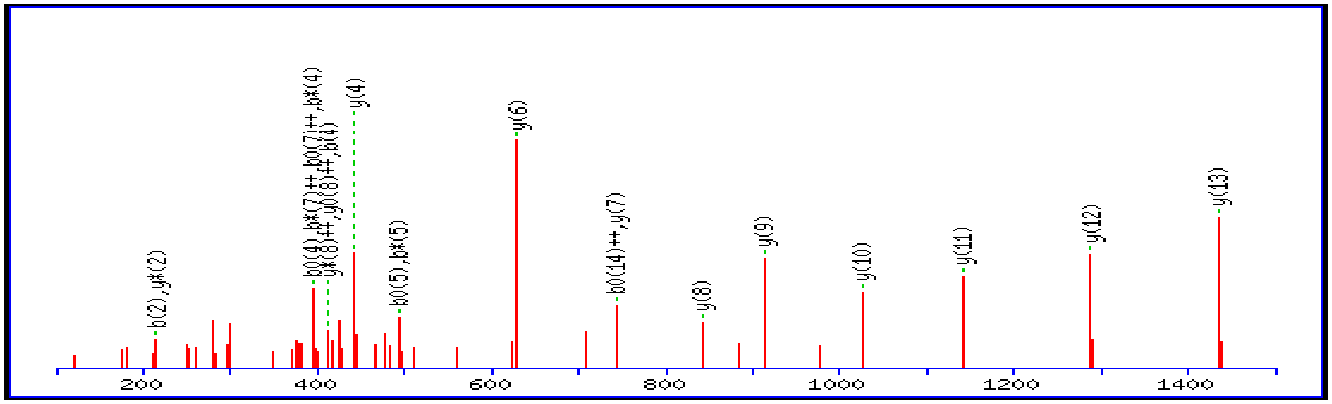


```
Monoisotopic mass of neutral peptide Mr(calc): 1944.9946
Fixed modifications: Carbamidomethyl (C) (apply to specified residues or termini only)
Ions Score: 61  Expect: 8e-05
Matches : 21/188 fragment ions using 21 most intense peaks  (help)
```

#	b	b^{++}	b^*	b^{*++}	b^0	b^{0++}	Seq.	y	y^{++}	y^*	y^{*++}	y^0	y^{0++}	#
1	100.0757	50.5415					V							18
2	**214.1186**	107.5629	197.0921	99.0497			N	1846.9334	923.9703	1829.9068	915.4571	1828.9228	914.9651	17
3	311.1714	156.0893	294.1448	147.5761			P	1732.8905	866.9489	1715.8639	858.4356	1714.8799	857.9436	16
4	**412.2191**	206.6132	395.1925	198.0999	394.2085	197.6079	T	1635.8377	818.4225	1618.8112	809.9092	1617.8271	809.4172	15
5	511.2875	256.1474	494.2609	247.6341	493.2769	247.1421	V	1534.7900	767.8986	1517.7635	759.3854	1516.7795	758.8934	14
6	658.3559	329.6816	641.3293	321.1683	640.3453	320.6763	F	***1435.7216***	718.3644	1418.6951	709.8512	1417.7110	709.3592	13
7	805.4243	403.2158	788.3978	394.7025	787.4137	394.2105	F	***1288.6532***	644.8302	1271.6266	636.3170	1270.6426	635.8250	12
8	920.4512	460.7293	903.4247	452.2160	902.4407	451.7240	D	***1141.5848***	571.2960	1124.5582	562.7828	1123.5742	562.2907	11
9	1033.5353	517.2713	1016.5088	508.7580	1015.5247	508.2660	I	***1026.5578***	513.7826	1009.5313	505.2693	1008.5473	504.7773	10
10	1104.5724	552.7898	1087.5459	544.2766	1086.5619	543.7846	A	***913.4738***	457.2405	896.4472	448.7272	895.4632	448.2352	9
11	1203.6408	602.3241	1186.6143	593.8108	1185.6303	593.3188	V	***842.4367***	421.7220	825.4101	413.2087	824.4261	412.7167	8
12	1318.6678	659.8375	1301.6412	651.3243	1300.6572	650.8322	D	***743.3682***	372.1878	726.3417	363.6745	725.3577	363.1825	7
13	1375.6892	688.3483	1358.6627	679.8350	1357.6787	679.3430	G	***628.3413***	314.6743	611.3148	306.1610	610.3307	305.6690	6
14	1504.7318	752.8696	1487.7053	744.3563	1486.7213	743.8643	E	571.3198	286.1636	554.2933	277.6503	553.3093	277.1583	5
15	1601.7846	801.3959	1584.7581	792.8827	1583.7740	792.3907	P	***442.2772***	221.6423	425.2507	213.1290			4
16	1714.8687	857.9380	1697.8421	849.4247	1696.8581	848.9327	L	345.2245	173.1159	328.1979	164.6026			3
17	1771.8901	886.4487	1754.8636	877.9354	1753.8796	877.4434	G	232.1404	116.5738	215.1139	108.0606			2
18							R	175.1190	88.0631	158.0924	79.5498			1

MS/MS Fragmentation of **YSQFINFPIYVWSSK**
Found in **ENPL_HUMAN** in **SwissProt**, Endoplasmin OS=Homo sapiens GN=HSP90B1 PE=1 SV=1

Match to Query 547: 1877.632648 from(939.823600,2+) intensity(5825.1000) index(479)
Data file IS_111911_24.pkl

Click mouse within plot area to zoom in by factor of two about that point
Or, Plot from 100 to 1400 Da Full range
Label all possible matches ○ Label matches used for scoring ◉
Show Y-axis ☐

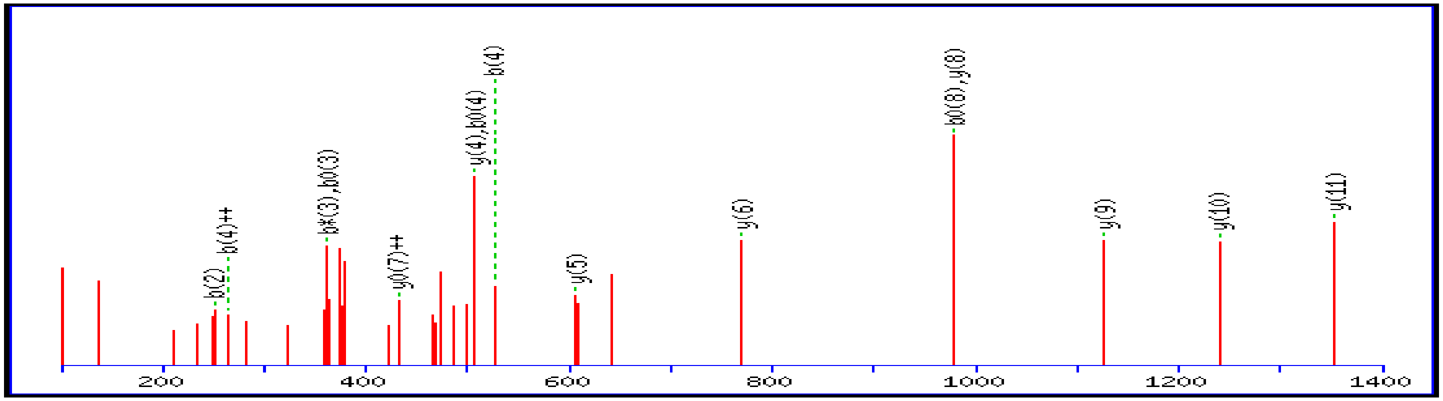

Monoisotopic mass of neutral peptide Mr(calc): 1877.9352
Fixed modifications: Carbamidomethyl (C) (apply to specified residues or termini only)
Ions Score: 45 Expect: 0.0037
Matches : 15/160 fragment ions using 17 most intense peaks (help)

#	b	b^{++}	b^{*}	b^{*++}	b^{0}	b^{0++}	Seq.	y	y^{++}	y^{*}	y^{*++}	y^{0}	y^{0++}	#
1	164.0706	82.5389					Y							15
2	**251.1026**	126.0550			233.0921	117.0497	S	1715.8792	858.4432	1698.8526	849.9299	1697.8686	849.4379	14
3	379.1612	190.0842	362.1347	181.5710	361.1506	181.0790	Q	1628.8471	814.9272	1611.8206	806.4139	1610.8366	805.9219	13
4	**526.2296**	263.6185	509.2031	255.1052	508.2191	254.6132	F	1500.7886	750.8979	1483.7620	742.3846	1482.7780	741.8926	12
5	639.3137	320.1605	622.2871	311.6472	621.3031	311.1552	I	*1353.7201*	677.3637	1336.6936	668.8504	1335.7096	668.3584	11
6	753.3566	377.1819	736.3301	368.6687	735.3461	368.1767	N	*1240.6361*	620.8217	1223.6095	612.3084	1222.6255	611.8164	10
7	900.4250	450.7162	883.3985	442.2029	882.4145	441.7109	F	*1126.5932*	563.8002	1109.5666	555.2869	1108.5826	554.7949	9
8	997.4778	499.2425	980.4512	490.7293	979.4672	490.2373	P	*979.5247*	490.2660	962.4982	481.7527	961.5142	481.2607	8
9	1110.5619	555.7846	1093.5353	547.2713	1092.5513	546.7793	I	882.4720	441.7396	865.4454	433.2264	864.4614	432.7343	7
10	1273.6252	637.3162	1256.5986	628.8030	1255.6146	628.3109	Y	*769.3879*	385.1976	752.3614	376.6843	751.3774	376.1923	6
11	1372.6936	686.8504	1355.6671	678.3372	1354.6830	677.8452	V	*606.3246*	303.6659	589.2980	295.1527	588.3140	294.6606	5
12	1558.7729	779.8901	1541.7464	771.3768	1540.7623	770.8848	W	*507.2562*	254.1317	490.2296	245.6185	489.2456	245.1264	4
13	1645.8049	823.4061	1628.7784	814.8928	1627.7944	814.4008	S	321.1769	161.0921	304.1503	152.5788	303.1663	152.0868	3
14	1732.8370	866.9221	1715.8104	858.4088	1714.8264	857.9168	S	234.1448	117.5761	217.1183	109.0628	216.1343	108.5708	2
15							K	147.1128	74.0600	130.0863	65.5468			1

MS/MS Fragmentation of **LGLLGLANSLAIEGR**
Found in **DHB4_HUMAN** in **SwissProt**, Peroxisomal multifunctional enzyme type 2 OS=Homo sapiens GN=HSD17B4 PE=1 SV=3

Match to Query 396: 1495.983848 from(748.999200,2+) intensity(19858.2000) index(111)
Data file IS_111911_25a.txt

Click mouse within plot area to zoom in by factor of two about that point
Or, Plot from 200 to 1500 Da Full range
Label all possible matches ○ Label matches used for scoring ◉
Show Y-axis ☐

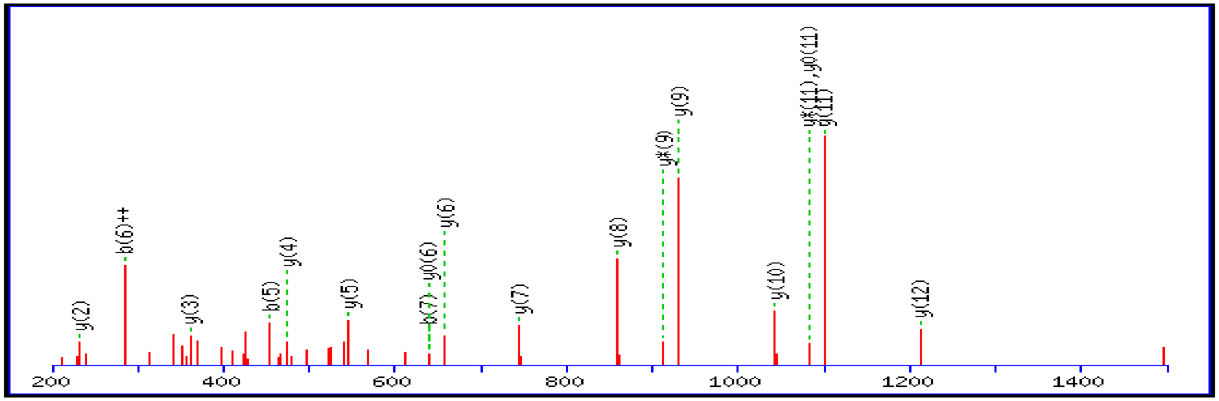

Monoisotopic mass of neutral peptide Mr(calc): 1495.8722
Fixed modifications: Carbamidomethyl (C) (apply to specified residues or termini only)
Ions Score: 90 Expect: 1.8e-07
Matches : 19/134 fragment ions using 26 most intense peaks (help)

#	b	b^{++}	b^*	b^{*++}	b^0	b^{0++}	Seq.	y	y^{++}	y^*	y^{*++}	y^0	y^{0++}	#
1	114.0913	57.5493					L							15
2	171.1128	86.0600					G	1383.7954	692.4014	1366.7689	683.8881	1365.7849	683.3961	14
3	**284.1969**	142.6021					L	1326.7740	663.8906	1309.7474	655.3774	1308.7634	654.8853	13
4	397.2809	199.1441					L	***1213.6899***	607.3486	1196.6634	598.8353	1195.6793	598.3433	12
5	**454.3024**	227.6548					G	***1100.6058***	550.8066	1083.5793	542.2933	1082.5953	541.8013	11
6	567.3865	284.1969					L	***1043.5844***	522.2958	1026.5578	513.7826	1025.5738	513.2905	10
7	**638.4236**	319.7154					A	***930.5003***	465.7538	913.4738	457.2405	912.4898	456.7485	9
8	752.4665	376.7369	735.4400	368.2236			N	***859.4632***	430.2352	842.4367	421.7220	841.4526	421.2300	8
9	839.4985	420.2529	822.4720	411.7396	821.4880	411.2476	S	***745.4203***	373.2138	728.3937	364.7005	727.4097	364.2085	7
10	952.5826	476.7949	935.5560	468.2817	934.5720	467.7897	L	***658.3883***	329.6978	641.3617	321.1845	640.3777	320.6925	6
11	1023.6197	512.3135	1006.5932	503.8002	1005.6091	503.3082	A	***545.3042***	273.1557	528.2776	264.6425	527.2936	264.1504	5
12	1136.7038	568.8555	1119.6772	560.3422	1118.6932	559.8502	I	***474.2671***	237.6372	457.2405	229.1239	456.2565	228.6319	4
13	1265.7464	633.3768	1248.7198	624.8635	1247.7358	624.3715	E	***361.1830***	181.0951	344.1565	172.5819	343.1724	172.0899	3
14	1322.7678	661.8876	1305.7413	653.3743	1304.7573	652.8823	G	***232.1404***	116.5738	215.1139	108.0606			2
15							R	175.1190	88.0631	158.0924	79.5498			1

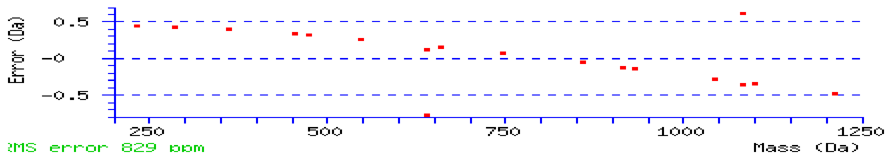

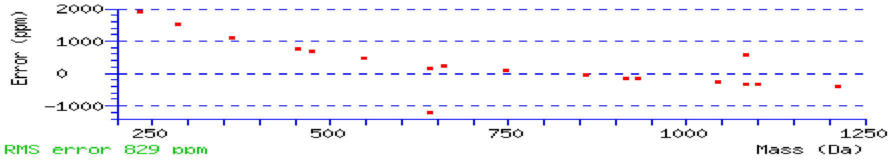

MS/MS Fragmentation of **GLGTDEESILTLLTSR**
Found in **ANXA5_HUMAN** in **SwissProt**, Annexin A5 OS=Homo sapiens GN=ANXA5 PE=1 SV=2

Match to Query 443: 1703.763448 from(852.889000,2+) intensity(20596.0000) index(129)
Data file IS_111911_25a.txt

Click mouse within plot area to zoom in by factor of two about that point
Or, Plot from 100 to 1600 Da Full range
Label all possible matches ○ Label matches used for scoring ◉
Show Y-axis ☐

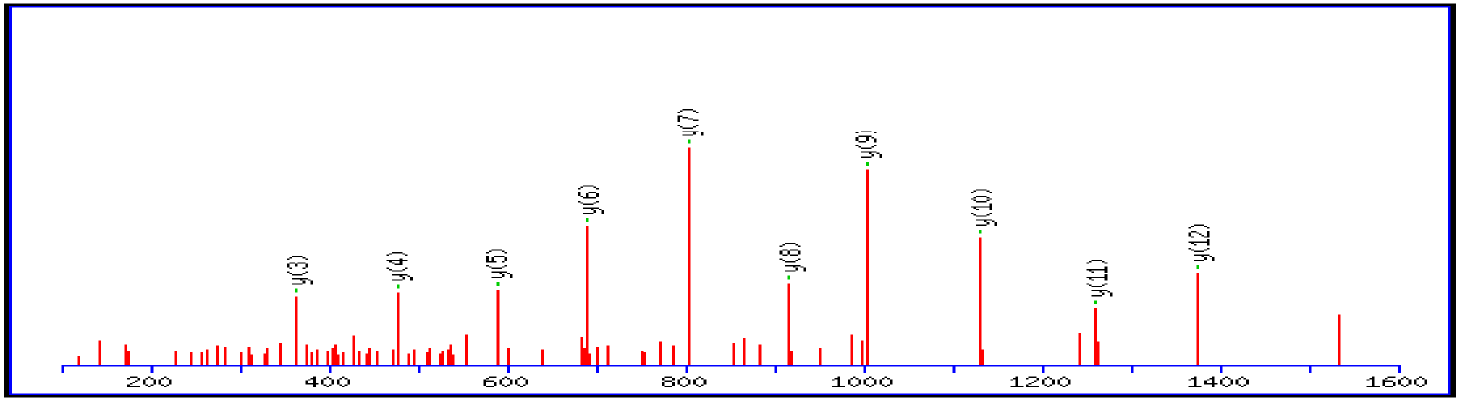

Monoisotopic mass of neutral peptide Mr(calc): 1703.8941
Fixed modifications: Carbamidomethyl (C) (apply to specified residues or termini only)
Ions Score: 89 Expect: 1.8e-07
Matches : 10/142 fragment ions using 13 most intense peaks (help)

#	b	b^{++}	b^0	b^{0++}	Seq.	y	y^{++}	y^*	y^{*++}	y^0	y^{0++}	#
1	58.0287	29.5180			G							16
2	171.1128	86.0600			L	1647.8800	824.4436	1630.8534	815.9303	1629.8694	815.4383	15
3	228.1343	114.5708			G	1534.7959	767.9016	1517.7693	759.3883	1516.7853	758.8963	14
4	329.1819	165.0946	311.1714	156.0893	T	1477.7744	739.3909	1460.7479	730.8776	1459.7639	730.3856	13
5	444.2089	222.6081	426.1983	213.6028	D	*1376.7268*	688.8670	1359.7002	680.3537	1358.7162	679.8617	12
6	573.2515	287.1294	555.2409	278.1241	E	*1261.6998*	631.3535	1244.6733	622.8403	1243.6892	622.3483	11
7	702.2941	351.6507	684.2835	342.6454	E	*1132.6572*	566.8322	1115.6307	558.3190	1114.6466	557.8270	10
8	789.3261	395.1667	771.3155	386.1614	S	*1003.6146*	502.3109	986.5881	493.7977	985.6041	493.3057	9
9	902.4102	451.7087	884.3996	442.7034	I	*916.5826*	458.7949	899.5560	450.2817	898.5720	449.7897	8
10	1015.4942	508.2508	997.4837	499.2455	L	*803.4985*	402.2529	786.4720	393.7396	785.4880	393.2476	7
11	1116.5419	558.7746	1098.5313	549.7693	T	*690.4145*	345.7109	673.3879	337.1976	672.4039	336.7056	6
12	1229.6260	615.3166	1211.6154	606.3113	L	*589.3668*	295.1870	572.3402	286.6738	571.3562	286.1817	5
13	1342.7100	671.8587	1324.6995	662.8534	L	*476.2827*	238.6450	459.2562	230.1317	458.2722	229.6397	4
14	1443.7577	722.3825	1425.7472	713.3772	T	*363.1987*	182.1030	346.1721	173.5897	345.1881	173.0977	3
15	1530.7897	765.8985	1512.7792	756.8932	S	262.1510	131.5791	245.1244	123.0659	244.1404	122.5738	2
16					R	175.1190	88.0631	158.0924	79.5498			1

MS/MS Fragmentation of **AFYVNVLNEEQR**
Found in **CATA_HUMAN** in **SwissProt**, Catalase OS=Homo sapiens GN=CAT PE=1 SV=3

Match to Query 394: 1480.867248 from(741.440900,2+) intensity(4739.8000) index(54)
Data file IS_111911_25a.txt

Click mouse within plot area to zoom in by factor of two about that point
Or, Plot from 100 to 1500 Da Full range
Label all possible matches ○ Label matches used for scoring ◉
Show Y-axis ☐

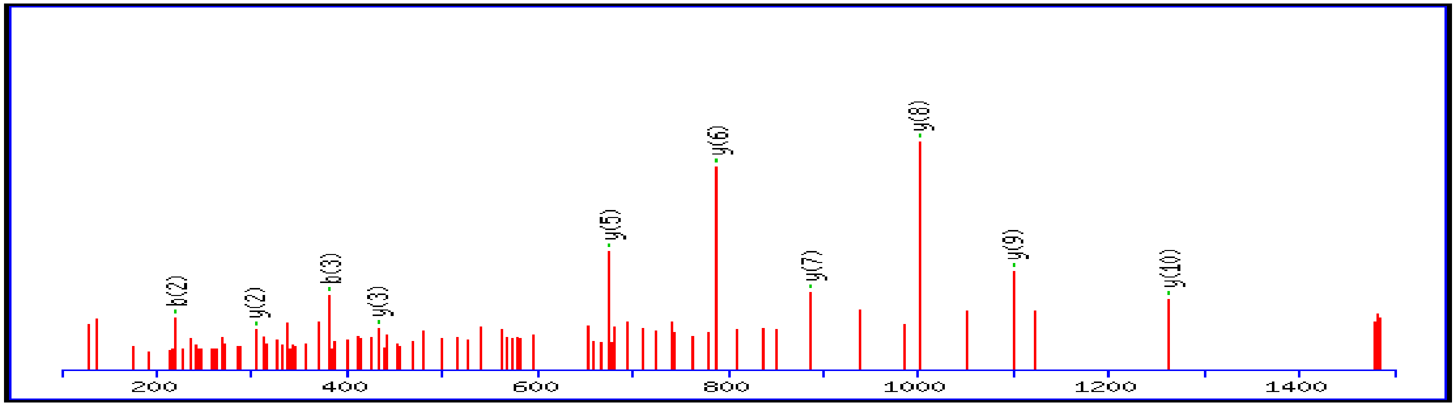

Monoisotopic mass of neutral peptide Mr(calc): 1480.7310
Fixed modifications: Carbamidomethyl (C) (apply to specified residues or termini only)
Ions Score: 70 Expect: 1.8e-05
Matches : 10/104 fragment ions using 12 most intense peaks (help)

#	b	b^{++}	b^{*}	b^{*++}	b^{0}	b^{0++}	Seq.	y	y^{++}	y^{*}	y^{*++}	y^{0}	y^{0++}	#
1	72.0444	36.5258					A							12
2	**219.1128**	110.0600					F	1410.7012	705.8542	1393.6747	697.3410	1392.6906	696.8490	11
3	**382.1761**	191.5917					Y	***1263.6328***	632.3200	1246.6062	623.8068	1245.6222	623.3148	10
4	481.2445	241.1259					V	***1100.5695***	550.7884	1083.5429	542.2751	1082.5589	541.7831	9
5	595.2875	298.1474	578.2609	289.6341			N	***1001.5010***	501.2542	984.4745	492.7409	983.4905	492.2489	8
6	694.3559	347.6816	677.3293	339.1683			V	***887.4581***	444.2327	870.4316	435.7194	869.4476	435.2274	7
7	807.4400	404.2236	790.4134	395.7103			L	***788.3897***	394.6985	771.3632	386.1852	770.3791	385.6932	6
8	921.4829	461.2451	904.4563	452.7318			N	***675.3056***	338.1565	658.2791	329.6432	657.2951	329.1512	5
9	1050.5255	525.7664	1033.4989	517.2531	1032.5149	516.7611	E	561.2627	281.1350	544.2362	272.6217	543.2522	272.1297	4
10	1179.5681	590.2877	1162.5415	581.7744	1161.5575	581.2824	E	***432.2201***	216.6137	415.1936	208.1004	414.2096	207.6084	3
11	1307.6266	654.3170	1290.6001	645.8037	1289.6161	645.3117	Q	***303.1775***	152.0924	286.1510	143.5791			2
12							R	175.1190	88.0631	158.0924	79.5498			1

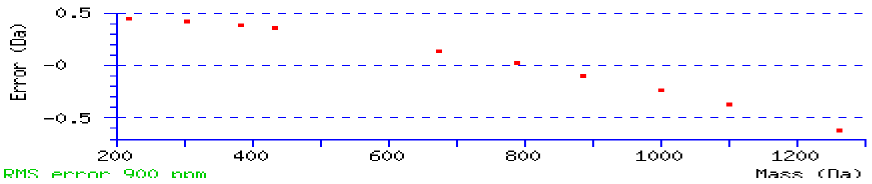

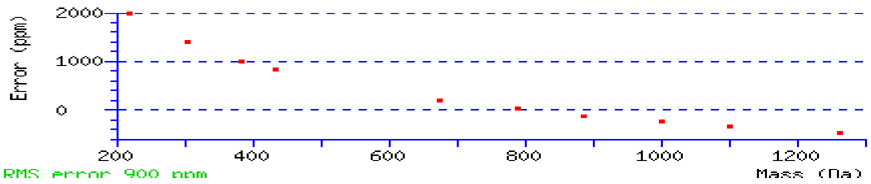

MS/MS Fragmentation of **IVPNVLLEQGK**
Found in **CISY_HUMAN** in **SwissProt**, Citrate synthase, mitochondrial OS=Homo sapiens GN=CS PE=1 SV=2

Match to Query 279: 1209.115248 from(605.564900,2+) intensity(4882.3000) index(430)
Data file IS_111911_25a.txt

Click mouse within plot area to zoom in by factor of two about that point
Or. Plot from 100 to 1300 Da Full range
Label all possible matches ○ Label matches used for scoring ◉
Show Y-axis ☐

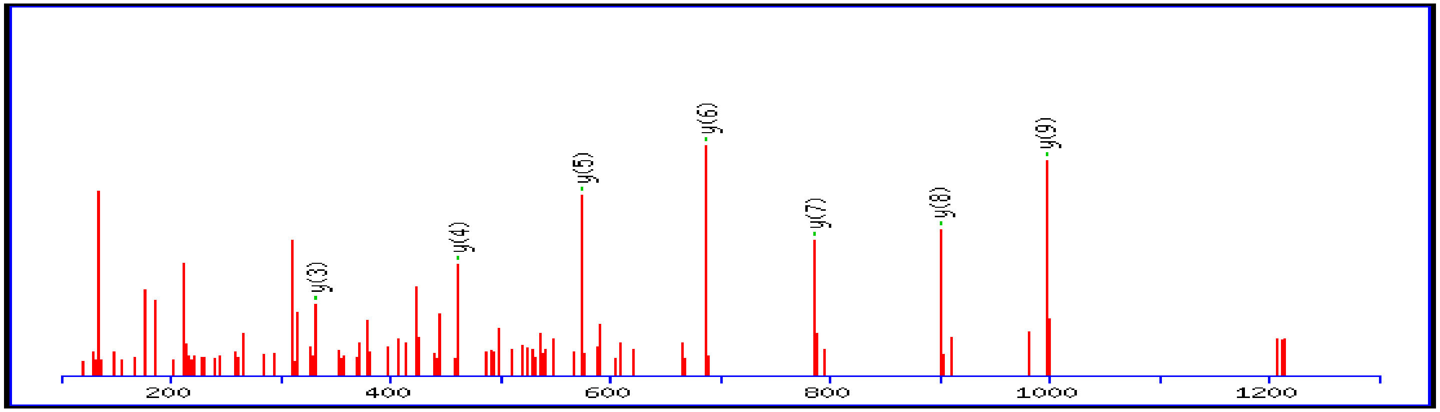

Monoisotopic mass of neutral peptide Mr(calc): 1208.7129
Fixed modifications: Carbamidomethyl (C) (apply to specified residues or termini only)
Ions Score: 58 Expect: 0.00026
Matches : 7/94 fragment ions using 10 most intense peaks (help)

#	b	b^{++}	b^{*}	b^{*++}	b^{0}	b^{0++}	Seq.	y	y^{++}	y^{*}	y^{*++}	y^{0}	y^{0++}	#
1	114.0913	57.5493					I							11
2	213.1598	107.0835					V	1096.6361	548.8217	1079.6095	540.3084	1078.6255	539.8164	10
3	310.2125	155.6099					P	*997.5677*	499.2875	980.5411	490.7742	979.5571	490.2822	9
4	424.2554	212.6314	407.2289	204.1181			N	*900.5149*	450.7611	883.4884	442.2478	882.5043	441.7558	8
5	523.3239	262.1656	506.2973	253.6523			V	*786.4720*	393.7396	769.4454	385.2264	768.4614	384.7343	7
6	636.4079	318.7076	619.3814	310.1943			L	*687.4036*	344.2054	670.3770	335.6921	669.3930	335.2001	6
7	749.4920	375.2496	732.4654	366.7364			L	*574.3195*	287.6634	557.2930	279.1501	556.3089	278.6581	5
8	878.5346	439.7709	861.5080	431.2577	860.5240	430.7656	E	*461.2354*	231.1214	444.2089	222.6081	443.2249	222.1161	4
9	1006.5932	503.8002	989.5666	495.2869	988.5826	494.7949	Q	*332.1928*	166.6001	315.1663	158.0868			3
10	1063.6146	532.3109	1046.5881	523.7977	1045.6041	523.3057	G	204.1343	102.5708	187.1077	94.0575			2
11							K	147.1128	74.0600	130.0863	65.5468			1

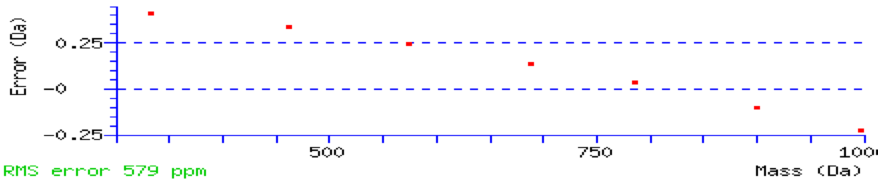

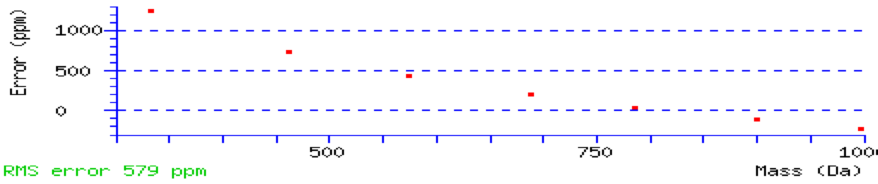

MS/MS Fragmentation of **VAFITGGGTGLGK**
Found in **DECR_HUMAN** in **SwissProt**, 2,4-dienoyl-CoA reductase, mitochondrial OS=Homo sapiens GN=DECR1 PE=1 SV=1

Match to Query 267: 1177.091848 from(589.553200,2+) intensity(4419.7000) index(33)
Data file IS_111911_25a.txt

Click mouse within plot area to zoom in by factor of two about that point
Or, Plot from 100 to 1100 Da Full range
Label all possible matches ◯ Label matches used for scoring ◉
Show Y-axis ☐

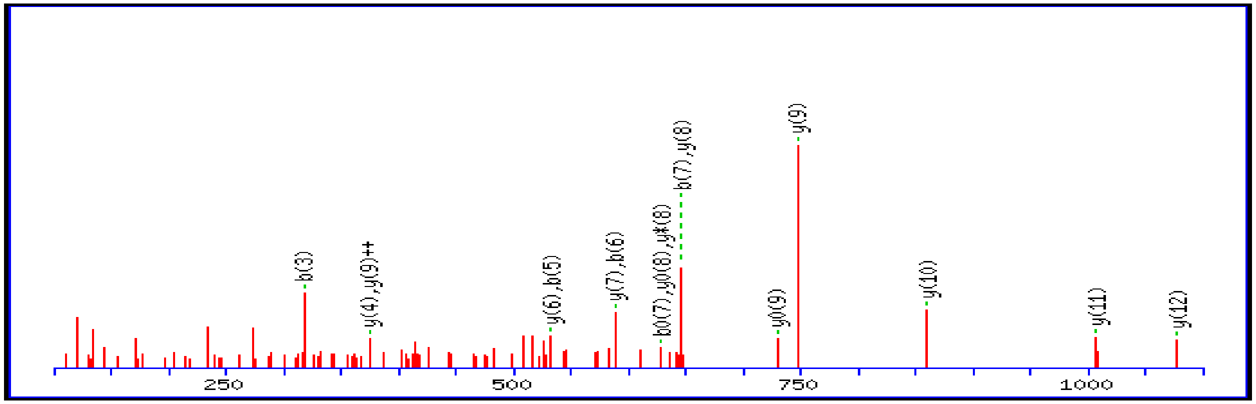

Monoisotopic mass of neutral peptide Mr(calc): 1176.6503
Fixed modifications: Carbamidomethyl (C) (apply to specified residues or termini only)
Ions Score: 58 Expect: 0.00029
Matches : 17/104 fragment ions using 18 most intense peaks (help)

#	b	b^{++}	b^0	b^{0++}	Seq.	y	y^{++}	y*	y^{*++}	y^0	y^{0++}	#
1	100.0757	50.5415			V							13
2	171.1128	86.0600			A	1078.5891	539.7982	1061.5626	531.2849	1060.5786	530.7929	12
3	318.1812	159.5942			F	1007.5520	504.2797	990.5255	495.7664	989.5415	495.2744	11
4	431.2653	216.1363			I	860.4836	430.7454	843.4571	422.2322	842.4730	421.7402	10
5	532.3130	266.6601	514.3024	257.6548	T	747.3995	374.2034	730.3730	365.6901	729.3890	365.1981	9
6	589.3344	295.1709	571.3239	286.1656	G	646.3519	323.6796	629.3253	315.1663	628.3413	314.6743	8
7	646.3559	323.6816	628.3453	314.6763	G	589.3304	295.1688	572.3039	286.6556	571.3198	286.1636	7
8	703.3774	352.1923	685.3668	343.1870	G	532.3089	266.6581	515.2824	258.1448	514.2984	257.6528	6
9	804.4250	402.7162	786.4145	393.7109	T	475.2875	238.1474	458.2609	229.6341	457.2769	229.1421	5
10	861.4465	431.2269	843.4359	422.2216	G	374.2398	187.6235	357.2132	179.1103			4
11	974.5306	487.7689	956.5200	478.7636	L	317.2183	159.1128	300.1918	150.5995			3
12	1031.5520	516.2797	1013.5415	507.2744	G	204.1343	102.5708	187.1077	94.0575			2
13					K	147.1128	74.0600	130.0863	65.5468			1

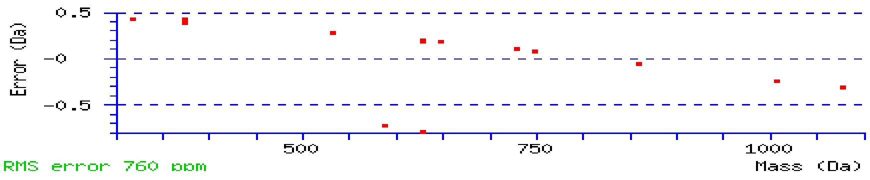

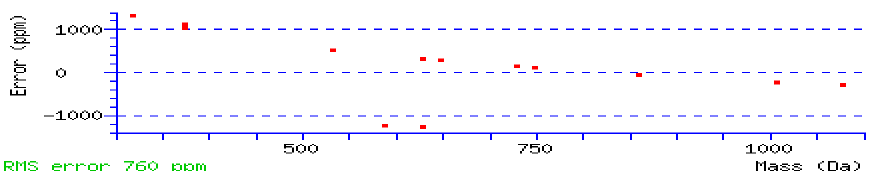

MS/MS Fragmentation of DATNVGDEGGFAPNILENNEALELLK
Found in ENOB_HUMAN in SwissProt, Beta-enolase OS=Homo sapiens GN=ENO3 PE=1 SV=5

Match to Query 561: 2741.957772 from(914.993200,3+) intensity(26589.9000) index(489)
Data file IS_111911_25a.txt

Click mouse within plot area to zoom in by factor of two about that point
Or, Plot from 200 to 2800 Da Full range
Label all possible matches ○ Label matches used for scoring ◉
Show Y-axis ☐

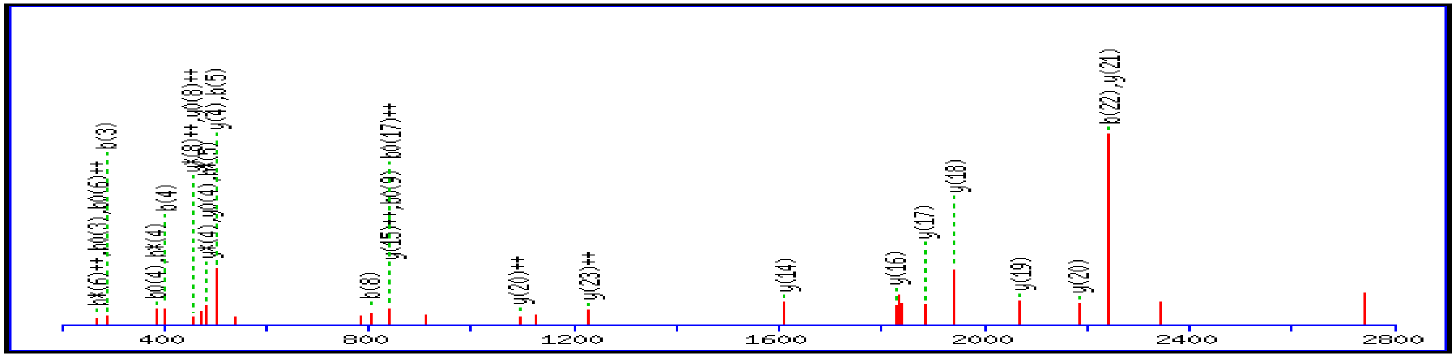

Monoisotopic mass of neutral peptide Mr(calc): 2742.3348
Fixed modifications: Carbamidomethyl (C) (apply to specified residues or termini only)
Ions Score: 51 Expect: 0.00055
Matches : 28/288 fragment ions using 26 most intense peaks (help)

#	b	b++	b*	b*++	b0	b0++	Seq.	y	y++	y*	y*++	y0	y0++	#
1	116.0342	58.5207			98.0237	49.5155	D							26
2	187.0713	94.0393			169.0608	85.0340	A	2628.3152	1314.6612	2611.2886	1306.1479	2610.3046	1305.6559	25
3	288.1190	144.5631			270.1084	135.5579	T	2557.2780	1279.1427	2540.2515	1270.6294	2539.2675	1270.1374	24
4	402.1619	201.5846	385.1354	193.0713	384.1514	192.5793	N	2456.2304	1228.6188	2439.2038	1220.1055	2438.2198	1219.6135	23
5	501.2304	251.1188	484.2038	242.6055	483.2198	242.1135	V	2342.1874	1171.5974	2325.1609	1163.0841	2324.1769	1162.5921	22
6	558.2518	279.6295	541.2253	271.1163	540.2413	270.6243	G	2243.1190	1122.0631	2226.0925	1113.5499	2225.1085	1113.0579	21
7	673.2788	337.1430	656.2522	328.6297	655.2682	328.1377	D	2186.0976	1093.5524	2169.0710	1085.0391	2168.0870	1084.5471	20
8	802.3214	401.6643	785.2948	393.1510	784.3108	392.6590	E	2071.0706	1036.0389	2054.0441	1027.5257	2053.0600	1027.0337	19
9	859.3428	430.1750	842.3163	421.6618	841.3323	421.1698	G	1942.0280	971.5176	1925.0015	963.0044	1924.0175	962.5124	18
10	916.3643	458.6858	899.3377	450.1725	898.3537	449.6805	G	1885.0066	943.0069	1867.9800	934.4936	1866.9960	934.0016	17
11	1063.4327	532.2200	1046.4061	523.7067	1045.4221	523.2147	F	1827.9851	914.4962	1810.9585	905.9829	1809.9745	905.4909	16
12	1134.4698	567.7385	1117.4433	559.2253	1116.4592	558.7333	A	1680.9167	840.9620	1663.8901	832.4487	1662.9061	831.9567	15
13	1231.5226	616.2649	1214.4960	607.7517	1213.5120	607.2596	P	1609.8796	805.4434	1592.8530	796.9301	1591.8690	796.4381	14
14	1345.5655	673.2864	1328.5390	664.7731	1327.5549	664.2811	N	1512.8268	756.9170	1495.8002	748.4038	1494.8162	747.9118	13
15	1458.6496	729.8284	1441.6230	721.3151	1440.6390	720.8231	I	1398.7839	699.8956	1381.7573	691.3823	1380.7733	690.8903	12
16	1571.7336	786.3705	1554.7071	777.8572	1553.7231	777.3652	L	1285.6998	643.3535	1268.6733	634.8403	1267.6892	634.3483	11
17	1700.7762	850.8917	1683.7497	842.3785	1682.7657	841.8865	E	1172.6157	586.8115	1155.5892	578.2982	1154.6052	577.8062	10
18	1814.8191	907.9132	1797.7926	899.3999	1796.8086	898.9079	N	1043.5732	522.2902	1026.5466	513.7769	1025.5626	513.2849	9
19	1928.8621	964.9347	1911.8355	956.4214	1910.8515	955.9294	N	929.5302	465.2687	912.5037	456.7555	911.5197	456.2635	8
20	2057.9047	1029.4560	2040.8781	1020.9427	2039.8941	1020.4507	E	815.4873	408.2473	798.4607	399.7340	797.4767	399.2420	7
21	2128.9418	1064.9745	2111.9152	1056.4613	2110.9312	1055.9692	A	686.4447	343.7260	669.4182	335.2127	668.4341	334.7207	6
22	2242.0258	1121.5166	2224.9993	1113.0033	2224.0153	1112.5113	L	615.4076	308.2074	598.3810	299.6942	597.3970	299.2022	5
23	2371.0684	1186.0379	2354.0419	1177.5246	2353.0579	1177.0326	E	502.3235	251.6654	485.2970	243.1521	484.3130	242.6601	4
24	2484.1525	1242.5799	2467.1260	1234.0666	2466.1419	1233.5746	L	373.2809	187.1441	356.2544	178.6308			3
25	2597.2366	1299.1219	2580.2100	1290.6086	2579.2260	1290.1166	L	260.1969	130.6021	243.1703	122.0888			2
26							K	147.1128	74.0600	130.0863	65.5468			1

MS/MS Fragmentation of **FYAFNPLAGGLLTGK**
Found in **ARK73_HUMAN** in **SwissProt**, Aflatoxin B1 aldehyde reductase member 3 OS=Homo sapiens GN=AKR7A3 PE=1 SV=2

Match to Query 413: 1566.774448 from(784.394500,2+) intensity(14022.1000) index(509)
Data file IS_111911_25a.txt

Click mouse within plot area to zoom in by factor of two about that point
Or, Plot from 100 to 1600 Da Full range
Label all possible matches ○ Label matches used for scoring ◉
Show Y-axis ☐

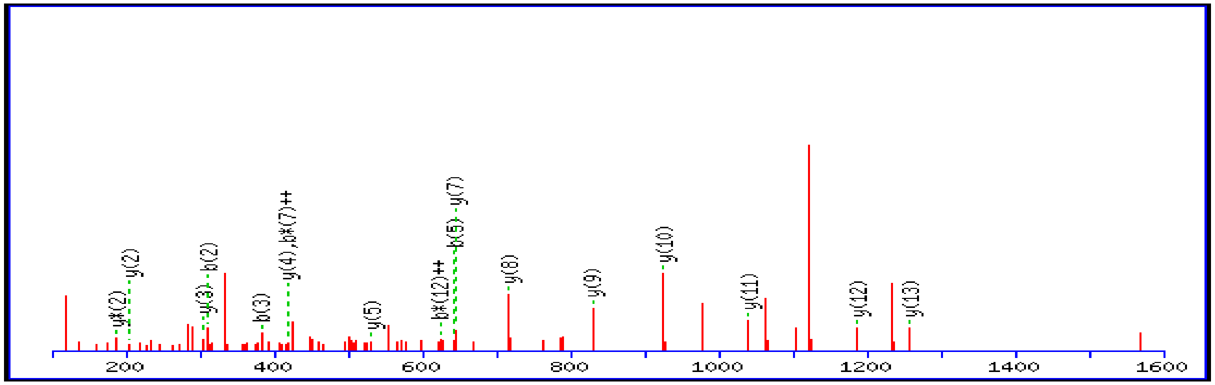

Monoisotopic mass of neutral peptide Mr(calc): 1567.8399
Fixed modifications: Carbamidomethyl (C) (apply to specified residues or termini only)
Ions Score: 43 Expect: 0.0081
Matches : 17/132 fragment ions using 57 most intense peaks (help)

#	b	b++	b*	b*++	b0	b0++	Seq.	y	y++	y*	y*++	y0	y0++	#
1	148.0757	74.5415					F							15
2	311.1390	156.0731					Y	1421.7787	711.3930	1404.7522	702.8797	1403.7682	702.3877	14
3	382.1761	191.5917					A	1258.7154	629.8613	1241.6889	621.3481	1240.7048	620.8561	13
4	529.2445	265.1259					F	1187.6783	594.3428	1170.6517	585.8295	1169.6677	585.3375	12
5	643.2875	322.1474	626.2609	313.6341			N	1040.6099	520.8086	1023.5833	512.2953	1022.5993	511.8033	11
6	740.3402	370.6738	723.3137	362.1605			P	926.5669	463.7871	909.5404	455.2738	908.5564	454.7818	10
7	853.4243	427.2158	836.3978	418.7025			L	829.5142	415.2607	812.4876	406.7475	811.5036	406.2554	9
8	924.4614	462.7343	907.4349	454.2211			A	716.4301	358.7187	699.4036	350.2054	698.4196	349.7134	8
9	981.4829	491.2451	964.4563	482.7318			G	645.3930	323.2001	628.3665	314.6869	627.3824	314.1949	7
10	1038.5043	519.7558	1021.4778	511.2425			G	588.3715	294.6894	571.3450	286.1761	570.3610	285.6841	6
11	1151.5884	576.2978	1134.5619	567.7846			L	531.3501	266.1787	514.3235	257.6654	513.3395	257.1734	5
12	1264.6725	632.8399	1247.6459	624.3266			L	418.2660	209.6366	401.2395	201.1234	400.2554	200.6314	4
13	1365.7202	683.3637	1348.6936	674.8504	1347.7096	674.3584	T	305.1819	153.0946	288.1554	144.5813	287.1714	144.0893	3
14	1422.7416	711.8744	1405.7151	703.3612	1404.7310	702.8692	G	204.1343	102.5708	187.1077	94.0575			2
15							K	147.1128	74.0600	130.0863	65.5468			1

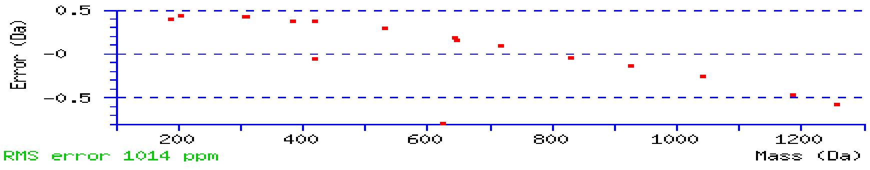

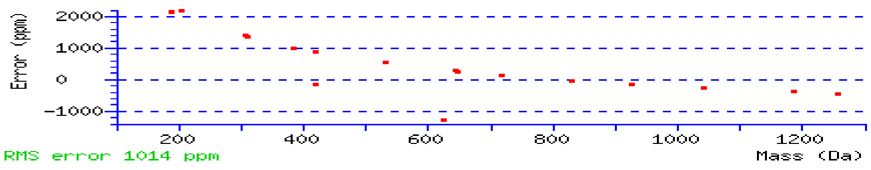

MS/MS Fragmentation of **SILFVPTSAPR**
Found in **ENPL_HUMAN** in **SwissProt**, Endoplasmin OS=Homo sapiens GN=HSP90B1 PE=1 SV=1

Match to Query 270: 1187.099048 from(594.556800,2+) intensity(3334.7000) index(448)
Data file IS_111911_25a.txt

Click mouse within plot area to zoom in by factor of two about that point
Or, Plot from 100 to 1200 Da Full range
Label all possible matches ○ Label matches used for scoring ◉
Show Y-axis ☐

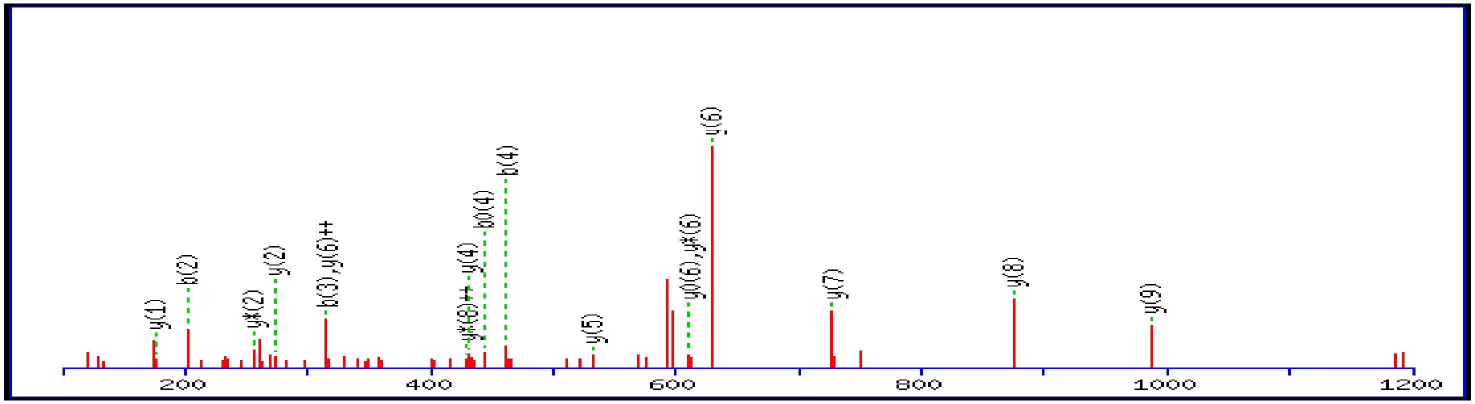

Monoisotopic mass of neutral peptide Mr(calc): 1186.6710
Fixed modifications: Carbamidomethyl (C) (apply to specified residues or termini only)
Ions Score: 42 Expect: 0.0089
Matches : 17/94 fragment ions using 39 most intense peaks (help)

#	b	b^{++}	b^0	b^{0++}	Seq.	y	y^{++}	y^*	y^{*++}	y^0	y^{0++}	#
1	88.0393	44.5233	70.0287	35.5180	S							11
2	**201.1234**	101.0653	183.1128	92.0600	I	1100.6463	550.8268	1083.6197	542.3135	1082.6357	541.8215	10
3	**314.2074**	157.6074	296.1969	148.6021	L	***987.5622***	494.2847	970.5356	485.7715	969.5516	485.2795	9
4	**461.2758**	231.1416	443.2653	222.1363	F	***874.4781***	437.7427	857.4516	429.2294	856.4676	428.7374	8
5	560.3443	280.6758	542.3337	271.6705	V	***727.4097***	364.2085	710.3832	355.6952	709.3991	355.2032	7
6	657.3970	329.2022	639.3865	320.1969	P	***628.3413***	314.6743	611.3148	306.1610	610.3307	305.6690	6
7	758.4447	379.7260	740.4341	370.7207	T	***531.2885***	266.1479	514.2620	257.6346	513.2780	257.1426	5
8	845.4767	423.2420	827.4662	414.2367	S	***430.2409***	215.6241	413.2143	207.1108	412.2303	206.6188	4
9	916.5138	458.7606	898.5033	449.7553	A	343.2088	172.1081	326.1823	163.5948			3
10	1013.5666	507.2869	995.5560	498.2817	P	***272.1717***	136.5895	255.1452	128.0762			2
11					R	***175.1190***	88.0631	158.0924	79.5498			1

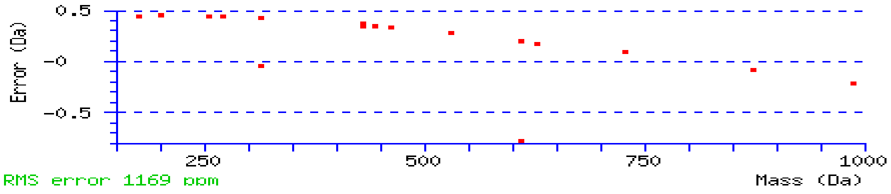

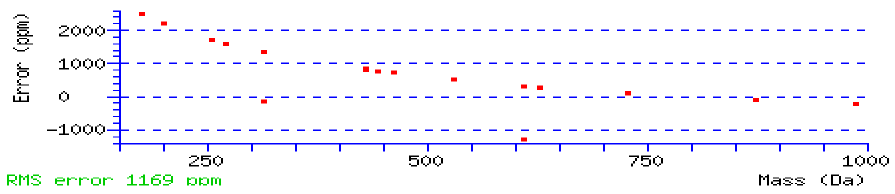

MS/MS Fragmentation of **ALGSVGPVDLLVNNAAVALLQPFLEVTK**
Found in **DCXR_HUMAN** in **SwissProt**, L-xylulose reductase OS=Homo sapiens GN=DCXR PE=1 SV=2

Match to Query 570: 2847.064272 from(950.028700,3+) intensity(651.1000) index(613)
Data file IS_111911_25a.txt

Click mouse within plot area to zoom in by factor of two about that point
Or, Plot from 400 to 1700 Da Full range
Label all possible matches ○ Label matches used for scoring ◉
Show Y-axis ☐

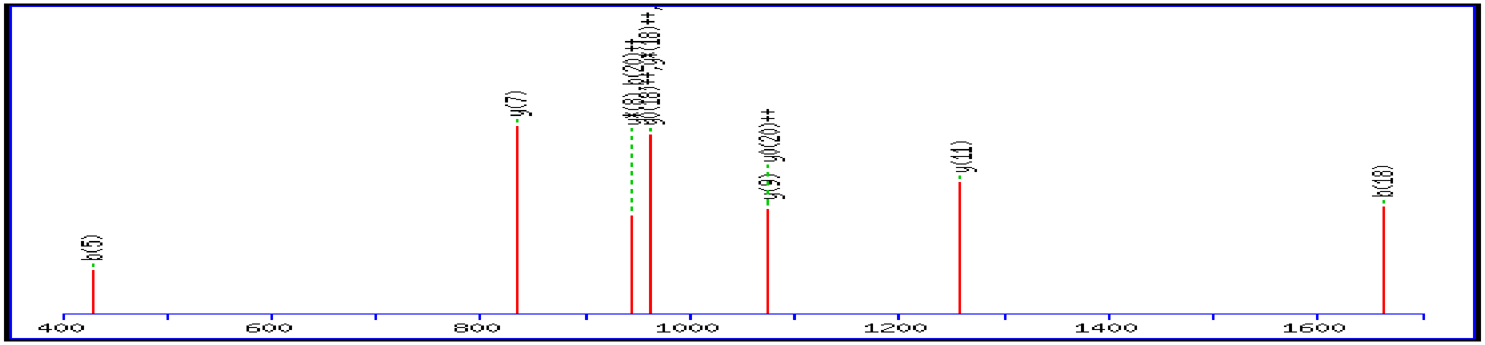

Monoisotopic mass of neutral peptide Mr(calc): 2847.6110
Fixed modifications: Carbamidomethyl (C) (apply to specified residues or termini only)
Ions Score: 34 Expect: 0.023
Matches : 11/292 fragment ions using 8 most intense peaks (help)

#	b	b^{++}	b^{*}	b^{*++}	b^{0}	b^{0++}	Seq.	y	y^{++}	y^{*}	y^{*++}	y^{0}	y^{0++}	#
1	72.0444	36.5258					A							28
2	185.1285	93.0679					L	2777.5811	1389.2942	2760.5546	1380.7809	2759.5706	1380.2889	27
3	242.1499	121.5786					G	2664.4971	1332.7522	2647.4705	1324.2389	2646.4865	1323.7469	26
4	329.1819	165.0946			311.1714	156.0893	S	2607.4756	1304.2414	2590.4491	1295.7282	2589.4651	1295.2362	25
5	***428.2504***	214.6288			410.2398	205.6235	V	2520.4436	1260.7254	2503.4170	1252.2122	2502.4330	1251.7201	24
6	485.2718	243.1395			467.2613	234.1343	G	2421.3752	1211.1912	2404.3486	1202.6780	2403.3646	1202.1859	23
7	582.3246	291.6659			564.3140	282.6606	P	2364.3537	1182.6805	2347.3272	1174.1672	2346.3431	1173.6752	22
8	681.3930	341.2001			663.3824	332.1949	V	2267.3009	1134.1541	2250.2744	1125.6408	2249.2904	1125.1488	21
9	796.4199	398.7136			778.4094	389.7083	D	2168.2325	1084.6199	2151.2060	1076.1066	2150.2220	1075.6146	20
10	909.5040	455.2556			891.4934	446.2504	L	2053.2056	1027.1064	2036.1790	1018.5932	2035.1950	1018.1012	19
11	1022.5881	511.7977			1004.5775	502.7924	L	1940.1215	970.5644	1923.0950	962.0511	1922.1110	961.5591	18
12	1121.6565	561.3319			1103.6459	552.3266	V	1827.0375	914.0224	1810.0109	905.5091	1809.0269	905.0171	17
13	1235.6994	618.3533	1218.6729	609.8401	1217.6888	609.3481	N	1727.9690	864.4882	1710.9425	855.9749	1709.9585	855.4829	16
14	1349.7423	675.3748	1332.7158	666.8615	1331.7318	666.3695	N	1613.9261	807.4667	1596.8996	798.9534	1595.9156	798.4614	15
15	1420.7795	710.8934	1403.7529	702.3801	1402.7689	701.8881	A	1499.8832	750.4452	1482.8566	741.9320	1481.8726	741.4400	14
16	1491.8166	746.4119	1474.7900	737.8986	1473.8060	737.4066	A	1428.8461	714.9267	1411.8195	706.4134	1410.8355	705.9214	13
17	1590.8850	795.9461	1573.8584	787.4329	1572.8744	786.9408	V	1357.8090	679.4081	1340.7824	670.8948	1339.7984	670.4028	12
18	***1661.9221***	831.4647	1644.8955	822.9514	1643.9115	822.4594	A	***1258.7406***	629.8739	1241.7140	621.3606	1240.7300	620.8686	11
19	1775.0062	888.0067	1757.9796	879.4934	1756.9956	879.0014	L	1187.7034	594.3554	1170.6769	585.8421	1169.6929	585.3501	10
20	1888.0902	944.5488	1871.0637	936.0355	1870.0797	935.5435	L	***1074.6194***	537.8133	1057.5928	529.3001	1056.6088	528.8080	9
21	2016.1488	1008.5780	1999.1223	1000.0648	1998.1382	999.5728	Q	***961.5353***	481.2713	944.5088	472.7580	943.5247	472.2660	8
22	2113.2016	1057.1044	2096.1750	1048.5911	2095.1910	1048.0991	P	***833.4767***	417.2420	816.4502	408.7287	815.4662	408.2367	7
23	2260.2700	1130.6386	2243.2434	1122.1254	2242.2594	1121.6333	F	736.4240	368.7156	719.3974	360.2023	718.4134	359.7103	6
24	2373.3540	1187.1807	2356.3275	1178.6674	2355.3435	1178.1754	L	589.3556	295.1814	572.3290	286.6681	571.3450	286.1761	5
25	2502.3966	1251.7020	2485.3701	1243.1887	2484.3861	1242.6967	E	476.2715	238.6394	459.2449	230.1261	458.2609	229.6341	4
26	2601.4651	1301.2362	2584.4385	1292.7229	2583.4545	1292.2309	V	347.2289	174.1181	330.2023	165.6048	329.2183	165.1128	3
27	2702.5127	1351.7600	2685.4862	1343.2467	2684.5022	1342.7547	T	248.1605	124.5839	231.1339	116.0706	230.1499	115.5786	2
28							K	147.1128	74.0600	130.0863	65.5468			1

MS/MS Fragmentation of **LIIVSNPVDILTYVAWK**
Found in **LDH6B_HUMAN** in **SwissProt**, L-lactate dehydrogenase A-like 6B OS=Homo sapiens GN=LDHAL6B PE=1 SV=3

Match to Query 483: 1942.680448 from(972.347500,2+) intensity(3916.8000) index(156)
Data file IS_111911_25a.txt

Click mouse within plot area to zoom in by factor of two about that point
Or, Plot from 100 to 1400 Da Full range
Label all possible matches ○ Label matches used for scoring ◉
Show Y-axis ☐

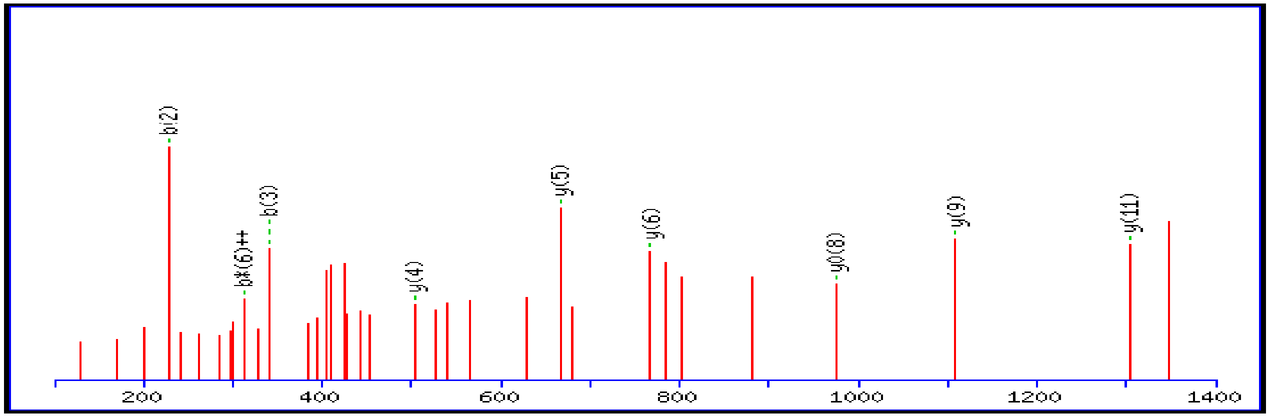


```
Monoisotopic mass of neutral peptide Mr(calc): 1943.1132
Fixed modifications: Carbamidomethyl (C) (apply to specified residues or termini only)
Ions Score: 31  Expect: 0.1
Matches : 9/164 fragment ions using 12 most intense peaks   (help)
```

#	b	b^{++}	b^*	b^{*++}	b^0	b^{0++}	Seq.	y	y^{++}	y^*	y^{*++}	y^0	y^{0++}	#
1	114.0913	57.5493					L							17
2	**227.1754**	114.0913					I	1831.0364	916.0218	1814.0099	907.5086	1813.0258	907.0166	16
3	**340.2595**	170.6334					I	1717.9523	859.4798	1700.9258	850.9665	1699.9418	850.4745	15
4	439.3279	220.1676					V	1604.8683	802.9378	1587.8417	794.4245	1586.8577	793.9325	14
5	526.3599	263.6836			508.3493	254.6783	S	1505.7999	753.4036	1488.7733	744.8903	1487.7893	744.3983	13
6	640.4028	320.7051	623.3763	312.1918	622.3923	311.6998	N	1418.7678	709.8876	1401.7413	701.3743	1400.7573	700.8823	12
7	737.4556	369.2314	720.4291	360.7182	719.4450	360.2262	P	***1304.7249***	652.8661	1287.6984	644.3528	1286.7143	643.8608	11
8	836.5240	418.7656	819.4975	410.2524	818.5134	409.7604	V	1207.6721	604.3397	1190.6456	595.8264	1189.6616	595.3344	10
9	951.5510	476.2791	934.5244	467.7658	933.5404	467.2738	D	***1108.6037***	554.8055	1091.5772	546.2922	1090.5932	545.8002	9
10	1064.6350	532.8211	1047.6085	524.3079	1046.6245	523.8159	I	993.5768	497.2920	976.5502	488.7788	975.5662	488.2867	8
11	1177.7191	589.3632	1160.6925	580.8499	1159.7085	580.3579	L	880.4927	440.7500	863.4662	432.2367	862.4822	431.7447	7
12	1278.7668	639.8870	1261.7402	631.3737	1260.7562	630.8817	T	***767.4087***	384.2080	750.3821	375.6947	749.3981	375.2027	6
13	1441.8301	721.4187	1424.8035	712.9054	1423.8195	712.4134	Y	***666.3610***	333.6841	649.3344	325.1709			5
14	1540.8985	770.9529	1523.8720	762.4396	1522.8879	761.9476	V	***503.2976***	252.1525	486.2711	243.6392			4
15	1611.9356	806.4714	1594.9091	797.9582	1593.9251	797.4662	A	404.2292	202.6183	387.2027	194.1050			3
16	1798.0149	899.5111	1780.9884	890.9978	1780.0044	890.5058	W	333.1921	167.0997	316.1656	158.5864			2
17							K	147.1128	74.0600	130.0863	65.5468			1

MS/MS Fragmentation of **GLGTDEDTLIEILASR**
Found in **ANXA1_HUMAN** in **SwissProt**, Annexin A1 OS=Homo sapiens GN=ANXA1 PE=1 SV=2

Match to Query 416: 1701.755448 from(851.885000,2+) intensity(9742.2000) index(226)
Data file IS_111911_25b.txt

Click mouse within plot area to zoom in by factor of two about that point
Or, Plot from 100 to 1600 Da Full range
Label all possible matches ○ Label matches used for scoring ◉
Show Y-axis ☐

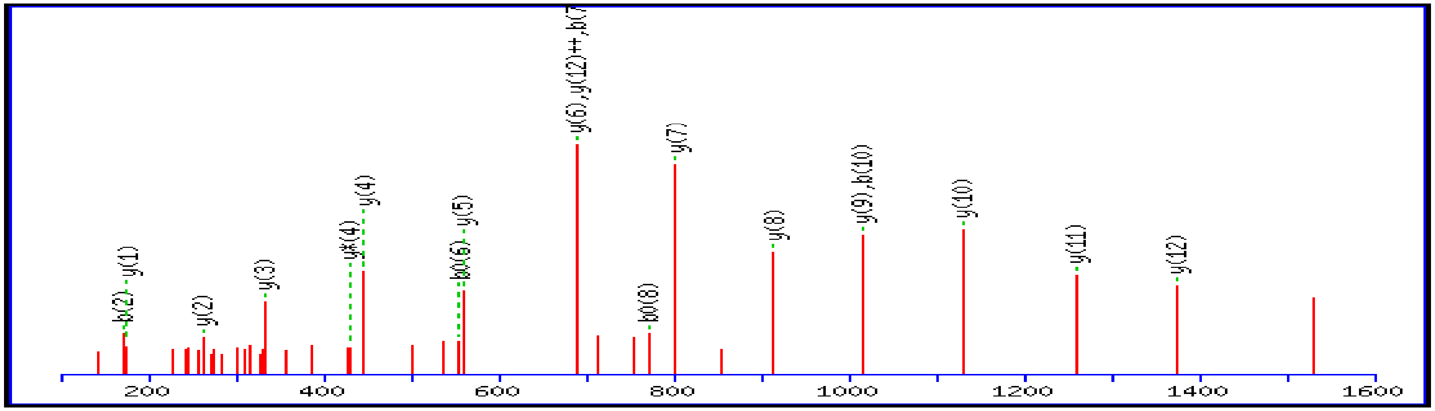

Monoisotopic mass of neutral peptide Mr(calc): 1701.8785
Fixed modifications: Carbamidomethyl (C) (apply to specified residues or termini only)
Ions Score: 112 Expect: 9.7e-10
Matches : 19/142 fragment ions using 22 most intense peaks (help)

#	b	b++	b0	b0++	Seq.	y	y++	y*	y*++	y0	y0++	#
1	58.0287	29.5180			G							16
2	171.1128	86.0600			L	1645.8643	823.4358	1628.8378	814.9225	1627.8537	814.4305	15
3	228.1343	114.5708			G	1532.7802	766.8938	1515.7537	758.3805	1514.7697	757.8885	14
4	329.1819	165.0946	311.1714	156.0893	T	1475.7588	738.3830	1458.7322	729.8698	1457.7482	729.3777	13
5	444.2089	222.6081	426.1983	213.6028	D	1374.7111	687.8592	1357.6846	679.3459	1356.7005	678.8539	12
6	573.2515	287.1294	555.2409	278.1241	E	1259.6842	630.3457	1242.6576	621.8324	1241.6736	621.3404	11
7	688.2784	344.6429	670.2679	335.6376	D	1130.6416	565.8244	1113.6150	557.3111	1112.6310	556.8191	10
8	789.3261	395.1667	771.3155	386.1614	T	1015.6146	508.3109	998.5881	499.7977	997.6041	499.3057	9
9	902.4102	451.7087	884.3996	442.7034	L	914.5669	457.7871	897.5404	449.2738	896.5564	448.7818	8
10	1015.4942	508.2508	997.4837	499.2455	I	801.4829	401.2451	784.4563	392.7318	783.4723	392.2398	7
11	1144.5368	572.7721	1126.5263	563.7668	E	688.3988	344.7030	671.3723	336.1898	670.3882	335.6978	6
12	1257.6209	629.3141	1239.6103	620.3088	I	559.3562	280.1817	542.3297	271.6685	541.3457	271.1765	5
13	1370.7050	685.8561	1352.6944	676.8508	L	446.2722	223.6397	429.2456	215.1264	428.2616	214.6344	4
14	1441.7421	721.3747	1423.7315	712.3694	A	333.1881	167.0977	316.1615	158.5844	315.1775	158.0924	3
15	1528.7741	764.8907	1510.7635	755.8854	S	262.1510	131.5791	245.1244	123.0659	244.1404	122.5738	2
16					R	175.1190	88.0631	158.0924	79.5498			1

MS/MS Fragmentation of ETSGNLEQLLLAVVK
Found in ANXA5_HUMAN in SwissProt, Annexin A5 OS=Homo sapiens GN=ANXA5 PE=1 SV=2

Match to Query 395: 1612.888248 from(807.451400,2+) intensity(9910.0000) index(250)
Data file IS_111911_25b.txt

Click mouse within plot area to zoom in by factor of two about that point
Or, Plot from 100 to 1400 Da Full range
Label all possible matches ○ Label matches used for scoring ◉
Show Y-axis ☐

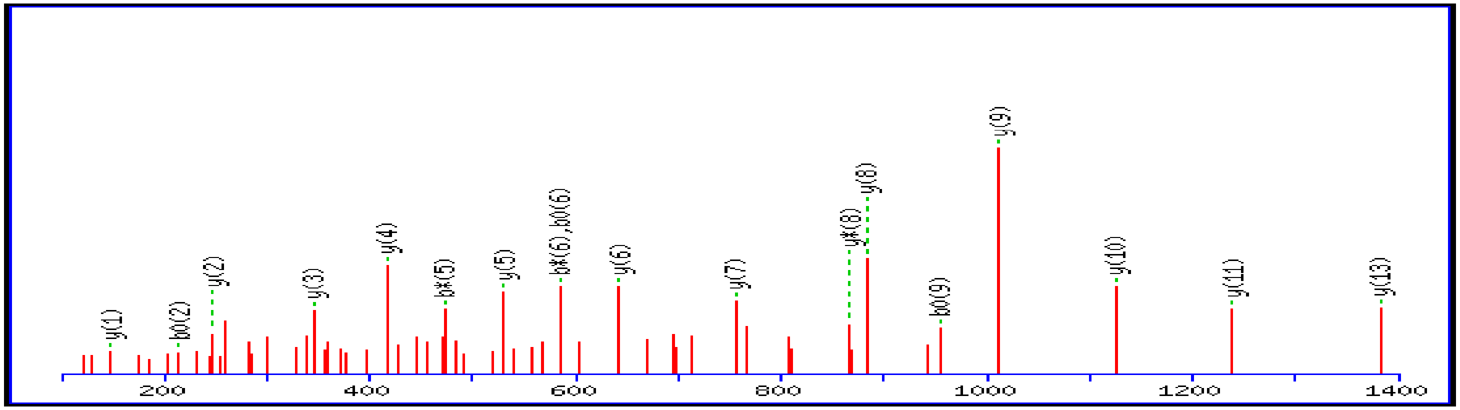

Monoisotopic mass of neutral peptide Mr(calc): 1612.9036
Fixed modifications: Carbamidomethyl (C) (apply to specified residues or termini only)
Ions Score: 102 Expect: 9.9e-09
Matches : 18/144 fragment ions using 22 most intense peaks (help)

#	b	b^{++}	b^{*}	b^{*++}	b^{0}	b^{0++}	Seq.	y	y^{++}	y^{*}	y^{*++}	y^{0}	y^{0++}	#
1	130.0499	65.5286			112.0393	56.5233	E							15
2	231.0975	116.0524			213.0870	107.0471	T	1484.8683	742.9378	1467.8417	734.4245	1466.8577	733.9325	14
3	318.1296	159.5684			300.1190	150.5631	S	1383.8206	692.4139	1366.7940	683.9007	1365.8100	683.4087	13
4	375.1510	188.0792			357.1405	179.0739	G	1296.7886	648.8979	1279.7620	640.3846	1278.7780	639.8926	12
5	489.1940	245.1006	472.1674	236.5873	471.1834	236.0953	N	1239.7671	620.3872	1222.7406	611.8739	1221.7565	611.3819	11
6	602.2780	301.6427	585.2515	293.1294	584.2675	292.6374	L	1125.7242	563.3657	1108.6976	554.8525	1107.7136	554.3604	10
7	731.3206	366.1640	714.2941	357.6507	713.3101	357.1587	E	1012.6401	506.8237	995.6136	498.3104	994.6295	497.8184	9
8	859.3792	430.1932	842.3527	421.6800	841.3686	421.1880	Q	883.5975	442.3024	866.5710	433.7891			8
9	972.4633	486.7353	955.4367	478.2220	954.4527	477.7300	L	755.5389	378.2731	738.5124	369.7598			7
10	1085.5473	543.2773	1068.5208	534.7640	1067.5368	534.2720	L	642.4549	321.7311	625.4283	313.2178			6
11	1198.6314	599.8193	1181.6048	591.3061	1180.6208	590.8141	L	529.3708	265.1890	512.3443	256.6758			5
12	1269.6685	635.3379	1252.6420	626.8246	1251.6579	626.3326	A	416.2867	208.6470	399.2602	200.1337			4
13	1368.7369	684.8721	1351.7104	676.3588	1350.7264	675.8668	V	345.2496	173.1285	328.2231	164.6152			3
14	1467.8053	734.4063	1450.7788	725.8930	1449.7948	725.4010	V	246.1812	123.5942	229.1547	115.0810			2
15							K	147.1128	74.0600	130.0863	65.5468			1

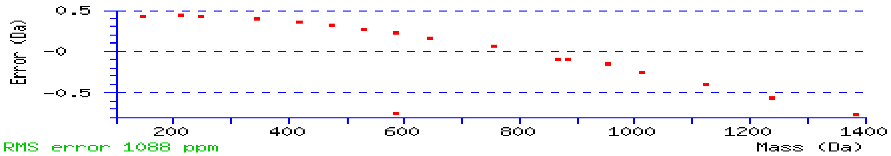

RMS error 1088 ppm

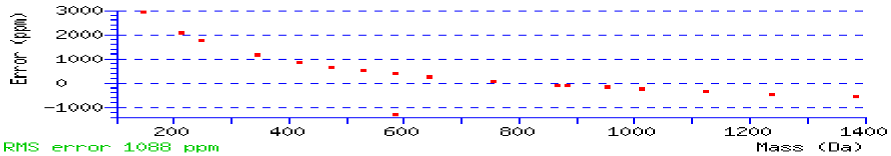

RMS error 1088 ppm

MS/MS Fragmentation of **ISSIQSIVPALEIANAHR**
Found in **CH60_HUMAN** in **SwissProt**, 60 kDa heat shock protein, mitochondrial OS=Homo sapiens GN=HSPD1 PE=1 SV=2

Match to Query 452: 1918.565472 from(640.529100,3+) intensity(8690.7000) index(463)
Data file IS_111911_25b.txt

Click mouse within plot area to zoom in by factor of two about that point
Or, Plot from 100 to 2000 Da Full range
Label all possible matches ○ Label matches used for scoring ◉
Show Y-axis ☐

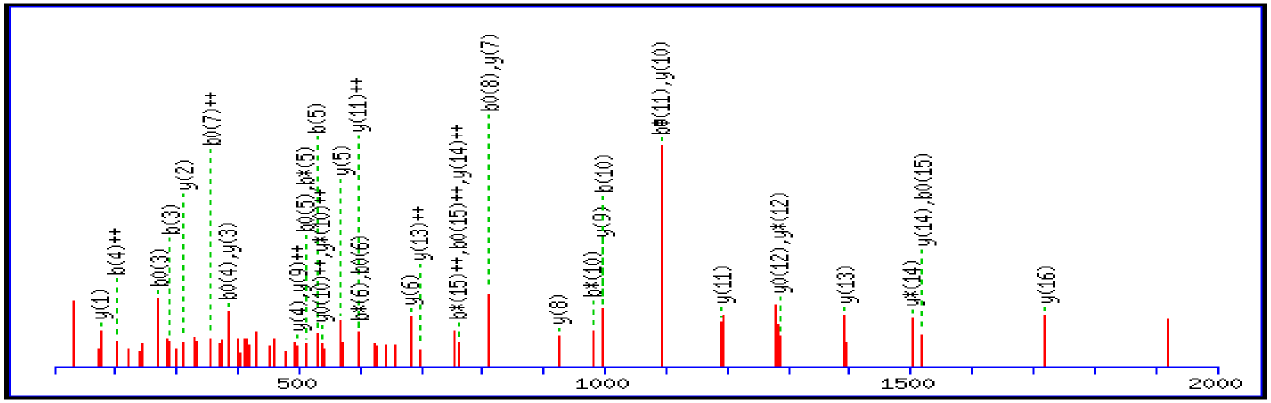


```
Monoisotopic mass of neutral peptide Mr(calc): 1918.0636
Fixed modifications: Carbamidomethyl (C) (apply to specified residues or termini only)
Ions Score: 95  Expect: 3.4e-08
Matches : 42/182 fragment ions using 57 most intense peaks   (help)
```

#	b	b^{++}	b^*	b^{*++}	b^0	b^{0++}	Seq.	y	y^{++}	y^*	y^{*++}	y^0	y^{0++}	#
1	114.0913	57.5493					I							18
2	201.1234	101.0653			183.1128	92.0600	S	1805.9868	903.4970	1788.9603	894.9838	1787.9763	894.4918	17
3	288.1554	144.5813			270.1448	135.5761	S	1718.9548	859.9810	1701.9282	851.4678	1700.9442	850.9758	16
4	401.2395	201.1234			383.2289	192.1181	I	1631.9228	816.4650	1614.8962	807.9517	1613.9122	807.4597	15
5	529.2980	265.1527	512.2715	256.6394	511.2875	256.1474	Q	1518.8387	759.9230	1501.8122	751.4097	1500.8281	750.9177	14
6	616.3301	308.6687	599.3035	300.1554	598.3195	299.6634	S	1390.7801	695.8937	1373.7536	687.3804	1372.7696	686.8884	13
7	729.4141	365.2107	712.3876	356.6974	711.4036	356.2054	I	1303.7481	652.3777	1286.7215	643.8644	1285.7375	643.3724	12
8	828.4825	414.7449	811.4560	406.2316	810.4720	405.7396	V	1190.6640	595.8357	1173.6375	587.3224	1172.6535	586.8304	11
9	925.5353	463.2713	908.5088	454.7580	907.5247	454.2660	P	1091.5956	546.3014	1074.5691	537.7882	1073.5851	537.2962	10
10	996.5724	498.7898	979.5459	490.2766	978.5619	489.7846	A	994.5429	497.7751	977.5163	489.2618	976.5323	488.7698	9
11	1109.6565	555.3319	1092.6299	546.8186	1091.6459	546.3266	L	923.5057	462.2565	906.4792	453.7432	905.4952	453.2512	8
12	1238.6991	619.8532	1221.6725	611.3399	1220.6885	610.8479	E	810.4217	405.7145	793.3951	397.2012	792.4111	396.7092	7
13	1351.7831	676.3952	1334.7566	667.8819	1333.7726	667.3899	I	681.3791	341.1932	664.3525	332.6799			6
14	1422.8203	711.9138	1405.7937	703.4005	1404.8097	702.9085	A	568.2950	284.6511	551.2685	276.1379			5
15	1536.8632	768.9352	1519.8366	760.4220	1518.8526	759.9299	N	497.2579	249.1326	480.2314	240.6193			4
16	1607.9003	804.4538	1590.8737	795.9405	1589.8897	795.4485	A	383.2150	192.1111	366.1884	183.5979			3
17	1744.9592	872.9832	1727.9327	864.4700	1726.9486	863.9780	H	312.1779	156.5926	295.1513	148.0793			2
18							R	175.1190	88.0631	158.0924	79.5498			1

MS/MS Fragmentation of **APSTYGGGLSVSSSR**
Found in **K1C14_HUMAN** in **SwissProt**, Keratin, type I cytoskeletal 14 OS=Homo sapiens GN=KRT14 PE=1 SV=4

Match to Query 325: 1424.868648 from(713.441600,2+) intensity(4015.8000) index(97)
Data file IS_111911_25b.txt

Click mouse within plot area to zoom in by factor of two about that point
Or, Plot from 100 to 1500 Da Full range
Label all possible matches ○ Label matches used for scoring ◉
Show Y-axis ☐

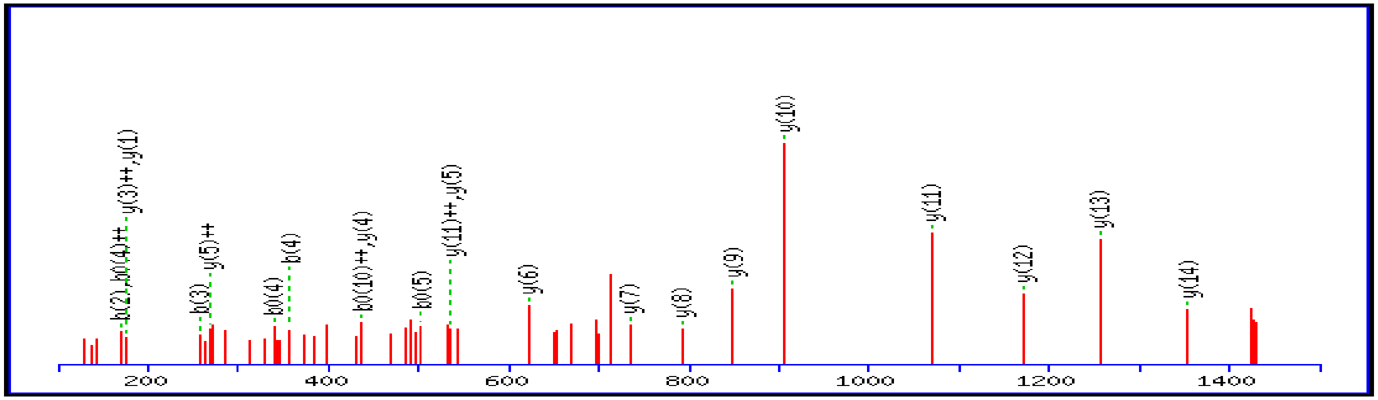

Monoisotopic mass of neutral peptide Mr(calc): 1424.6896
Fixed modifications: Carbamidomethyl (C) (apply to specified residues or termini only)
Ions Score: 90 Expect: 1.9e-07
Matches : 22/134 fragment ions using 35 most intense peaks (help)

#	b	b++	b0	b0++	Seq.	y	y++	y*	y*++	y0	y0++	#
1	72.0444	36.5258			A							15
2	169.0972	85.0522			P	1354.6597	677.8335	1337.6332	669.3202	1336.6492	668.8282	14
3	256.1292	128.5682	238.1186	119.5629	S	1257.6070	629.3071	1240.5804	620.7938	1239.5964	620.3018	13
4	357.1769	179.0921	339.1663	170.0868	T	1170.5749	585.7911	1153.5484	577.2778	1152.5644	576.7858	12
5	520.2402	260.6237	502.2296	251.6185	Y	1069.5273	535.2673	1052.5007	526.7540	1051.5167	526.2620	11
6	577.2617	289.1345	559.2511	280.1292	G	906.4639	453.7356	889.4374	445.2223	888.4534	444.7303	10
7	634.2831	317.6452	616.2726	308.6399	G	849.4425	425.2249	832.4159	416.7116	831.4319	416.2196	9
8	691.3046	346.1559	673.2940	337.1506	G	792.4210	396.7141	775.3945	388.2009	774.4104	387.7089	8
9	804.3886	402.6980	786.3781	393.6927	L	735.3995	368.2034	718.3730	359.6901	717.3890	359.1981	7
10	891.4207	446.2140	873.4101	437.2087	S	622.3155	311.6614	605.2889	303.1481	604.3049	302.6561	6
11	990.4891	495.7482	972.4785	486.7429	V	535.2835	268.1454	518.2569	259.6321	517.2729	259.1401	5
12	1077.5211	539.2642	1059.5106	530.2589	S	436.2150	218.6112	419.1885	210.0979	418.2045	209.6059	4
13	1164.5531	582.7802	1146.5426	573.7749	S	349.1830	175.0951	332.1565	166.5819	331.1724	166.0899	3
14	1251.5852	626.2962	1233.5746	617.2909	S	262.1510	131.5791	245.1244	123.0659	244.1404	122.5738	2
15					R	175.1190	88.0631	158.0924	79.5498			1

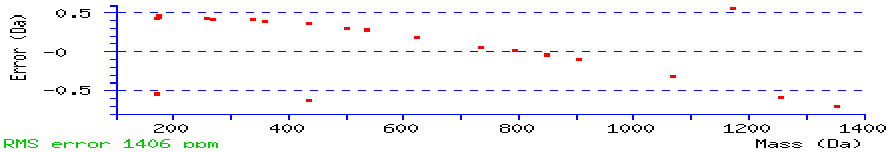

RMS error 1406 ppm

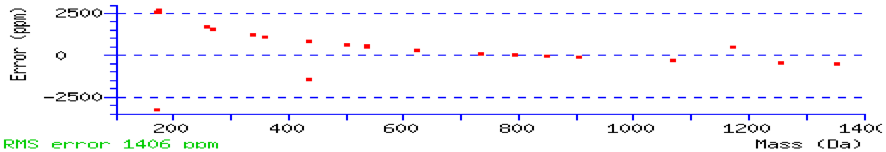

RMS error 1406 ppm

MS/MS Fragmentation of VAGHPNIVINNAAGNFISPTER
Found in DECR_HUMAN in SwissProt, 2,4-dienoyl-CoA reductase, mitochondrial OS=Homo sapiens GN=DECR1 PE=1 SV=1

Match to Query 516: 2290.314372 from(764.445400,3+) intensity(5313.8000) index(425)
Data file IS_111911_25b.txt

Click mouse within plot area to zoom in by factor of two about that point
Or, Plot from 0 to 2400 Da Full range
Label all possible matches ○ Label matches used for scoring ◉
Show Y-axis ☐

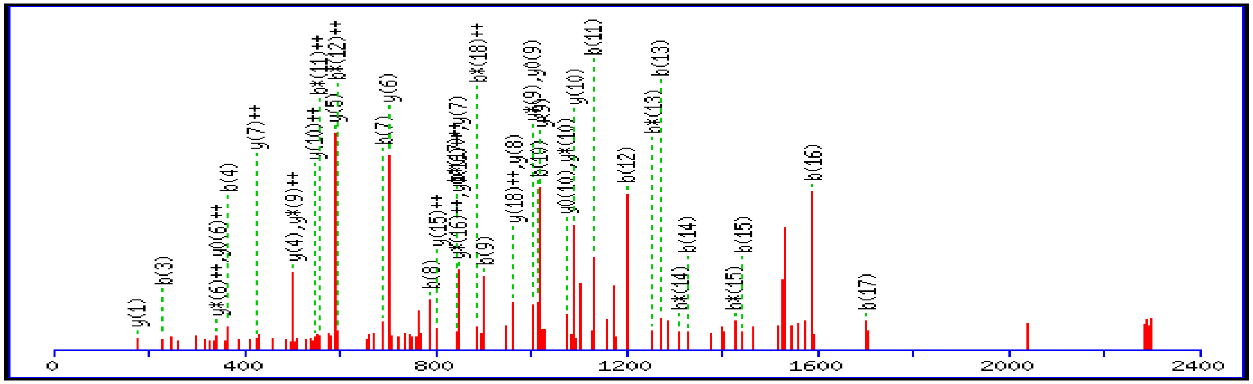

Monoisotopic mass of neutral peptide Mr(calc): 2290.1818
Fixed modifications: Carbamidomethyl (C) (apply to specified residues or termini only)
Ions Score: 75 Expect: 3.6e-06
Matches : 41/206 fragment ions using 63 most intense peaks (help)

#	b	b^{++}	b^{*}	b^{*++}	b^{0}	b^{0++}	Seq.	y	y^{++}	y^{*}	y^{*++}	y^{0}	y^{0++}	#
1	100.0757	50.5415					V							22
2	171.1128	86.0600					A	2192.1207	1096.5640	2175.0941	1088.0507	2174.1101	1087.5587	21
3	228.1343	114.5708					G	2121.0836	1061.0454	2104.0570	1052.5322	2103.0730	1052.0401	20
4	365.1932	183.1002					H	2064.0621	1032.5347	2047.0356	1024.0214	2046.0515	1023.5294	19
5	462.2459	231.6266					P	1927.0032	964.0052	1909.9767	955.4920	1908.9926	955.0000	18
6	576.2889	288.6481	559.2623	280.1348			N	1829.9504	915.4789	1812.9239	906.9656	1811.9399	906.4736	17
7	689.3729	345.1901	672.3464	336.6768			I	1715.9075	858.4574	1698.8810	849.9441	1697.8969	849.4521	16
8	788.4413	394.7243	771.4148	386.2110			V	1602.8234	801.9154	1585.7969	793.4021	1584.8129	792.9101	15
9	901.5254	451.2663	884.4989	442.7531			I	1503.7550	752.3812	1486.7285	743.8679	1485.7445	743.3759	14
10	1015.5683	508.2878	998.5418	499.7745			N	1390.6710	695.8391	1373.6444	687.3258	1372.6604	686.8338	13
11	1129.6113	565.3093	1112.5847	556.7960			N	1276.6280	638.8177	1259.6015	630.3044	1258.6175	629.8124	12
12	1200.6484	600.8278	1183.6218	592.3146			A	1162.5851	581.7962	1145.5586	573.2829	1144.5745	572.7909	11
13	1271.6855	636.3464	1254.6589	627.8331			A	1091.5480	546.2776	1074.5214	537.7644	1073.5374	537.2724	10
14	1328.7070	664.8571	1311.6804	656.3438			G	1020.5109	510.7591	1003.4843	502.2458	1002.5003	501.7538	9
15	1442.7499	721.8786	1425.7233	713.3653			N	963.4894	482.2483	946.4629	473.7351	945.4789	473.2431	8
16	1589.8183	795.4128	1572.7918	786.8995			F	849.4465	425.2269	832.4199	416.7136	831.4359	416.2216	7
17	1702.9024	851.9548	1685.8758	843.4415			I	702.3781	351.6927	685.3515	343.1794	684.3675	342.6874	6
18	1789.9344	895.4708	1772.9078	886.9576	1771.9238	886.4656	S	589.2940	295.1506	572.2675	286.6374	571.2835	286.1454	5
19	1886.9872	943.9972	1869.9606	935.4839	1868.9766	934.9919	P	502.2620	251.6346	485.2354	243.1214	484.2514	242.6293	4
20	1988.0348	994.5211	1971.0083	986.0078	1970.0243	985.5158	T	405.2092	203.1082	388.1827	194.5950	387.1987	194.1030	3
21	2117.0774	1059.0424	2100.0509	1050.5291	2099.0669	1050.0371	E	304.1615	152.5844	287.1350	144.0711	286.1510	143.5791	2
22							R	175.1190	88.0631	158.0924	79.5498			1

MS/MS Fragmentation of **TLAQLNPESSLFIIASK**
Found in **G6PI_HUMAN** in **SwissProt**, Glucose-6-phosphate isomerase OS=Homo sapiens GN=GPI PE=1 SV=4

Match to Query 435: 1830.734248 from(916.374400,2+) intensity(10559.4000) index(206)
Data file IS_111911_25b.txt

Click mouse within plot area to zoom in by factor of two about that point
Or, Plot from 100 to 1400 Da Full range
Label all possible matches ○ Label matches used for scoring ◉
Show Y-axis ☐

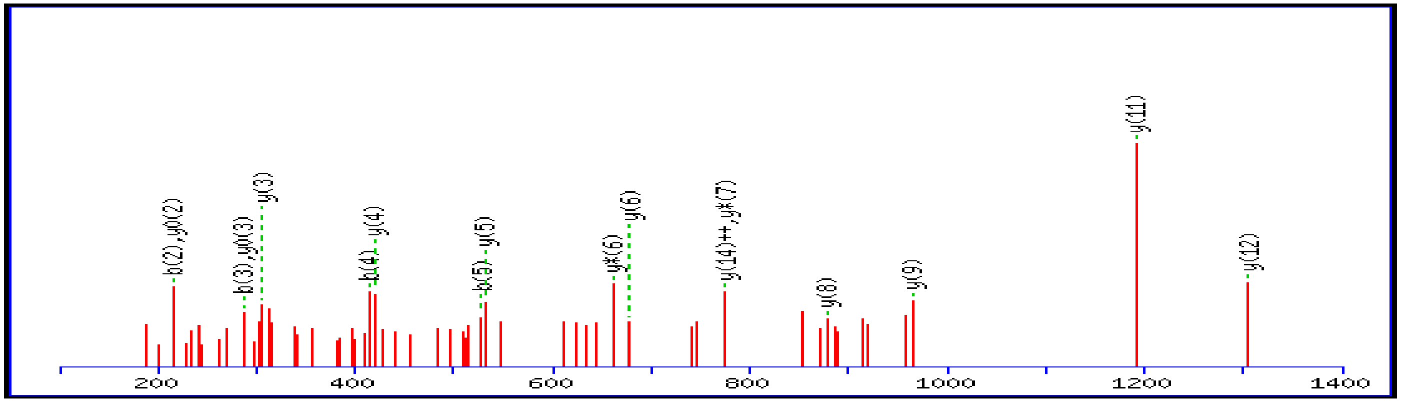

Monoisotopic mass of neutral peptide Mr(calc): 1831.0091
Fixed modifications: Carbamidomethyl (C) (apply to specified residues or termini only)
Ions Score: 63 Expect: 6.7e-05
Matches : 17/184 fragment ions using 19 most intense peaks (help)

#	b	b^{++}	b^{*}	b^{*++}	b^{0}	b^{0++}	Seq.	y	y^{++}	y^{*}	y^{*++}	y^{0}	y^{0++}	#
1	102.0550	51.5311			84.0444	42.5258	T							17
2	215.1390	108.0731			197.1285	99.0679	L	1730.9687	865.9880	1713.9422	857.4747	1712.9581	856.9827	16
3	286.1761	143.5917			268.1656	134.5864	A	1617.8846	809.4460	1600.8581	800.9327	1599.8741	800.4407	15
4	414.2347	207.6210	397.2082	199.1077	396.2241	198.6157	Q	1546.8475	773.9274	1529.8210	765.4141	1528.8370	764.9221	14
5	527.3188	264.1630	510.2922	255.6498	509.3082	255.1577	L	1418.7890	709.8981	1401.7624	701.3848	1400.7784	700.8928	13
6	641.3617	321.1845	624.3352	312.6712	623.3511	312.1792	N	1305.7049	653.3561	1288.6783	644.8428	1287.6943	644.3508	12
7	738.4145	369.7109	721.3879	361.1976	720.4039	360.7056	P	1191.6620	596.3346	1174.6354	587.8213	1173.6514	587.3293	11
8	867.4571	434.2322	850.4305	425.7189	849.4465	425.2269	E	1094.6092	547.8082	1077.5827	539.2950	1076.5986	538.8030	10
9	954.4891	477.7482	937.4625	469.2349	936.4785	468.7429	S	965.5666	483.2869	948.5401	474.7737	947.5560	474.2817	9
10	1041.5211	521.2642	1024.4946	512.7509	1023.5105	512.2589	S	878.5346	439.7709	861.5080	431.2577	860.5240	430.7656	8
11	1154.6052	577.8062	1137.5786	569.2930	1136.5946	568.8009	L	791.5026	396.2549	774.4760	387.7416	773.4920	387.2496	7
12	1301.6736	651.3404	1284.6470	642.8272	1283.6630	642.3352	F	678.4185	339.7129	661.3919	331.1996	660.4079	330.7076	6
13	1414.7577	707.8825	1397.7311	699.3692	1396.7471	698.8772	I	531.3501	266.1787	514.3235	257.6654	513.3395	257.1734	5
14	1527.8417	764.4245	1510.8152	755.9112	1509.8312	755.4192	I	418.2660	209.6366	401.2395	201.1234	400.2554	200.6314	4
15	1598.8788	799.9431	1581.8523	791.4298	1580.8683	790.9378	A	305.1819	153.0946	288.1554	144.5813	287.1714	144.0893	3
16	1685.9109	843.4591	1668.8843	834.9458	1667.9003	834.4538	S	234.1448	117.5761	217.1183	109.0628	216.1343	108.5708	2
17							K	147.1128	74.0600	130.0863	65.5468			1

MS/MS Fragmentation of **LAMQEFMILPVGAANFR**
Found in **ENOA_HUMAN** in **SwissProt**, Alpha-enolase OS=Homo sapiens GN=ENO1 PE=1 SV=2

Match to Query 453: 1938.562048 from(970.288300,2+) intensity(5578.7000) index(191)
Data file IS_111911_25b.txt

Click mouse within plot area to zoom in by factor of two about that point
Or, Plot from 100 to 1400 Da Full range
Label all possible matches ○ Label matches used for scoring ◉
Show Y-axis ☐

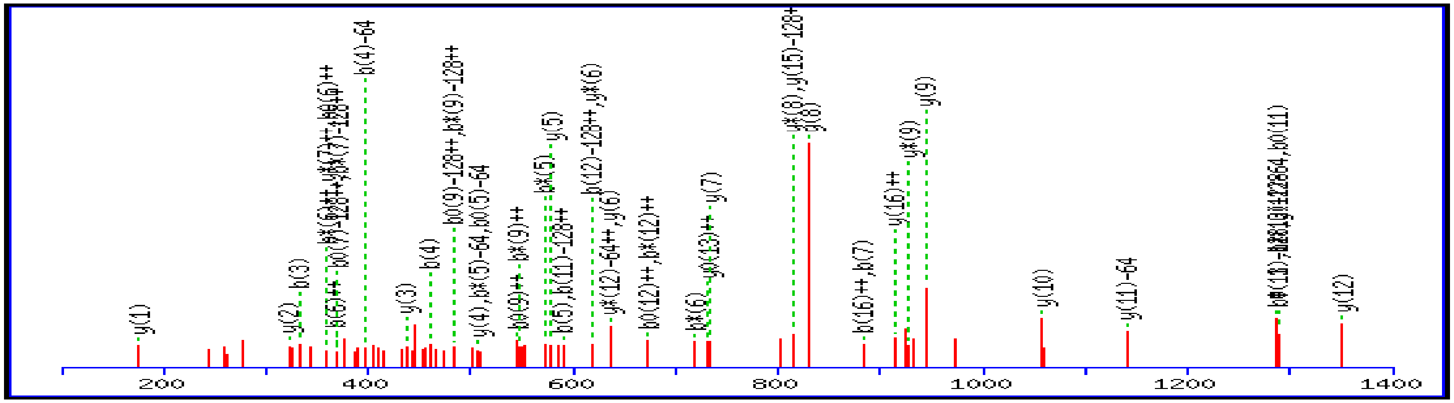

Monoisotopic mass of neutral peptide Mr(calc): 1938.9696
Fixed modifications: Carbamidomethyl (C) (apply to specified residues or termini only)
Variable modifications:
M3 : Oxidation (M), with neutral losses 0.0000(shown in table), 63.9983
M7 : Oxidation (M), with neutral losses 0.0000(shown in table), 63.9983
Ions Score: 62 Expect: 6.7e-05
Matches : 48/264 fragment ions using 56 most intense peaks (help)

#	b	b^{++}	b^{*}	b^{*++}	b^{0}	b^{0++}	Seq.	y	y^{++}	y^{*}	y^{*++}	y^{0}	y^{0++}	#
1	114.0913	57.5493					L							17
2	185.1285	93.0679					A	1826.8928	913.9500	1809.8662	905.4368	1808.8822	904.9448	16
3	**332.1639**	166.5856					M	1755.8557	878.4315	1738.8291	869.9182	1737.8451	869.4262	15
4	**460.2224**	230.6149	443.1959	222.1016			Q	1608.8203	804.9138	1591.7937	796.4005	1590.8097	795.9085	14
5	**589.2650**	295.1362	572.2385	286.6229	571.2545	286.1309	E	1480.7617	740.8845	1463.7351	732.3712	1462.7511	731.8792	13
6	736.3334	368.6704	719.3069	360.1571	718.3229	359.6651	F	***1351.7191***	676.3632	1334.6926	667.8499			12
7	**883.3688**	442.1881	866.3423	433.6748	865.3583	433.1828	M	1204.6507	602.8290	1187.6241	594.3157			11
8	996.4529	498.7301	979.4264	490.2168	978.4423	489.7248	I	***1057.6153***	529.3113	1040.5887	520.7980			10
9	1109.5370	555.2721	1092.5104	546.7588	1091.5264	546.2668	L	***944.5312***	472.7693	927.5047	464.2560			9
10	1206.5897	603.7985	1189.5632	595.2852	1188.5792	594.7932	P	***831.4472***	416.2272	814.4206	407.7139			8
11	1305.6581	653.3327	1288.6316	644.8194	1287.6476	644.3274	V	***734.3944***	367.7008	717.3678	359.1876			7
12	1362.6796	681.8434	1345.6531	673.3302	1344.6690	672.8382	G	***635.3260***	318.1666	618.2994	309.6534			6
13	1433.7167	717.3620	1416.6902	708.8487	1415.7062	708.3567	A	***578.3045***	289.6559	561.2780	281.1426			5
14	1504.7538	752.8806	1487.7273	744.3673	1486.7433	743.8753	A	***507.2674***	254.1373	490.2409	245.6241			4
15	1618.7968	809.9020	1601.7702	801.3887	1600.7862	800.8967	N	***436.2303***	218.6188	419.2037	210.1055			3
16	1765.8652	883.4362	1748.8386	874.9230	1747.8546	874.4309	F	***322.1874***	161.5973	305.1608	153.0840			2
17							R	***175.1190***	88.0631	158.0924	79.5498			1

MS/MS Fragmentation of ESLQQMAEVTR
Found in GLU2B_HUMAN in SwissProt, Glucosidase 2 subunit beta OS=Homo sapiens GN=PRKCSH PE=1 SV=2

Match to Query 278: 1306.917248 from(654.465900,2+) intensity(1010.1000) index(375)
Data file IS_111911_25b.txt

Click mouse within plot area to zoom in by factor of two about that point
Or, Plot from 100 to 1400 Da Full range
Label all possible matches ○ Label matches used for scoring ◉
Show Y-axis ☐

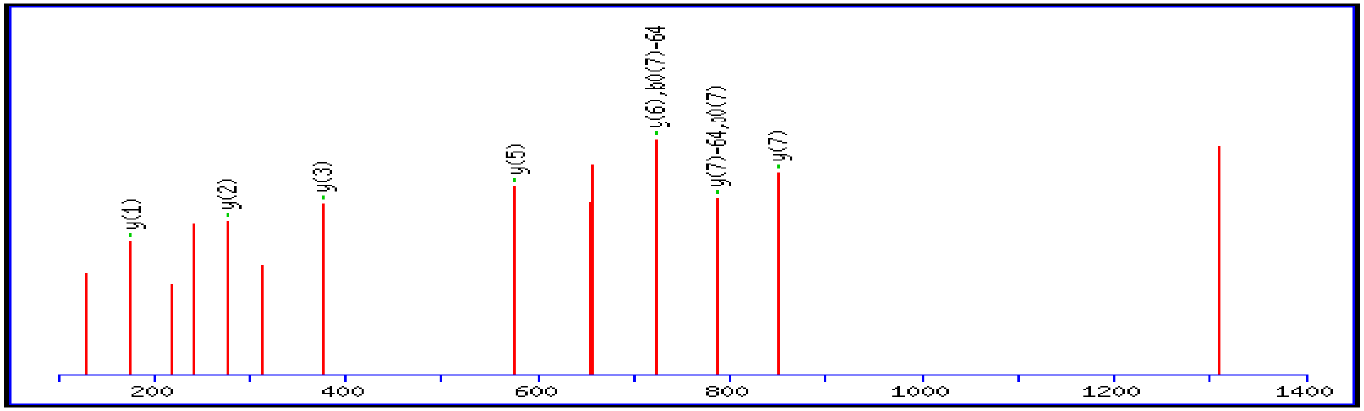

Monoisotopic mass of neutral peptide Mr(calc): 1306.6187
Fixed modifications: Carbamidomethyl (C) (apply to specified residues or termini only)
Variable modifications:
M6 : Oxidation (M), with neutral losses 0.0000(shown in table), 63.9983
Ions Score: 60 Expect: 0.00017
Matches : 9/172 fragment ions using 7 most intense peaks (help)

#	b	b++	b*	b*++	b0	b0++	Seq.	y	y++	y*	y*++	y0	y0++	#
1	130.0499	65.5286			112.0393	56.5233	E							11
2	217.0819	109.0446			199.0713	100.0393	S	1178.5834	589.7953	1161.5569	581.2821	1160.5728	580.7901	10
3	330.1660	165.5866			312.1554	156.5813	L	1091.5514	546.2793	1074.5248	537.7660	1073.5408	537.2740	9
4	458.2245	229.6159	441.1980	221.1026	440.2140	220.6106	Q	978.4673	489.7373	961.4408	481.2240	960.4567	480.7320	8
5	586.2831	293.6452	569.2566	285.1319	568.2726	284.6399	Q	***850.4087***	425.7080	833.3822	417.1947	832.3982	416.7027	7
6	733.3185	367.1629	716.2920	358.6496	715.3080	358.1576	M	***722.3502***	361.6787	705.3236	353.1654	704.3396	352.6734	6
7	804.3556	402.6815	787.3291	394.1682	786.3451	393.6762	A	***575.3148***	288.1610	558.2882	279.6477	557.3042	279.1557	5
8	933.3982	467.2028	916.3717	458.6895	915.3877	458.1975	E	504.2776	252.6425	487.2511	244.1292	486.2671	243.6372	4
9	1032.4666	516.7370	1015.4401	508.2237	1014.4561	507.7317	V	***375.2350***	188.1212	358.2085	179.6079	357.2245	179.1159	3
10	1133.5143	567.2608	1116.4878	558.7475	1115.5038	558.2555	T	***276.1666***	138.5870	259.1401	130.0737	258.1561	129.5817	2
11							R	***175.1190***	88.0631	158.0924	79.5498			1

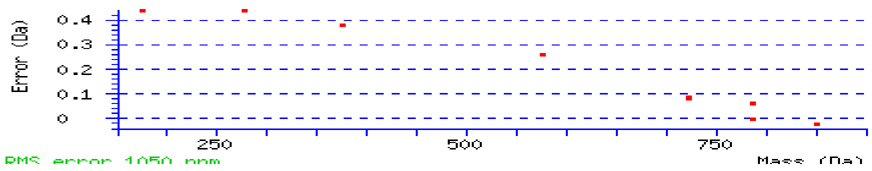

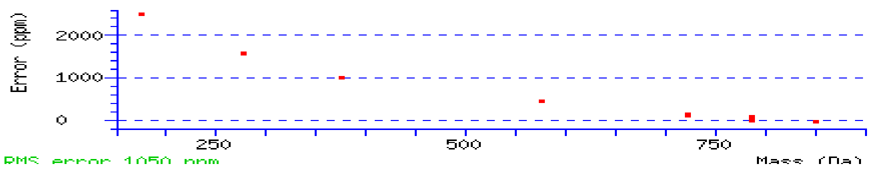

MS/MS Fragmentation of **IGFFQGDIR**
Found in **ACSL1_HUMAN** in **SwissProt**, Long-chain-fatty-acid--CoA ligase 1 OS=Homo sapiens GN=ACSL1 PE=1 SV=1

Match to Query 191: 1052.095848 from(527.055200,2+) intensity(7188.3000) index(433)
Data file IS_111911_25b.txt

Click mouse within plot area to zoom in by factor of two about that point
Or, Plot from 100 to 950 Da Full range
Label all possible matches ○ Label matches used for scoring ◉
Show Y-axis ☐

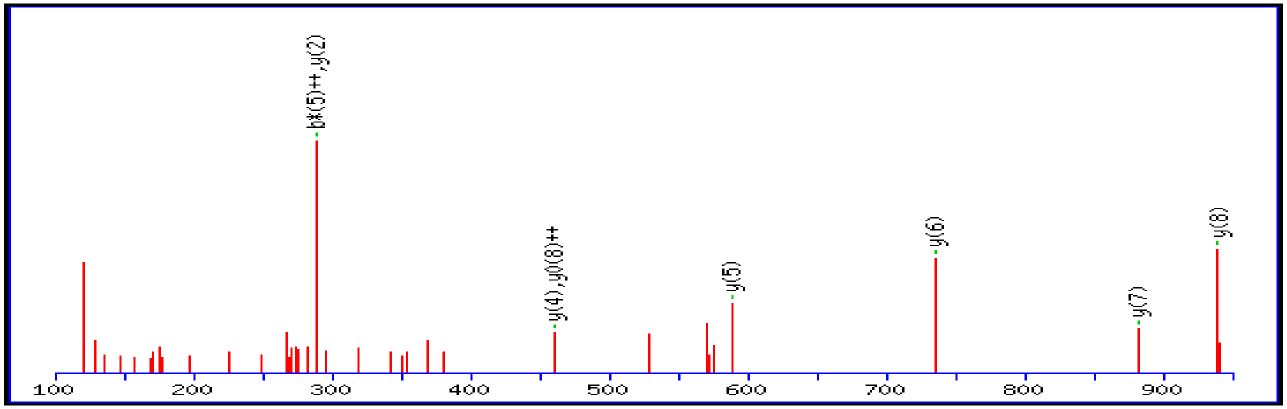

Monoisotopic mass of neutral peptide Mr(calc): 1051.5451
Fixed modifications: Carbamidomethyl (C) (apply to specified residues or termini only)
Ions Score: 52 Expect: 0.001
Matches : 8/72 fragment ions using 8 most intense peaks (help)

#	b	b^{++}	b^{*}	b^{*++}	b^{0}	b^{0++}	Seq.	y	y^{++}	y^{*}	y^{*++}	y^{0}	y^{0++}	#
1	114.0913	57.5493					I							9
2	171.1128	86.0600					G	939.4683	470.2378	922.4417	461.7245	921.4577	461.2325	8
3	318.1812	159.5942					F	882.4468	441.7271	865.4203	433.2138	864.4363	432.7218	7
4	465.2496	233.1285					F	735.3784	368.1928	718.3519	359.6796	717.3678	359.1876	6
5	593.3082	297.1577	576.2817	288.6445			Q	588.3100	294.6586	571.2835	286.1454	570.2994	285.6534	5
6	650.3297	325.6685	633.3031	317.1552			G	460.2514	230.6293	443.2249	222.1161	442.2409	221.6241	4
7	765.3566	383.1819	748.3301	374.6687	747.3461	374.1767	D	403.2300	202.1186	386.2034	193.6053	385.2194	193.1133	3
8	878.4407	439.7240	861.4141	431.2107	860.4301	430.7187	I	288.2030	144.6051	271.1765	136.0919			2
9							R	175.1190	88.0631	158.0924	79.5498			1

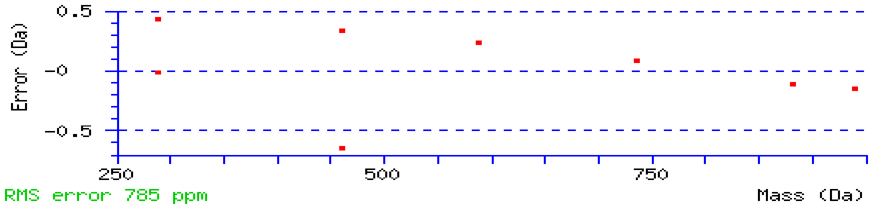

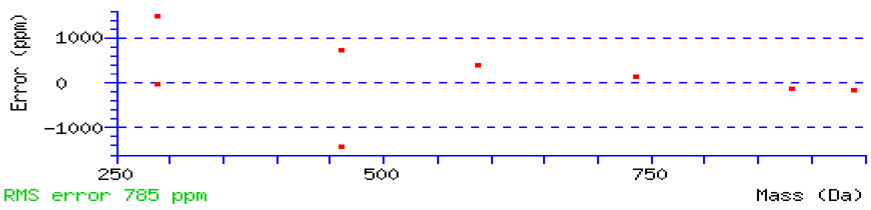

MS/MS Fragmentation of QITLNDLPVGR
Found in PRDX4_HUMAN in SwissProt, Peroxiredoxin-4 OS=Homo sapiens GN=PRDX4 PE=1 SV=1

Match to Query 237: 1225.075848 from(613.545200,2+) intensity(6424.3000) index(428)
Data file IS_111911_25b.txt

Click mouse within plot area to zoom in by factor of two about that point
Or, Plot from 100 to 1300 Da Full range
Label all possible matches ○ Label matches used for scoring ◉
Show Y-axis ☐

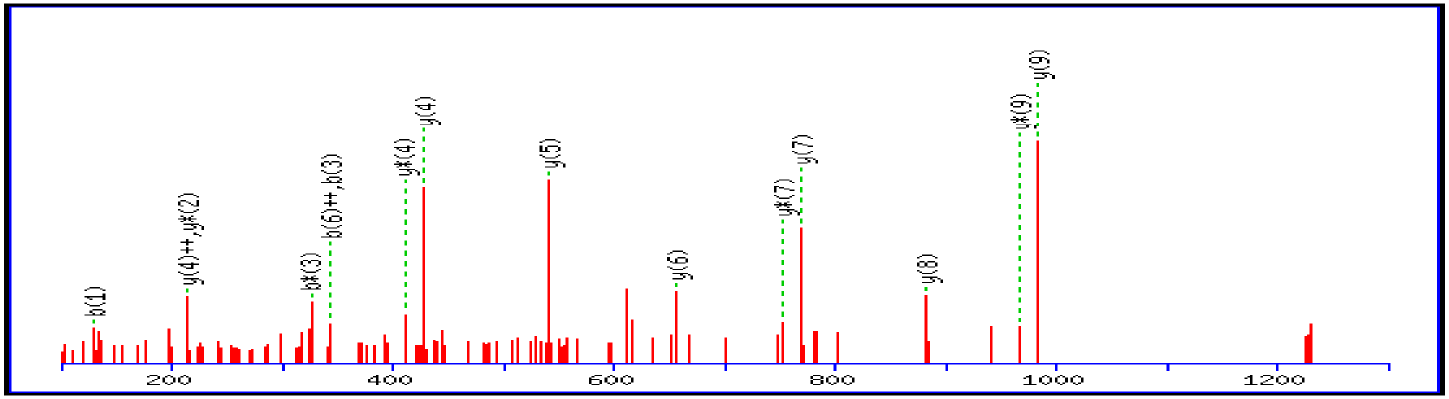

Monoisotopic mass of neutral peptide Mr(calc): 1224.6826
Fixed modifications: Carbamidomethyl (C) (apply to specified residues or termini only)
Ions Score: 43 Expect: 0.0075
Matches : 15/106 fragment ions using 18 most intense peaks (help)

#	b	b++	b*	b*++	b0	b0++	Seq.	y	y++	y*	y*++	y0	y0++	#
1	129.0659	65.0366	112.0393	56.5233			Q							11
2	242.1499	121.5786	225.1234	113.0653			I	1097.6313	549.3193	1080.6048	540.8060	1079.6208	540.3140	10
3	343.1976	172.1024	326.1710	163.5892	325.1870	163.0972	T	984.5473	492.7773	967.5207	484.2640	966.5367	483.7720	9
4	456.2817	228.6445	439.2551	220.1312	438.2711	219.6392	L	883.4996	442.2534	866.4730	433.7402	865.4890	433.2482	8
5	570.3246	285.6659	553.2980	277.1527	552.3140	276.6606	N	770.4155	385.7114	753.3890	377.1981	752.4050	376.7061	7
6	685.3515	343.1794	668.3250	334.6661	667.3410	334.1741	D	656.3726	328.6899	639.3461	320.1767	638.3620	319.6847	6
7	798.4356	399.7214	781.4090	391.2082	780.4250	390.7162	L	541.3457	271.1765	524.3191	262.6632			5
8	895.4884	448.2478	878.4618	439.7345	877.4778	439.2425	P	428.2616	214.6344	411.2350	206.1212			4
9	994.5568	497.7820	977.5302	489.2688	976.5462	488.7767	V	331.2088	166.1081	314.1823	157.5948			3
10	1051.5782	526.2928	1034.5517	517.7795	1033.5677	517.2875	G	232.1404	116.5738	215.1139	108.0606			2
11							R	175.1190	88.0631	158.0924	79.5498			1

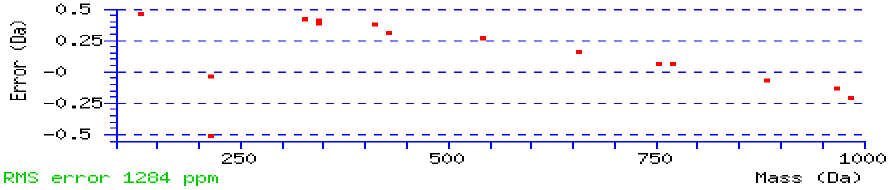

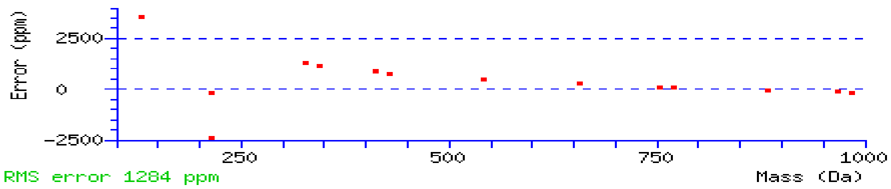

MS/MS Fragmentation of **AQIFANTVDNAR**
Found in **K1C18_HUMAN** in **SwissProt**, Keratin, type I cytoskeletal 18 OS=Homo sapiens GN=KRT18 PE=1 SV=2

Match to Query 283: 1318.956648 from(660.485600,2+) intensity(4652.9000) index(388)
Data file IS_111911_25b.txt

Click mouse within plot area to zoom in by factor of two about that point
Or, Plot from 100 to 1400 Da Full range
Label all possible matches ○ Label matches used for scoring ◉
Show Y-axis ☐

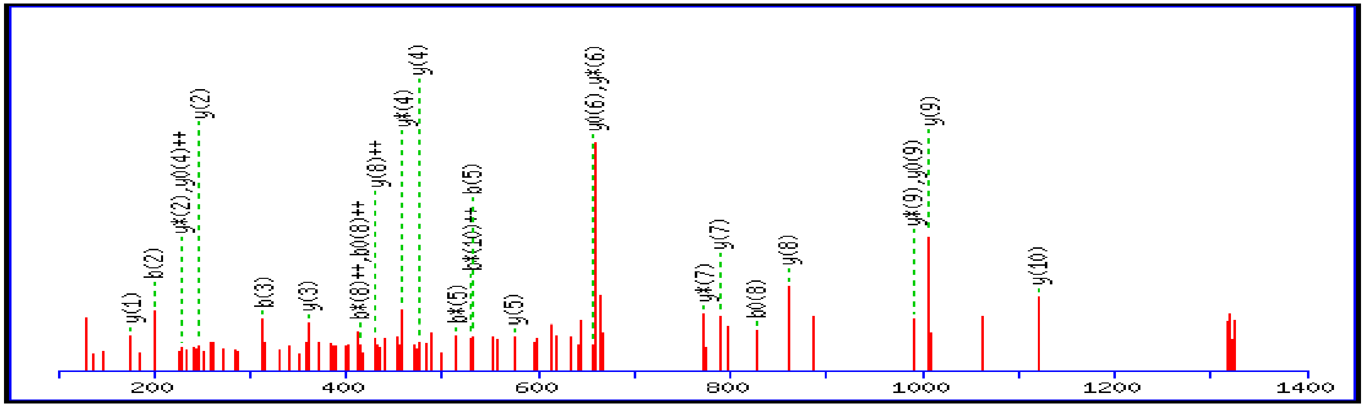

Monoisotopic mass of neutral peptide Mr(calc): 1318.6629
Fixed modifications: Carbamidomethyl (C) (apply to specified residues or termini only)
Ions Score: 43 Expect: 0.0097
Matches : 26/112 fragment ions using 56 most intense peaks (help)

#	b	b^{++}	b^{*}	b^{*++}	b^{0}	b^{0++}	Seq.	y	y^{++}	y^{*}	y^{*++}	y^{0}	y^{0++}	#
1	72.0444	36.5258					A							12
2	**200.1030**	100.5551	183.0764	92.0418			Q	1248.6331	624.8202	1231.6066	616.3069	1230.6226	615.8149	11
3	**313.1870**	157.0972	296.1605	148.5839			I	***1120.5745***	560.7909	1103.5480	552.2776	1102.5640	551.7856	10
4	460.2554	230.6314	443.2289	222.1181			F	***1007.4905***	504.2489	990.4639	495.7356	989.4799	495.2436	9
5	**531.2926**	266.1499	514.2660	257.6366			A	***860.4221***	430.7147	843.3955	422.2014	842.4115	421.7094	8
6	645.3355	323.1714	628.3089	314.6581			N	***789.3850***	395.1961	772.3584	386.6828	771.3744	386.1908	7
7	746.3832	373.6952	729.3566	365.1819	728.3726	364.6899	T	675.3420	338.1747	658.3155	329.6614	657.3315	329.1694	6
8	845.4516	423.2294	828.4250	414.7162	827.4410	414.2241	V	***574.2944***	287.6508	557.2678	279.1375	556.2838	278.6455	5
9	960.4785	480.7429	943.4520	472.2296	942.4680	471.7376	D	***475.2259***	238.1166	458.1994	229.6033	457.2154	229.1113	4
10	1074.5214	537.7644	1057.4949	529.2511	1056.5109	528.7591	N	***360.1990***	180.6031	343.1724	172.0899			3
11	1145.5586	573.2829	1128.5320	564.7696	1127.5480	564.2776	A	***246.1561***	123.5817	229.1295	115.0684			2
12							R	***175.1190***	88.0631	158.0924	79.5498			1

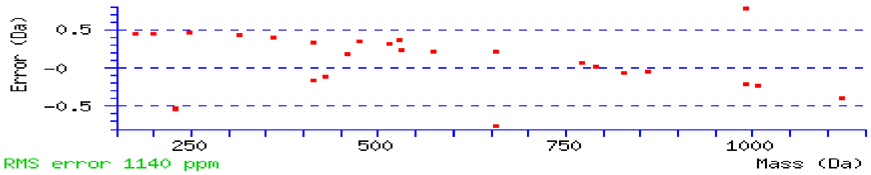

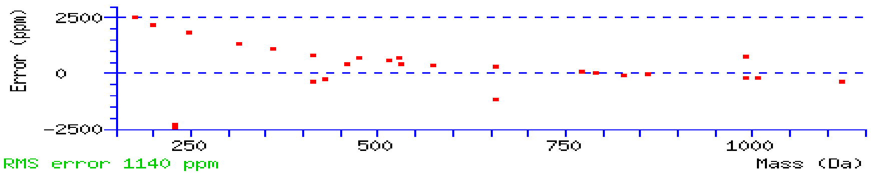

MS/MS Fragmentation of **TVPFCSTFAAFFTR**
Found in **TKT_HUMAN** in **SwissProt**, Transketolase OS=Homo sapiens GN=TKT PE=1 SV=3

Match to Query 407: 1650.727848 from(826.371200,2+) intensity(15321.9000) index(514)
Data file IS_111911_25b.txt

Click mouse within plot area to zoom in by factor of two about that point
Or, [Plot from] 100 to 1700 Da [Full range]
Label all possible matches ○ Label matches used for scoring ◉
Show Y-axis ☐

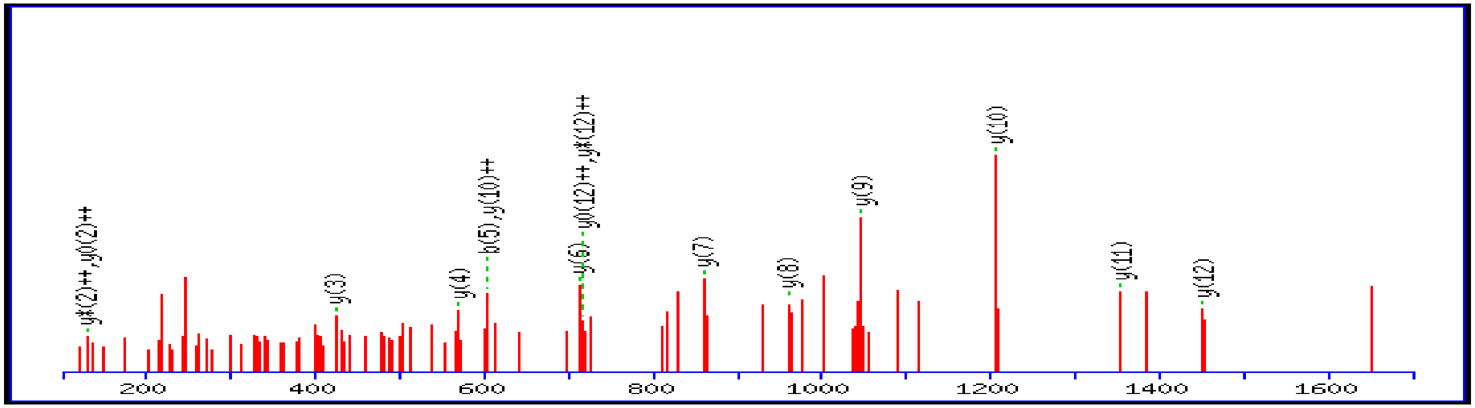


```
Monoisotopic mass of neutral peptide Mr(calc): 1650.7865
Fixed modifications: Carbamidomethyl (C) (apply to specified residues or termini only)
Ions Score: 38  Expect: 0.025
Matches : 15/128 fragment ions using 39 most intense peaks   (help)
```

#	b	b^{++}	b^{0}	b^{0++}	Seq.	y	y^{++}	y^{*}	y^{*++}	y^{0}	y^{0++}	#
1	102.0550	51.5311	84.0444	42.5258	T							14
2	201.1234	101.0653	183.1128	92.0600	V	1550.7460	775.8767	1533.7195	767.3634	1532.7355	766.8714	13
3	298.1761	149.5917	280.1656	140.5864	P	*1451.6776*	726.3425	1434.6511	717.8292	1433.6671	717.3372	12
4	445.2445	223.1259	427.2340	214.1206	F	*1354.6249*	677.8161	1337.5983	669.3028	1336.6143	668.8108	11
5	605.2752	303.1412	587.2646	294.1360	C	*1207.5565*	604.2819	1190.5299	595.7686	1189.5459	595.2766	10
6	692.3072	346.6573	674.2967	337.6520	S	*1047.5258*	524.2665	1030.4993	515.7533	1029.5152	515.2613	9
7	793.3549	397.1811	775.3443	388.1758	T	*960.4938*	480.7505	943.4672	472.2373	942.4832	471.7452	8
8	940.4233	470.7153	922.4128	461.7100	F	*859.4461*	430.2267	842.4196	421.7134	841.4355	421.2214	7
9	1011.4604	506.2339	993.4499	497.2286	A	*712.3777*	356.6925	695.3511	348.1792	694.3671	347.6872	6
10	1082.4975	541.7524	1064.4870	532.7471	A	641.3406	321.1739	624.3140	312.6607	623.3300	312.1686	5
11	1229.5660	615.2866	1211.5554	606.2813	F	*570.3035*	285.6554	553.2769	277.1421	552.2929	276.6501	4
12	1376.6344	688.8208	1358.6238	679.8155	F	*423.2350*	212.1212	406.2085	203.6079	405.2245	203.1159	3
13	1477.6821	739.3447	1459.6715	730.3394	T	276.1666	138.5870	259.1401	130.0737	258.1561	129.5817	2
14					R	175.1190	88.0631	158.0924	79.5498			1

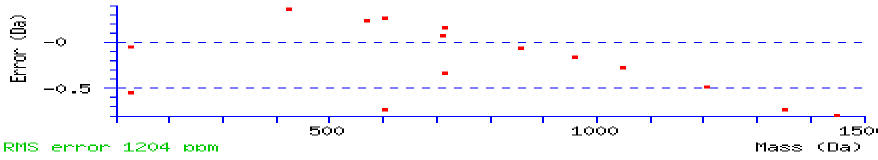

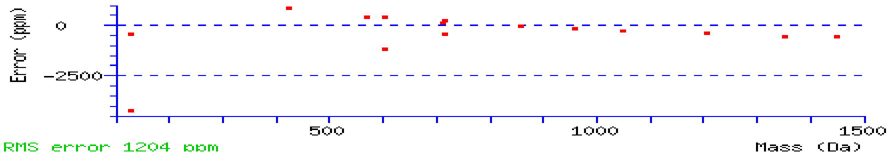

MS/MS Fragmentation of IVAFADAAVEPIDFPIAPVYAASMVLK
Found in THIL_HUMAN in SwissProt, Acetyl-CoA acetyltransferase, mitochondrial OS=Homo sapiens GN=ACAT1 PE=1 SV=1

Match to Query 544: 2832.970572 from(945.330800,3+) intensity(7684.3000) index(261)
Data file IS_111911_25b.txt

Click mouse within plot area to zoom in by factor of two about that point
Or, Plot from 200 to 1900 Da Full range
Label all possible matches ○ Label matches used for scoring ◉
Show Y-axis ☐

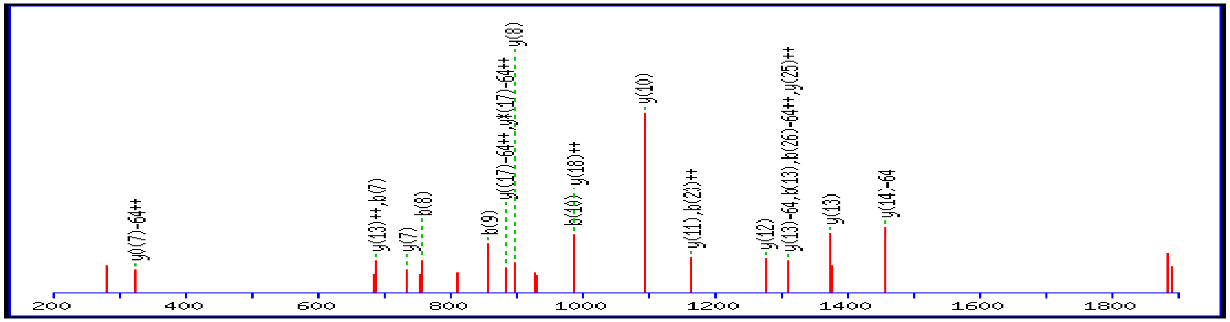

Monoisotopic mass of neutral peptide Mr(calc): 2833.4976
Fixed modifications: Carbamidomethyl (C) (apply to specified residues or termini only)
Variable modifications:
M24 : Oxidation (M), with neutral losses 0.0000(shown in table), 63.9983
Ions Score: 37 Expect: 0.012
Matches : 21/390 fragment ions using 22 most intense peaks (help)

#	b	b^{++}	b^0	b^{0++}	Seq.	y	y^{++}	y^*	y^{*++}	y^0	y^{0++}	#
1	114.0913	57.5493			I							27
2	213.1598	107.0835			V	2721.4208	1361.2140	2704.3943	1352.7008	2703.4102	1352.2088	26
3	284.1969	142.6021			A	2622.3524	1311.6798	2605.3258	1303.1666	2604.3418	1302.6746	25
4	431.2653	216.1363			F	2551.3153	1276.1613	2534.2887	1267.6480	2533.3047	1267.1560	24
5	502.3024	251.6548			A	2404.2469	1202.6271	2387.2203	1194.1138	2386.2363	1193.6218	23
6	617.3293	309.1683	599.3188	300.1630	D	2333.2097	1167.1085	2316.1832	1158.5952	2315.1992	1158.1032	22
7	688.3665	344.6869	670.3559	335.6816	A	2218.1828	1109.5950	2201.1563	1101.0818	2200.1722	1100.5898	21
8	759.4036	380.2054	741.3930	371.2001	A	2147.1457	1074.0765	2130.1191	1065.5632	2129.1351	1065.0712	20
9	858.4720	429.7396	840.4614	420.7343	V	2076.1086	1038.5579	2059.0820	1030.0447	2058.0980	1029.5526	19
10	987.5146	494.2609	969.5040	485.2556	E	1977.0402	989.0237	1960.0136	980.5104	1959.0296	980.0184	18
11	1084.5673	542.7873	1066.5568	533.7820	P	1847.9976	924.5024	1830.9710	915.9891	1829.9870	915.4971	17
12	1197.6514	599.3293	1179.6408	590.3241	I	1750.9448	875.9760	1733.9183	867.4628	1732.9342	866.9708	16
13	1312.6783	656.8428	1294.6678	647.8375	D	1637.8607	819.4340	1620.8342	810.9207	1619.8502	810.4287	15
14	1459.7468	730.3770	1441.7362	721.3717	F	1522.8338	761.9205	1505.8073	753.4073	1504.8232	752.9153	14
15	1556.7995	778.9034	1538.7890	769.8981	P	1375.7654	688.3863	1358.7388	679.8731	1357.7548	679.3810	13
16	1669.8836	835.4454	1651.8730	826.4401	I	1278.7126	639.8599	1261.6861	631.3467	1260.7021	630.8547	12
17	1740.9207	870.9640	1722.9101	861.9587	A	1165.6286	583.3179	1148.6020	574.8046	1147.6180	574.3126	11
18	1837.9735	919.4904	1819.9629	910.4851	P	1094.5914	547.7994	1077.5649	539.2861	1076.5809	538.7941	10
19	1937.0419	969.0246	1919.0313	960.0193	V	997.5387	499.2730	980.5121	490.7597	979.5281	490.2677	9
20	2100.1052	1050.5562	2082.0946	1041.5510	Y	898.4703	449.7388	881.4437	441.2255	880.4597	440.7335	8
21	2171.1423	1086.0748	2153.1318	1077.0695	A	735.4069	368.2071	718.3804	359.6938	717.3964	359.2018	7
22	2242.1794	1121.5934	2224.1689	1112.5881	A	664.3698	332.6886	647.3433	324.1753	646.3593	323.6833	6
23	2329.2115	1165.1094	2311.2009	1156.1041	S	593.3327	297.1700	576.3062	288.6567	575.3221	288.1647	5
24	2476.2469	1238.6271	2458.2363	1229.6218	M	506.3007	253.6540	489.2741	245.1407			4
25	2575.3153	1288.1613	2557.3047	1279.1560	V	359.2653	180.1363	342.2387	171.6230			3
26	2688.3993	1344.7033	2670.3888	1335.6980	L	260.1969	130.6021	243.1703	122.0888			2
27					K	147.1128	74.0600	130.0863	65.5468			1

MS/MS Fragmentation of QQNAPLPPWAHSMLR
Found in ZCC18_HUMAN in SwissProt, Putative zinc finger CCHC domain-containing protein 18 OS=Homo sapiens GN=ZCCHC18 PE=5 SV=1

Match to Query 424: 1760.638448 from(881.326500,2+) intensity(14801.6000) index(128)
Data file IS_111911_25b.txt

Click mouse within plot area to zoom in by factor of two about that point
Or, Plot from 100 to 1800 Da Full range
Label all possible matches ○ Label matches used for scoring ◉
Show Y-axis ☐

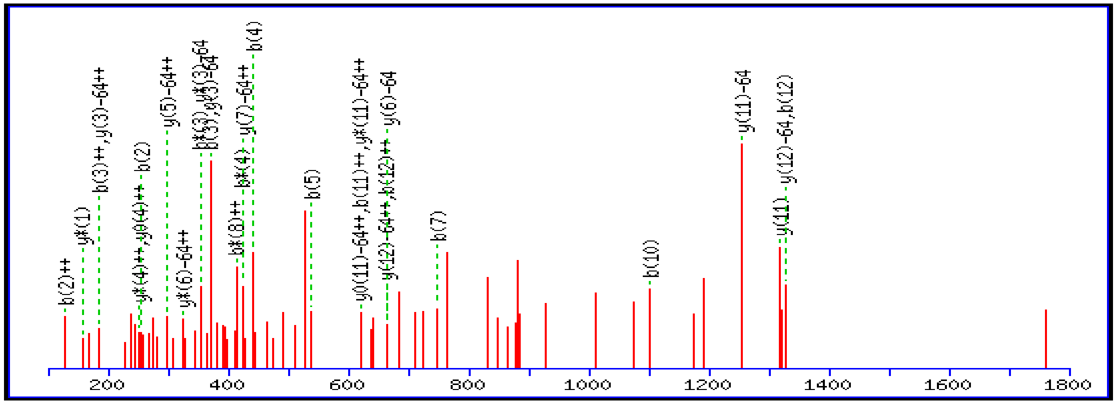


```
Monoisotopic mass of neutral peptide Mr(calc): 1760.8780
Fixed modifications: Carbamidomethyl (C) (apply to specified residues or termini only)
Variable modifications:
M13     : Oxidation (M), with neutral losses 63.9983(shown in table), 0.0000
Ions Score: 28  Expect: 0.21
Matches : 31/222 fragment ions using 54 most intense peaks   (help)
```

#	b	b^{++}	b^*	b^{*++}	b^0	b^{0++}	Seq.	y	y^{++}	y^*	y^{*++}	y^0	y^{0++}	#
1	129.0659	65.0366	112.0393	56.5233			Q							15
2	257.1244	129.0659	240.0979	120.5526			Q	1569.8285	785.4179	1552.8019	776.9046	1551.8179	776.4126	14
3	371.1674	186.0873	354.1408	177.5740			N	1441.7699	721.3886	1424.7433	712.8753	1423.7593	712.3833	13
4	442.2045	221.6059	425.1779	213.0926			A	1327.7270	664.3671	1310.7004	655.8538	1309.7164	655.3618	12
5	539.2572	270.1323	522.2307	261.6190			P	1256.6899	628.8486	1239.6633	620.3353	1238.6793	619.8433	11
6	652.3413	326.6743	635.3148	318.1610			L	1159.6371	580.3222	1142.6105	571.8089	1141.6265	571.3169	10
7	749.3941	375.2007	732.3675	366.6874			P	1046.5530	523.7802	1029.5265	515.2669	1028.5425	514.7749	9
8	846.4468	423.7271	829.4203	415.2138			P	949.5003	475.2538	932.4737	466.7405	931.4897	466.2485	8
9	1032.5261	516.7667	1015.4996	508.2534			W	852.4475	426.7274	835.4209	418.2141	834.4369	417.7221	7
10	1103.5633	552.2853	1086.5367	543.7720			A	666.3682	333.6877	649.3416	325.1745	648.3576	324.6824	6
11	1240.6222	620.8147	1223.5956	612.3014			H	595.3311	298.1692	578.3045	289.6559	577.3205	289.1639	5
12	1327.6542	664.3307	1310.6276	655.8175	1309.6436	655.3255	S	458.2722	229.6397	441.2456	221.1264	440.2616	220.6344	4
13	1410.6913	705.8493	1393.6648	697.3360	1392.6807	696.8440	M	371.2401	186.1237	354.2136	177.6104			3
14	1523.7754	762.3913	1506.7488	753.8781	1505.7648	753.3860	L	288.2030	144.6051	271.1765	136.0919			2
15							R	175.1190	88.0631	158.0924	79.5498			1

MS/MS Fragmentation of **TSFFQALGITTK**
Found in **RLA0L_HUMAN** in **SwissProt**, 60S acidic ribosomal protein P0-like OS=Homo sapiens GN=RPLP0P6 PE=5 SV=1

Match to Query 281: 1313.011848 from(657.513200,2+) intensity(3512.7000) index(199)
Data file IS_111911_25b.txt

Click mouse within plot area to zoom in by factor of two about that point
Or, Plot from 100 to 1400 Da Full range
Label all possible matches ○ Label matches used for scoring ◉
Show Y-axis ☐

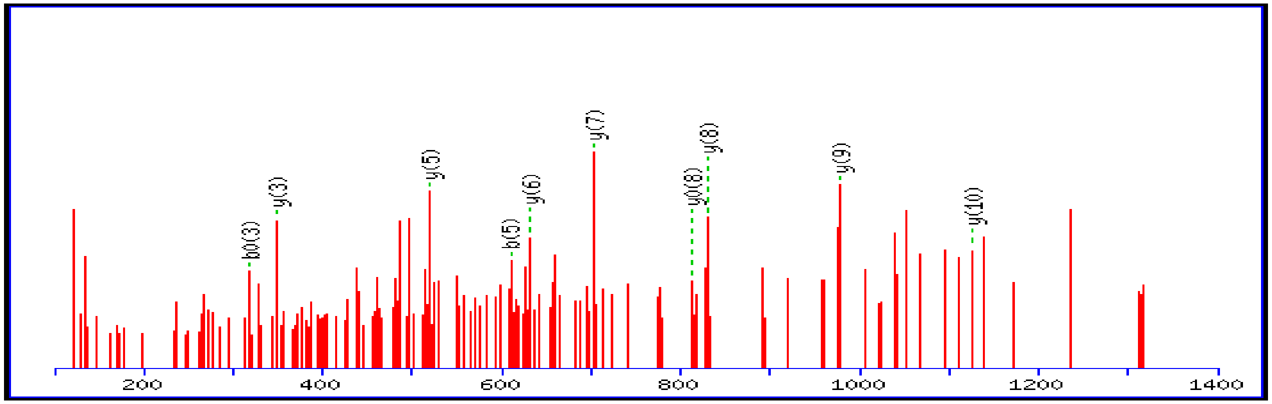

Monoisotopic mass of neutral peptide Mr(calc): 1312.7027
Fixed modifications: Carbamidomethyl (C) (apply to specified residues or termini only)
Ions Score: 28 Expect: 0.25
Matches : 10/122 fragment ions using 24 most intense peaks (help)

#	b	b^{++}	b^{*}	b^{*++}	b^{0}	b^{0++}	Seq.	y	y^{++}	y^{*}	y^{*++}	y^{0}	y^{0++}	#
1	102.0550	51.5311			84.0444	42.5258	T							12
2	189.0870	95.0471			171.0764	86.0418	S	1212.6623	606.8348	1195.6358	598.3215	1194.6517	597.8295	11
3	336.1554	168.5813			318.1448	159.5761	F	1125.6303	563.3188	1108.6037	554.8055	1107.6197	554.3135	10
4	483.2238	242.1155			465.2132	233.1103	F	978.5619	489.7846	961.5353	481.2713	960.5513	480.7793	9
5	611.2824	306.1448	594.2558	297.6316	593.2718	297.1395	Q	831.4934	416.2504	814.4669	407.7371	813.4829	407.2451	8
6	682.3195	341.6634	665.2930	333.1501	664.3089	332.6581	A	703.4349	352.2211	686.4083	343.7078	685.4243	343.2158	7
7	795.4036	398.2054	778.3770	389.6921	777.3930	389.2001	L	632.3978	316.7025	615.3712	308.1892	614.3872	307.6972	6
8	852.4250	426.7162	835.3985	418.2029	834.4145	417.7109	G	519.3137	260.1605	502.2871	251.6472	501.3031	251.1552	5
9	965.5091	483.2582	948.4825	474.7449	947.4985	474.2529	I	462.2922	231.6498	445.2657	223.1365	444.2817	222.6445	4
10	1066.5568	533.7820	1049.5302	525.2688	1048.5462	524.7767	T	349.2082	175.1077	332.1816	166.5944	331.1976	166.1024	3
11	1167.6045	584.3059	1150.5779	575.7926	1149.5939	575.3006	T	248.1605	124.5839	231.1339	116.0706	230.1499	115.5786	2
12							K	147.1128	74.0600	130.0863	65.5468			1

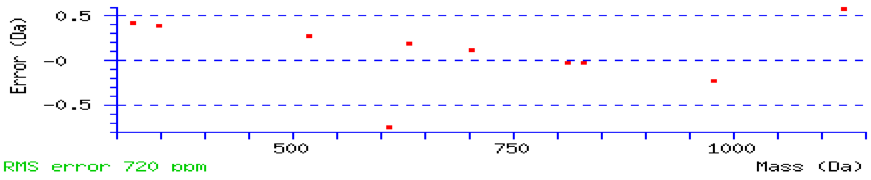

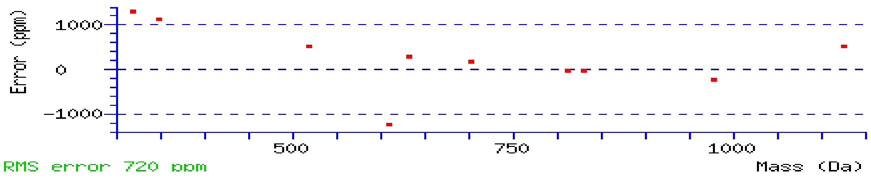

MS/MS Fragmentation of GELLEAIKR
Found in SODM_HUMAN in SwissProt, Superoxide dismutase [Mn], mitochondrial OS=Homo sapiens GN=SOD2 PE=1 SV=2

Match to Query 300: 1028.182848 from(515.098700,2+) intensity(949.8000) index(26)
Data file IS_111911_26.pkl

Click mouse within plot area to zoom in by factor of two about that point
Or, Plot from 100 to 850 Da Full range
Label all possible matches ○ Label matches used for scoring ◉
Show Y-axis ☐

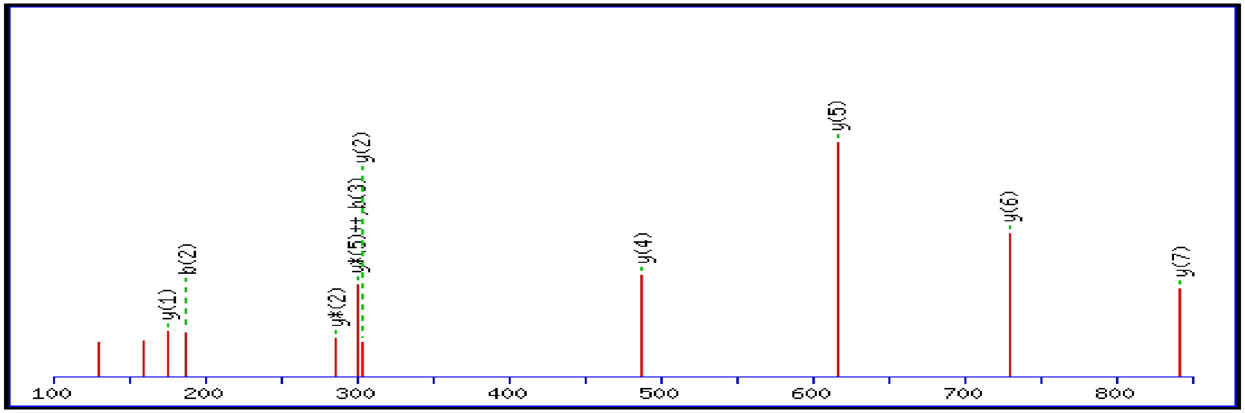

Monoisotopic mass of neutral peptide Mr(calc): 1027.6026
Fixed modifications: Carbamidomethyl (C) (apply to specified residues or termini only)
Ions Score: 56 Expect: 0.00045
Matches : 10/72 fragment ions using 11 most intense peaks (help)

#	b	b^{++}	b^{*}	b^{*++}	b^{0}	b^{0++}	Seq.	y	y^{++}	y^{*}	y^{*++}	y^{0}	y^{0++}	#
1	58.0287	29.5180					G							9
2	187.0713	94.0393			169.0608	85.0340	E	971.5884	486.2978	954.5619	477.7846	953.5778	477.2926	8
3	300.1554	150.5813			282.1448	141.5761	L	842.5458	421.7765	825.5193	413.2633	824.5352	412.7713	7
4	413.2395	207.1234			395.2289	198.1181	L	729.4618	365.2345	712.4352	356.7212	711.4512	356.2292	6
5	542.2821	271.6447			524.2715	262.6394	E	616.3777	308.6925	599.3511	300.1792	598.3671	299.6872	5
6	613.3192	307.1632			595.3086	298.1579	A	487.3351	244.1712	470.3085	235.6579			4
7	726.4032	363.7053			708.3927	354.7000	I	416.2980	208.6526	399.2714	200.1394			3
8	854.4982	427.7527	837.4716	419.2395	836.4876	418.7475	K	303.2139	152.1106	286.1874	143.5973			2
9							R	175.1190	88.0631	158.0924	79.5498			1

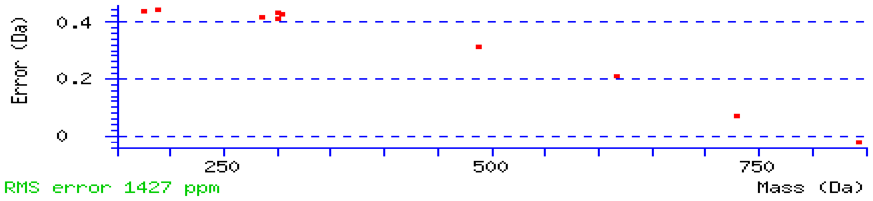

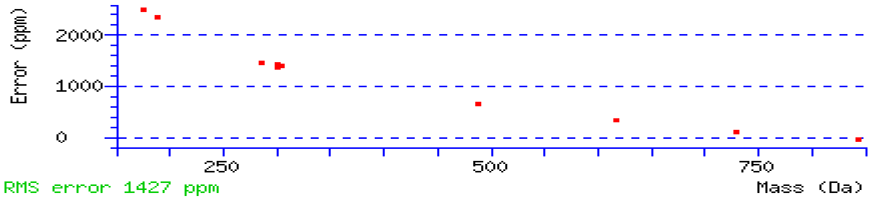

MS/MS Fragmentation of GVDEVTIVNILTNR
Found in ANXA2_HUMAN in SwissProt, Annexin A2 OS=Homo sapiens GN=ANXA2 PE=1 SV=2

Match to Query 441: 1541.916248 from(771.965400,2+) intensity(105.4000) index(57)
Data file IS_111911_26.pkl

Click mouse within plot area to zoom in by factor of two about that point
Or, Plot from 200 to 1050 Da Full range
Label all possible matches ○ Label matches used for scoring ◉
Show Y-axis ☐

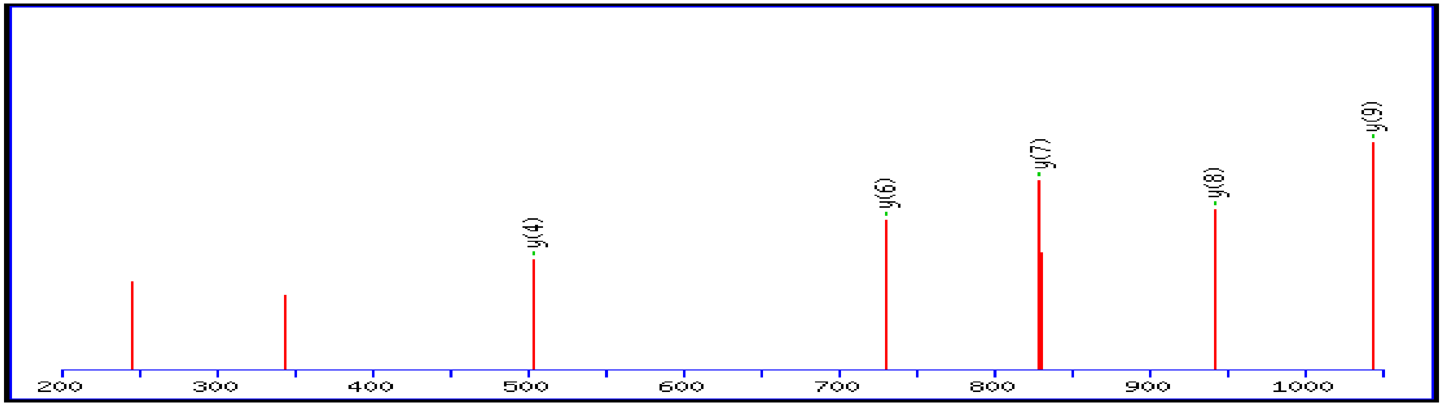

Monoisotopic mass of neutral peptide Mr(calc): 1541.8413
Fixed modifications: Carbamidomethyl (C) (apply to specified residues or termini only)
Ions Score: 42 Expect: 0.011
Matches : 5/132 fragment ions using 6 most intense peaks (help)

#	b	b++	b*	b*++	b0	b0++	Seq.	y	y++	y*	y*++	y0	y0++	#
1	58.0287	29.5180					G							14
2	157.0972	79.0522					V	1485.8271	743.4172	1468.8006	734.9039	1467.8166	734.4119	13
3	272.1241	136.5657			254.1135	127.5604	D	1386.7587	693.8830	1369.7322	685.3697	1368.7482	684.8777	12
4	401.1667	201.0870			383.1561	192.0817	E	1271.7318	636.3695	1254.7052	627.8563	1253.7212	627.3642	11
5	500.2351	250.6212			482.2245	241.6159	V	1142.6892	571.8482	1125.6626	563.3350	1124.6786	562.8429	10
6	601.2828	301.1450			583.2722	292.1397	T	1043.6208	522.3140	1026.5942	513.8007	1025.6102	513.3087	9
7	714.3668	357.6871			696.3563	348.6818	I	942.5731	471.7902	925.5465	463.2769	924.5625	462.7849	8
8	813.4353	407.2213			795.4247	398.2160	V	829.4890	415.2482	812.4625	406.7349	811.4785	406.2429	7
9	927.4782	464.2427	910.4516	455.7295	909.4676	455.2374	N	730.4206	365.7139	713.3941	357.2007	712.4100	356.7087	6
10	1040.5623	520.7848	1023.5357	512.2715	1022.5517	511.7795	I	616.3777	308.6925	599.3511	300.1792	598.3671	299.6872	5
11	1153.6463	577.3268	1136.6198	568.8135	1135.6358	568.3215	L	503.2936	252.1504	486.2671	243.6372	485.2831	243.1452	4
12	1254.6940	627.8506	1237.6674	619.3374	1236.6834	618.8454	T	390.2096	195.6084	373.1830	187.0951	372.1990	186.6031	3
13	1368.7369	684.8721	1351.7104	676.3588	1350.7264	675.8668	N	289.1619	145.0846	272.1353	136.5713			2
14							R	175.1190	88.0631	158.0924	79.5498			1

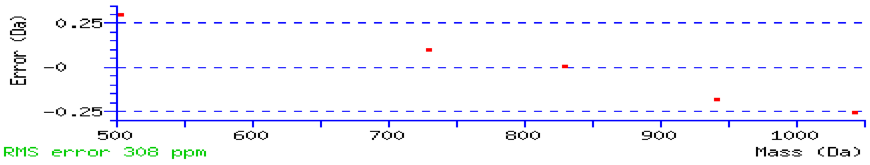

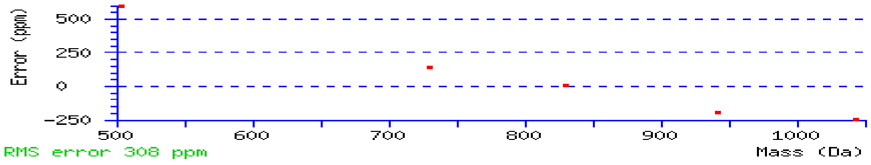

MS/MS Fragmentation of **DYGVLLEGSGLALR**
Found in **PRDX3_HUMAN** in **SwissProt**, Thioredoxin-dependent peroxide reductase, mitochondrial OS=Homo sapiens GN=PRDX3 PE=1 SV=3

Match to Query 430: 1461.948448 from(731.981500,2+) intensity(427.9000) index(53)
Data file IS_111911_26.pkl

Click mouse within plot area to zoom in by factor of two about that point
Or, Plot from 250 to 1050 Da Full range
Label all possible matches ○ Label matches used for scoring ◉
Show Y-axis ☐

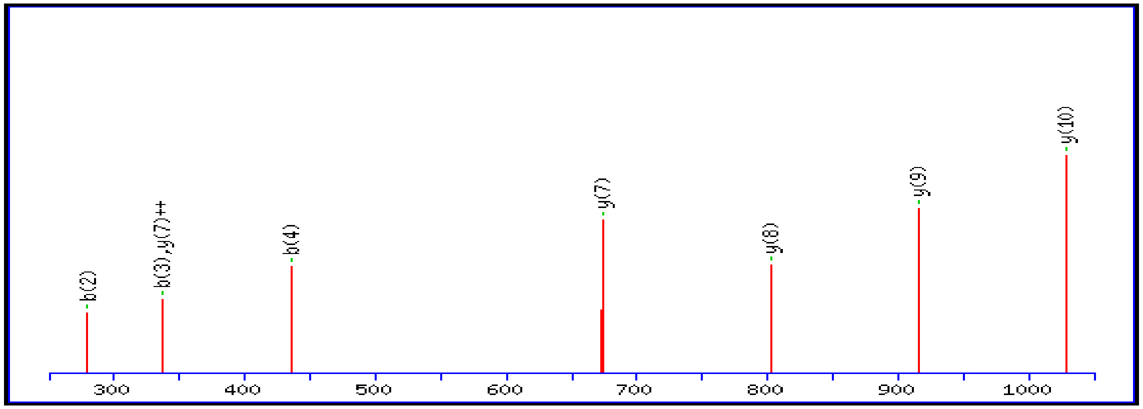

Monoisotopic mass of neutral peptide Mr(calc): 1461.7827
Fixed modifications: Carbamidomethyl (C) (apply to specified residues or termini only)
Ions Score: 39 Expect: 0.022
Matches : 8/120 fragment ions using 8 most intense peaks (help)

#	b	b^{++}	b^{0}	b^{0++}	Seq.	y	y^{++}	y^{*}	y^{*++}	y^{0}	y^{0++}	#
1	116.0342	58.5207	98.0237	49.5155	D							14
2	*279.0975*	140.0524	261.0870	131.0471	Y	1347.7631	674.3852	1330.7365	665.8719	1329.7525	665.3799	13
3	*336.1190*	168.5631	318.1084	159.5579	G	1184.6997	592.8535	1167.6732	584.3402	1166.6892	583.8482	12
4	*435.1874*	218.0974	417.1769	209.0921	V	1127.6783	564.3428	1110.6517	555.8295	1109.6677	555.3375	11
5	548.2715	274.6394	530.2609	265.6341	L	*1028.6099*	514.8086	1011.5833	506.2953	1010.5993	505.8033	10
6	661.3556	331.1814	643.3450	322.1761	L	*915.5258*	458.2665	898.4993	449.7533	897.5152	449.2613	9
7	790.3981	395.7027	772.3876	386.6974	E	*802.4417*	401.7245	785.4152	393.2112	784.4312	392.7192	8
8	847.4196	424.2134	829.4090	415.2082	G	*673.3991*	337.2032	656.3726	328.6899	655.3886	328.1979	7
9	934.4516	467.7295	916.4411	458.7242	S	616.3777	308.6925	599.3511	300.1792	598.3671	299.6872	6
10	991.4731	496.2402	973.4625	487.2349	G	529.3457	265.1765	512.3191	256.6632			5
11	1104.5572	552.7822	1086.5466	543.7769	L	472.3242	236.6657	455.2976	228.1525			4
12	1175.5943	588.3008	1157.5837	579.2955	A	359.2401	180.1237	342.2136	171.6104			3
13	1288.6783	644.8428	1270.6678	635.8375	L	288.2030	144.6051	271.1765	136.0919			2
14					R	175.1190	88.0631	158.0924	79.5498			1

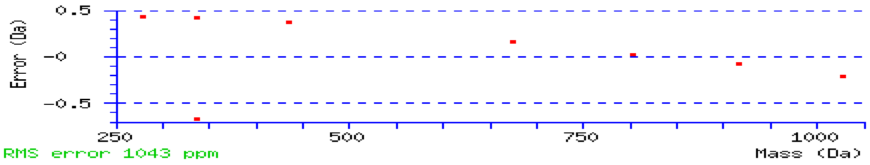

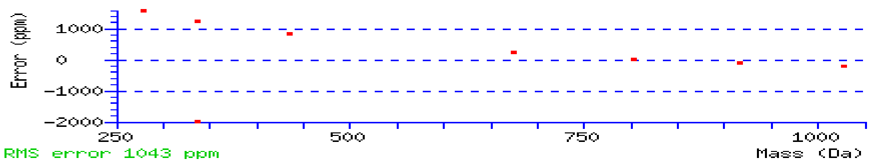

Supplement: Figure S2 — MS/MS spectra of proteins identified by one peptide (I). The proteins were found in PM of (ND) cells, in experiment 1 (Mascot scores higher than 25). (PDF) [file pone.0071859.s002.pdf]
