# Supplementary material for: Comparative Proteomics Reveals Novel Components at the Plasma Membrane of Differentiated HepaRG Cells and Different Distribution in Hepatocyte- and Biliary-Like Cells
Source: PLoS One. 2013 Aug 20;8(8):e71859. doi: 10.1371/journal.pone.0071859 (PMC3748114; doi:10.1371/journal.pone.0071859)
Supplement: Figure S3 — MS/MS spectra of proteins identified by one peptide (II). The proteins were found in PM of (D) cells, in experiment 1 (Mascot scores higher than 25). (PDF) [file pone.0071859.s003.pdf]

MS/MS Fragmentation of **EKPYFPIPEEYTFIQNVPLEDR**
Found in **HNRPU_HUMAN** in **SwissProt**, Heterogeneous nuclear ribonucleoprotein U OS=Homo sapiens GN=HNRNPU PE=1 SV=6

Match to Query 798: 2723.085072 from(908.702300,3+) intensity(3915.0000) index(86)
Data file IS_111911_21.pkl

Click mouse within plot area to zoom in by factor of two about that point
Or, Plot from 0 to 2800 Da Full range
Label all possible matches ○ Label matches used for scoring ◉
Show Y-axis ☐

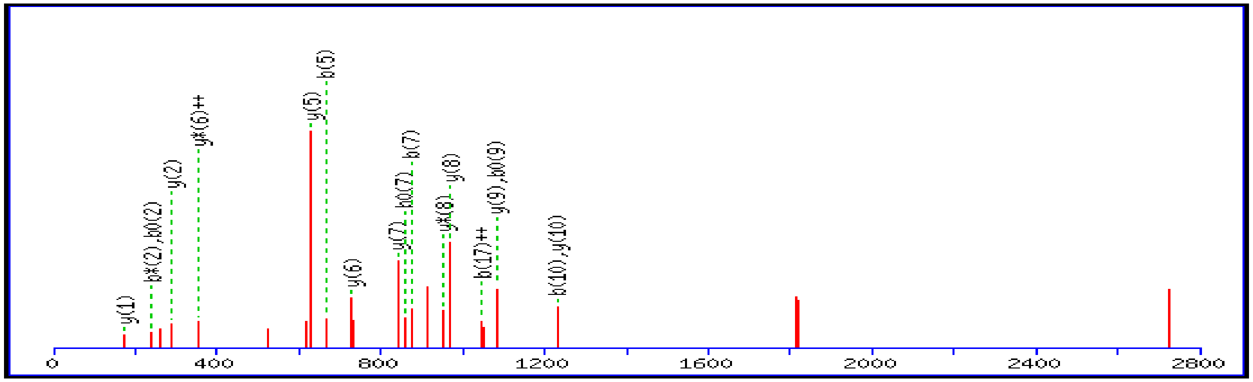


```
Monoisotopic mass of neutral peptide Mr(calc): 2723.3483
Fixed modifications: Carbamidomethyl (C) (apply to specified residues or termini only)
Ions Score: 41  Expect: 0.0057
Matches : 18/248 fragment ions using 25 most intense peaks   (help)
```

#	b	b^{++}	b^{*}	b^{*++}	b^{0}	b^{0++}	Seq.	y	y^{++}	y^{*}	y^{*++}	y^{0}	y^{0++}	#
1	130.0499	65.5286			112.0393	56.5233	E							22
2	258.1448	129.5761	241.1183	121.0628	240.1343	120.5708	K	2595.3130	1298.1601	2578.2864	1289.6468	2577.3024	1289.1548	21
3	355.1976	178.1024	338.1710	169.5892	337.1870	169.0972	P	2467.2180	1234.1126	2450.1915	1225.5994	2449.2074	1225.1074	20
4	518.2609	259.6341	501.2344	251.1208	500.2504	250.6288	Y	2370.1652	1185.5863	2353.1387	1177.0730	2352.1547	1176.5810	19
5	**665.3293**	333.1683	648.3028	324.6550	647.3188	324.1630	F	2207.1019	1104.0546	2190.0754	1095.5413	2189.0913	1095.0493	18
6	762.3821	381.6947	745.3556	373.1814	744.3715	372.6894	P	2060.0335	1030.5204	2043.0069	1022.0071	2042.0229	1021.5151	17
7	**875.4662**	438.2367	858.4396	429.7234	857.4556	429.2314	I	1962.9807	981.9940	1945.9542	973.4807	1944.9702	972.9887	16
8	972.5189	486.7631	955.4924	478.2498	954.5084	477.7578	P	1849.8967	925.4520	1832.8701	916.9387	1831.8861	916.4467	15
9	1101.5615	551.2844	1084.5350	542.7711	1083.5510	542.2791	E	1752.8439	876.9256	1735.8174	868.4123	1734.8333	867.9203	14
10	**1230.6041**	615.8057	1213.5776	607.2924	1212.5936	606.8004	E	1623.8013	812.4043	1606.7748	803.8910	1605.7907	803.3990	13
11	1393.6674	697.3374	1376.6409	688.8241	1375.6569	688.3321	Y	1494.7587	747.8830	1477.7322	739.3697	1476.7482	738.8777	12
12	1494.7151	747.8612	1477.6886	739.3479	1476.7046	738.8559	T	1331.6954	666.3513	1314.6688	657.8381	1313.6848	657.3461	11
13	1641.7835	821.3954	1624.7570	812.8821	1623.7730	812.3901	F	*1230.6477*	615.8275	1213.6212	607.3142	1212.6371	606.8222	10
14	1754.8676	877.9374	1737.8411	869.4242	1736.8570	868.9322	I	*1083.5793*	542.2933	1066.5527	533.7800	1065.5687	533.2880	9
15	1882.9262	941.9667	1865.8996	933.4535	1864.9156	932.9614	Q	*970.4952*	485.7513	953.4687	477.2380	952.4847	476.7460	8
16	1996.9691	998.9882	1979.9426	990.4749	1978.9585	989.9829	N	*842.4367*	421.7220	825.4101	413.2087	824.4261	412.7167	7
17	2096.0375	1048.5224	2079.0110	1040.0091	2078.0270	1039.5171	V	*728.3937*	364.7005	711.3672	356.1872	710.3832	355.6952	6
18	2193.0903	1097.0488	2176.0637	1088.5355	2175.0797	1088.0435	P	*629.3253*	315.1663	612.2988	306.6530	611.3148	306.1610	5
19	2306.1743	1153.5908	2289.1478	1145.0775	2288.1638	1144.5855	L	532.2726	266.6399	515.2460	258.1266	514.2620	257.6346	4
20	2435.2169	1218.1121	2418.1904	1209.5988	2417.2064	1209.1068	E	419.1885	210.0979	402.1619	201.5846	401.1779	201.0926	3
21	2550.2439	1275.6256	2533.2173	1267.1123	2532.2333	1266.6203	D	*290.1459*	145.5766	273.1193	137.0633	272.1353	136.5713	2
22							R	*175.1190*	88.0631	158.0924	79.5498			1

MS/MS Fragmentation of **ALLVEPVINSYLLAER**
Found in **TGM2_HUMAN** in **SwissProt**, Protein-glutamine gamma-glutamyltransferase 2 OS=Homo sapiens GN=TGM2 PE=1 SV=2

Match to Query 678: 1798.880048 from(900.447300,2+) intensity(5259.5000) index(411)
Data file IS_111911_21.pkl

Click mouse within plot area to zoom in by factor of two about that point
Or, Plot from 100 to 1500 Da Full range
Label all possible matches ○ Label matches used for scoring ◉
Show Y-axis ☐

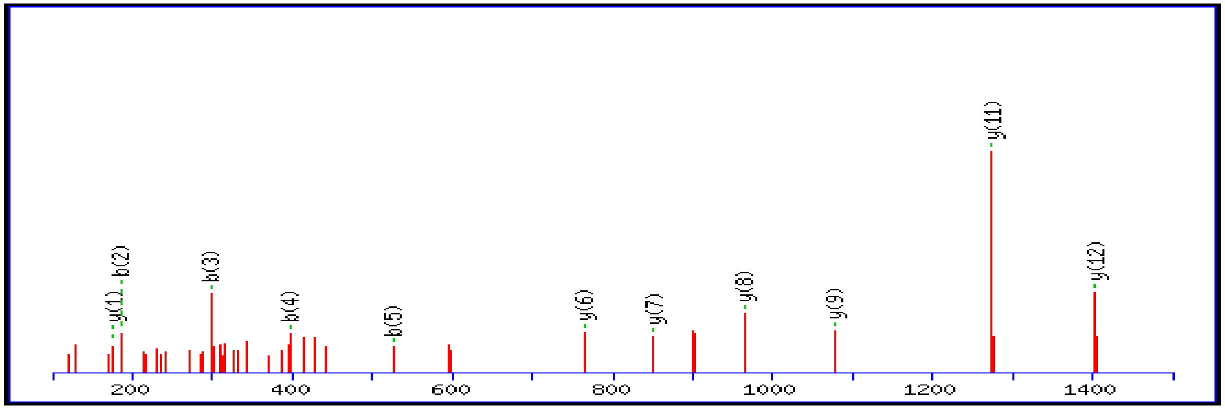


```
Monoisotopic mass of neutral peptide Mr(calc): 1799.0192
Fixed modifications: Carbamidomethyl (C) (apply to specified residues or termini only)
Ions Score: 36  Expect: 0.034
Matches : 11/154 fragment ions using 25 most intense peaks   (help)
```

#	b	b^{++}	b^*	b^{*++}	b^0	b^{0++}	Seq.	y	y^{++}	y^*	y^{*++}	y^0	y^{0++}	#
1	72.0444	36.5258					A							16
2	*185.1285*	93.0679					L	1728.9894	864.9984	1711.9629	856.4851	1710.9789	855.9931	15
3	*298.2125*	149.6099					L	1615.9054	808.4563	1598.8788	799.9431	1597.8948	799.4510	14
4	*397.2809*	199.1441					V	1502.8213	751.9143	1485.7948	743.4010	1484.8108	742.9090	13
5	*526.3235*	263.6654			508.3130	254.6601	E	*1403.7529*	702.3801	1386.7264	693.8668	1385.7423	693.3748	12
6	623.3763	312.1918			605.3657	303.1865	P	*1274.7103*	637.8588	1257.6838	629.3455	1256.6997	628.8535	11
7	722.4447	361.7260			704.4341	352.7207	V	1177.6575	589.3324	1160.6310	580.8191	1159.6470	580.3271	10
8	835.5288	418.2680			817.5182	409.2627	I	*1078.5891*	539.7982	1061.5626	531.2849	1060.5786	530.7929	9
9	949.5717	475.2895	932.5451	466.7762	931.5611	466.2842	N	*965.5051*	483.2562	948.4785	474.7429	947.4945	474.2509	8
10	1036.6037	518.8055	1019.5772	510.2922	1018.5932	509.8002	S	*851.4621*	426.2347	834.4356	417.7214	833.4516	417.2294	7
11	1199.6671	600.3372	1182.6405	591.8239	1181.6565	591.3319	Y	*764.4301*	382.7187	747.4036	374.2054	746.4196	373.7134	6
12	1312.7511	656.8792	1295.7246	648.3659	1294.7405	647.8739	L	601.3668	301.1870	584.3402	292.6738	583.3562	292.1817	5
13	1425.8352	713.4212	1408.8086	704.9080	1407.8246	704.4159	L	488.2827	244.6450	471.2562	236.1317	470.2722	235.6397	4
14	1496.8723	748.9398	1479.8457	740.4265	1478.8617	739.9345	A	375.1987	188.1030	358.1721	179.5897	357.1881	179.0977	3
15	1625.9149	813.4611	1608.8883	804.9478	1607.9043	804.4558	E	304.1615	152.5844	287.1350	144.0711	286.1510	143.5791	2
16							R	*175.1190*	88.0631	158.0924	79.5498			1

MS/MS Fragmentation of **EDSDMLAAGGK**
Found in **RTN4_HUMAN** in **SwissProt**, Reticulon-4 OS=Homo sapiens GN=RTN4 PE=1 SV=2

Match to Query 314: 1093.090648 from(547.552600,2+) intensity(980.3000) index(317)
Data file IS_111911_21.pkl

Click mouse within plot area to zoom in by factor of two about that point
Or, Plot from 100 to 850 Da Full range
Label all possible matches ○ Label matches used for scoring ◉
Show Y-axis ☐

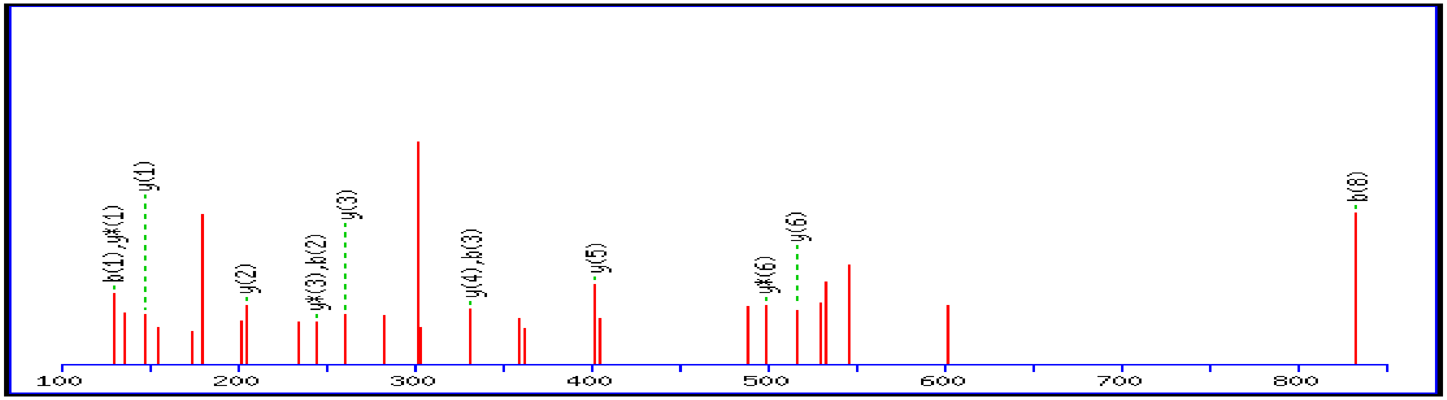

Monoisotopic mass of neutral peptide Mr(calc): 1092.4757
Fixed modifications: Carbamidomethyl (C) (apply to specified residues or termini only)
Ions Score: 35 Expect: 0.05
Matches : 13/86 fragment ions using 23 most intense peaks (help)

#	b	b^{++}	b^{0}	b^{0++}	Seq.	y	y^{++}	y^{*}	y^{*++}	y^{0}	y^{0++}	#
1	*130.0499*	65.5286	112.0393	56.5233	E							11
2	*245.0768*	123.0420	227.0662	114.0368	D	964.4404	482.7238	947.4139	474.2106	946.4299	473.7186	10
3	*332.1088*	166.5581	314.0983	157.5528	S	849.4135	425.2104	832.3869	416.6971	831.4029	416.2051	9
4	447.1358	224.0715	429.1252	215.0662	D	762.3815	381.6944	745.3549	373.1811	744.3709	372.6891	8
5	578.1763	289.5918	560.1657	280.5865	M	647.3545	324.1809	630.3280	315.6676			7
6	691.2603	346.1338	673.2498	337.1285	L	*516.3140*	258.6607	499.2875	250.1474			6
7	762.2974	381.6524	744.2869	372.6471	A	*403.2300*	202.1186	386.2034	193.6053			5
8	*833.3346*	417.1709	815.3240	408.1656	A	*332.1928*	166.6001	315.1663	158.0868			4
9	890.3560	445.6817	872.3455	436.6764	G	*261.1557*	131.0815	244.1292	122.5682			3
10	947.3775	474.1924	929.3669	465.1871	G	*204.1343*	102.5708	187.1077	94.0575			2
11					K	*147.1128*	74.0600	130.0863	65.5468			1

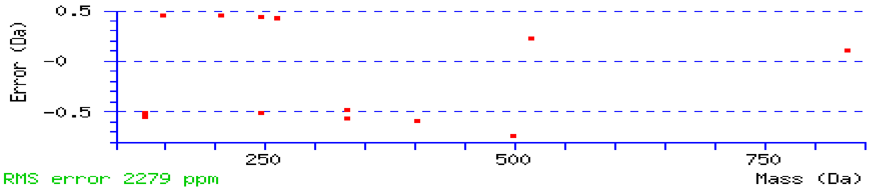

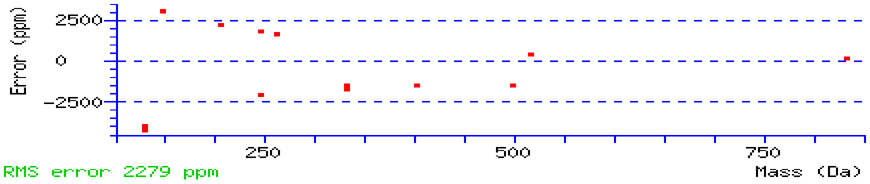

MS/MS Fragmentation of **IPAFLNVVDIAGLVK**
Found in **OLA1_HUMAN** in **SwissProt**, Obg-like ATPase 1 OS=Homo sapiens GN=OLA1 PE=1 SV=2

Match to Query 430: 1568.044448 from(785.029500,2+) intensity(532.4000) index(141)
Data file IS_111911_22.pkl

Click mouse within plot area to zoom in by factor of two about that point
Or, Plot from 100 to 1600 Da Full range
Label all possible matches ○ Label matches used for scoring ◉
Show Y-axis ☐

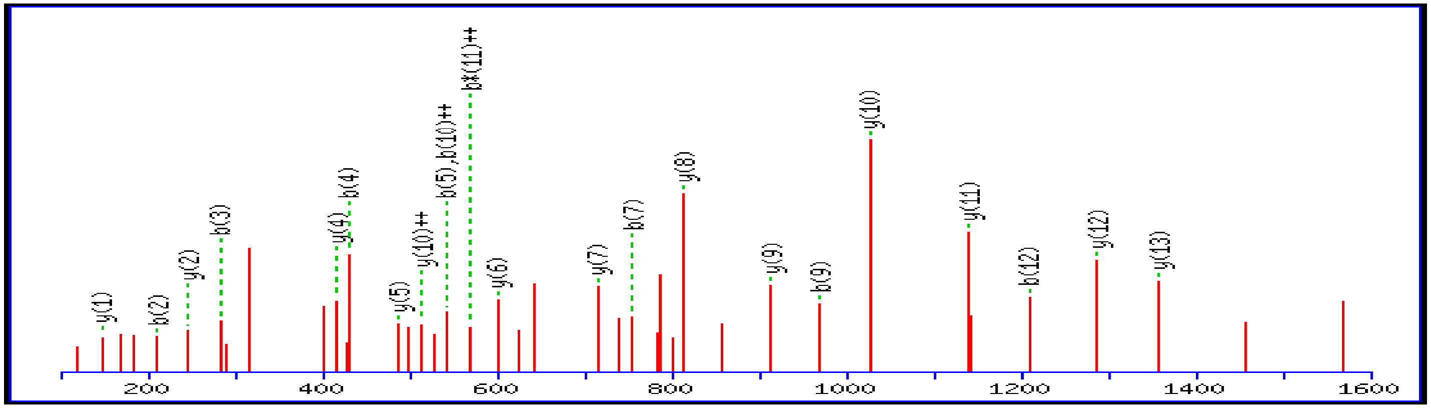

Monoisotopic mass of neutral peptide Mr(calc): 1567.9338
Fixed modifications: Carbamidomethyl (C) (apply to specified residues or termini only)
Ions Score: 89 Expect: 1.9e-07
Matches : 22/130 fragment ions using 37 most intense peaks (help)

#	b	b++	b*	b*++	b0	b0++	Seq.	y	y++	y*	y*++	y0	y0++	#
1	114.0913	57.5493					I							15
2	**211.1441**	106.0757					P	1455.8570	728.4321	1438.8304	719.9189	1437.8464	719.4268	14
3	**282.1812**	141.5942					A	**1358.8042**	679.9057	1341.7777	671.3925	1340.7936	670.9005	13
4	**429.2496**	215.1285					F	**1287.7671**	644.3872	1270.7406	635.8739	1269.7565	635.3819	12
5	**542.3337**	271.6705					L	**1140.6987**	570.8530	1123.6721	562.3397	1122.6881	561.8477	11
6	656.3766	328.6919	639.3501	320.1787			N	**1027.6146**	**514.3109**	1010.5881	505.7977	1009.6041	505.3057	10
7	**755.4450**	378.2262	738.4185	369.7129			V	**913.5717**	457.2895	896.5451	448.7762	895.5611	448.2842	9
8	854.5135	427.7604	837.4869	419.2471			V	**814.5033**	407.7553	797.4767	399.2420	796.4927	398.7500	8
9	**969.5404**	485.2738	952.5138	476.7606	951.5298	476.2686	D	**715.4349**	358.2211	698.4083	349.7078	697.4243	349.2158	7
10	1082.6245	**541.8159**	1065.5979	533.3026	1064.6139	532.8106	I	**600.4079**	300.7076	583.3814	292.1943			6
11	1153.6616	577.3344	1136.6350	**568.8211**	1135.6510	568.3291	A	**487.3239**	244.1656	470.2973	235.6523			5
12	**1210.6830**	605.8452	1193.6565	597.3319	1192.6725	596.8399	G	**416.2867**	208.6470	399.2602	200.1337			4
13	1323.7671	662.3872	1306.7406	653.8739	1305.7565	653.3819	L	359.2653	180.1363	342.2387	171.6230			3
14	1422.8355	711.9214	1405.8090	703.4081	1404.8249	702.9161	V	**246.1812**	123.5942	229.1547	115.0810			2
15							K	**147.1128**	74.0600	130.0863	65.5468			1

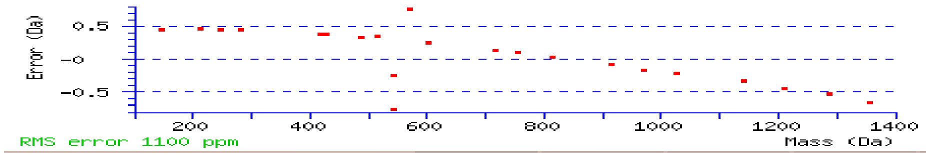

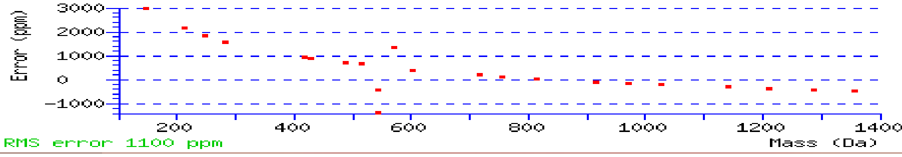

MS/MS Fragmentation of **GLGTDEESILTLLLTSR**
Found in **ANXA5_HUMAN** in **SwissProt**, Annexin A5 OS=Homo sapiens GN=ANXA5 PE=1 SV=2

Match to Query 498: 1703.840048 from(852.927300,2+) intensity(1958.0000) index(288)
Data file IS_111911_22.pkl

Click mouse within plot area to zoom in by factor of two about that point
Or, [Plot from] [150] to [1150] Da [Full range]
Label all possible matches ○ Label matches used for scoring ◉
Show Y-axis ☐

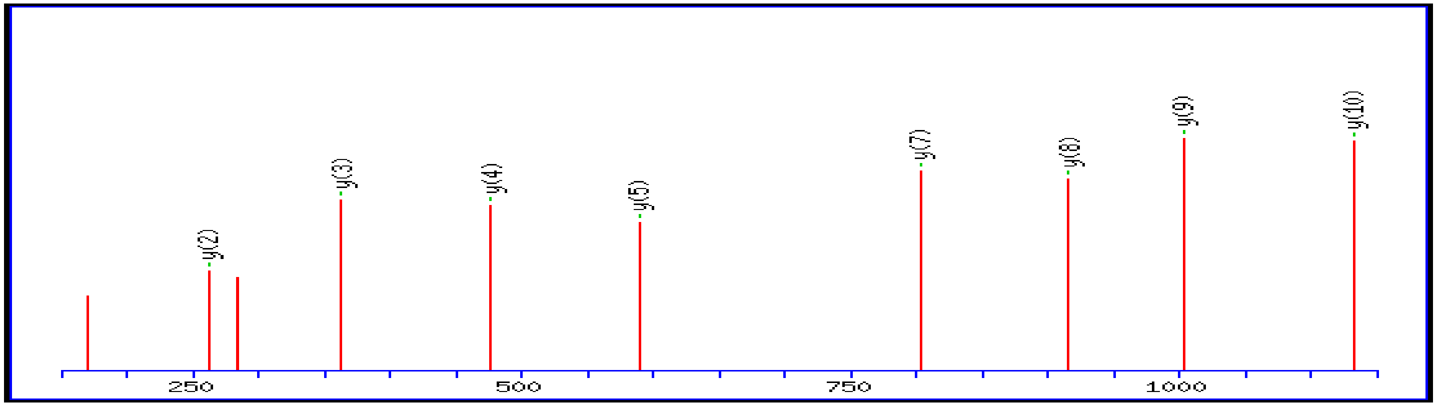

Monoisotopic mass of neutral peptide Mr(calc): 1703.8941
Fixed modifications: Carbamidomethyl (C) (apply to specified residues or termini only)
Ions Score: 82 Expect: 8.7e-07
Matches : 8/142 fragment ions using 8 most intense peaks (help)

#	b	b^{++}	b^{0}	b^{0++}	Seq.	y	y^{++}	y^{*}	y^{*++}	y^{0}	y^{0++}	#
1	58.0287	29.5180			G							16
2	171.1128	86.0600			L	1647.8800	824.4436	1630.8534	815.9303	1629.8694	815.4383	15
3	228.1343	114.5708			G	1534.7959	767.9016	1517.7693	759.3883	1516.7853	758.8963	14
4	329.1819	165.0946	311.1714	156.0893	T	1477.7744	739.3909	1460.7479	730.8776	1459.7639	730.3856	13
5	444.2089	222.6081	426.1983	213.6028	D	1376.7268	688.8670	1359.7002	680.3537	1358.7162	679.8617	12
6	573.2515	287.1294	555.2409	278.1241	E	1261.6998	631.3535	1244.6733	622.8403	1243.6892	622.3483	11
7	702.2941	351.6507	684.2835	342.6454	E	*1132.6572*	566.8322	1115.6307	558.3190	1114.6466	557.8270	10
8	789.3261	395.1667	771.3155	386.1614	S	*1003.6146*	502.3109	986.5881	493.7977	985.6041	493.3057	9
9	902.4102	451.7087	884.3996	442.7034	I	*916.5826*	458.7949	899.5560	450.2817	898.5720	449.7897	8
10	1015.4942	508.2508	997.4837	499.2455	L	*803.4985*	402.2529	786.4720	393.7396	785.4880	393.2476	7
11	1116.5419	558.7746	1098.5313	549.7693	T	690.4145	345.7109	673.3879	337.1976	672.4039	336.7056	6
12	1229.6260	615.3166	1211.6154	606.3113	L	*589.3668*	295.1870	572.3402	286.6738	571.3562	286.1817	5
13	1342.7100	671.8587	1324.6995	662.8534	L	*476.2827*	238.6450	459.2562	230.1317	458.2722	229.6397	4
14	1443.7577	722.3825	1425.7472	713.3772	T	*363.1987*	182.1030	346.1721	173.5897	345.1881	173.0977	3
15	1530.7897	765.8985	1512.7792	756.8932	S	*262.1510*	131.5791	245.1244	123.0659	244.1404	122.5738	2
16					R	175.1190	88.0631	158.0924	79.5498			1

MS/MS Fragmentation of **VGGTSDVEVNEK**
Found in **CH60_HUMAN** in **SwissProt**, 60 kDa heat shock protein, mitochondrial OS=Homo sapiens GN=HSPD1 PE=1 SV=2

Match to Query 263: 1233.022648 from(617.518600,2+) intensity(2258.8000) index(9)
Data file IS_111911_22.pkl

Click mouse within plot area to zoom in by factor of two about that point
Or, Plot from 100 to 1300 Da Full range
Label all possible matches ○ Label matches used for scoring ◉
Show Y-axis ☐

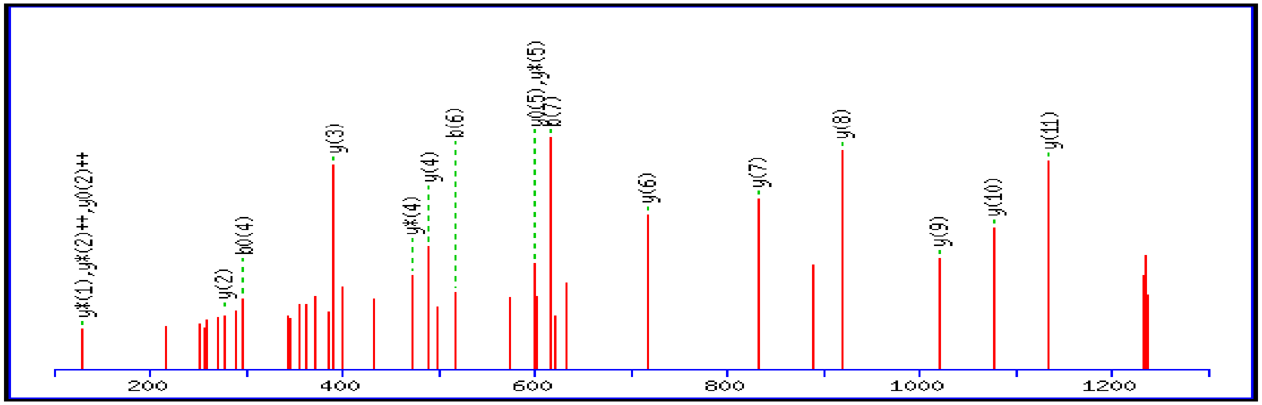

Monoisotopic mass of neutral peptide Mr(calc): 1232.5885
Fixed modifications: Carbamidomethyl (C) (apply to specified residues or termini only)
Ions Score: 74 Expect: 7e-06
Matches : 18/106 fragment ions using 25 most intense peaks (help)

#	b	b++	b*	b*++	b0	b0++	Seq.	y	y++	y*	y*++	y0	y0++	#
1	100.0757	50.5415					V							12
2	157.0972	79.0522					G	1134.5273	567.7673	1117.5008	559.2540	1116.5168	558.7620	11
3	214.1186	107.5629					G	1077.5059	539.2566	1060.4793	530.7433	1059.4953	530.2513	10
4	315.1663	158.0868			297.1557	149.0815	T	1020.4844	510.7458	1003.4578	502.2326	1002.4738	501.7406	9
5	402.1983	201.6028			384.1878	192.5975	S	919.4367	460.2220	902.4102	451.7087	901.4262	451.2167	8
6	517.2253	259.1163			499.2147	250.1110	D	832.4047	416.7060	815.3781	408.1927	814.3941	407.7007	7
7	616.2937	308.6505			598.2831	299.6452	V	717.3777	359.1925	700.3512	350.6792	699.3672	350.1872	6
8	745.3363	373.1718			727.3257	364.1665	E	618.3093	309.6583	601.2828	301.1450	600.2988	300.6530	5
9	844.4047	422.7060			826.3941	413.7007	V	489.2667	245.1370	472.2402	236.6237	471.2562	236.1317	4
10	958.4476	479.7274	941.4211	471.2142	940.4371	470.7222	N	390.1983	195.6028	373.1718	187.0895	372.1878	186.5975	3
11	1087.4902	544.2487	1070.4637	535.7355	1069.4796	535.2435	E	276.1554	138.5813	259.1288	130.0681	258.1448	129.5761	2
12							K	147.1128	74.0600	130.0863	65.5468			1

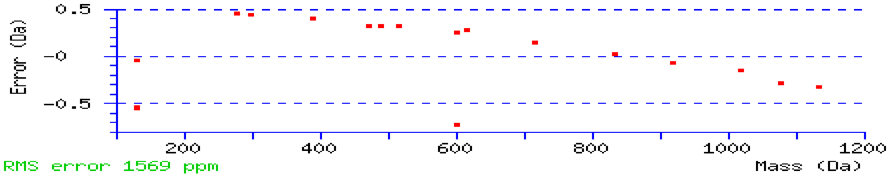

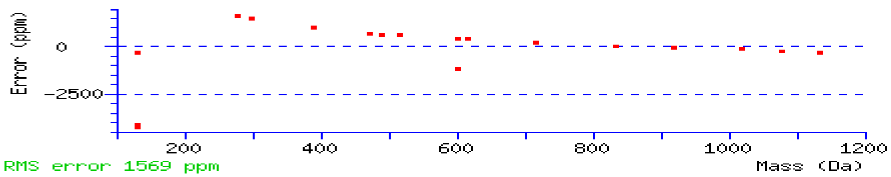

MS/MS Fragmentation of **EGIPALDNFLDKL**
Found in **EF2_HUMAN** in **SwissProt**, Elongation factor 2 OS=Homo sapiens GN=EEF2 PE=1 SV=4

Match to Query 363: 1444.010648 from(723.012600,2+) intensity(636.7000) index(138)
Data file IS_111911_22.pkl

Click mouse within plot area to zoom in by factor of two about that point
Or, Plot from 100 to 1200 Da Full range
Label all possible matches ○ Label matches used for scoring ◉
Show Y-axis ☐

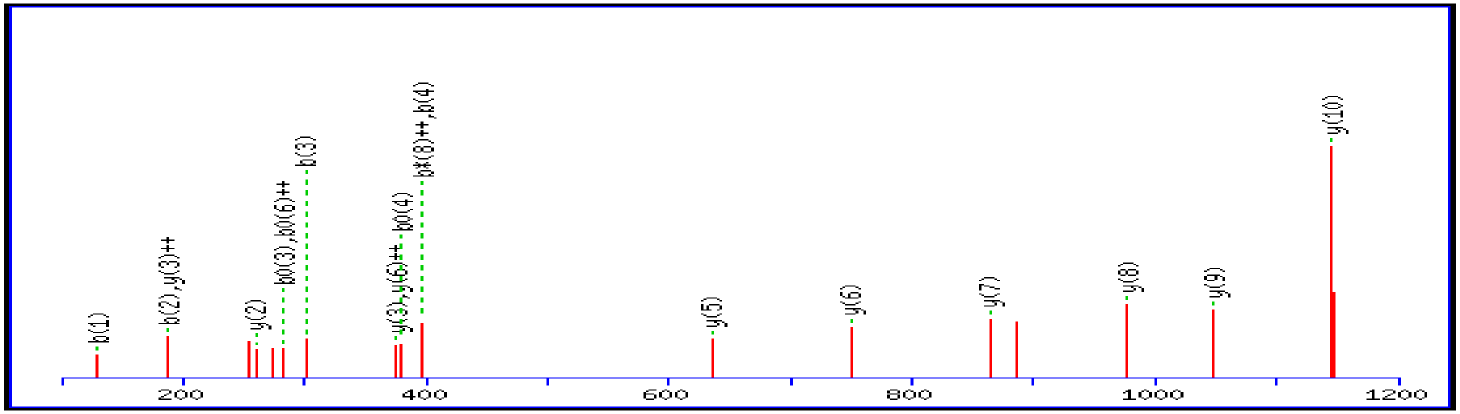

Monoisotopic mass of neutral peptide Mr(calc): 1443.7609
Fixed modifications: Carbamidomethyl (C) (apply to specified residues or termini only)
Ions Score: 68 Expect: 2.4e-05
Matches : 18/124 fragment ions using 19 most intense peaks (help)

#	b	b++	b*	b*++	b0	b0++	Seq.	y	y++	y*	y*++	y0	y0++	#
1	130.0499	65.5286			112.0393	56.5233	E							13
2	187.0713	94.0393			169.0608	85.0340	G	1315.7256	658.3665	1298.6991	649.8532	1297.7151	649.3612	12
3	300.1554	150.5813			282.1448	141.5761	I	1258.7042	629.8557	1241.6776	621.3424	1240.6936	620.8504	11
4	397.2082	199.1077			379.1976	190.1024	P	1145.6201	573.3137	1128.5936	564.8004	1127.6095	564.3084	10
5	468.2453	234.6263			450.2347	225.6210	A	1048.5673	524.7873	1031.5408	516.2740	1030.5568	515.7820	9
6	581.3293	291.1683			563.3188	282.1630	L	977.5302	489.2687	960.5037	480.7555	959.5197	480.2635	8
7	696.3563	348.6818			678.3457	339.6765	D	864.4462	432.7267	847.4196	424.2134	846.4356	423.7214	7
8	810.3992	405.7032	793.3727	397.1900	792.3886	396.6980	N	749.4192	375.2132	732.3927	366.7000	731.4087	366.2080	6
9	957.4676	479.2374	940.4411	470.7242	939.4571	470.2322	F	635.3763	318.1918	618.3497	309.6785	617.3657	309.1865	5
10	1070.5517	535.7795	1053.5251	527.2662	1052.5411	526.7742	L	488.3079	244.6576	471.2813	236.1443	470.2973	235.6523	4
11	1185.5786	593.2930	1168.5521	584.7797	1167.5681	584.2877	D	375.2238	188.1155	358.1973	179.6023	357.2132	179.1103	3
12	1313.6736	657.3404	1296.6470	648.8272	1295.6630	648.3352	K	260.1969	130.6021	243.1703	122.0888			2
13							L	132.1019	66.5546					1

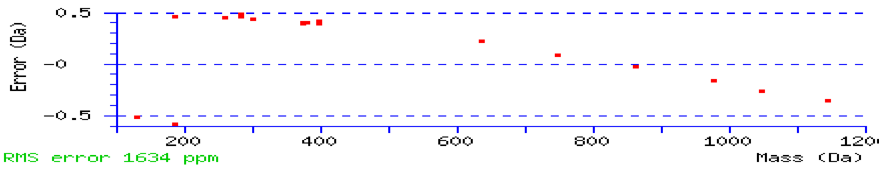

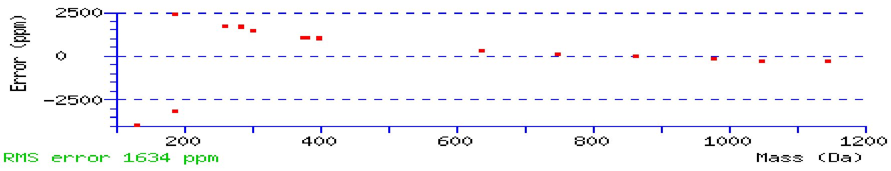

MS/MS Fragmentation of TPIGSFLGSLSLLPATK
Found in THIL_HUMAN in SwissProt, Acetyl-CoA acetyltransferase, mitochondrial OS=Homo sapiens GN=ACAT1 PE=1 SV=1

Match to Query 493: 1700.917248 from(851.465900,2+) intensity(567.3000) index(135)
Data file IS_111911_22.pkl

Click mouse within plot area to zoom in by factor of two about that point
Or, Plot from 200 to 1400 Da Full range
Label all possible matches ○ Label matches used for scoring ◉
Show Y-axis ☐

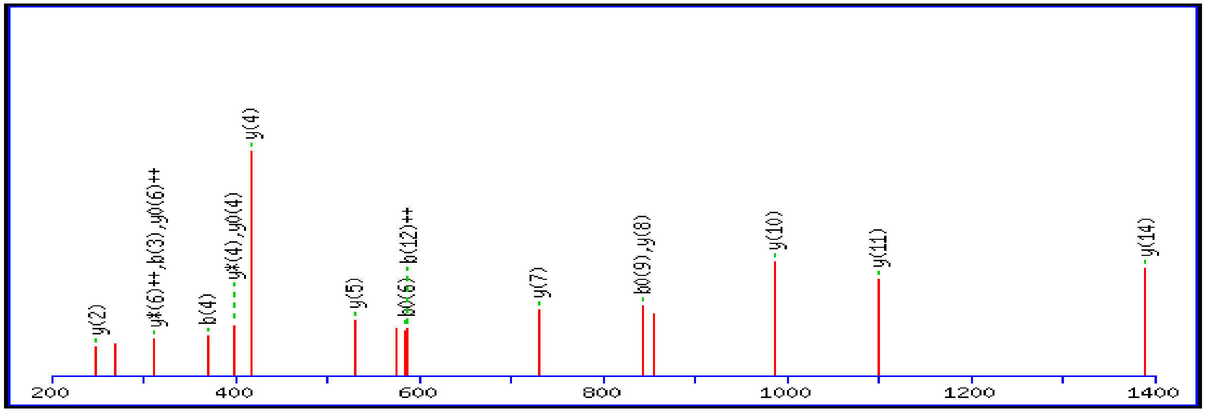

Monoisotopic mass of neutral peptide Mr(calc): 1700.9713
Fixed modifications: Carbamidomethyl (C) (apply to specified residues or termini only)
Ions Score: 60 Expect: 0.00013
Matches : 17/158 fragment ions using 17 most intense peaks (help)

#	b	b++	b0	b0++	Seq.	y	y++	y*	y*++	y0	y0++	#
1	102.0550	51.5311	84.0444	42.5258	T							17
2	199.1077	100.0575	181.0972	91.0522	P	1600.9309	800.9691	1583.9043	792.4558	1582.9203	791.9638	16
3	312.1918	156.5995	294.1812	147.5942	I	1503.8781	752.4427	1486.8516	743.9294	1485.8675	743.4374	15
4	369.2132	185.1103	351.2027	176.1050	G	1390.7940	695.9007	1373.7675	687.3874	1372.7835	686.8954	14
5	456.2453	228.6263	438.2347	219.6210	S	1333.7726	667.3899	1316.7460	658.8767	1315.7620	658.3846	13
6	603.3137	302.1605	585.3031	293.1552	F	1246.7406	623.8739	1229.7140	615.3606	1228.7300	614.8686	12
7	716.3978	358.7025	698.3872	349.6972	L	1099.6721	550.3397	1082.6456	541.8264	1081.6616	541.3344	11
8	773.4192	387.2132	755.4087	378.2080	G	986.5881	493.7977	969.5615	485.2844	968.5775	484.7924	10
9	860.4512	430.7293	842.4407	421.7240	S	929.5666	465.2869	912.5401	456.7737	911.5560	456.2817	9
10	973.5353	487.2713	955.5247	478.2660	L	842.5346	421.7709	825.5080	413.2577	824.5240	412.7656	8
11	1060.5673	530.7873	1042.5568	521.7820	S	729.4505	365.2289	712.4240	356.7156	711.4400	356.2236	7
12	1173.6514	587.3293	1155.6408	578.3241	L	642.4185	321.7129	625.3919	313.1996	624.4079	312.7076	6
13	1286.7355	643.8714	1268.7249	634.8661	L	529.3344	265.1709	512.3079	256.6576	511.3239	256.1656	5
14	1383.7882	692.3978	1365.7777	683.3925	P	416.2504	208.6288	399.2238	200.1155	398.2398	199.6235	4
15	1454.8253	727.9163	1436.8148	718.9110	A	319.1976	160.1024	302.1710	151.5892	301.1870	151.0972	3
16	1555.8730	778.4401	1537.8625	769.4349	T	248.1605	124.5839	231.1339	116.0706	230.1499	115.5786	2
17					K	147.1128	74.0600	130.0863	65.5468			1

MS/MS Fragmentation of **VPAINVNDSVTK**
Found in **SAHH_HUMAN** in **SwissProt**, Adenosylhomocysteinase OS=Homo sapiens GN=AHCY PE=1 SV=4

Match to Query 275: 1256.107448 from(629.061000,2+) intensity(13749.4000) index(546)
Data file IS_111911_22.pkl

Click mouse within plot area to zoom in by factor of two about that point
Or, [Plot from] [100] to [1300] Da [Full range]
Label all possible matches ○ Label matches used for scoring ◉
Show Y-axis ☐

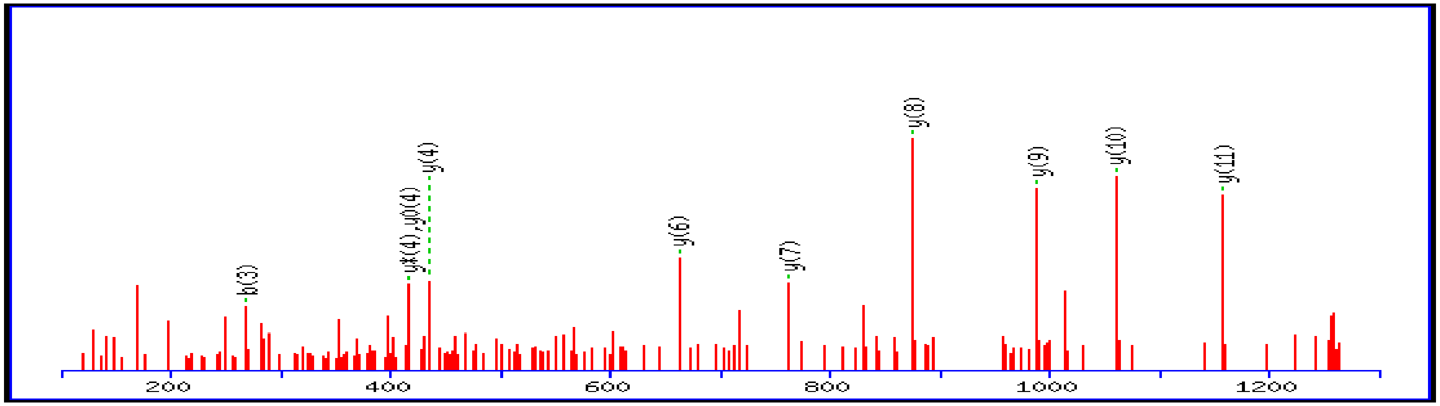

Monoisotopic mass of neutral peptide Mr(calc): 1255.6772
Fixed modifications: Carbamidomethyl (C) (apply to specified residues or termini only)
Ions Score: 53 Expect: 0.00078
Matches : 10/108 fragment ions using 12 most intense peaks (help)

#	b	b^{++}	b^{*}	b^{*++}	b^{0}	b^{0++}	Seq.	y	y^{++}	y^{*}	y^{*++}	y^{0}	y^{0++}	#
1	100.0757	50.5415					V							12
2	197.1285	99.0679					P	*1157.6161*	579.3117	1140.5895	570.7984	1139.6055	570.3064	11
3	268.1656	134.5864					A	*1060.5633*	530.7853	1043.5368	522.2720	1042.5527	521.7800	10
4	381.2496	191.1285					I	*989.5262*	495.2667	972.4997	486.7535	971.5156	486.2615	9
5	495.2926	248.1499	478.2660	239.6366			N	*876.4421*	438.7247	859.4156	430.2114	858.4316	429.7194	8
6	594.3610	297.6841	577.3344	289.1709			V	*762.3992*	381.7032	745.3727	373.1900	744.3886	372.6980	7
7	708.4039	354.7056	691.3774	346.1923			N	*663.3308*	332.1690	646.3042	323.6558	645.3202	323.1638	6
8	823.4308	412.2191	806.4043	403.7058	805.4203	403.2138	D	549.2879	275.1476	532.2613	266.6343	531.2773	266.1423	5
9	910.4629	455.7351	893.4363	447.2218	892.4523	446.7298	S	*434.2609*	217.6341	417.2344	209.1208	416.2504	208.6288	4
10	1009.5313	505.2693	992.5047	496.7560	991.5207	496.2640	V	347.2289	174.1181	330.2023	165.6048	329.2183	165.1128	3
11	1110.5790	555.7931	1093.5524	547.2798	1092.5684	546.7878	T	248.1605	124.5839	231.1339	116.0706	230.1499	115.5786	2
12							K	147.1128	74.0600	130.0863	65.5468			1

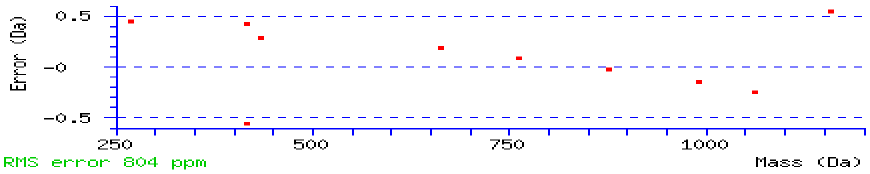

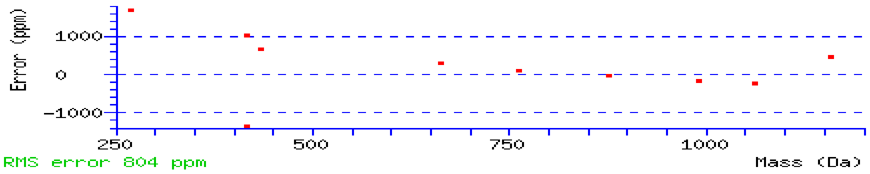

MS/MS Fragmentation of **LQMEAPHIIVGTPGR**
Found in **IF4A1_HUMAN** in **SwissProt**, Eukaryotic initiation factor 4A-I OS=Homo sapiens GN=EIF4A1 PE=1 SV=1

Match to Query 459: 1634.706072 from(545.909300,3+) intensity(6274.3000) index(193)
Data file IS_111911_22.pkl

Click mouse within plot area to zoom in by factor of two about that point
Or, Plot from 200 to 1700 Da Full range
Label all possible matches ○ Label matches used for scoring ◉
Show Y-axis ☐

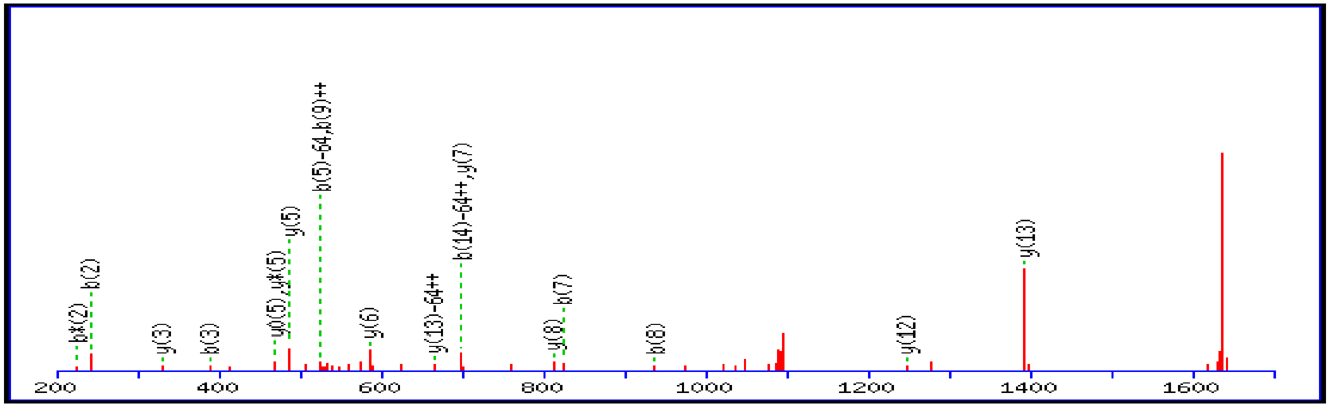

Monoisotopic mass of neutral peptide Mr(calc): 1633.8610
Fixed modifications: Carbamidomethyl (C) (apply to specified residues or termini only)
Variable modifications:
M3 : Oxidation (M), with neutral losses 0.0000(shown in table), 63.9983

#	b	b++	b*	b*++	b0	b0++	Seq.	y	y++	y*	y*++	y0	y0++	#
1	114.0913	57.5493					L							15
2	242.1499	121.5786	225.1234	113.0653			Q	1521.7842	761.3958	1504.7577	752.8825	1503.7737	752.3905	14
3	389.1853	195.0963	372.1588	186.5830			M	1393.7256	697.3665	1376.6991	688.8532	1375.7151	688.3612	13
4	518.2279	259.6176	501.2014	251.1043	500.2173	250.6123	E	1246.6902	623.8488	1229.6637	615.3355	1228.6797	614.8435	12
5	589.2650	295.1362	572.2385	286.6229	571.2545	286.1309	A	1117.6477	559.3275	1100.6211	550.8142	1099.6371	550.3222	11
6	686.3178	343.6625	669.2912	335.1493	668.3072	334.6573	P	1046.6105	523.8089	1029.5840	515.2956	1028.6000	514.8036	10
7	823.3767	412.1920	806.3502	403.6787	805.3661	403.1867	H	949.5578	475.2825	932.5312	466.7693	931.5472	466.2772	9
8	936.4608	468.7340	919.4342	460.2207	918.4502	459.7287	I	812.4989	406.7531	795.4723	398.2398	794.4883	397.7478	8
9	1049.5448	525.2761	1032.5183	516.7628	1031.5343	516.2708	I	699.4148	350.2110	682.3883	341.6978	681.4042	341.2058	7
10	1148.6132	574.8103	1131.5867	566.2970	1130.6027	565.8050	V	586.3307	293.6690	569.3042	285.1557	568.3202	284.6637	6
11	1205.6347	603.3210	1188.6082	594.8077	1187.6241	594.3157	G	487.2623	244.1348	470.2358	235.6215	469.2518	235.1295	5
12	1306.6824	653.8448	1289.6558	645.3316	1288.6718	644.8395	T	430.2409	215.6241	413.2143	207.1108	412.2303	206.6188	4
13	1403.7351	702.3712	1386.7086	693.8579	1385.7246	693.3659	P	329.1932	165.1002	312.1666	156.5870			3
14	1460.7566	730.8819	1443.7301	722.3687	1442.7460	721.8767	G	232.1404	116.5738	215.1139	108.0606			2
15							R	175.1190	88.0631	158.0924	79.5498			1

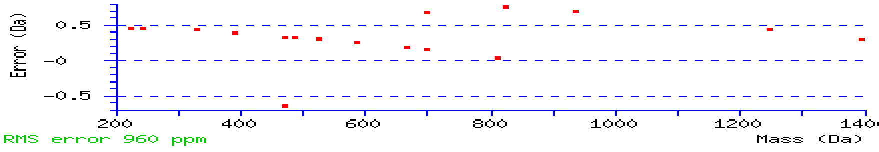

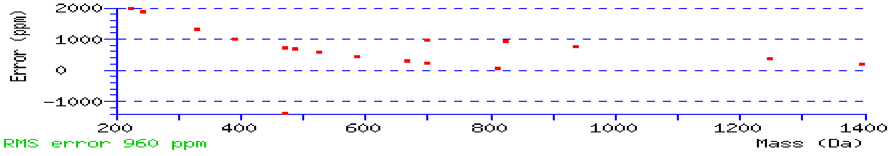

MS/MS Fragmentation of NKSPDAEAK
Found in DNJB9_HUMAN in SwissProt, DnaJ homolog subfamily B member 9 OS=Homo sapiens GN=DNAJB9 PE=1 SV=1

Match to Query 149: 959.181048 from(480.597800,2+) intensity(11477.2000) index(175)
Data file IS_111911_22.pkl

Click mouse within plot area to zoom in by factor of two about that point
Or, Plot from 100 to 900 Da Full range
Label all possible matches ○ Label matches used for scoring ◉
Show Y-axis ☐

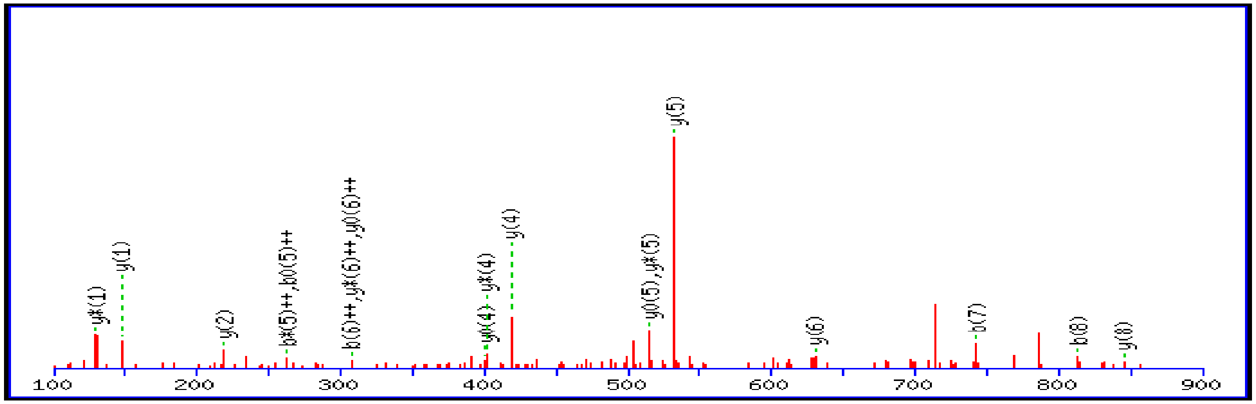

Monoisotopic mass of neutral peptide Mr(calc): 958.4719
Fixed modifications: Carbamidomethyl (C) (apply to specified residues or termini only)
Ions Score: 43 Expect: 0.0096
Matches : 18/88 fragment ions using 24 most intense peaks (help)

#	b	b^{++}	b*	b^{*++}	b^{0}	b^{0++}	Seq.	y	y^{++}	y*	y^{*++}	y^{0}	y^{0++}	#
1	115.0502	58.0287	98.0237	49.5155			N							9
2	243.1452	122.0762	226.1186	113.5629			K	845.4363	423.2218	828.4098	414.7085	827.4258	414.2165	8
3	330.1772	165.5922	313.1506	157.0790	312.1666	156.5870	S	717.3414	359.1743	700.3148	350.6610	699.3308	350.1690	7
4	427.2300	214.1186	410.2034	205.6053	409.2194	205.1133	P	630.3093	315.6583	613.2828	307.1450	612.2988	306.6530	6
5	542.2569	271.6321	525.2304	263.1188	524.2463	262.6268	D	533.2566	267.1319	516.2300	258.6186	515.2460	258.1266	5
6	613.2940	307.1506	596.2675	298.6374	595.2835	298.1454	A	418.2296	209.6185	401.2031	201.1052	400.2191	200.6132	4
7	742.3366	371.6719	725.3101	363.1587	724.3260	362.6667	E	347.1925	174.0999	330.1660	165.5866	329.1819	165.0946	3
8	813.3737	407.1905	796.3472	398.6772	795.3632	398.1852	A	218.1499	109.5786	201.1234	101.0653			2
9							K	147.1128	74.0600	130.0863	65.5468			1

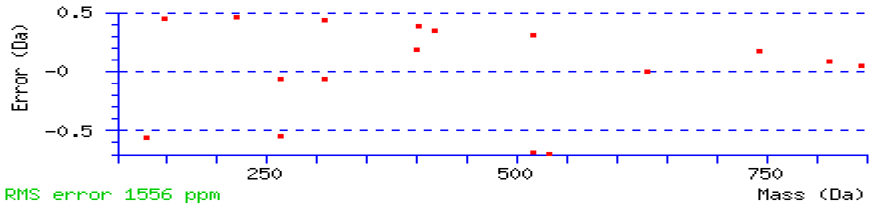

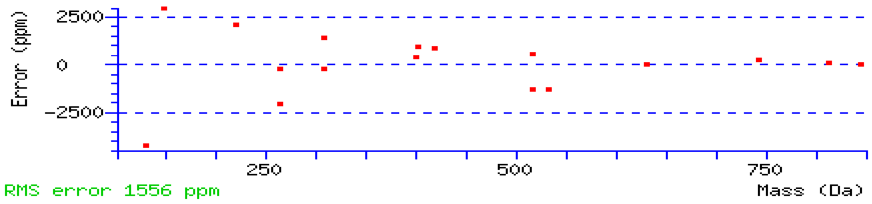

MS/MS Fragmentation of **MSVQPTVSLGGFEITPPVVLR**
Found in **NPM_HUMAN** in **SwissProt**, Nucleophosmin OS=Homo sapiens GN=NPM1 PE=1 SV=2

Match to Query 625: 2241.534448 from(1121.774500,2+) intensity(2062.0000) index(279)
Data file IS_111911_22.pkl

Click mouse within plot area to zoom in by factor of two about that point
Or, Plot from 100 to 1800 Da Full range
Label all possible matches ○ Label matches used for scoring ◉
Show Y-axis ☐

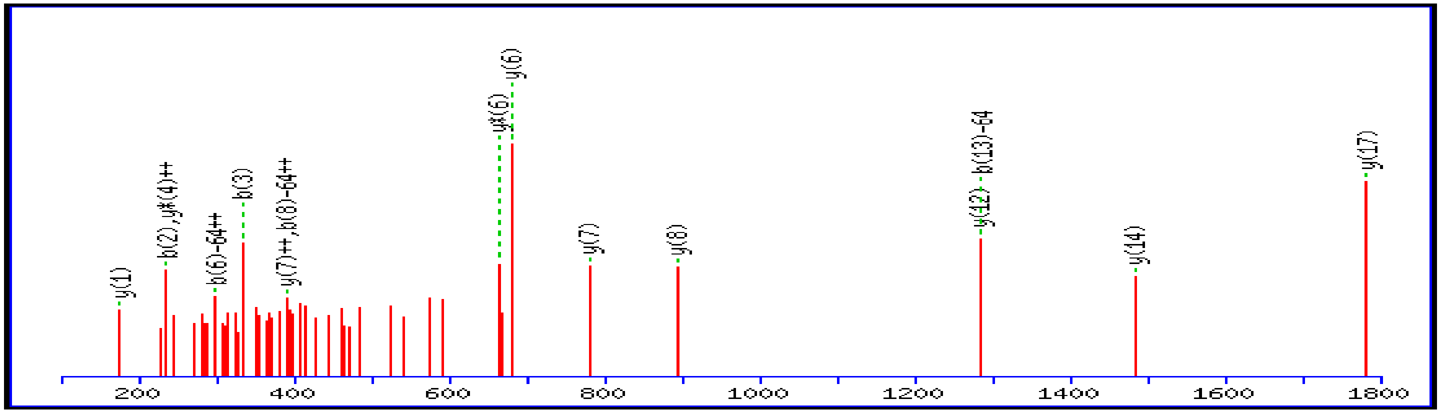

Monoisotopic mass of neutral peptide Mr(calc): 2242.2032
Fixed modifications: Carbamidomethyl (C) (apply to specified residues or termini only)
Variable modifications:
M1 : Oxidation (M), with neutral losses 0.0000(shown in table), 63.9983

#	b	b^{++}	b^{*}	b^{*++}	b^{0}	b^{0++}	Seq.	y	y^{++}	y^{*}	y^{*++}	y^{0}	y^{0++}	#
1	148.0427	74.5250					M							21
2	235.0747	118.0410			217.0641	109.0357	S	2096.1750	1048.5911	2079.1485	1040.0779	2078.1645	1039.5859	20
3	334.1431	167.5752			316.1326	158.5699	V	2009.1430	1005.0751	1992.1164	996.5619	1991.1324	996.0699	19
4	462.2017	231.6045	445.1751	223.0912	444.1911	222.5992	Q	1910.0746	955.5409	1893.0480	947.0277	1892.0640	946.5356	18
5	559.2545	280.1309	542.2279	271.6176	541.2439	271.1256	P	*1782.0160*	891.5116	1764.9895	882.9984	1764.0054	882.5064	17
6	660.3021	330.6547	643.2756	322.1414	642.2916	321.6494	T	1684.9632	842.9853	1667.9367	834.4720	1666.9527	833.9800	16
7	759.3706	380.1889	742.3440	371.6756	741.3600	371.1836	V	1583.9156	792.4614	1566.8890	783.9481	1565.9050	783.4561	15
8	846.4026	423.7049	829.3760	415.1917	828.3920	414.6996	S	*1484.8471*	742.9272	1467.8206	734.4139	1466.8366	733.9219	14
9	959.4866	480.2470	942.4601	471.7337	941.4761	471.2417	L	1397.8151	699.4112	1380.7886	690.8979	1379.8045	690.4059	13
10	1016.5081	508.7577	999.4816	500.2444	998.4975	499.7524	G	*1284.7311*	642.8692	1267.7045	634.3559	1266.7205	633.8639	12
11	1073.5296	537.2684	1056.5030	528.7552	1055.5190	528.2631	G	1227.7096	614.3584	1210.6830	605.8452	1209.6990	605.3531	11
12	1220.5980	610.8026	1203.5714	602.2894	1202.5874	601.7973	F	1170.6881	585.8477	1153.6616	577.3344	1152.6776	576.8424	10
13	1349.6406	675.3239	1332.6140	666.8107	1331.6300	666.3186	E	1023.6197	512.3135	1006.5932	503.8002	1005.6091	503.3082	9
14	1462.7246	731.8660	1445.6981	723.3527	1444.7141	722.8607	I	*894.5771*	447.7922	877.5506	439.2789	876.5666	438.7869	8
15	1563.7723	782.3898	1546.7458	773.8765	1545.7618	773.3845	T	*781.4931*	391.2502	764.4665	382.7369	763.4825	382.2449	7
16	1660.8251	830.9162	1643.7985	822.4029	1642.8145	821.9109	P	*680.4454*	340.7263	663.4188	332.2130			6
17	1757.8779	879.4426	1740.8513	870.9293	1739.8673	870.4373	P	583.3926	292.1999	566.3661	283.6867			5
18	1856.9463	928.9768	1839.9197	920.4635	1838.9357	919.9715	V	486.3398	243.6736	469.3133	235.1603			4
19	1956.0147	978.5110	1938.9881	969.9977	1938.0041	969.5057	V	387.2714	194.1394	370.2449	185.6261			3
20	2069.0987	1035.0530	2052.0722	1026.5397	2051.0882	1026.0477	L	288.2030	144.6051	271.1765	136.0919			2
21							R	*175.1190*	88.0631	158.0924	79.5498			1

MS/MS Fragmentation of **ILLTEPPMNPTK**
Found in **ARP2_HUMAN** in **SwissProt**, Actin-related protein 2 OS=Homo sapiens GN=ACTR2 PE=1 SV=1

Match to Query 320: 1369.061848 from(685.538200,2+) intensity(6883.2000) index(368)
Data file IS_111911_22.pkl

Click mouse within plot area to zoom in by factor of two about that point
Or, Plot from 100 to 1400 Da Full range
Label all possible matches ○ Label matches used for scoring ◉
Show Y-axis ☐

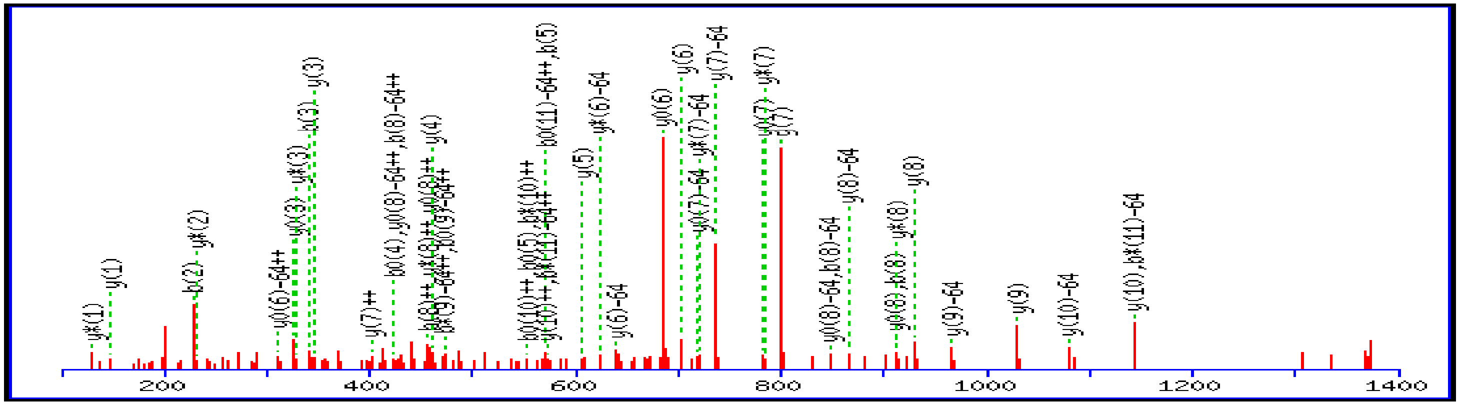

Monoisotopic mass of neutral peptide Mr(calc): 1368.7323
Fixed modifications: Carbamidomethyl (C) (apply to specified residues or termini only)
Variable modifications:
M8 : Oxidation (M), with neutral losses 0.0000(shown in table), 63.9983

#	b	b++	b*	b*++	b0	b0++	Seq.	y	y++	y*	y*++	y0	y0++	#
1	114.0913	57.5493					I							12
2	227.1754	114.0913					L	1256.6555	628.8314	1239.6290	620.3181	1238.6449	619.8261	11
3	340.2595	170.6334					L	1143.5714	572.2894	1126.5449	563.7761	1125.5609	563.2841	10
4	441.3071	221.1572			423.2966	212.1519	T	1030.4874	515.7473	1013.4608	507.2341	1012.4768	506.7420	9
5	570.3497	285.6785			552.3392	276.6732	E	929.4397	465.2235	912.4131	456.7102	911.4291	456.2182	8
6	667.4025	334.2049			649.3919	325.1996	P	800.3971	400.7022	783.3706	392.1889	782.3865	391.6969	7
7	764.4553	382.7313			746.4447	373.7260	P	703.3443	352.1758	686.3178	343.6625	685.3338	343.1705	6
8	911.4907	456.2490			893.4801	447.2437	M	606.2916	303.6494	589.2650	295.1362	588.2810	294.6441	5
9	1025.5336	513.2704	1008.5070	504.7572	1007.5230	504.2652	N	459.2562	230.1317	442.2296	221.6185	441.2456	221.1264	4
10	1122.5864	561.7968	1105.5598	553.2835	1104.5758	552.7915	P	345.2132	173.1103	328.1867	164.5970	327.2027	164.1050	3
11	1223.6340	612.3207	1206.6075	603.8074	1205.6235	603.3154	T	248.1605	124.5839	231.1339	116.0706	230.1499	115.5786	2
12							K	147.1128	74.0600	130.0863	65.5468			1

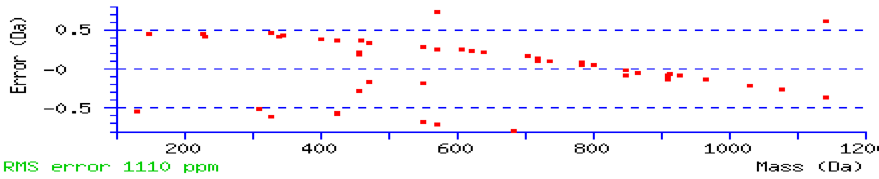

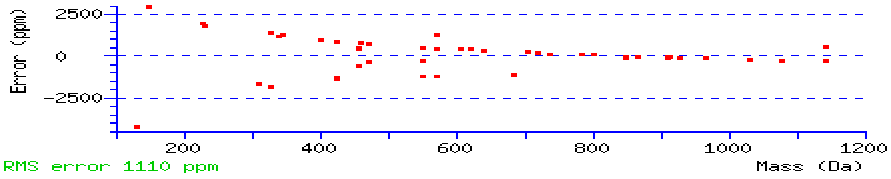

MS/MS Fragmentation of **YLYGIRVEVR**
Found in **PLCA_HUMAN** in **SwissProt**, 1-acyl-sn-glycerol-3-phosphate acyltransferase alpha OS=Homo sapiens GN=AGPAT1 PE=2 SV=2

Match to Query 287: 1267.086848 from(634.550700,2+) intensity(320.6000) index(465)
Data file IS_111911_22.pkl

Click mouse within plot area to zoom in by factor of two about that point
Or, Plot from 100 to 1150 Da Full range
Label all possible matches ○ Label matches used for scoring ◉
Show Y-axis ☐

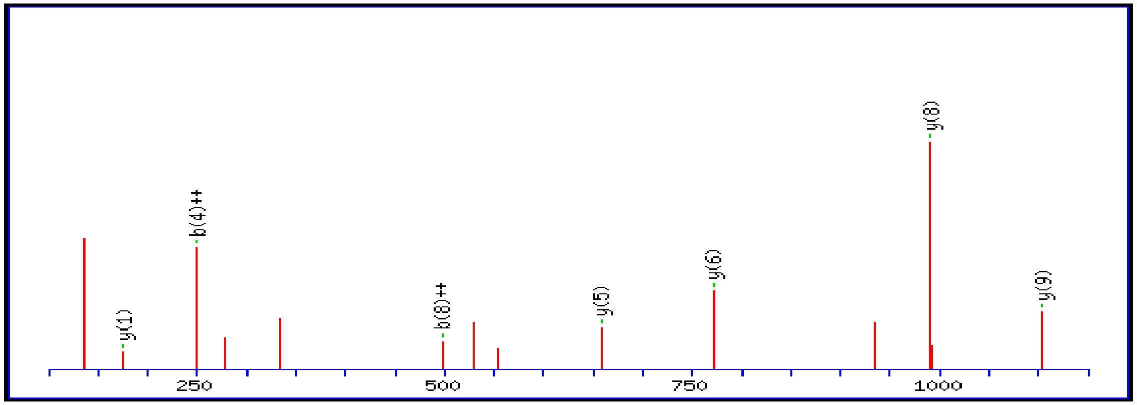

Monoisotopic mass of neutral peptide Mr(calc): 1266.7084
Fixed modifications: Carbamidomethyl (C) (apply to specified residues or termini only)
Ions Score: 26 Expect: 0.33
Matches : 7/80 fragment ions using 15 most intense peaks (help)

#	b	b^{++}	b^*	b^{*++}	b^0	b^{0++}	Seq.	y	y^{++}	y^*	y^{*++}	y^0	y^{0++}	#
1	164.0706	82.5389					Y							10
2	277.1547	139.0810					L	*1104.6524*	552.8298	1087.6259	544.3166	1086.6418	543.8246	9
3	440.2180	220.6126					Y	*991.5683*	496.2878	974.5418	487.7745	973.5578	487.2825	8
4	497.2395	**249.1234**					G	828.5050	414.7561	811.4785	406.2429	810.4944	405.7509	7
5	610.3235	305.6654					I	*771.4835*	386.2454	754.4570	377.7321	753.4730	377.2401	6
6	766.4246	383.7160	749.3981	375.2027			R	*658.3995*	329.7034	641.3729	321.1901	640.3889	320.6981	5
7	865.4931	433.2502	848.4665	424.7369			V	502.2984	251.6528	485.2718	243.1395	484.2878	242.6475	4
8	994.5356	**497.7715**	977.5091	489.2582	976.5251	488.7662	E	403.2300	202.1186	386.2034	193.6053	385.2194	193.1133	3
9	1093.6041	547.3057	1076.5775	538.7924	1075.5935	538.3004	V	274.1874	137.5973	257.1608	129.0840			2
10							R	*175.1190*	88.0631	158.0924	79.5498			1

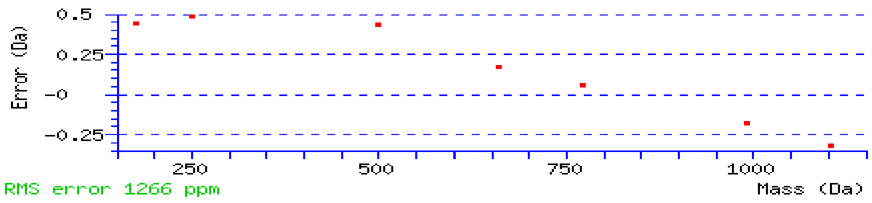

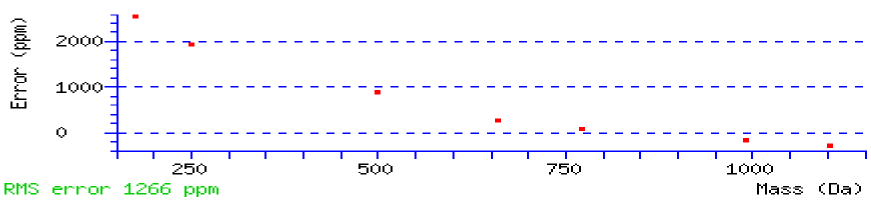

MS/MS Fragmentation of **VVLAYEPVWAIGTGK**
Found in **TPIS_HUMAN** in **SwissProt**, Triosephosphate isomerase OS=Homo sapiens GN=TPI1 PE=1 SV=3

Match to Query 294: 1601.954848 from(801.984700,2+) intensity(22043.7000) index(345)
Data file IS_111911_23a.txt

Click mouse within plot area to zoom in by factor of two about that point
Or, Plot from 100 to 1700 Da Full range
Label all possible matches ○ Label matches used for scoring ◉
Show Y-axis ☐

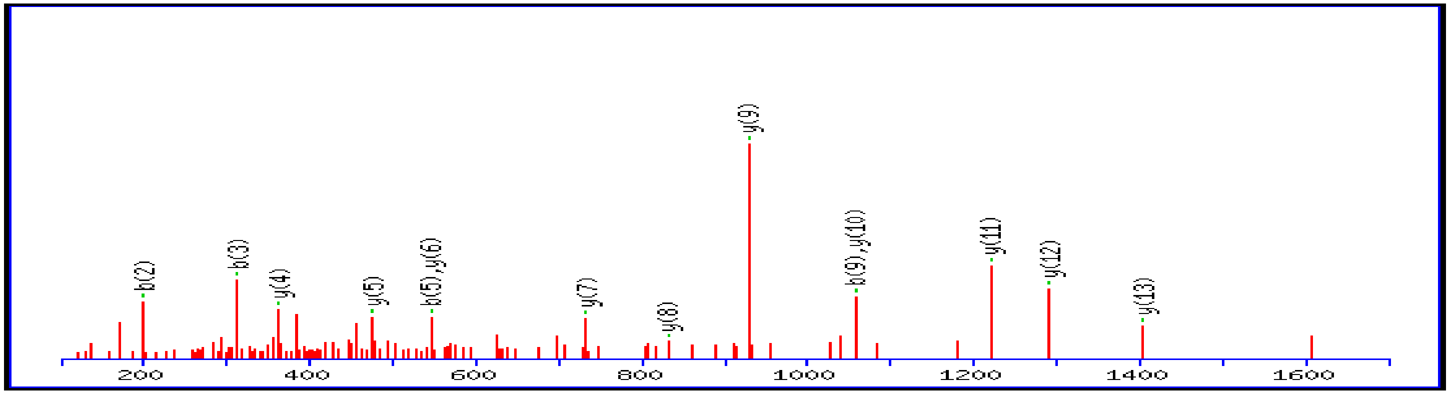

Monoisotopic mass of neutral peptide Mr(calc): 1601.8817
Fixed modifications: Carbamidomethyl (C) (apply to specified residues or termini only)
Ions Score: 101 Expect: 1.2e-08
Matches : 14/126 fragment ions using 13 most intense peaks (help)

#	b	b++	b0	b0++	Seq.	y	y++	y*	y*++	y0	y0++	#
1	100.0757	50.5415			V							15
2	**199.1441**	100.0757			V	1503.8206	752.4139	1486.7940	743.9007	1485.8100	743.4087	14
3	**312.2282**	156.6177			L	***1404.7522***	702.8797	1387.7256	694.3665	1386.7416	693.8744	13
4	383.2653	192.1363			A	***1291.6681***	646.3377	1274.6416	637.8244	1273.6576	637.3324	12
5	**546.3286**	273.6679			Y	***1220.6310***	610.8191	1203.6045	602.3059	1202.6204	601.8139	11
6	675.3712	338.1892	657.3606	329.1840	E	***1057.5677***	529.2875	1040.5411	520.7742	1039.5571	520.2822	10
7	772.4240	386.7156	754.4134	377.7103	P	***928.5251***	464.7662	911.4985	456.2529	910.5145	455.7609	9
8	871.4924	436.2498	853.4818	427.2445	V	***831.4723***	416.2398	814.4458	407.7265	813.4618	407.2345	8
9	**1057.5717**	529.2895	1039.5611	520.2842	W	***732.4039***	366.7056	715.3774	358.1923	714.3933	357.7003	7
10	1128.6088	564.8080	1110.5982	555.8028	A	***546.3246***	273.6659	529.2980	265.1527	528.3140	264.6606	6
11	1241.6929	621.3501	1223.6823	612.3448	I	***475.2875***	238.1474	458.2609	229.6341	457.2769	229.1421	5
12	1298.7143	649.8608	1280.7038	640.8555	G	***362.2034***	181.6053	345.1769	173.0921	344.1928	172.6001	4
13	1399.7620	700.3846	1381.7515	691.3794	T	305.1819	153.0946	288.1554	144.5813	287.1714	144.0893	3
14	1456.7835	728.8954	1438.7729	719.8901	G	204.1343	102.5708	187.1077	94.0575			2
15					K	147.1128	74.0600	130.0863	65.5468			1

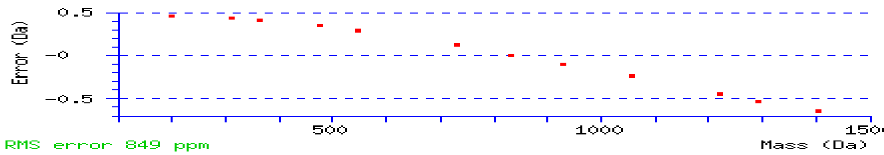

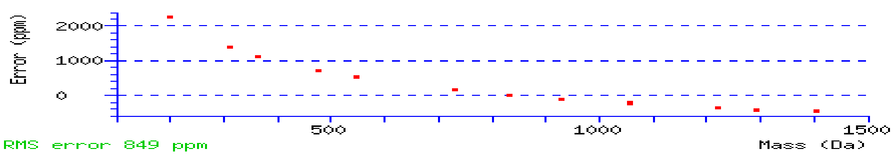

MS/MS Fragmentation of **LDNLVAILDINR**
Found in **TKT_HUMAN** in **SwissProt**, Transketolase OS=Homo sapiens GN=TKT PE=1 SV=3

Match to Query 238: 1368.086848 from(685.050700,2+) intensity(10547.2000) index(97)
Data file IS_111911_23a.txt

Click mouse within plot area to zoom in by factor of two about that point
Or, [Plot from] 100 to 1300 Da [Full range]
Label all possible matches ○ Label matches used for scoring ◉
Show Y-axis ☐

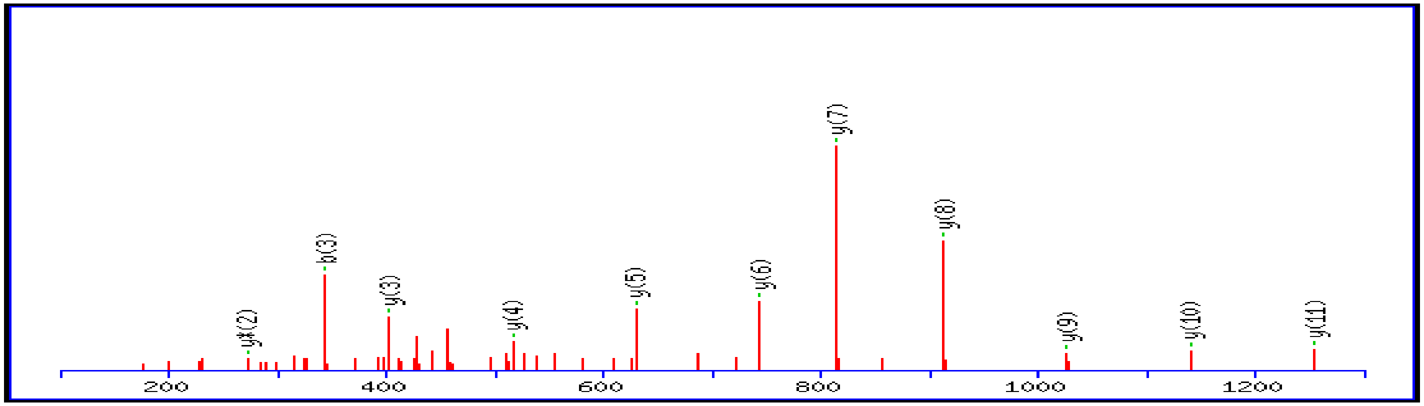

Monoisotopic mass of neutral peptide Mr(calc): 1367.7772
Fixed modifications: Carbamidomethyl (C) (apply to specified residues or termini only)
Ions Score: 93 Expect: 7e-08
Matches : 11/120 fragment ions using 11 most intense peaks (help)

#	b	b^{++}	b*	b^{*++}	b^{0}	b^{0++}	Seq.	y	y^{++}	y*	y^{*++}	y^{0}	y^{0++}	#
1	114.0913	57.5493					L							12
2	229.1183	115.0628			211.1077	106.0575	D	1255.7005	628.3539	1238.6739	619.8406	1237.6899	619.3486	11
3	343.1612	172.0842	326.1347	163.5710	325.1506	163.0790	N	1140.6735	570.8404	1123.6470	562.3271	1122.6630	561.8351	10
4	456.2453	228.6263	439.2187	220.1130	438.2347	219.6210	L	1026.6306	513.8189	1009.6041	505.3057	1008.6200	504.8137	9
5	555.3137	278.1605	538.2871	269.6472	537.3031	269.1552	V	913.5465	457.2769	896.5200	448.7636	895.5360	448.2716	8
6	626.3508	313.6790	609.3243	305.1658	608.3402	304.6738	A	814.4781	407.7427	797.4516	399.2294	796.4676	398.7374	7
7	739.4349	370.2211	722.4083	361.7078	721.4243	361.2158	I	743.4410	372.2241	726.4145	363.7109	725.4304	363.2189	6
8	852.5189	426.7631	835.4924	418.2498	834.5084	417.7578	L	630.3569	315.6821	613.3304	307.1688	612.3464	306.6768	5
9	967.5459	484.2766	950.5193	475.7633	949.5353	475.2713	D	517.2729	259.1401	500.2463	250.6268	499.2623	250.1348	4
10	1080.6299	540.8186	1063.6034	532.3053	1062.6194	531.8133	I	402.2459	201.6266	385.2194	193.1133			3
11	1194.6729	597.8401	1177.6463	589.3268	1176.6623	588.8348	N	289.1619	145.0846	272.1353	136.5713			2
12							R	175.1190	88.0631	158.0924	79.5498			1

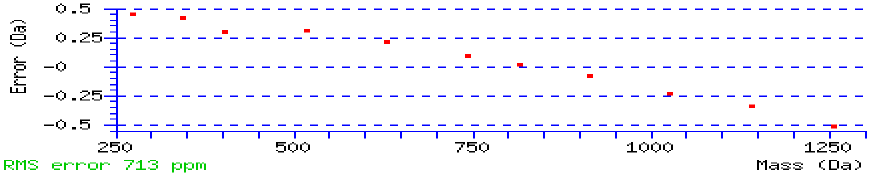

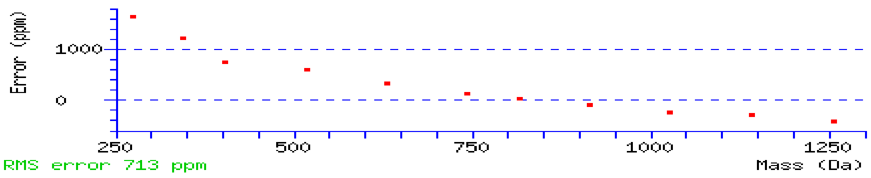

MS/MS Fragmentation of **GNDISSGTVLSDYVGSGPPK**
Found in **PEBP1_HUMAN** in **SwissProt**, Phosphatidylethanolamine-binding protein 1 OS=Homo sapiens GN=PEBP1 PE=1 SV=3

Match to Query 371: 1948.596848 from(975.305700,2+) intensity(2187.1000) index(312)
Data file IS_111911_23a.txt

Click mouse within plot area to zoom in by factor of two about that point
Or, Plot from 100 to 1600 Da Full range
Label all possible matches ○ Label matches used for scoring ◉
Show Y-axis ☐

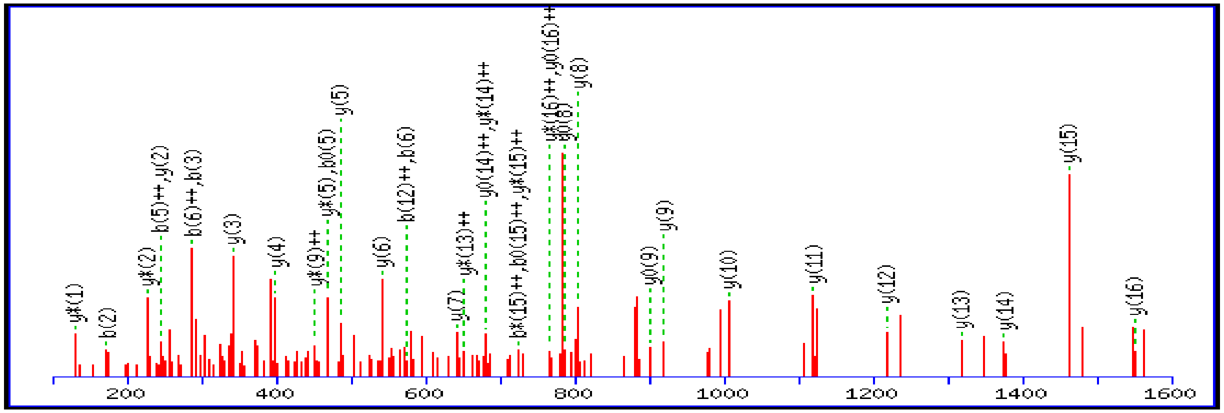

Monoisotopic mass of neutral peptide Mr(calc): 1948.9378
Fixed modifications: Carbamidomethyl (C) (apply to specified residues or termini only)
Ions Score: 90 Expect: 1.1e-07
Matches : 37/214 fragment ions using 62 most intense peaks (help)

#	b	b^{++}	b^{*}	b^{*++}	b^{0}	b^{0++}	Seq.	y	y^{++}	y^{*}	y^{*++}	y^{0}	y^{0++}	#
1	58.0287	29.5180					G							20
2	172.0717	86.5395	155.0451	78.0262			N	1892.9236	946.9654	1875.8971	938.4522	1874.9130	937.9602	19
3	287.0986	144.0529	270.0721	135.5397	269.0880	135.0477	D	1778.8807	889.9440	1761.8541	881.4307	1760.8701	880.9387	18
4	400.1827	200.5950	383.1561	192.0817	382.1721	191.5897	I	1663.8537	832.4305	1646.8272	823.9172	1645.8432	823.4252	17
5	487.2147	244.1110	470.1882	235.5977	469.2041	235.1057	S	1550.7697	775.8885	1533.7431	767.3752	1532.7591	766.8832	16
6	574.2467	287.6270	557.2202	279.1137	556.2362	278.6217	S	1463.7377	732.3725	1446.7111	723.8592	1445.7271	723.3672	15
7	631.2682	316.1377	614.2416	307.6245	613.2576	307.1325	G	1376.7056	688.8564	1359.6791	680.3432	1358.6951	679.8512	14
8	732.3159	366.6616	715.2893	358.1483	714.3053	357.6563	T	1319.6842	660.3457	1302.6576	651.8324	1301.6736	651.3404	13
9	831.3843	416.1958	814.3577	407.6825	813.3737	407.1905	V	1218.6365	609.8219	1201.6099	601.3086	1200.6259	600.8166	12
10	944.4684	472.7378	927.4418	464.2245	926.4578	463.7325	L	1119.5681	560.2877	1102.5415	551.7744	1101.5575	551.2824	11
11	1031.5004	516.2538	1014.4738	507.7406	1013.4898	507.2485	S	1006.4840	503.7456	989.4575	495.2324	988.4734	494.7404	10
12	1146.5273	573.7673	1129.5008	565.2540	1128.5168	564.7620	D	919.4520	460.2296	902.4254	451.7164	901.4414	451.2243	9
13	1309.5907	655.2990	1292.5641	646.7857	1291.5801	646.2937	Y	804.4250	402.7162	787.3985	394.2029	786.4145	393.7109	8
14	1408.6591	704.8332	1391.6325	696.3199	1390.6485	695.8279	V	641.3617	321.1845	624.3352	312.6712	623.3511	312.1792	7
15	1465.6805	733.3439	1448.6540	724.8306	1447.6700	724.3386	G	542.2933	271.6503	525.2667	263.1370	524.2827	262.6450	6
16	1552.7126	776.8599	1535.6860	768.3466	1534.7020	767.8546	S	485.2718	243.1396	468.2453	234.6263	467.2613	234.1343	5
17	1609.7340	805.3706	1592.7075	796.8574	1591.7235	796.3654	G	398.2398	199.6235	381.2132	191.1103			4
18	1706.7868	853.8970	1689.7602	845.3838	1688.7762	844.8917	P	341.2183	171.1128	324.1918	162.5995			3
19	1803.8395	902.4234	1786.8130	893.9101	1785.8290	893.4181	P	244.1656	122.5864	227.1390	114.0731			2
20							K	147.1128	74.0600	130.0863	65.5468			1

MS/MS Fragmentation of **FGVEQDVDMVFASFIR**
Found in **KPYM_HUMAN** in **SwissProt**, Pyruvate kinase isozymes M1/M2 OS=Homo sapiens GN=PKM PE=1 SV=4

Match to Query 353: 1874.648848 from(938.331700,2+) intensity(6246.6000) index(140)
Data file IS_111911_23a.txt

Click mouse within plot area to zoom in by factor of two about that point
Or, Plot from 100 to 1600 Da Full range
Label all possible matches ○ Label matches used for scoring ◉
Show Y-axis ☐

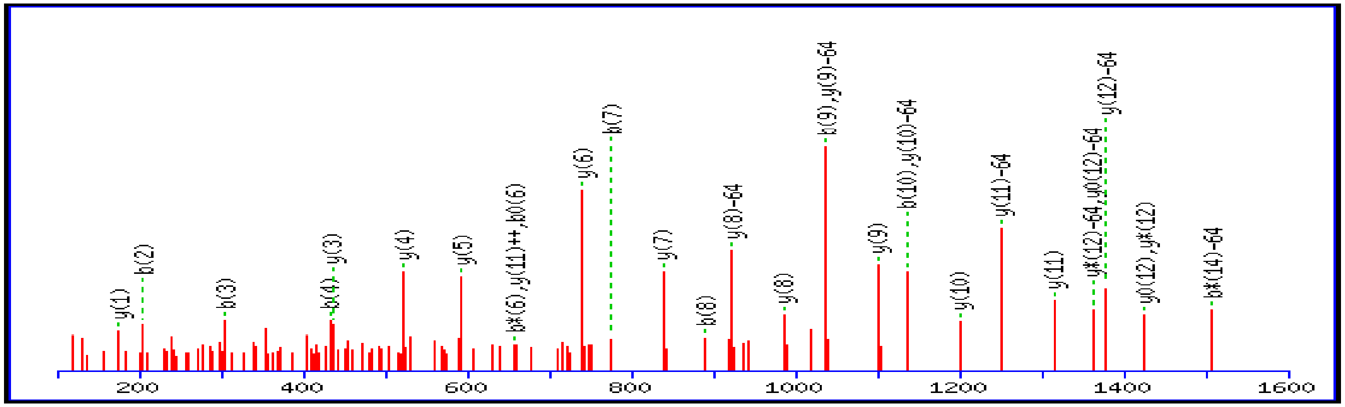

Monoisotopic mass of neutral peptide Mr(calc): 1874.8873
Fixed modifications: Carbamidomethyl (C) (apply to specified residues or termini only)
Variable modifications:
M9 : Oxidation (M), with neutral losses 0.0000(shown in table), 63.9983

Ions Score: 87 Expect: 2.4e-07
Matches : 30/250 fragment ions using 28 most intense peaks (help)

#	b	b^{++}	b^{*}	b^{*++}	b^{0}	b^{0++}	Seq.	y	y^{++}	y^{*}	y^{*++}	y^{0}	y^{0++}	#
1	148.0757	74.5415					F							16
2	*205.0972*	103.0522					G	1728.8261	864.9167	1711.7996	856.4034	1710.8156	855.9114	15
3	*304.1656*	152.5864					V	1671.8047	836.4060	1654.7781	827.8927	1653.7941	827.4007	14
4	*433.2082*	217.1077			415.1976	208.1024	E	1572.7363	786.8718	1555.7097	778.3585	1554.7257	777.8665	13
5	561.2667	281.1370	544.2402	272.6237	543.2562	272.1317	Q	1443.6937	722.3505	1426.6671	713.8372	1425.6831	713.3452	12
6	676.2937	338.6505	659.2671	330.1372	658.2831	329.6452	D	*1315.6351*	658.3212	1298.6086	649.8079	1297.6245	649.3159	11
7	*775.3621*	388.1847	758.3355	379.6714	757.3515	379.1794	V	*1200.6082*	600.8077	1183.5816	592.2944	1182.5976	591.8024	10
8	*890.3890*	445.6982	873.3625	437.1849	872.3785	436.6929	D	*1101.5397*	551.2735	1084.5132	542.7602	1083.5292	542.2682	9
9	*1037.4244*	519.2159	1020.3979	510.7026	1019.4139	510.2106	M	*986.5128*	493.7600	969.4863	485.2468	968.5022	484.7548	8
10	*1136.4929*	568.7501	1119.4663	560.2368	1118.4823	559.7448	V	*839.4774*	420.2423	822.4509	411.7291	821.4668	411.2371	7
11	1283.5613	642.2843	1266.5347	633.7710	1265.5507	633.2790	F	*740.4090*	370.7081	723.3824	362.1949	722.3984	361.7028	6
12	1354.5984	677.8028	1337.5718	669.2896	1336.5878	668.7975	A	*593.3406*	297.1739	576.3140	288.6606	575.3300	288.1686	5
13	1441.6304	721.3188	1424.6039	712.8056	1423.6198	712.3136	S	*522.3035*	261.6554	505.2769	253.1421	504.2929	252.6501	4
14	1588.6988	794.8530	1571.6723	786.3398	1570.6883	785.8478	F	*435.2714*	218.1394	418.2449	209.6261			3
15	1701.7829	851.3951	1684.7563	842.8818	1683.7723	842.3898	I	288.2030	144.6051	271.1765	136.0919			2
16							R	*175.1190*	88.0631	158.0924	79.5498			1

MS/MS Fragmentation of **FYALSASFEPFSNK**
Found in **CALR_HUMAN** in **SwissProt**, Calreticulin OS=Homo sapiens GN=CALR PE=1 SV=1

Match to Query 295: 1606.841248 from(804.427900,2+) intensity(5931.3000) index(342)
Data file IS_111911_23a.txt

Click mouse within plot area to zoom in by factor of two about that point
Or, Plot from 100 to 1700 Da Full range
Label all possible matches ○ Label matches used for scoring ◉
Show Y-axis ☐

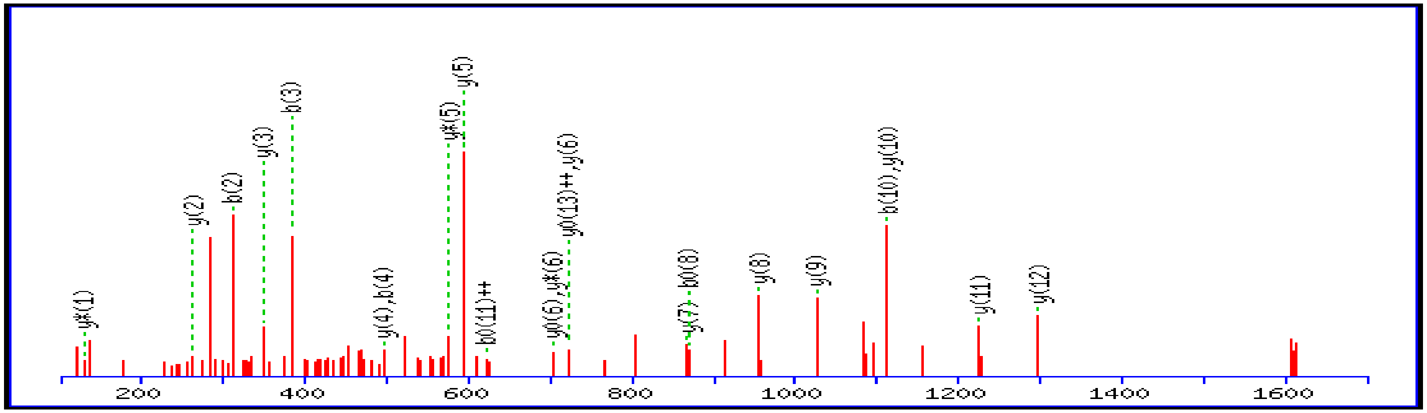

Monoisotopic mass of neutral peptide Mr(calc): 1606.7667
Fixed modifications: Carbamidomethyl (C) (apply to specified residues or termini only)
Ions Score: 81 Expect: 1.2e-06
Matches : 22/120 fragment ions using 33 most intense peaks (help)

#	b	b^{++}	b^*	b^{*++}	b^0	b^{0++}	Seq.	y	y^{++}	y^*	y^{*++}	y^0	y^{0++}	#
1	148.0757	74.5415					F							14
2	311.1390	156.0731					Y	1460.7056	730.8564	1443.6791	722.3432	1442.6951	721.8512	13
3	382.1761	191.5917					A	1297.6423	649.3248	1280.6157	640.8115	1279.6317	640.3195	12
4	495.2602	248.1337					L	1226.6052	613.8062	1209.5786	605.2930	1208.5946	604.8009	11
5	582.2922	291.6498			564.2817	282.6445	S	1113.5211	557.2642	1096.4946	548.7509	1095.5105	548.2589	10
6	653.3293	327.1683			635.3188	318.1630	A	1026.4891	513.7482	1009.4625	505.2349	1008.4785	504.7429	9
7	740.3614	370.6843			722.3508	361.6790	S	955.4520	478.2296	938.4254	469.7163	937.4414	469.2243	8
8	887.4298	444.2185			869.4192	435.2132	F	868.4199	434.7136	851.3934	426.2003	850.4094	425.7083	7
9	1016.4724	508.7398			998.4618	499.7345	E	721.3515	361.1794	704.3250	352.6661	703.3410	352.1741	6
10	1113.5251	557.2662			1095.5146	548.2609	P	592.3089	296.6581	575.2824	288.1448	574.2984	287.6528	5
11	1260.5936	630.8004			1242.5830	621.7951	F	495.2562	248.1317	478.2296	239.6185	477.2456	239.1264	4
12	1347.6256	674.3164			1329.6150	665.3111	S	348.1878	174.5975	331.1612	166.0842	330.1772	165.5922	3
13	1461.6685	731.3379	1444.6420	722.8246	1443.6579	722.3326	N	261.1557	131.0815	244.1292	122.5682			2
14							K	147.1128	74.0600	130.0863	65.5468			1

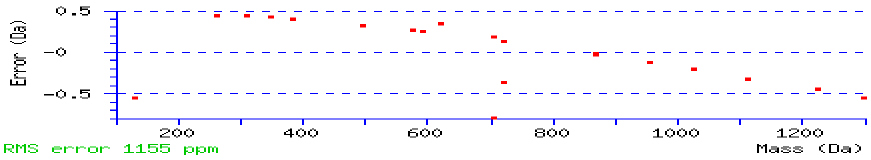

RMS error 1155 ppm

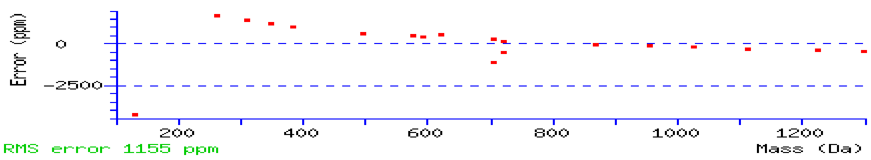

RMS error 1155 ppm

MS/MS Fragmentation of **LAALNPESNTAGLDIFAK**
Found in **CLIC1_HUMAN** in **SwissProt**, Chloride intracellular channel protein 1 OS=Homo sapiens GN=CLIC1 PE=1 SV=4

Match to Query 345: 1843.755648 from(922.885100,2+) intensity(51048.2000) index(72)
Data file IS_111911_23a.txt

Click mouse within plot area to zoom in by factor of two about that point
Or, Plot from 100 to 1500 Da Full range
Label all possible matches ○ Label matches used for scoring ◉
Show Y-axis ☐

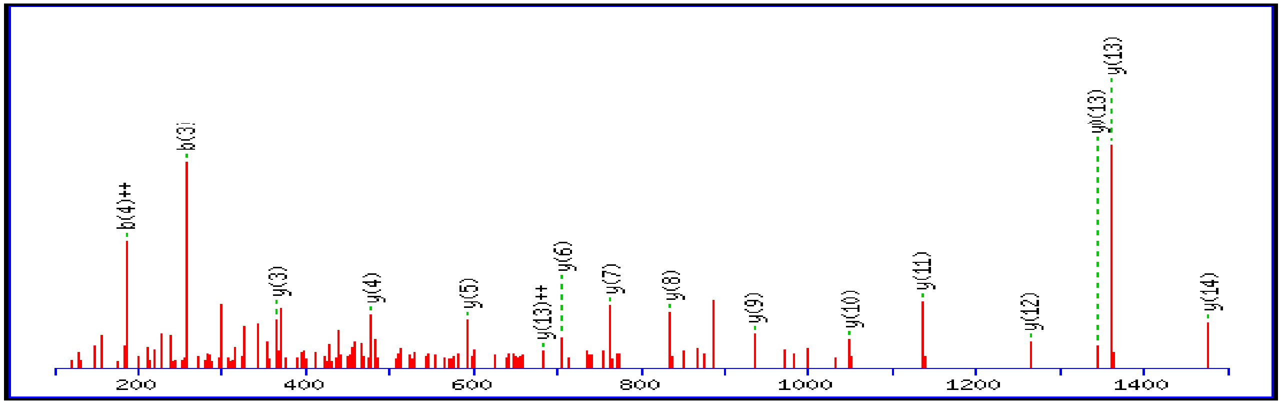


```
Monoisotopic mass of neutral peptide Mr(calc): 1843.9679
Fixed modifications: Carbamidomethyl (C) (apply to specified residues or termini only)
Ions Score: 81  Expect: 1.1e-06
Matches : 17/176 fragment ions using 27 most intense peaks   (help)
```

#	b	b^{++}	b^*	b^{*++}	b^0	b^{0++}	Seq.	y	y^{++}	y^*	y^{*++}	y^0	y^{0++}	#
1	114.0913	57.5493					L							18
2	185.1285	93.0679					A	1731.8912	866.4492	1714.8646	857.9360	1713.8806	857.4440	17
3	256.1656	128.5864					A	1660.8541	830.9307	1643.8275	822.4174	1642.8435	821.9254	16
4	369.2496	185.1285					L	1589.8170	795.4121	1572.7904	786.8988	1571.8064	786.4068	15
5	483.2926	242.1499	466.2660	233.6366			N	*1476.7329*	738.8701	1459.7063	730.3568	1458.7223	729.8648	14
6	580.3453	290.6763	563.3188	282.1630			P	*1362.6900*	681.8486	1345.6634	673.3353	1344.6794	672.8433	13
7	709.3879	355.1976	692.3614	346.6843	691.3774	346.1923	E	*1265.6372*	633.3222	1248.6107	624.8090	1247.6266	624.3170	12
8	796.4199	398.7136	779.3934	390.2003	778.4094	389.7083	S	*1136.5946*	568.8009	1119.5681	560.2877	1118.5840	559.7957	11
9	910.4629	455.7351	893.4363	447.2218	892.4523	446.7298	N	*1049.5626*	525.2849	1032.5360	516.7717	1031.5520	516.2796	10
10	1011.5105	506.2589	994.4840	497.7456	993.5000	497.2536	T	*935.5197*	468.2635	918.4931	459.7502	917.5091	459.2582	9
11	1082.5477	541.7775	1065.5211	533.2642	1064.5371	532.7722	A	*834.4720*	417.7396	817.4454	409.2264	816.4614	408.7343	8
12	1139.5691	570.2882	1122.5426	561.7749	1121.5586	561.2829	G	*763.4349*	382.2211	746.4083	373.7078	745.4243	373.2158	7
13	1252.6532	626.8302	1235.6266	618.3170	1234.6426	617.8250	L	*706.4134*	353.7103	689.3869	345.1971	688.4028	344.7051	6
14	1367.6801	684.3437	1350.6536	675.8304	1349.6696	675.3384	D	*593.3293*	297.1683	576.3028	288.6550	575.3188	288.1630	5
15	1480.7642	740.8857	1463.7376	732.3725	1462.7536	731.8805	I	*478.3024*	239.6548	461.2758	231.1416			4
16	1627.8326	814.4199	1610.8061	805.9067	1609.8220	805.4147	F	*365.2183*	183.1128	348.1918	174.5995			3
17	1698.8697	849.9385	1681.8432	841.4252	1680.8592	840.9332	A	218.1499	109.5786	201.1234	101.0653			2
18							K	147.1128	74.0600	130.0863	65.5468			1

MS/MS Fragmentation of VLEQLTGQTPVFSK
Found in RL11_HUMAN in SwissProt, 60S ribosomal protein L11 OS=Homo sapiens GN=RPL11 PE=1 SV=

Match to Query 286: 1545.965448 from(773.990000,2+) intensity(6716.6000) index(44)
Data file IS_111911_23a.txt

Click mouse within plot area to zoom in by factor of two about that point
Or, Plot from 100 to 1600 Da Full range
Label all possible matches ○ Label matches used for scoring ◉
Show Y-axis ☐

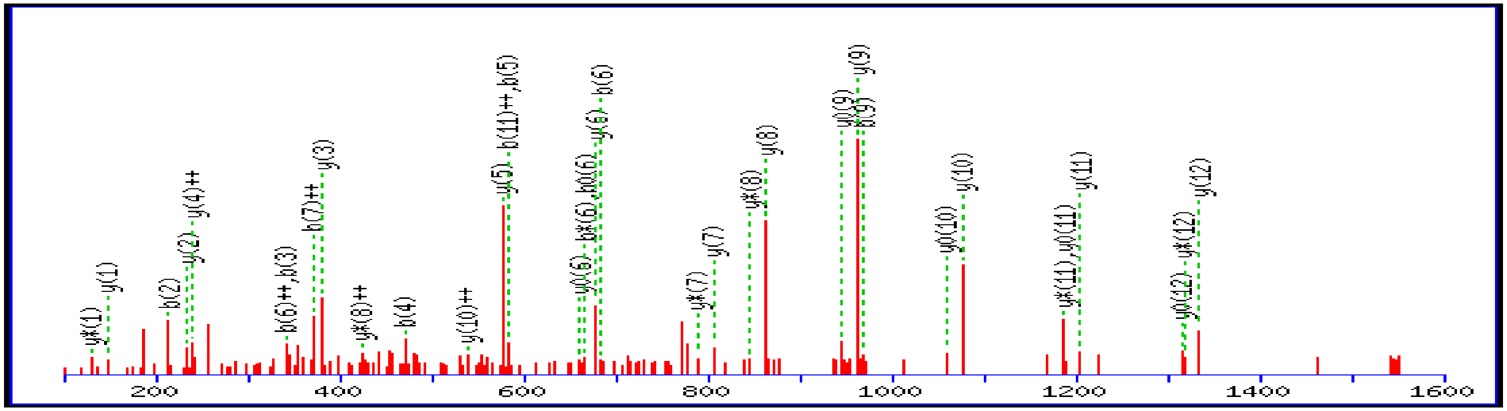

Monoisotopic mass of neutral peptide Mr(calc): 1545.8403
Fixed modifications: Carbamidomethyl (C) (apply to specified residues or termini only)
Ions Score: 76 Expect: 4.4e-06
Matches : 35/144 fragment ions using 51 most intense peaks (help)

#	b	b^{++}	b^{*}	b^{*++}	b^{0}	b^{0++}	Seq.	y	y^{++}	y^{*}	y^{*++}	y^{0}	y^{0++}	#
1	100.0757	50.5415					V							14
2	213.1598	107.0835					L	1447.7791	724.3932	1430.7526	715.8799	1429.7686	715.3879	13
3	342.2023	171.6048			324.1918	162.5995	E	1334.6951	667.8512	1317.6685	659.3379	1316.6845	658.8459	12
4	470.2609	235.6341	453.2344	227.1208	452.2504	226.6288	Q	1205.6525	603.3299	1188.6259	594.8166	1187.6419	594.3246	11
5	583.3450	292.1761	566.3184	283.6629	565.3344	283.1709	L	1077.5939	539.3006	1060.5673	530.7873	1059.5833	530.2953	10
6	684.3927	342.7000	667.3661	334.1867	666.3821	333.6947	T	964.5098	482.7586	947.4833	474.2453	946.4993	473.7533	9
7	741.4141	371.2107	724.3876	362.6974	723.4036	362.2054	G	863.4621	432.2347	846.4356	423.7214	845.4516	423.2294	8
8	869.4727	435.2400	852.4462	426.7267	851.4621	426.2347	Q	806.4407	403.7240	789.4141	395.2107	788.4301	394.7187	7
9	970.5204	485.7638	953.4938	477.2506	952.5098	476.7586	T	678.3821	339.6947	661.3556	331.1814	660.3715	330.6894	6
10	1067.5732	534.2902	1050.5466	525.7769	1049.5626	525.2849	P	577.3344	289.1709	560.3079	280.6576	559.3239	280.1656	5
11	1166.6416	583.8244	1149.6150	575.3111	1148.6310	574.8191	V	480.2817	240.6445	463.2551	232.1312	462.2711	231.6392	4
12	1313.7100	657.3586	1296.6834	648.8454	1295.6994	648.3533	F	381.2132	191.1103	364.1867	182.5970	363.2027	182.1050	3
13	1400.7420	700.8746	1383.7155	692.3614	1382.7314	691.8694	S	234.1448	117.5761	217.1183	109.0628	216.1343	108.5708	2
14							K	147.1128	74.0600	130.0863	65.5468			1

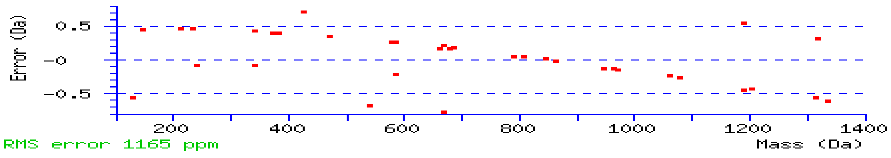

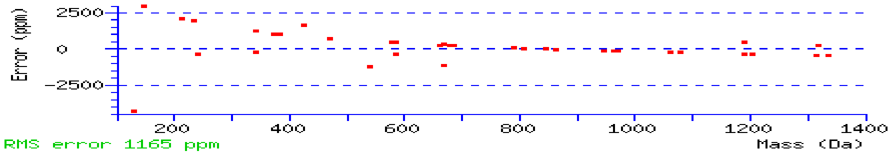

MS/MS Fragmentation of YNEDLELEDAIHTAILTLK
Found in PSA2_HUMAN in SwissProt, Proteasome subunit alpha type-2 OS=Homo sapiens GN=PSMA2 PE=1 SV=2

Match to Query 412: 2200.432572 from(734.484800,3+) intensity(14457.1000) index(399)
Data file IS_111911_23a.txt

Click mouse within plot area to zoom in by factor of two about that point
Or, Plot from 0 to 2400 Da Full range
Label all possible matches ○ Label matches used for scoring ◉
Show Y-axis ☐

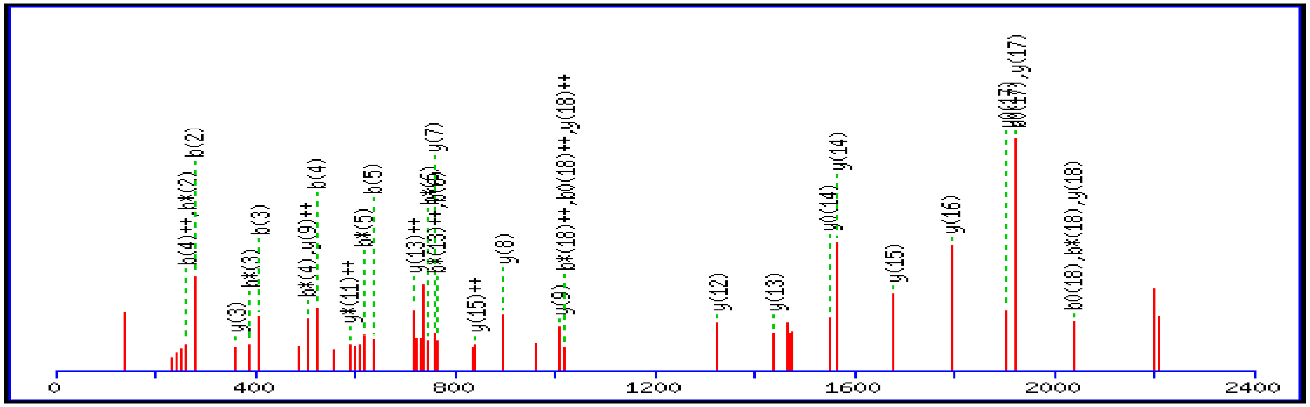

Monoisotopic mass of neutral peptide Mr(calc): 2200.1263
Fixed modifications: Carbamidomethyl (C) (apply to specified residues or termini only)
Ions Score: 76 Expect: 2.6e-06
Matches : 35/206 fragment ions using 41 most intense peaks (help)

#	b	b++	b*	b*++	b0	b0++	Seq.	y	y++	y*	y*++	y0	y0++	#
1	164.0706	82.5389					Y							19
2	**278.1135**	139.5604	261.0870	131.0471			N	***2038.0703***	1019.5388	2021.0437	1011.0255	2020.0597	1010.5335	18
3	**407.1561**	204.0817	390.1296	195.5684	389.1456	195.0764	E	***1924.0274***	962.5173	1907.0008	954.0040	1906.0168	953.5120	17
4	**522.1831**	261.5952	505.1565	253.0819	504.1725	252.5899	D	***1794.9848***	897.9960	1777.9582	889.4827	1776.9742	888.9907	16
5	**635.2671**	318.1372	618.2406	309.6239	617.2566	309.1319	L	***1679.9578***	840.4825	1662.9313	831.9693	1661.9472	831.4773	15
6	**764.3097**	382.6585	747.2832	374.1452	746.2992	373.6532	E	***1566.8738***	783.9405	1549.8472	775.4272	1548.8632	774.9352	14
7	877.3938	439.2005	860.3672	430.6873	859.3832	430.1953	L	***1437.8312***	719.4192	1420.8046	710.9059	1419.8206	710.4139	13
8	1006.4364	503.7218	989.4098	495.2086	988.4258	494.7165	E	***1324.7471***	662.8772	1307.7205	654.3639	1306.7365	653.8719	12
9	1121.4633	561.2353	1104.4368	552.7220	1103.4528	552.2300	D	1195.7045	598.3559	1178.6780	589.8426	1177.6939	589.3506	11
10	1192.5004	596.7539	1175.4739	588.2406	1174.4899	587.7486	A	1080.6776	540.8424	1063.6510	532.3291	1062.6670	531.8371	10
11	1305.5845	653.2959	1288.5580	644.7826	1287.5739	644.2906	I	***1009.6404***	505.3239	992.6139	496.8106	991.6299	496.3186	9
12	1442.6434	721.8253	1425.6169	713.3121	1424.6328	712.8201	H	***896.5564***	448.7818	879.5298	440.2686	878.5458	439.7765	8
13	1543.6911	772.3492	1526.6645	763.8359	1525.6805	763.3439	T	***759.4975***	380.2524	742.4709	371.7391	741.4869	371.2471	7
14	1614.7282	807.8677	1597.7017	799.3545	1596.7176	798.8625	A	658.4498	329.7285	641.4232	321.2153	640.4392	320.7233	6
15	1727.8123	864.4098	1710.7857	855.8965	1709.8017	855.4045	I	587.4127	294.2100	570.3861	285.6967	569.4021	285.2047	5
16	1840.8963	920.9518	1823.8698	912.4385	1822.8858	911.9465	L	474.3286	237.6679	457.3021	229.1547	456.3180	228.6627	4
17	1941.9440	971.4756	1924.9175	962.9624	1923.9334	962.4704	T	***361.2445***	181.1259	344.2180	172.6126	343.2340	172.1206	3
18	2055.0281	1028.0177	2038.0015	1019.5044	2037.0175	1019.0124	L	260.1969	130.6021	243.1703	122.0888			2
19							K	147.1128	74.0600	130.0863	65.5468			1

MS/MS Fragmentation of **TQSSLVPALTDFVR**
Found in **CUTA_HUMAN** in **SwissProt**, Protein CutA OS=Homo sapiens GN=CUTA PE=1 SV=2

Match to Query 281: 1532.966248 from(767.490400,2+) intensity(8139.9000) index(354)
Data file IS_111911_23a.txt

Click mouse within plot area to zoom in by factor of two about that point
Or, Plot from 100 to 1600 Da Full range
Label all possible matches ○ Label matches used for scoring ◉
Show Y-axis ☐

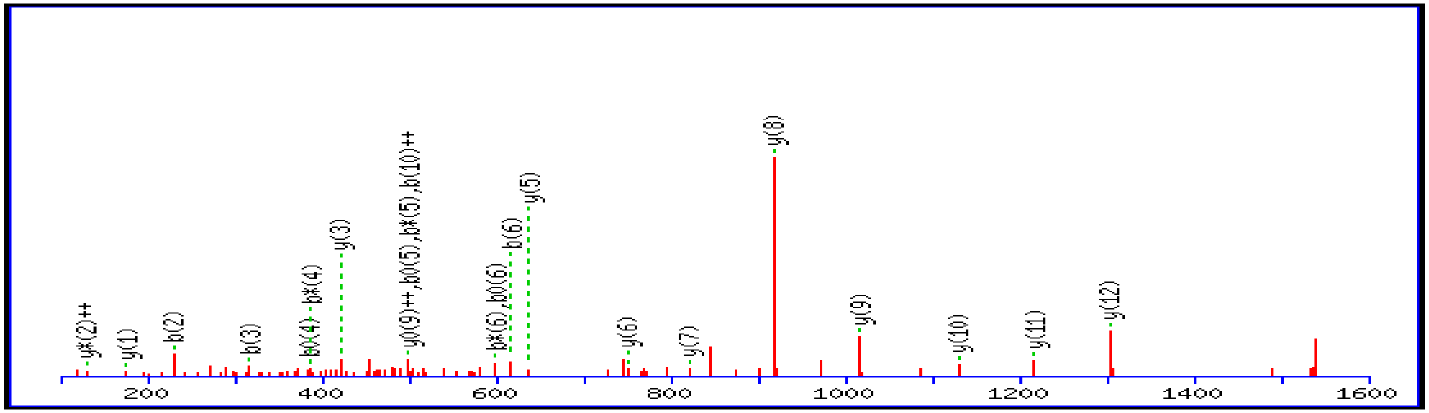


```
Monoisotopic mass of neutral peptide Mr(calc): 1532.8199
Fixed modifications: Carbamidomethyl (C) (apply to specified residues or termini only)
Ions Score: 72  Expect: 1.2e-05
Matches : 22/148 fragment ions using 32 most intense peaks  (help)
```

#	b	b^{++}	b^{*}	b^{*++}	b^{0}	b^{0++}	Seq.	y	y^{++}	y^{*}	y^{*++}	y^{0}	y^{0++}	#
1	102.0550	51.5311			84.0444	42.5258	T							14
2	**230.1135**	115.5604	213.0870	107.0471	212.1030	106.5551	Q	1432.7795	716.8934	1415.7529	708.3801	1414.7689	707.8881	13
3	**317.1456**	159.0764	300.1190	150.5631	299.1350	150.0711	S	***1304.7209***	652.8641	1287.6943	644.3508	1286.7103	643.8588	12
4	404.1776	202.5924	387.1510	194.0792	386.1670	193.5871	S	***1217.6889***	609.3481	1200.6623	600.8348	1199.6783	600.3428	11
5	517.2617	259.1345	500.2351	250.6212	499.2511	250.1292	L	***1130.6568***	565.8320	1113.6303	557.3188	1112.6463	556.8268	10
6	**616.3301**	308.6687	599.3035	300.1554	598.3195	299.6634	V	***1017.5728***	509.2900	1000.5462	500.7767	999.5622	500.2847	9
7	713.3828	357.1951	696.3563	348.6818	695.3723	348.1898	P	***918.5043***	459.7558	901.4778	451.2425	900.4938	450.7505	8
8	784.4199	392.7136	767.3934	384.2003	766.4094	383.7083	A	***821.4516***	411.2294	804.4250	402.7162	803.4410	402.2241	7
9	897.5040	449.2556	880.4775	440.7424	879.4934	440.2504	L	***750.4145***	375.7109	733.3879	367.1976	732.4039	366.7056	6
10	998.5517	499.7795	981.5251	491.2662	980.5411	490.7742	T	***637.3304***	319.1688	620.3039	310.6556	619.3198	310.1636	5
11	1113.5786	557.2930	1096.5521	548.7797	1095.5681	548.2877	D	536.2827	268.6450	519.2562	260.1317	518.2722	259.6397	4
12	1260.6470	630.8272	1243.6205	622.3139	1242.6365	621.8219	F	***421.2558***	211.1315	404.2292	202.6183			3
13	1359.7155	680.3614	1342.6889	671.8481	1341.7049	671.3561	V	274.1874	137.5973	257.1608	129.0840			2
14							R	***175.1190***	88.0631	158.0924	79.5498			1

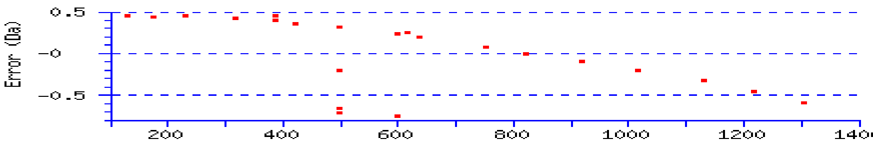

RMS error 1246 ppm

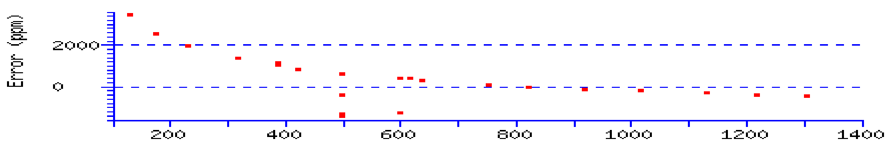

RMS error 1246 ppm

MS/MS Fragmentation of SLEEAIQFINQR
Found in AL3A1_HUMAN in SwissProt, Aldehyde dehydrogenase, dimeric NADP-preferring OS=Homo sapiens GN=ALDH3A1 PE=1 SV=3

Match to Query 264: 1446.992048 from(724.503300,2+) intensity(5194.4000) index(335)
Data file IS_111911_23a.txt

Click mouse within plot area to zoom in by factor of two about that point
Or, Plot from 100 to 1500 Da Full range
Label all possible matches ○ Label matches used for scoring ◉
Show Y-axis ☐

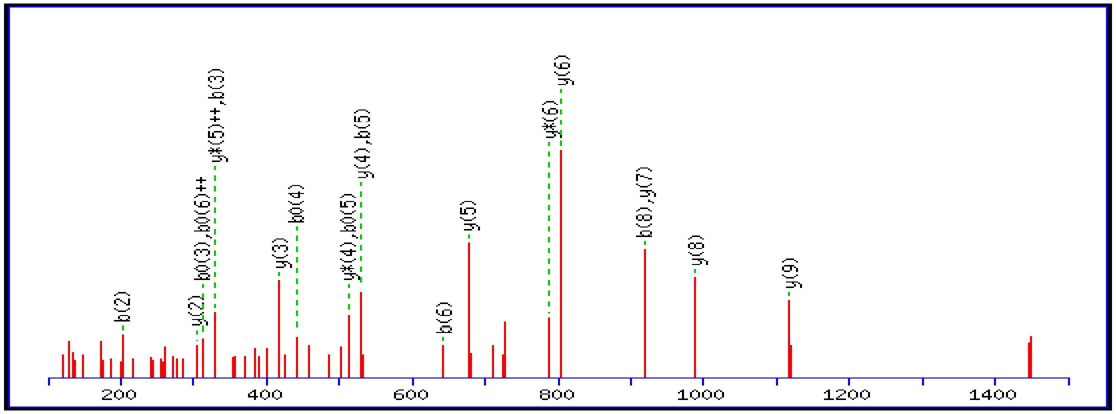


```
Monoisotopic mass of neutral peptide Mr(calc): 1446.7467
Fixed modifications: Carbamidomethyl (C) (apply to specified residues or termini only)
Ions Score: 67  Expect: 3.4e-05
Matches : 20/104 fragment ions using 20 most intense peaks  (help)
```

#	b	b++	b*	b*++	b0	b0++	Seq.	y	y++	y*	y*++	y0	y0++	#
1	88.0393	44.5233			70.0287	35.5180	S							12
2	201.1234	101.0653			183.1128	92.0600	L	1360.7219	680.8646	1343.6954	672.3513	1342.7114	671.8593	11
3	330.1660	165.5866			312.1554	156.5813	E	1247.6379	624.3226	1230.6113	615.8093	1229.6273	615.3173	10
4	459.2086	230.1079			441.1980	221.1026	E	1118.5953	559.8013	1101.5687	551.2880	1100.5847	550.7960	9
5	530.2457	265.6265			512.2351	256.6212	A	989.5527	495.2800	972.5261	486.7667			8
6	643.3297	322.1685			625.3192	313.1632	I	918.5156	459.7614	901.4890	451.2482			7
7	771.3883	386.1978	754.3618	377.6845	753.3777	377.1925	Q	805.4315	403.2194	788.4050	394.7061			6
8	918.4567	459.7320	901.4302	451.2187	900.4462	450.7267	F	677.3729	339.1901	660.3464	330.6768			5
9	1031.5408	516.2740	1014.5142	507.7608	1013.5302	507.2687	I	530.3045	265.6559	513.2780	257.1426			4
10	1145.5837	573.2955	1128.5572	564.7822	1127.5731	564.2902	N	417.2205	209.1139	400.1939	200.6006			3
11	1273.6423	637.3248	1256.6157	628.8115	1255.6317	628.3195	Q	303.1775	152.0924	286.1510	143.5791			2
12							R	175.1190	88.0631	158.0924	79.5498			1

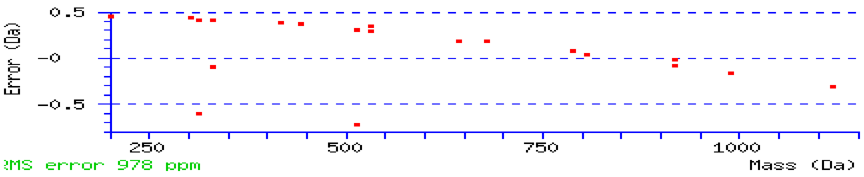

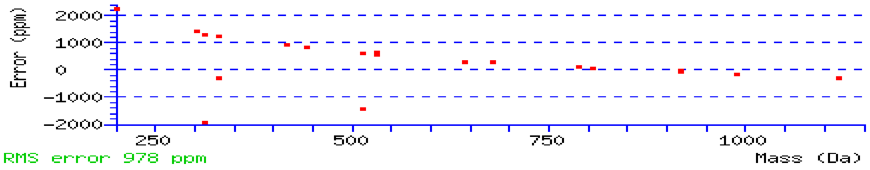

MS/MS Fragmentation of **VPSLVGSFIR**
Found in **GSTO1_HUMAN** in **SwissProt**, Glutathione S-transferase omega-1 OS=Homo sapiens GN=GSTO1 PE=1 SV=2

Match to Query 124: 1074.207848 from(538.111200,2+) intensity(11276.6000) index(64)
Data file IS_111911_23a.txt

Click mouse within plot area to zoom in by factor of two about that point
Or, Plot from 100 to 1000 Da Full range
Label all possible matches ○ Label matches used for scoring ◉
Show Y-axis ☐

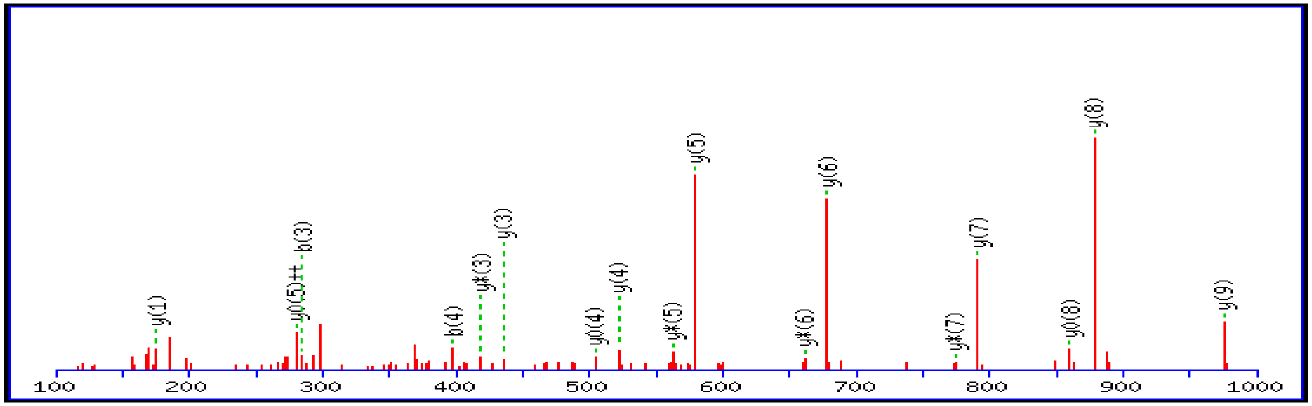

Monoisotopic mass of neutral peptide Mr(calc): 1073.6233
Fixed modifications: Carbamidomethyl (C) (apply to specified residues or termini only)
Ions Score: 65 Expect: 6.4e-05
Matches : 17/80 fragment ions using 26 most intense peaks (help)

#	b	b++	b0	b0++	Seq.	y	y++	y*	y*++	y0	y0++	#
1	100.0757	50.5415			V							10
2	197.1285	99.0679			P	975.5622	488.2847	958.5356	479.7715	957.5516	479.2795	9
3	284.1605	142.5839	266.1499	133.5786	S	878.5094	439.7584	861.4829	431.2451	860.4989	430.7531	8
4	397.2445	199.1259	379.2340	190.1206	L	791.4774	396.2423	774.4509	387.7291	773.4668	387.2371	7
5	496.3130	248.6601	478.3024	239.6548	V	678.3933	339.7003	661.3668	331.1870	660.3828	330.6950	6
6	553.3344	277.1709	535.3239	268.1656	G	579.3249	290.1661	562.2984	281.6528	561.3144	281.1608	5
7	640.3665	320.6869	622.3559	311.6816	S	522.3035	261.6554	505.2769	253.1421	504.2929	252.6501	4
8	787.4349	394.2211	769.4243	385.2158	F	435.2714	218.1394	418.2449	209.6261			3
9	900.5189	450.7631	882.5084	441.7578	I	288.2030	144.6051	271.1765	136.0919			2
10					R	175.1190	88.0631	158.0924	79.5498			1

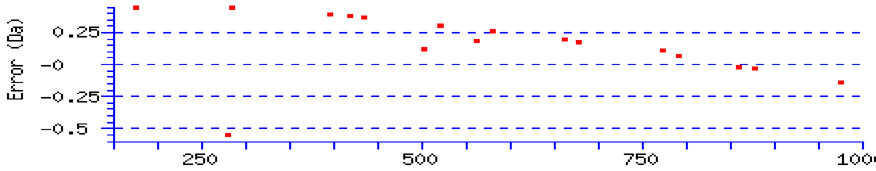

RMS error 976 ppm

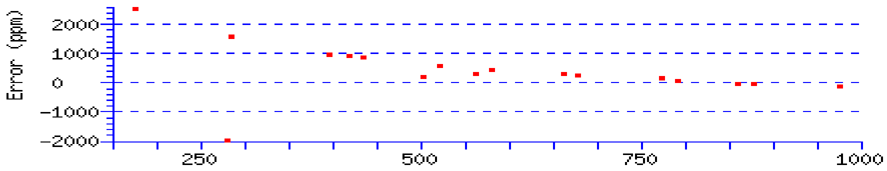

RMS error 976 ppm

MS/MS Fragmentation of **TTPDVIFVFGFR**
Found in **RS24_HUMAN** in **SwissProt**, 40S ribosomal protein S24 OS=Homo sapiens GN=RPS24 PE=1 SV=1

Match to Query 251: 1398.023448 from(700.019000,2+) intensity(9698.5000) index(384)
Data file IS_111911_23a.txt

Click mouse within plot area to zoom in by factor of two about that point
Or, Plot from 100 to 1500 Da Full range
Label all possible matches ○ Label matches used for scoring ◉
Show Y-axis ☐

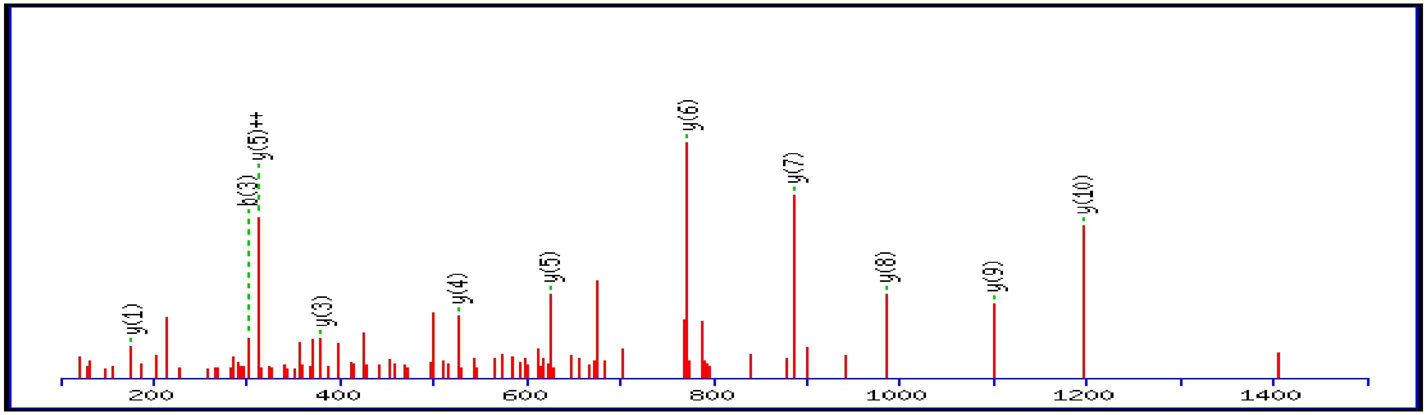

Monoisotopic mass of neutral peptide Mr(calc): 1397.7344
Fixed modifications: Carbamidomethyl (C) (apply to specified residues or termini only)
Ions Score: 60 Expect: 0.00014
Matches : 11/94 fragment ions using 22 most intense peaks (help)

#	b	b++	b0	b0++	Seq.	y	y++	y*	y*++	y0	y0++	#
1	102.0550	51.5311	84.0444	42.5258	T							12
2	203.1026	102.0550	185.0921	93.0497	T	1297.6939	649.3506	1280.6674	640.8373	1279.6834	640.3453	11
3	300.1554	150.5813	282.1448	141.5761	P	1196.6463	598.8268	1179.6197	590.3135	1178.6357	589.8215	10
4	415.1823	208.0948	397.1718	199.0895	D	1099.5935	550.3004	1082.5669	541.7871	1081.5829	541.2951	9
5	514.2508	257.6290	496.2402	248.6237	V	984.5665	492.7869	967.5400	484.2736			8
6	627.3348	314.1710	609.3243	305.1658	I	885.4981	443.2527	868.4716	434.7394			7
7	774.4032	387.7053	756.3927	378.7000	F	772.4141	386.7107	755.3875	378.1974			6
8	873.4716	437.2395	855.4611	428.2342	V	625.3457	313.1765	608.3191	304.6632			5
9	1020.5401	510.7737	1002.5295	501.7684	F	526.2772	263.6423	509.2507	255.1290			4
10	1077.5615	539.2844	1059.5510	530.2791	G	379.2088	190.1081	362.1823	181.5948			3
11	1224.6299	612.8186	1206.6194	603.8133	F	322.1874	161.5973	305.1608	153.0840			2
12					R	175.1190	88.0631	158.0924	79.5498			1

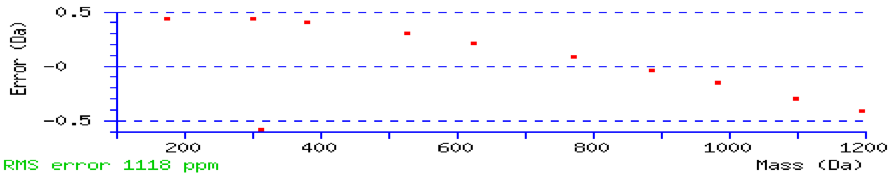

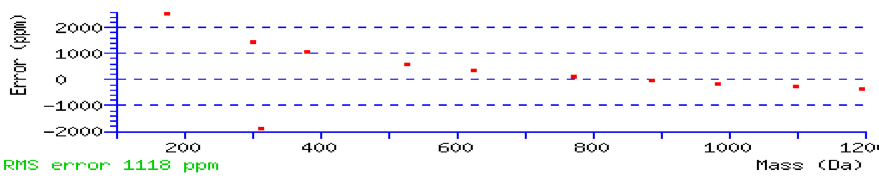

MS/MS Fragmentation of **GVDLDQLLDMSYEQLMQLYSAR**
Found in **RS15_HUMAN** in **SwissProt**, 40S ribosomal protein S15 OS=Homo sapiens GN=RPS15 PE=1 SV=2

Match to Query 435: 2619.084972 from(874.035600,3+) intensity(6674.8000) index(419)
Data file IS_111911_23a.txt

Click mouse within plot area to zoom in by factor of two about that point
Or, Plot from 100 to 2000 Da Full range
Label all possible matches ○ Label matches used for scoring ◉
Show Y-axis ☐

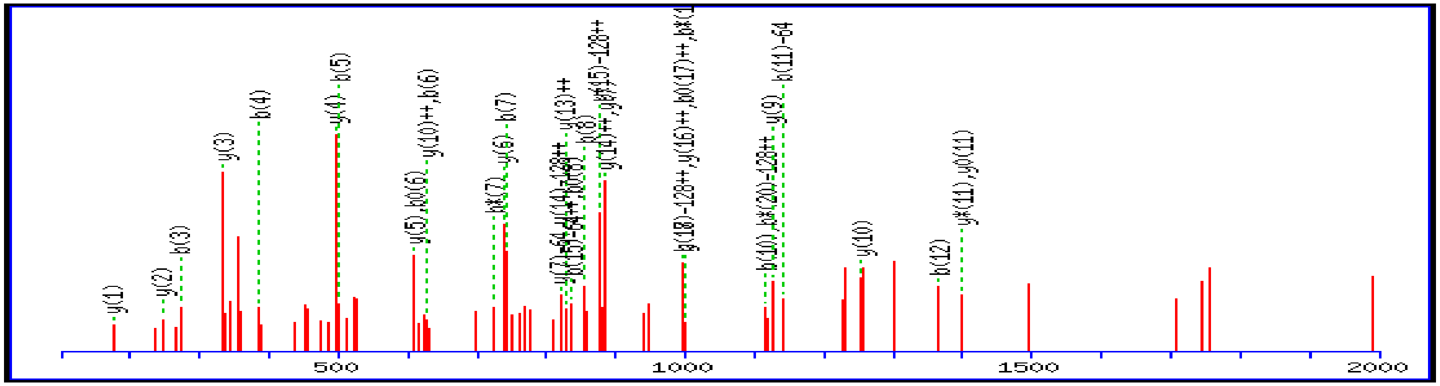


```
Monoisotopic mass of neutral peptide Mr(calc): 2619.2196
Fixed modifications: Carbamidomethyl (C) (apply to specified residues or termini only)
Variable modifications:
M10      : Oxidation (M), with neutral losses 0.0000(shown in table), 63.9983
M16       : Oxidation (M), with neutral losses 0.0000(shown in table), 63.9983
 Ions Score: 56  Expect: 0.0002
Matches : 37/396 fragment ions using 51 most intense peaks  (help)
```

#	b	b^{++}	b^{*}	b^{*++}	b^{0}	b^{0++}	Seq.	y	y^{++}	y^{*}	y^{*++}	y^{0}	y^{0++}	#
1	58.0287	29.5180					G							22
2	157.0972	79.0522					V	2563.2055	1282.1064	2546.1789	1273.5931	2545.1949	1273.1011	21
3	*272.1241*	136.5657			254.1135	127.5604	D	2464.1371	1232.5722	2447.1105	1224.0589	2446.1265	1223.5669	20
4	*385.2082*	193.1077			367.1976	184.1024	L	2349.1101	1175.0587	2332.0836	1166.5454	2331.0995	1166.0534	19
5	*500.2351*	250.6212			482.2245	241.6159	D	2236.0260	1118.5167	2218.9995	1110.0034	2218.0155	1109.5114	18
6	*628.2937*	314.6505	611.2671	306.1372	610.2831	305.6452	Q	2120.9991	1061.0032	2103.9726	1052.4899	2102.9885	1051.9979	17
7	*741.3777*	371.1925	724.3512	362.6792	723.3672	362.1872	L	1992.9405	996.9739	1975.9140	988.4606	1974.9300	987.9686	16
8	*854.4618*	427.7345	837.4353	419.2213	836.4512	418.7293	L	1879.8565	940.4319	1862.8299	931.9186	1861.8459	931.4266	15
9	969.4888	485.2480	952.4622	476.7347	951.4782	476.2427	D	1766.7724	883.8898	1749.7459	875.3766	1748.7618	874.8846	14
10	*1116.5242*	558.7657	1099.4976	550.2524	1098.5136	549.7604	M	1651.7455	826.3764	1634.7189	817.8631	1633.7349	817.3711	13
11	1203.5562	602.2817	1186.5296	593.7685	1185.5456	593.2764	S	1504.7101	752.8587	1487.6835	744.3454	1486.6995	743.8534	12
12	*1366.6195*	683.8134	1349.5930	675.3001	1348.6089	674.8081	Y	1417.6780	709.3427	1400.6515	700.8294	1399.6675	700.3374	11
13	1495.6621	748.3347	1478.6356	739.8214	1477.6515	739.3294	E	*1254.6147*	627.8110	1237.5882	619.2977	1236.6041	618.8057	10
14	1623.7207	812.3640	1606.6941	803.8507	1605.7101	803.3587	Q	*1125.5721*	563.2897	1108.5456	554.7764	1107.5615	554.2844	9
15	1736.8047	868.9060	1719.7782	860.3927	1718.7942	859.9007	L	*997.5135*	499.2604	980.4870	490.7471	979.5030	490.2551	8
16	1883.8401	942.4237	1866.8136	933.9104	1865.8296	933.4184	M	*884.4295*	442.7184	867.4029	434.2051	866.4189	433.7131	7
17	2011.8987	1006.4530	1994.8722	997.9397	1993.8882	997.4477	Q	*737.3941*	369.2007	720.3675	360.6874	719.3835	360.1954	6
18	2124.9828	1062.9950	2107.9562	1054.4818	2106.9722	1053.9897	L	*609.3355*	305.1714	592.3089	296.6581	591.3249	296.1661	5
19	2288.0461	1144.5267	2271.0196	1136.0134	2270.0356	1135.5214	Y	*496.2514*	248.6293	479.2249	240.1161	478.2409	239.6241	4
20	2375.0781	1188.0427	2358.0516	1179.5294	2357.0676	1179.0374	S	*333.1881*	167.0977	316.1615	158.5844	315.1775	158.0924	3
21	2446.1153	1223.5613	2429.0887	1215.0480	2428.1047	1214.5560	A	*246.1561*	123.5817	229.1295	115.0684			2
22							R	*175.1190*	88.0631	158.0924	79.5498			1

MS/MS Fragmentation of **LLEPVLLLGK**
Found in **RS16_HUMAN** in **SwissProt**, 40S ribosomal protein S16 OS=Homo sapiens GN=RPS16 PE=1 SV=2

Match to Query 131: 1094.277048 from(548.145800,2+) intensity(8371.5000) index(353)
Data file IS_111911_23a.txt

Click mouse within plot area to zoom in by factor of two about that point
Or, Plot from 100 to 1100 Da Full range
Label all possible matches ○ Label matches used for scoring ◉
Show Y-axis ☐

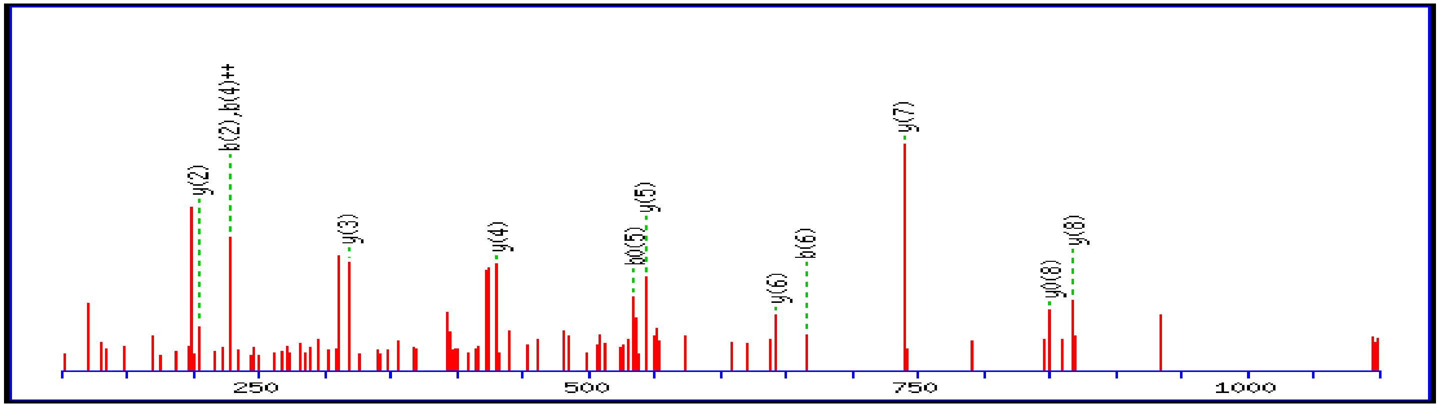

Monoisotopic mass of neutral peptide Mr(calc): 1093.7111
Fixed modifications: Carbamidomethyl (C) (apply to specified residues or termini only)
Ions Score: 54 Expect: 0.00077
Matches : 12/72 fragment ions using 18 most intense peaks (help)

#	b	b++	b0	b0++	Seq.	y	y++	y*	y*++	y0	y0++	#
1	114.0913	57.5493			L							10
2	227.1754	114.0913			L	981.6343	491.3208	964.6077	482.8075	963.6237	482.3155	9
3	356.2180	178.6126	338.2074	169.6074	E	868.5502	434.7788	851.5237	426.2655	850.5397	425.7735	8
4	453.2708	227.1390	435.2602	218.1337	P	739.5076	370.2575	722.4811	361.7442			7
5	552.3392	276.6732	534.3286	267.6679	V	642.4549	321.7311	625.4283	313.2178			6
6	665.4232	333.2153	647.4127	324.2100	L	543.3865	272.1969	526.3599	263.6836			5
7	778.5073	389.7573	760.4967	380.7520	L	430.3024	215.6548	413.2758	207.1416			4
8	891.5914	446.2993	873.5808	437.2940	L	317.2183	159.1128	300.1918	150.5995			3
9	948.6128	474.8101	930.6023	465.8048	G	204.1343	102.5708	187.1077	94.0575			2
10					K	147.1128	74.0600	130.0863	65.5468			1

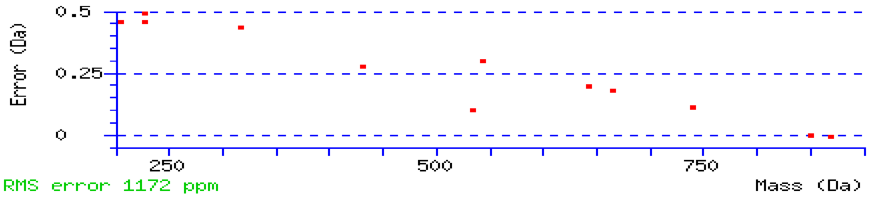

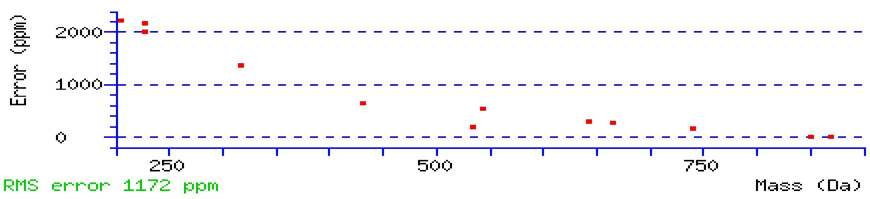

MS/MS Fragmentation of AVQQPDGLAVLGIFLK
Found in CAH2_HUMAN in SwissProt, Carbonic anhydrase 2 OS=Homo sapiens GN=CA2 PE=1 SV=2

Match to Query 315: 1667.969248 from(834.991900,2+) intensity(4688.2000) index(137)
Data file IS_111911_23a.txt

Click mouse within plot area to zoom in by factor of two about that point
Or, Plot from 100 to 1400 Da Full range
Label all possible matches ○ Label matches used for scoring ◉
Show Y-axis ☐

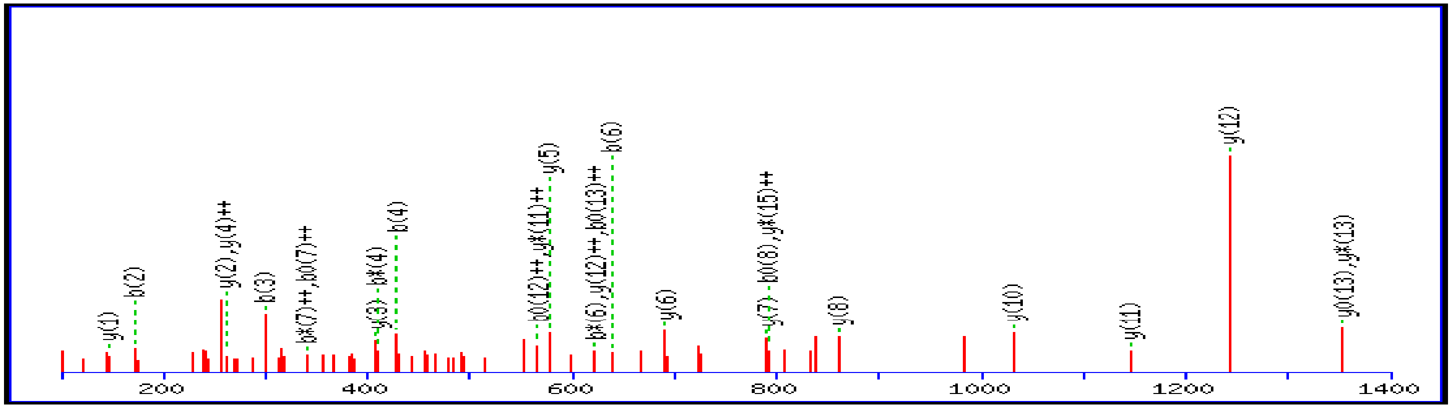

Monoisotopic mass of neutral peptide Mr(calc): 1667.9610
Fixed modifications: Carbamidomethyl (C) (apply to specified residues or termini only)
Ions Score: 50 Expect: 0.0015
Matches : 27/146 fragment ions using 48 most intense peaks (help)

#	b	b^{++}	b^{*}	b^{*++}	b^{0}	b^{0++}	Seq.	y	y^{++}	y^{*}	y^{*++}	y^{0}	y^{0++}	#
1	72.0444	36.5258					A							16
2	**171.1128**	86.0600					V	1597.9312	799.4692	1580.9047	790.9560	1579.9206	790.4640	15
3	**299.1714**	150.0893	282.1448	141.5761			Q	1498.8628	749.9350	1481.8362	741.4218	1480.8522	740.9298	14
4	**427.2300**	214.1186	410.2034	205.6053			Q	1370.8042	685.9057	1353.7777	677.3925	1352.7936	676.9005	13
5	524.2827	262.6450	507.2562	254.1317			P	*1242.7456*	621.8765	1225.7191	613.3632	1224.7351	612.8712	12
6	**639.3097**	320.1585	622.2831	311.6452	621.2991	311.1532	D	*1145.6929*	573.3501	1128.6663	564.8368	1127.6823	564.3448	11
7	696.3311	348.6692	679.3046	340.1559	678.3206	339.6639	G	*1030.6659*	515.8366	1013.6394	507.3233			10
8	809.4152	405.2112	792.3886	396.6980	791.4046	396.2060	L	973.6445	487.3259	956.6179	478.8126			9
9	880.4523	440.7298	863.4258	432.2165	862.4417	431.7245	A	*860.5604*	430.7838	843.5339	422.2706			8
10	979.5207	490.2640	962.4942	481.7507	961.5102	481.2587	V	*789.5233*	395.2653	772.4967	386.7520			7
11	1092.6048	546.8060	1075.5782	538.2928	1074.5942	537.8007	L	*690.4549*	345.7311	673.4283	337.2178			6
12	1149.6263	575.3168	1132.5997	566.8035	1131.6157	566.3115	G	*577.3708*	289.1890	560.3443	280.6758			5
13	1262.7103	631.8588	1245.6838	623.3455	1244.6997	622.8535	I	520.3493	260.6783	503.3228	252.1650			4
14	1409.7787	705.3930	1392.7522	696.8797	1391.7682	696.3877	F	*407.2653*	204.1363	390.2387	195.6230			3
15	1522.8628	761.9350	1505.8362	753.4218	1504.8522	752.9298	L	*260.1969*	130.6021	243.1703	122.0888			2
16							K	*147.1128*	74.0600	130.0863	65.5468			1

MS/MS Fragmentation of **NAYAVLYDIILK**
Found in **PSME1_HUMAN** in **SwissProt**, Proteasome activator complex subunit 1 OS=Homo sapiens GN=PSME1 PE=1 SV=1

Match to Query 249: 1395.077448 from(698.546000,2+) intensity(14325.2000) index(390)
Data file IS_111911_23a.txt

Click mouse within plot area to zoom in by factor of two about that point
Or, Plot from 100 to 1500 Da Full range
Label all possible matches ○ Label matches used for scoring ◉
Show Y-axis ☐

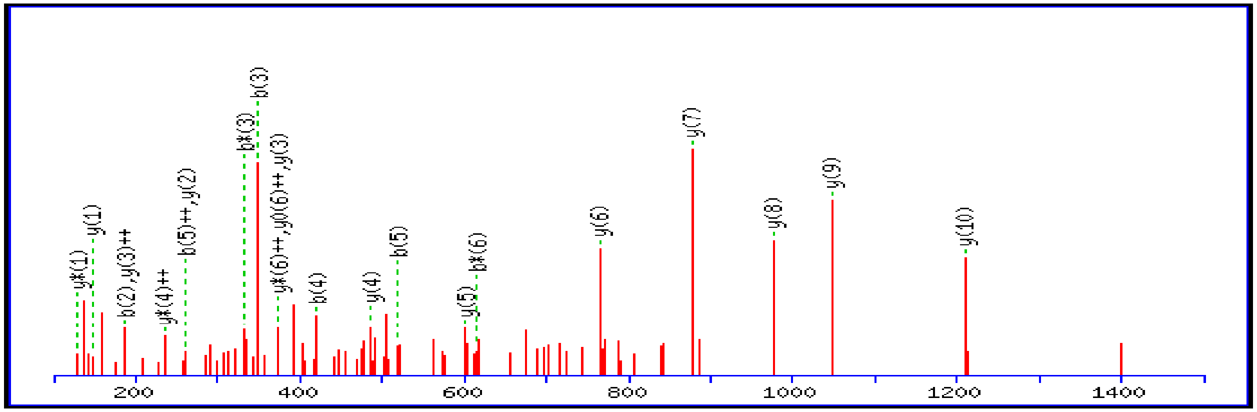


```
Monoisotopic mass of neutral peptide Mr(calc): 1394.7809
Fixed modifications: Carbamidomethyl (C) (apply to specified residues or termini only)
Ions Score: 50  Expect: 0.0015
Matches : 22/110 fragment ions using 52 most intense peaks   (help)
```

#	b	b^{++}	b^{*}	b^{*++}	b^{0}	b^{0++}	Seq.	y	y^{++}	y^{*}	y^{*++}	y^{0}	y^{0++}	#
1	115.0502	58.0287	98.0237	49.5155			N							12
2	**186.0873**	93.5473	169.0608	85.0340			A	1281.7453	641.3763	1264.7188	632.8630	1263.7347	632.3710	11
3	**349.1506**	175.0790	332.1241	166.5657			Y	*1210.7082*	605.8577	1193.6816	597.3445	1192.6976	596.8524	10
4	**420.1878**	210.5975	403.1612	202.0842			A	*1047.6449*	524.3261	1030.6183	515.8128	1029.6343	515.3208	9
5	**519.2562**	260.1317	502.2296	251.6185			V	*976.6077*	488.8075	959.5812	480.2942	958.5972	479.8022	8
6	632.3402	316.6738	615.3137	308.1605			L	*877.5393*	439.2733	860.5128	430.7600	859.5288	430.2680	7
7	795.4036	398.2054	778.3770	389.6921			Y	*764.4553*	382.7313	747.4287	374.2180	746.4447	373.7260	6
8	910.4305	455.7189	893.4040	447.2056	892.4199	446.7136	D	*601.3919*	301.1996	584.3654	292.6863	583.3814	292.1943	5
9	1023.5146	512.2609	1006.4880	503.7477	1005.5040	503.2556	I	*486.3650*	243.6861	469.3384	235.1729			4
10	1136.5986	568.8030	1119.5721	560.2897	1118.5881	559.7977	I	*373.2809*	187.1441	356.2544	178.6308			3
11	1249.6827	625.3450	1232.6562	616.8317	1231.6721	616.3397	L	*260.1969*	130.6021	243.1703	122.0888			2
12							K	*147.1128*	74.0600	130.0863	65.5468			1

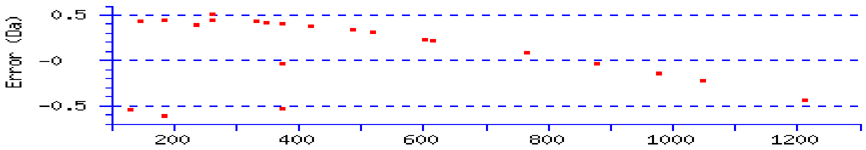

RMS error 1667 ppm

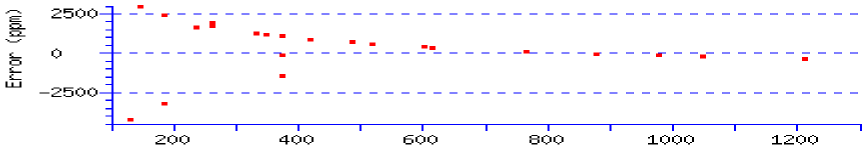

RMS error 1667 ppm

MS/MS Fragmentation of **TDELKNDLLK**

Found in **STALP_HUMAN** in **SwissProt**, AMSH-like protease OS=Homo sapiens GN=STAMBPL1 PE=1 SV=2

Match to Query 153: 1189.157848 from(595.586200,2+) intensity(36096.4000) index(336)

Data file IS_111911_23a.txt

Click mouse within plot area to zoom in by factor of two about that point

Or, Plot from 100 to 1200 Da Full range

Label all possible matches ○ Label matches used for scoring ◉

Show Y-axis ☐

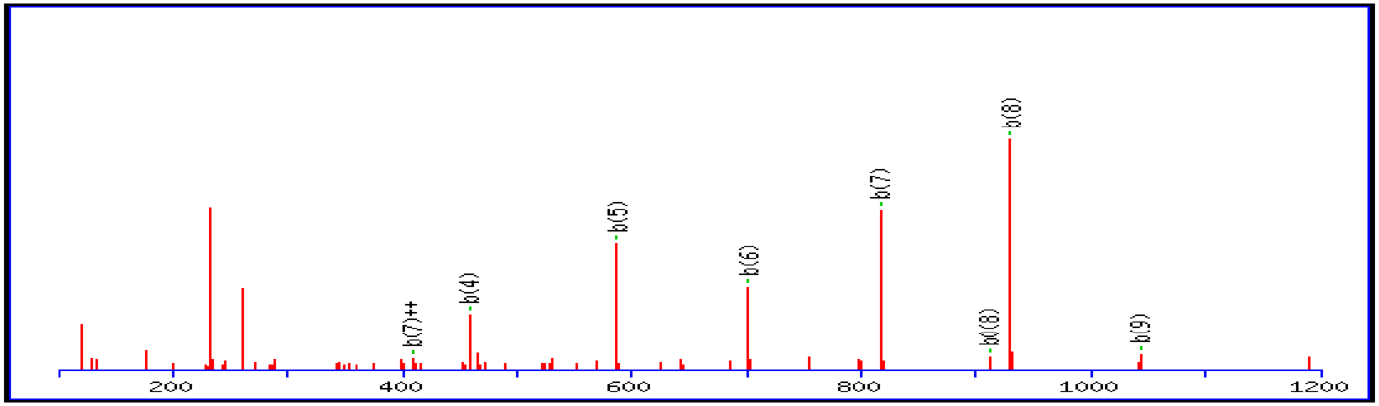

Monoisotopic mass of neutral peptide Mr(calc): 1187.6397

Fixed modifications: Carbamidomethyl (C) (apply to specified residues or termini only)

Ions Score: 48 Expect: 0.0028

Matches : 8/94 fragment ions using 10 most intense peaks (help)

#	b	b^{++}	b^*	b^{*++}	b^0	b^{0++}	Seq.	y	y^{++}	y^*	y^{*++}	y^0	y^{0++}	#
1	102.0550	51.5311			84.0444	42.5258	T							10
2	217.0819	109.0446			199.0713	100.0393	D	1087.5994	544.3033	1070.5728	535.7900	1069.5888	535.2980	9
3	346.1245	173.5659			328.1139	164.5606	E	972.5724	486.7898	955.5459	478.2766	954.5619	477.7846	8
4	*459.2086*	230.1079			441.1980	221.1026	L	843.5298	422.2686	826.5033	413.7553	825.5193	413.2633	7
5	*587.3035*	294.1554	570.2770	285.6421	569.2930	285.1501	K	730.4458	365.7265	713.4192	357.2132	712.4352	356.7212	6
6	*701.3464*	351.1769	684.3199	342.6636	683.3359	342.1716	N	602.3508	301.6790	585.3243	293.1658	584.3402	292.6738	5
7	*816.3734*	408.6903	799.3468	400.1771	798.3628	399.6850	D	488.3079	244.6576	471.2813	236.1443	470.2973	235.6523	4
8	*929.4575*	465.2324	912.4309	456.7191	911.4469	456.2271	L	373.2809	187.1441	356.2544	178.6308			3
9	*1042.5415*	521.7744	1025.5150	513.2611	1024.5310	512.7691	L	260.1969	130.6021	243.1703	122.0888			2
10							K	147.1128	74.0600	130.0863	65.5468			1

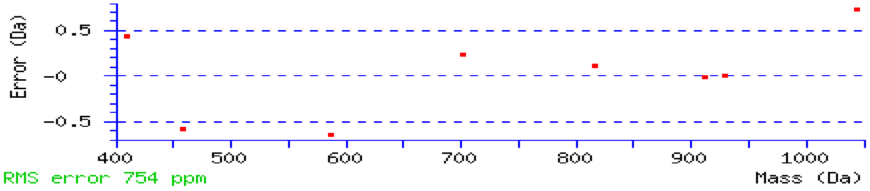

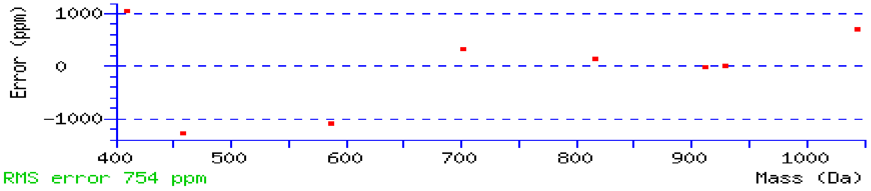

MS/MS Fragmentation of VETGVLKPGMVVTFAPVNVTTEVK
Found in EF1A1_HUMAN in SwissProt, Elongation factor 1-alpha 1 OS=Homo sapiens GN=EEF1A1 PE=1 SV=1

Match to Query 431: 2530.341372 from(844.454400,3+) intensity(10022.0000) index(329)
Data file IS_111911_23a.txt

Click mouse within plot area to zoom in by factor of two about that point
Or, Plot from 0 to 2600 Da Full range
Label all possible matches ○ Label matches used for scoring ◉
Show Y-axis ☐

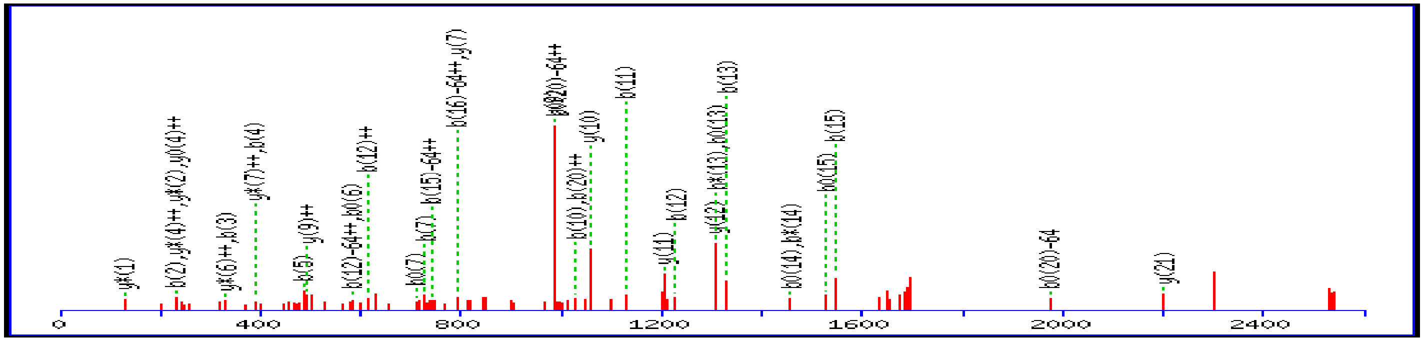

Monoisotopic mass of neutral peptide Mr(calc): 2530.3717
Fixed modifications: Carbamidomethyl (C) (apply to specified residues or termini only)
Variable modifications:
M10 : Oxidation (M), with neutral losses 0.0000(shown in table), 63.9983

Ions Score: 47 Expect: 0.002
Matches : 37/396 fragment ions using 47 most intense peaks (help)

#	b	b++	b*	b*++	b0	b0++	Seq.	y	y++	y*	y*++	y0	y0++	#
1	100.0757	50.5415					V							24
2	229.1183	115.0628			211.1077	106.0575	E	2432.3105	1216.6589	2415.2840	1208.1456	2414.3000	1207.6536	23
3	330.1660	165.5866			312.1554	156.5813	T	2303.2679	1152.1376	2286.2414	1143.6243	2285.2574	1143.1323	22
4	387.1874	194.0974			369.1769	185.0921	G	2202.2203	1101.6138	2185.1937	1093.1005	2184.2097	1092.6085	21
5	486.2558	243.6316			468.2453	234.6263	V	2145.1988	1073.1030	2128.1722	1064.5898	2127.1882	1064.0978	20
6	599.3399	300.1736			581.3293	291.1683	L	2046.1304	1023.5688	2029.1038	1015.0556	2028.1198	1014.5635	19
7	727.4349	364.2211	710.4083	355.7078	709.4243	355.2158	K	1933.0463	967.0268	1916.0198	958.5135	1915.0357	958.0215	18
8	824.4876	412.7475	807.4611	404.2342	806.4771	403.7422	P	1804.9514	902.9793	1787.9248	894.4660	1786.9408	893.9740	17
9	881.5091	441.2582	864.4825	432.7449	863.4985	432.2529	G	1707.8986	854.4529	1690.8720	845.9397	1689.8880	845.4476	16
10	1028.5445	514.7759	1011.5179	506.2626	1010.5339	505.7706	M	1650.8771	825.9422	1633.8506	817.4289	1632.8666	816.9369	15
11	1127.6129	564.3101	1110.5864	555.7968	1109.6023	555.3048	V	1503.8417	752.4245	1486.8152	743.9112	1485.8312	743.4192	14
12	1226.6813	613.8443	1209.6548	605.3310	1208.6708	604.8390	V	1404.7733	702.8903	1387.7468	694.3770	1386.7627	693.8850	13
13	1327.7290	664.3681	1310.7025	655.8549	1309.7184	655.3629	T	1305.7049	653.3561	1288.6783	644.8428	1287.6943	644.3508	12
14	1474.7974	737.9023	1457.7709	729.3891	1456.7869	728.8971	F	1204.6572	602.8322	1187.6307	594.3190	1186.6467	593.8270	11
15	1545.8345	773.4209	1528.8080	764.9076	1527.8240	764.4156	A	1057.5888	529.2980	1040.5623	520.7848	1039.5782	520.2928	10
16	1642.8873	821.9473	1625.8607	813.4340	1624.8767	812.9420	P	986.5517	493.7795	969.5251	485.2662	968.5411	484.7742	9
17	1741.9557	871.4815	1724.9292	862.9682	1723.9451	862.4762	V	889.4989	445.2531	872.4724	436.7398	871.4884	436.2478	8
18	1855.9986	928.5030	1838.9721	919.9897	1837.9881	919.4977	N	790.4305	395.7189	773.4040	387.2056	772.4199	386.7136	7
19	1955.0670	978.0372	1938.0405	969.5239	1937.0565	969.0319	V	676.3876	338.6974	659.3610	330.1842	658.3770	329.6921	6
20	2056.1147	1028.5610	2039.0882	1020.0477	2038.1042	1019.5557	T	577.3192	289.1632	560.2926	280.6499	559.3086	280.1579	5
21	2157.1624	1079.0848	2140.1359	1070.5716	2139.1518	1070.0796	T	476.2715	238.6394	459.2449	230.1261	458.2609	229.6341	4
22	2286.2050	1143.6061	2269.1785	1135.0929	2268.1944	1134.6009	E	375.2238	188.1155	358.1973	179.6023	357.2132	179.1103	3
23	2385.2734	1193.1403	2368.2469	1184.6271	2367.2628	1184.1351	V	246.1812	123.5942	229.1547	115.0810			2
24							K	147.1128	74.0600	130.0863	65.5468			1

MS/MS Fragmentation of **LLIVSNPVDILTYVAWK**
Found in **LDHA_HUMAN** in **SwissProt**, L-lactate dehydrogenase A chain OS=Homo sapiens GN=LDHA PE=1 SV=2

Match to Query 363: 1942.793048 from(972.403800,2+) intensity(15696.6000) index(157)
Data file IS_111911_23a.txt

Click mouse within plot area to zoom in by factor of two about that point
Or, Plot from 100 to 1700 Da Full range
Label all possible matches ○ Label matches used for scoring ◉
Show Y-axis ☐

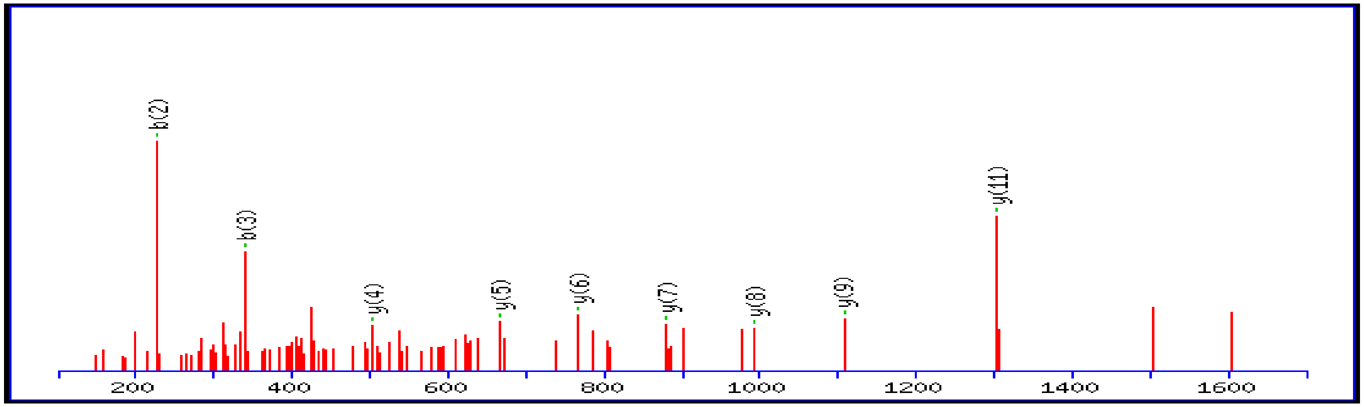


```
Monoisotopic mass of neutral peptide Mr(calc): 1943.1132
Fixed modifications: Carbamidomethyl (C) (apply to specified residues or termini only)
Ions Score: 46  Expect: 0.0029
Matches : 9/164 fragment ions using 13 most intense peaks  (help)
```

#	b	b^{++}	b^*	b^{*++}	b^0	b^{0++}	Seq.	y	y^{++}	y^*	y^{*++}	y^0	y^{0++}	#
1	114.0913	57.5493					L							17
2	**227.1754**	114.0913					L	1831.0364	916.0218	1814.0099	907.5086	1813.0258	907.0166	16
3	**340.2595**	170.6334					I	1717.9523	859.4798	1700.9258	850.9665	1699.9418	850.4745	15
4	439.3279	220.1676					V	1604.8683	802.9378	1587.8417	794.4245	1586.8577	793.9325	14
5	526.3599	263.6836			508.3493	254.6783	S	1505.7999	753.4036	1488.7733	744.8903	1487.7893	744.3983	13
6	640.4028	320.7051	623.3763	312.1918	622.3923	311.6998	N	1418.7678	709.8876	1401.7413	701.3743	1400.7573	700.8823	12
7	737.4556	369.2314	720.4291	360.7182	719.4450	360.2262	P	***1304.7249***	652.8661	1287.6984	644.3528	1286.7143	643.8608	11
8	836.5240	418.7656	819.4975	410.2524	818.5134	409.7604	V	1207.6721	604.3397	1190.6456	595.8264	1189.6616	595.3344	10
9	951.5510	476.2791	934.5244	467.7658	933.5404	467.2738	D	***1108.6037***	554.8055	1091.5772	546.2922	1090.5932	545.8002	9
10	1064.6350	532.8211	1047.6085	524.3079	1046.6245	523.8159	I	***993.5768***	497.2920	976.5502	488.7788	975.5662	488.2867	8
11	1177.7191	589.3632	1160.6925	580.8499	1159.7085	580.3579	L	***880.4927***	440.7500	863.4662	432.2367	862.4822	431.7447	7
12	1278.7668	639.8870	1261.7402	631.3737	1260.7562	630.8817	T	***767.4087***	384.2080	750.3821	375.6947	749.3981	375.2027	6
13	1441.8301	721.4187	1424.8035	712.9054	1423.8195	712.4134	Y	***666.3610***	333.6841	649.3344	325.1709			5
14	1540.8985	770.9529	1523.8720	762.4396	1522.8879	761.9476	V	***503.2976***	252.1525	486.2711	243.6392			4
15	1611.9356	806.4714	1594.9091	797.9582	1593.9251	797.4662	A	404.2292	202.6183	387.2027	194.1050			3
16	1798.0149	899.5111	1780.9884	890.9978	1780.0044	890.5058	W	333.1921	167.0997	316.1656	158.5864			2
17							K	147.1128	74.0600	130.0863	65.5468			1

MS/MS Fragmentation of **LIIVSNPVDILTYVAWK**
Found in **LDH6B_HUMAN** in **SwissProt**, L-lactate dehydrogenase A-like 6B OS=Homo sapiens GN=LDHAL6B PE=1 SV=3

Match to Query 363: 1942.793048 from(972.403800,2+) intensity(15696.6000) index(157)
Data file IS_111911_23a.txt

Click mouse within plot area to zoom in by factor of two about that point
Or, Plot from 100 to 1700 Da Full range
Label all possible matches ○ Label matches used for scoring ◉
Show Y-axis ☐

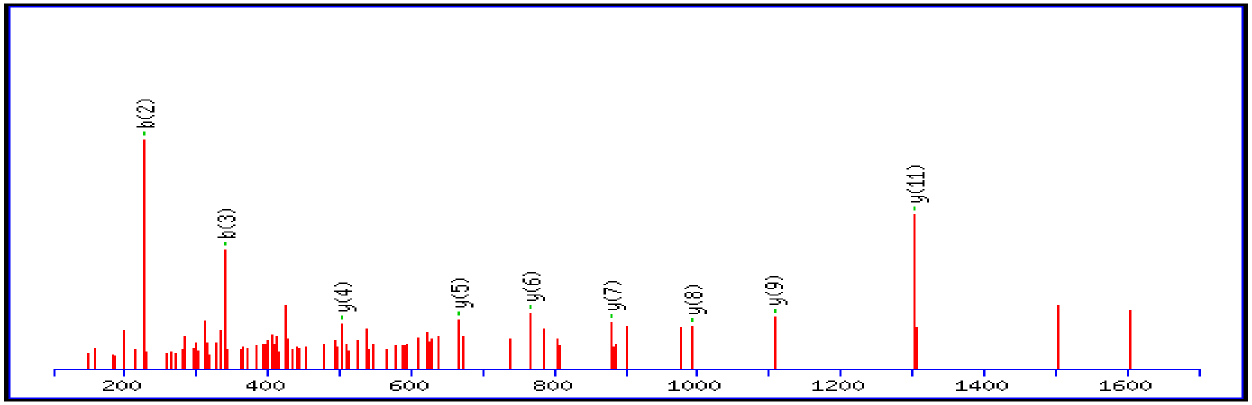


```
Monoisotopic mass of neutral peptide Mr(calc): 1943.1132
Fixed modifications: Carbamidomethyl (C) (apply to specified residues or termini only)
Ions Score: 46  Expect: 0.0029
Matches : 9/164 fragment ions using 13 most intense peaks   (help)
```

#	b	b^{++}	b^{*}	b^{*++}	b^{0}	b^{0++}	Seq.	y	y^{++}	y^{*}	y^{*++}	y^{0}	y^{0++}	#
1	114.0913	57.5493					L							17
2	227.1754	114.0913					I	1831.0364	916.0218	1814.0099	907.5086	1813.0258	907.0166	16
3	340.2595	170.6334					I	1717.9523	859.4798	1700.9258	850.9665	1699.9418	850.4745	15
4	439.3279	220.1676					V	1604.8683	802.9378	1587.8417	794.4245	1586.8577	793.9325	14
5	526.3599	263.6836			508.3493	254.6783	S	1505.7999	753.4036	1488.7733	744.8903	1487.7893	744.3983	13
6	640.4028	320.7051	623.3763	312.1918	622.3923	311.6998	N	1418.7678	709.8876	1401.7413	701.3743	1400.7573	700.8823	12
7	737.4556	369.2314	720.4291	360.7182	719.4450	360.2262	P	1304.7249	652.8661	1287.6984	644.3528	1286.7143	643.8608	11
8	836.5240	418.7656	819.4975	410.2524	818.5134	409.7604	V	1207.6721	604.3397	1190.6456	595.8264	1189.6616	595.3344	10
9	951.5510	476.2791	934.5244	467.7658	933.5404	467.2738	D	1108.6037	554.8055	1091.5772	546.2922	1090.5932	545.8002	9
10	1064.6350	532.8211	1047.6085	524.3079	1046.6245	523.8159	I	993.5768	497.2920	976.5502	488.7788	975.5662	488.2867	8
11	1177.7191	589.3632	1160.6925	580.8499	1159.7085	580.3579	L	880.4927	440.7500	863.4662	432.2367	862.4822	431.7447	7
12	1278.7668	639.8870	1261.7402	631.3737	1260.7562	630.8817	T	767.4087	384.2080	750.3821	375.6947	749.3981	375.2027	6
13	1441.8301	721.4187	1424.8035	712.9054	1423.8195	712.4134	Y	666.3610	333.6841	649.3344	325.1709			5
14	1540.8985	770.9529	1523.8720	762.4396	1522.8879	761.9476	V	503.2976	252.1525	486.2711	243.6392			4
15	1611.9356	806.4714	1594.9091	797.9582	1593.9251	797.4662	A	404.2292	202.6183	387.2027	194.1050			3
16	1798.0149	899.5111	1780.9884	890.9978	1780.0044	890.5058	W	333.1921	167.0997	316.1656	158.5864			2
17							K	147.1128	74.0600	130.0863	65.5468			1

MS/MS Fragmentation of AAGVNVEPFWPGLFAK
Found in RLA1_HUMAN in SwissProt, 60S acidic ribosomal protein P1 OS=Homo sapiens GN=RPLP1 PE=1 SV=1

Match to Query 324: 1701.847248 from(851.930900,2+) intensity(17553.6000) index(378)
Data file IS_111911_23a.txt

Click mouse within plot area to zoom in by factor of two about that point
Or, Plot from 100 to 1500 Da Full range
Label all possible matches ○ Label matches used for scoring ◉
Show Y-axis ☐

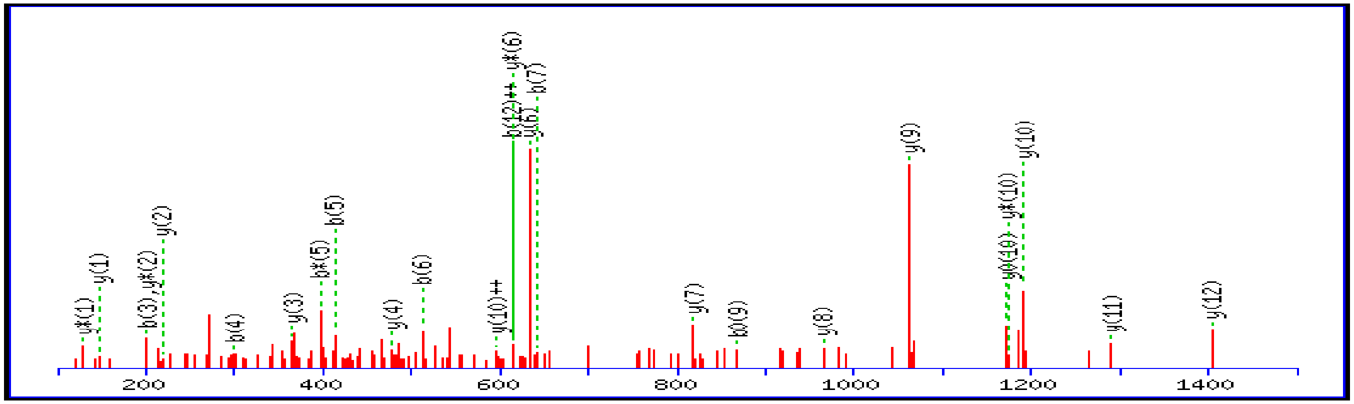

Monoisotopic mass of neutral peptide Mr(calc): 1701.8879
Fixed modifications: Carbamidomethyl (C) (apply to specified residues or termini only)
Ions Score: 46 Expect: 0.0041
Matches : 25/142 fragment ions using 69 most intense peaks (help)

#	b	b^{++}	b^{*}	b^{*++}	b^{0}	b^{0++}	Seq.	y	y^{++}	y^{*}	y^{*++}	y^{0}	y^{0++}	#
1	72.0444	36.5258					A							16
2	143.0815	72.0444					A	1631.8580	816.4327	1614.8315	807.9194	1613.8475	807.4274	15
3	**200.1030**	100.5551					G	1560.8209	780.9141	1543.7944	772.4008	1542.8104	771.9088	14
4	**299.1714**	150.0893					V	1503.7995	752.4034	1486.7729	743.8901	1485.7889	743.3981	13
5	**413.2143**	207.1108	396.1878	198.5975			N	***1404.7310***	702.8692	1387.7045	694.3559	1386.7205	693.8639	12
6	**512.2827**	256.6450	495.2562	248.1317			V	***1290.6881***	645.8477	1273.6616	637.3344	1272.6776	636.8424	11
7	**641.3253**	321.1663	624.2988	312.6530	623.3148	312.1610	E	***1191.6197***	596.3135	1174.5932	587.8002	1173.6091	587.3082	10
8	738.3781	369.6927	721.3515	361.1794	720.3675	360.6874	P	***1062.5771***	531.7922	1045.5506	523.2789			9
9	885.4465	443.2269	868.4199	434.7136	867.4359	434.2216	F	***965.5244***	483.2658	948.4978	474.7525			8
10	1071.5258	536.2665	1054.4993	527.7533	1053.5152	527.2613	W	***818.4559***	409.7316	801.4294	401.2183			7
11	1168.5786	584.7929	1151.5520	576.2796	1150.5680	575.7876	P	***632.3766***	316.6920	615.3501	308.1787			6
12	1225.6000	613.3037	1208.5735	604.7904	1207.5895	604.2984	G	535.3239	268.1656	518.2973	259.6523			5
13	1338.6841	669.8457	1321.6576	661.3324	1320.6735	660.8404	L	***478.3024***	239.6548	461.2758	231.1416			4
14	1485.7525	743.3799	1468.7260	734.8666	1467.7419	734.3746	F	***365.2183***	183.1128	348.1918	174.5995			3
15	1556.7896	778.8985	1539.7631	770.3852	1538.7791	769.8932	A	***218.1499***	109.5786	201.1234	101.0653			2
16							K	***147.1128***	74.0600	130.0863	65.5468			1

MS/MS Fragmentation of SVDEVFDEVVQIFDKEG
Found in KCY_HUMAN in SwissProt, UMP-CMP kinase OS=Homo sapiens GN=CMPK1 PE=1 SV=3

Match to Query 372: 1953.590848 from(977.802700,2+) intensity(14076.2000) index(156)
Data file IS_111911_23a.txt

Click mouse within plot area to zoom in by factor of two about that point
Or, Plot from 100 to 1500 Da Full range
Label all possible matches ○ Label matches used for scoring ◉
Show Y-axis ☐

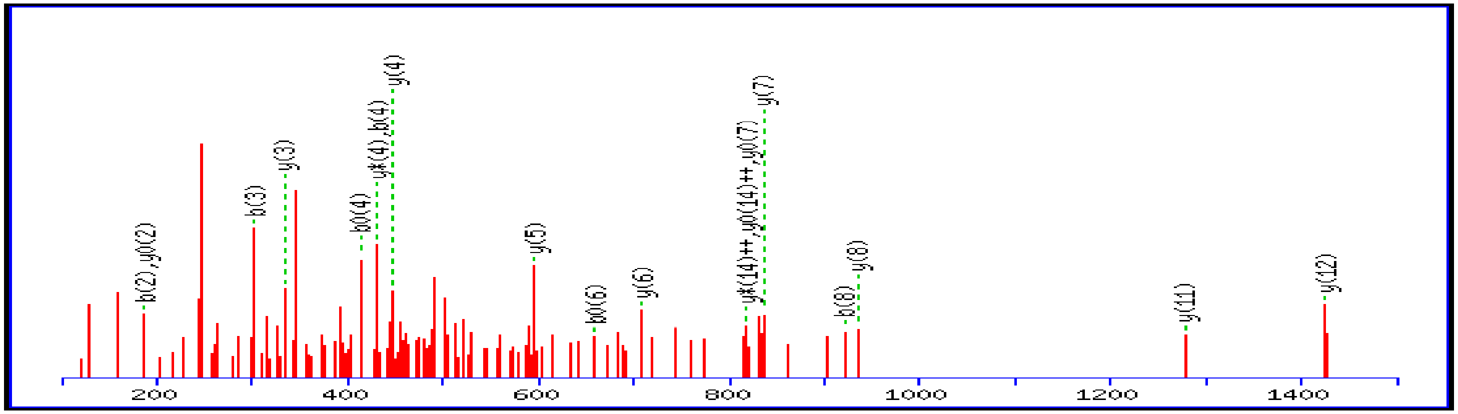

Monoisotopic mass of neutral peptide Mr(calc): 1953.9208
Fixed modifications: Carbamidomethyl (C) (apply to specified residues or termini only)
Ions Score: 45 Expect: 0.0029
Matches : 19/166 fragment ions using 30 most intense peaks (help)

#	b	b^{++}	b^{*}	b^{*++}	b^{0}	b^{0++}	Seq.	y	y^{++}	y^{*}	y^{*++}	y^{0}	y^{0++}	#
1	88.0393	44.5233			70.0287	35.5180	S							17
2	187.1077	94.0575			169.0972	85.0522	V	1867.8960	934.4516	1850.8695	925.9384	1849.8854	925.4464	16
3	302.1347	151.5710			284.1241	142.5657	D	1768.8276	884.9174	1751.8010	876.4042	1750.8170	875.9121	15
4	431.1773	216.0923			413.1667	207.0870	E	1653.8006	827.4040	1636.7741	818.8907	1635.7901	818.3987	14
5	530.2457	265.6265			512.2351	256.6212	V	1524.7581	762.8827	1507.7315	754.3694	1506.7475	753.8774	13
6	677.3141	339.1607			659.3035	330.1554	F	1425.6896	713.3485	1408.6631	704.8352	1407.6791	704.3432	12
7	792.3410	396.6742			774.3305	387.6689	D	1278.6212	639.8142	1261.5947	631.3010	1260.6107	630.8090	11
8	921.3836	461.1954			903.3731	452.1902	E	1163.5943	582.3008	1146.5677	573.7875	1145.5837	573.2955	10
9	1020.4520	510.7297			1002.4415	501.7244	V	1034.5517	517.7795	1017.5251	509.2662	1016.5411	508.7742	9
10	1119.5204	560.2639			1101.5099	551.2586	V	935.4833	468.2453	918.4567	459.7320	917.4727	459.2400	8
11	1247.5790	624.2931	1230.5525	615.7799	1229.5685	615.2879	Q	836.4149	418.7111	819.3883	410.1978	818.4043	409.7058	7
12	1360.6631	680.8352	1343.6365	672.3219	1342.6525	671.8299	I	708.3563	354.6818	691.3297	346.1685	690.3457	345.6765	6
13	1507.7315	754.3694	1490.7050	745.8561	1489.7209	745.3641	F	595.2722	298.1397	578.2457	289.6265	577.2617	289.1345	5
14	1622.7584	811.8829	1605.7319	803.3696	1604.7479	802.8776	D	448.2038	224.6055	431.1773	216.0923	430.1932	215.6003	4
15	1750.8534	875.9303	1733.8269	867.4171	1732.8428	866.9251	K	333.1769	167.0921	316.1503	158.5788	315.1663	158.0868	3
16	1879.8960	940.4516	1862.8695	931.9384	1861.8854	931.4464	E	205.0819	103.0446			187.0713	94.0393	2
17							G	76.0393	38.5233					1

MS/MS Fragmentation of AGQLISELFTNR
Found in CES1P_HUMAN in SwissProt, Putative inactive carboxylesterase 4 OS=Homo sapiens GN=CES1P1 PE=5 SV=2

Match to Query 229: 1348.051648 from(675.033100,2+) intensity(9656.8000) index(76)
Data file IS_111911_23a.txt

Click mouse within plot area to zoom in by factor of two about that point
Or, Plot from 100 to 1400 Da Full range
Label all possible matches ○ Label matches used for scoring ◉
Show Y-axis ☐

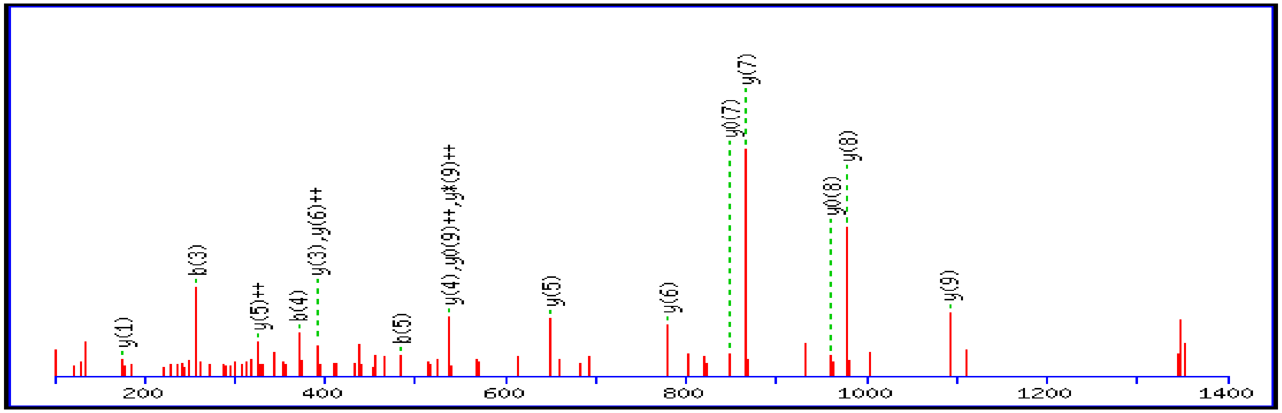

Monoisotopic mass of neutral peptide Mr(calc): 1347.7146
Fixed modifications: Carbamidomethyl (C) (apply to specified residues or termini only)
Ions Score: 44 Expect: 0.0071
Matches : 17/114 fragment ions using 31 most intense peaks (help)

#	b	b^{++}	b^*	b^{*++}	b^0	b^{0++}	Seq.	y	y^{++}	y^*	y^{*++}	y^0	y^{0++}	#
1	72.0444	36.5258					A							12
2	129.0659	65.0366					G	1277.6848	639.3461	1260.6583	630.8328	1259.6743	630.3408	11
3	257.1244	129.0659	240.0979	120.5526			Q	1220.6634	610.8353	1203.6368	602.3220	1202.6528	601.8300	10
4	370.2085	185.6079	353.1819	177.0946			L	*1092.6048*	546.8060	1075.5782	538.2928	1074.5942	537.8007	9
5	483.2926	242.1499	466.2660	233.6366			I	*979.5207*	490.2640	962.4942	481.7507	961.5102	481.2587	8
6	570.3246	285.6659	553.2980	277.1527	552.3140	276.6606	S	*866.4367*	433.7220	849.4101	425.2087	848.4261	424.7167	7
7	699.3672	350.1872	682.3406	341.6740	681.3566	341.1819	E	*779.4046*	390.2060	762.3781	381.6927	761.3941	381.2007	6
8	812.4512	406.7293	795.4247	398.2160	794.4407	397.7240	L	*650.3620*	325.6847	633.3355	317.1714	632.3515	316.6794	5
9	959.5197	480.2635	942.4931	471.7502	941.5091	471.2582	F	*537.2780*	269.1426	520.2514	260.6293	519.2674	260.1373	4
10	1060.5673	530.7873	1043.5408	522.2740	1042.5568	521.7820	T	*390.2096*	195.6084	373.1830	187.0951	372.1990	186.6031	3
11	1174.6103	587.8088	1157.5837	579.2955	1156.5997	578.8035	N	289.1619	145.0846	272.1353	136.5713			2
12							R	*175.1190*	88.0631	158.0924	79.5498			1

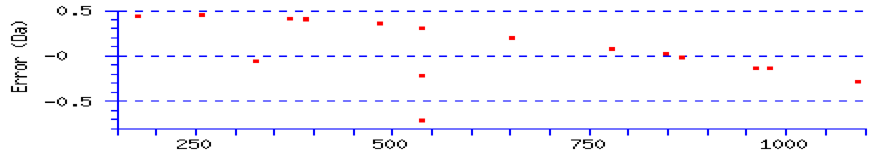

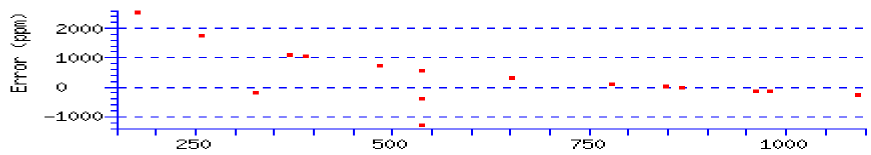

MS/MS Fragmentation of **SFPDFPTPGVVFR**
Found in **APT_HUMAN** in **SwissProt**, Adenine phosphoribosyltransferase OS=Homo sapiens GN=APRT PE=1 SV=2

Match to Query 270: 1464.937648 from(733.476100,2+) intensity(9063.3000) index(355)
Data file IS_111911_23a.txt

Click mouse within plot area to zoom in by factor of two about that point
Or, Plot from 100 to 1500 Da Full range
Label all possible matches ○ Label matches used for scoring ◉
Show Y-axis ☐

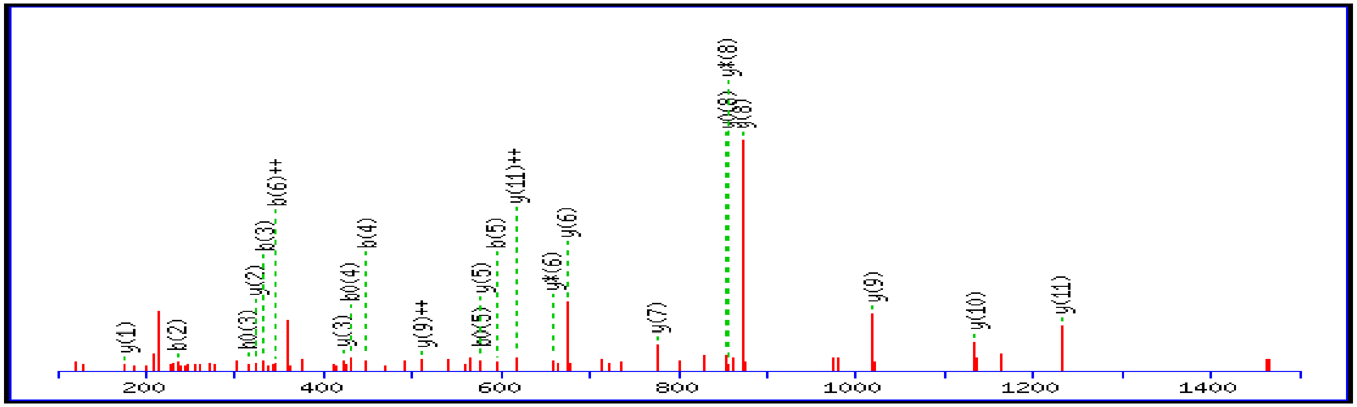

Monoisotopic mass of neutral peptide Mr(calc): 1464.7402
Fixed modifications: Carbamidomethyl (C) (apply to specified residues or termini only)
Ions Score: 42 Expect: 0.011
Matches : 23/108 fragment ions using 62 most intense peaks (help)

#	b	b^{++}	b^{0}	b^{0++}	Seq.	y	y^{++}	y^{*}	y^{*++}	y^{0}	y^{0++}	#
1	88.0393	44.5233	70.0287	35.5180	S							13
2	**235.1077**	118.0575	217.0972	109.0522	F	1378.7154	689.8613	1361.6889	681.3481	1360.7048	680.8561	12
3	**332.1605**	166.5839	**314.1499**	157.5786	P	***1231.6470***	**616.3271**	1214.6204	607.8139	1213.6364	607.3218	11
4	**447.1874**	224.0974	**429.1769**	215.0921	D	***1134.5942***	567.8007	1117.5677	559.2875	1116.5837	558.7955	10
5	**594.2558**	297.6316	**576.2453**	288.6263	F	***1019.5673***	**510.2873**	1002.5407	501.7740	1001.5567	501.2820	9
6	691.3086	**346.1579**	673.2980	337.1527	P	***872.4989***	436.7531	**855.4723**	428.2398	**854.4883**	427.7478	8
7	792.3563	396.6818	774.3457	387.6765	T	***775.4461***	388.2267	758.4196	379.7134	757.4355	379.2214	7
8	889.4090	445.2082	871.3985	436.2029	P	***674.3984***	337.7028	**657.3719**	329.1896			6
9	946.4305	473.7189	928.4199	464.7136	G	***577.3457***	289.1765	560.3191	280.6632			5
10	1045.4989	523.2531	1027.4884	514.2478	V	520.3242	260.6657	503.2976	252.1525			4
11	1144.5673	572.7873	1126.5568	563.7820	V	***421.2558***	211.1315	404.2292	202.6183			3
12	1291.6358	646.3215	1273.6252	637.3162	F	***322.1874***	161.5973	305.1608	153.0840			2
13					R	***175.1190***	88.0631	158.0924	79.5498			1

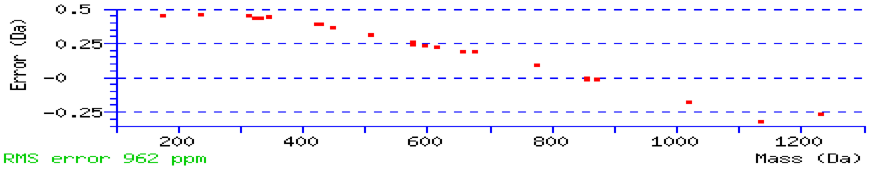

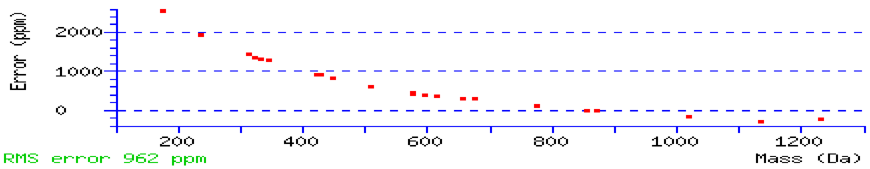

MS/MS Fragmentation of **LFEGNALLR**
Found in **RS9_HUMAN** in **SwissProt**, 40S ribosomal protein S9 OS=Homo sapiens GN=RPS9 PE=1 SV=3

Match to Query 114: 1032.185448 from(517.100000,2+) intensity(5002.5000) index(40)
Data file IS_111911_23a.txt

Click mouse within plot area to zoom in by factor of two about that point
Or, [Plot from] 100 to 950 Da [Full range]
Label all possible matches ○ Label matches used for scoring ◉
Show Y-axis ☐

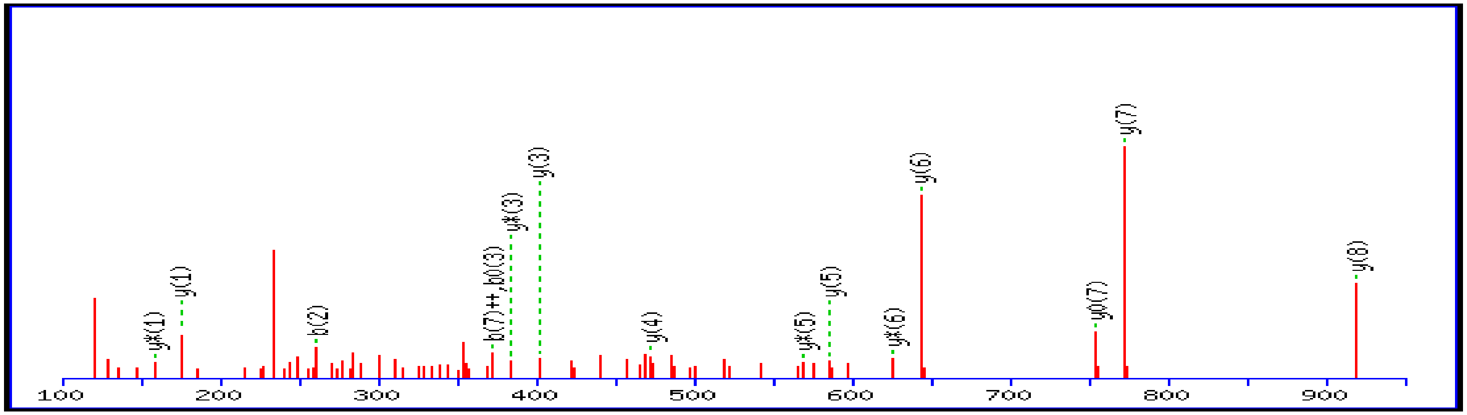

Monoisotopic mass of neutral peptide Mr(calc): 1031.5764
Fixed modifications: Carbamidomethyl (C) (apply to specified residues or termini only)
Ions Score: 39 Expect: 0.022
Matches : 15/72 fragment ions using 28 most intense peaks (help)

#	b	b^{++}	b^{*}	b^{*++}	b^{0}	b^{0++}	Seq.	y	y^{++}	y^{*}	y^{*++}	y^{0}	y^{0++}	#
1	114.0913	57.5493					L							9
2	261.1598	131.0835					F	*919.4996*	460.2534	902.4730	451.7402	901.4890	451.2482	8
3	390.2023	195.6048			372.1918	186.5995	E	*772.4312*	386.7192	755.4046	378.2060	754.4206	377.7139	7
4	447.2238	224.1155			429.2132	215.1103	G	*643.3886*	322.1979	626.3620	313.6847			6
5	561.2667	281.1370	544.2402	272.6237	543.2562	272.1317	N	*586.3671*	293.6872	569.3406	285.1739			5
6	632.3039	316.6556	615.2773	308.1423	614.2933	307.6503	A	*472.3242*	236.6657	455.2976	228.1525			4
7	745.3879	373.1976	728.3614	364.6843	727.3774	364.1923	L	*401.2871*	201.1472	384.2605	192.6339			3
8	858.4720	429.7396	841.4454	421.2264	840.4614	420.7343	L	288.2030	144.6051	271.1765	136.0919			2
9							R	*175.1190*	88.0631	158.0924	79.5498			1

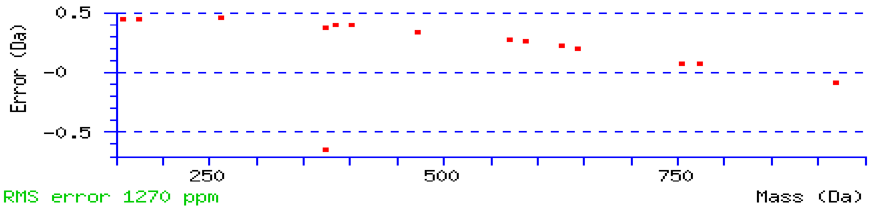

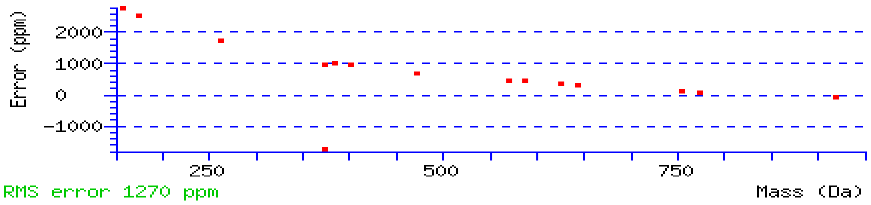

MS/MS Fragmentation of **INVNEIFYDLVR**
Found in **RAP1A_HUMAN** in **SwissProt**, Ras-related protein Rap-1A OS=Homo sapiens GN=RAP1A PE=1 SV=1

Match to Query 277: 1493.966048 from(747.990300,2+) intensity(3111.1000) index(131)
Data file IS_111911_23a.txt

Click mouse within plot area to zoom in by factor of two about that point
Or, Plot from 0 to 2400 Da Full range
Label all possible matches ○ Label matches used for scoring ◉
Show Y-axis ☐

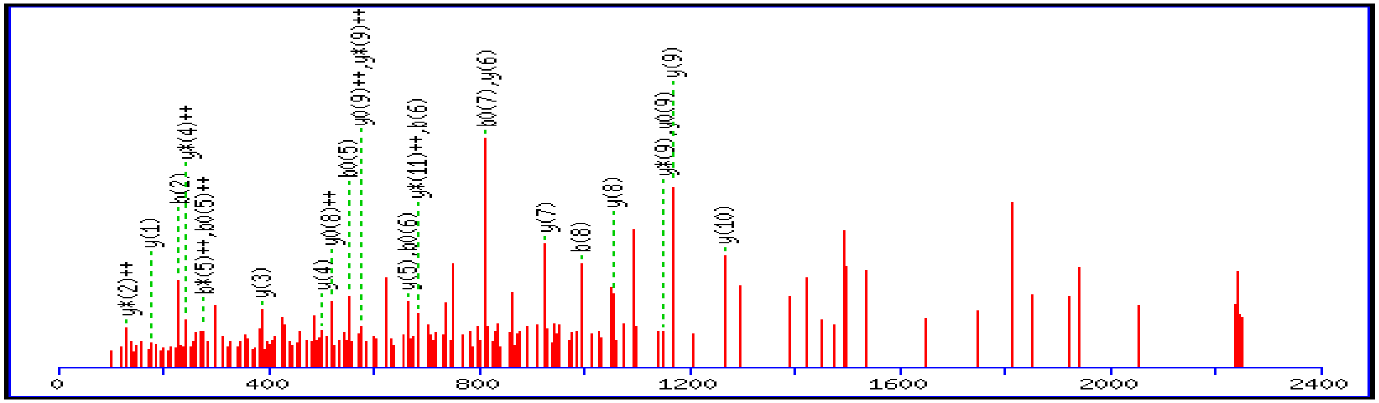

Monoisotopic mass of neutral peptide Mr(calc): 1493.7878
Fixed modifications: Carbamidomethyl (C) (apply to specified residues or termini only)
Ions Score: 37 Expect: 0.036
Matches : 25/116 fragment ions using 53 most intense peaks (help)

#	b	b^{++}	b^{*}	b^{*++}	b^{0}	b^{0++}	Seq.	y	y^{++}	y^{*}	y^{*++}	y^{0}	y^{0++}	#
1	114.0913	57.5493					I							12
2	**228.1343**	114.5708	211.1077	106.0575			N	1381.7110	691.3592	1364.6845	682.8459	1363.7005	682.3539	11
3	327.2027	164.1050	310.1761	155.5917			V	***1267.6681***	634.3377	1250.6416	625.8244	1249.6575	625.3324	10
4	441.2456	221.1264	424.2191	212.6132			N	***1168.5997***	584.8035	1151.5732	576.2902	1150.5891	575.7982	9
5	570.2882	285.6477	553.2617	277.1345	552.2776	276.6425	E	***1054.5568***	527.7820	1037.5302	519.2688	1036.5462	518.7767	8
6	**683.3723**	342.1898	666.3457	333.6765	665.3617	333.1845	I	***925.5142***	463.2607	908.4876	454.7475	907.5036	454.2554	7
7	830.4407	415.7240	813.4141	407.2107	812.4301	406.7187	F	***812.4301***	406.7187	795.4036	398.2054	794.4196	397.7134	6
8	**993.5040**	497.2556	976.4775	488.7424	975.4934	488.2504	Y	***665.3617***	333.1845	648.3352	324.6712	647.3511	324.1792	5
9	1108.5310	554.7691	1091.5044	546.2558	1090.5204	545.7638	D	***502.2984***	251.6528	485.2718	243.1395	484.2878	242.6475	4
10	1221.6150	611.3111	1204.5885	602.7979	1203.6045	602.3059	L	***387.2714***	194.1394	370.2449	185.6261			3
11	1320.6834	660.8454	1303.6569	652.3321	1302.6729	651.8401	V	274.1874	137.5973	257.1608	129.0840			2
12							R	***175.1190***	88.0631	158.0924	79.5498			1

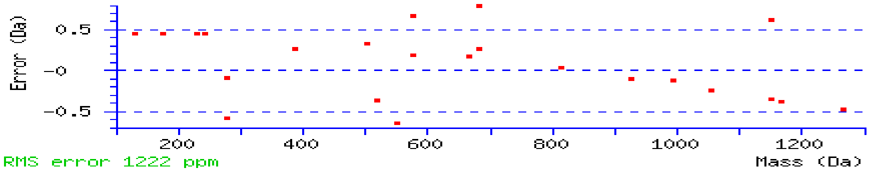

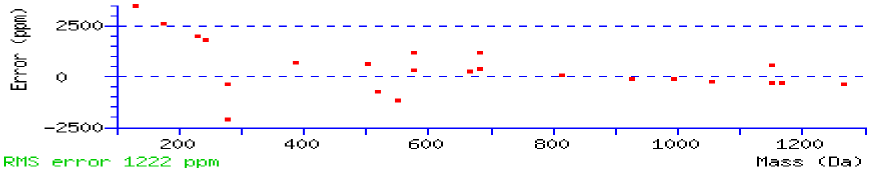

MS/MS Fragmentation of **FILNLPTFSVR**
Found in **PSB2_HUMAN** in **SwissProt**, Proteasome subunit beta type-2 OS=Homo sapiens GN=PSMB2 PE=1 SV=1

Match to Query 208: 1306.118648 from(654.066600,2+) intensity(24217.6000) index(377)
Data file IS_111911_23a.txt

Click mouse within plot area to zoom in by factor of two about that point
Or, Plot from 100 to 1400 Da Full range
Label all possible matches ○ Label matches used for scoring ◉
Show Y-axis ☐

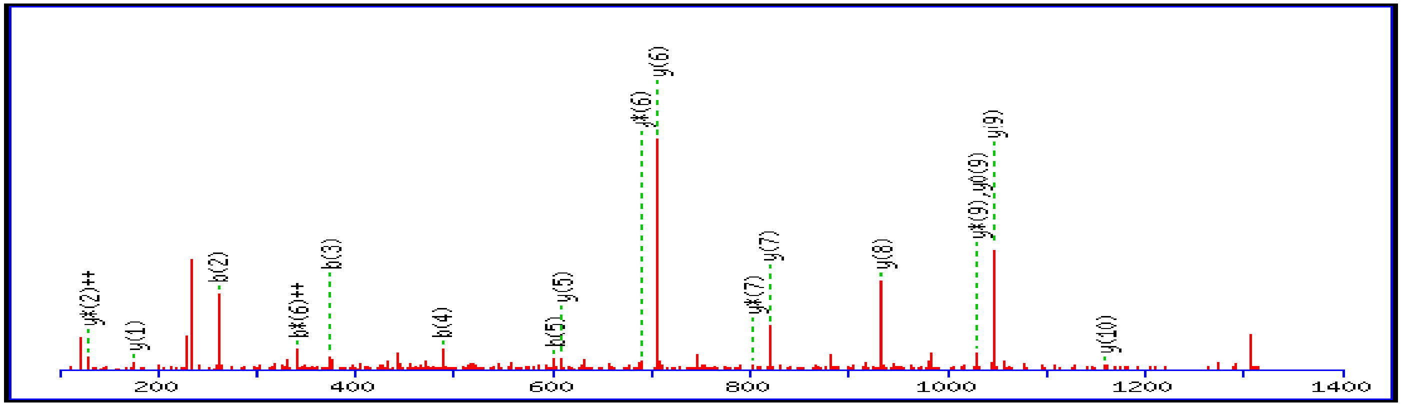


```
Monoisotopic mass of neutral peptide Mr(calc): 1305.7445
Fixed modifications: Carbamidomethyl (C) (apply to specified residues or termini only)
Ions Score: 35 Expect: 0.04
Matches : 17/98 fragment ions using 36 most intense peaks (help)
```

#	b	b^{++}	b^{*}	b^{*++}	b^{0}	b^{0++}	Seq.	y	y^{++}	y^{*}	y^{*++}	y^{0}	y^{0++}	#
1	148.0757	74.5415					F							11
2	**261.1598**	131.0835					I	***1159.6834***	580.3453	1142.6568	571.8320	1141.6728	571.3400	10
3	**374.2438**	187.6255					L	***1046.5993***	523.8033	1029.5728	515.2900	1028.5887	514.7980	9
4	**488.2867**	244.6470	471.2602	236.1337			N	***933.5152***	467.2613	916.4887	458.7480	915.5047	458.2560	8
5	**601.3708**	301.1890	584.3443	292.6758			L	***819.4723***	410.2398	802.4458	401.7265	801.4617	401.2345	7
6	698.4236	349.7154	681.3970	341.2022			P	***706.3883***	353.6978	689.3617	345.1845	688.3777	344.6925	6
7	799.4713	400.2393	782.4447	391.7260	781.4607	391.2340	T	***609.3355***	305.1714	592.3089	296.6581	591.3249	296.1661	5
8	946.5397	473.7735	929.5131	465.2602	928.5291	464.7682	F	508.2878	254.6475	491.2613	246.1343	490.2772	245.6423	4
9	1033.5717	517.2895	1016.5451	508.7762	1015.5611	508.2842	S	361.2194	181.1133	344.1928	172.6001	343.2088	172.1081	3
10	1132.6401	566.8237	1115.6136	558.3104	1114.6295	557.8184	V	274.1874	137.5973	257.1608	129.0840			2
11							R	***175.1190***	88.0631	158.0924	79.5498			1

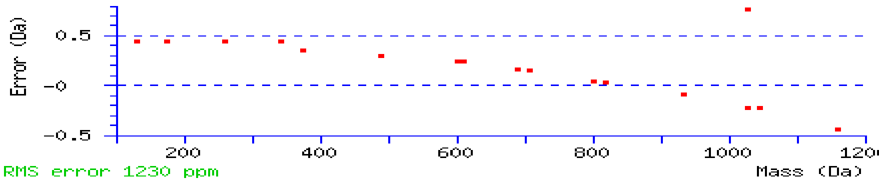

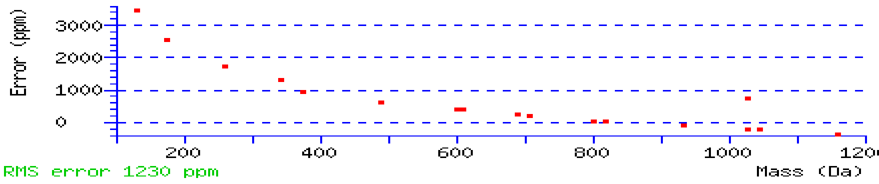

MS/MS Fragmentation of NLSDLIDLVPSLCEDLLSSVDQPLK
Found in CAPZB_HUMAN in SwissProt, F-actin-capping protein subunit beta OS=Homo sapiens GN=CAPZB PE=1 SV=4

Match to Query 445: 2782.086972 from(928.369600,3+) intensity(1259.4000) index(176)
Data file IS_111911_23a.txt

Click mouse within plot area to zoom in by factor of two about that point
Or, Plot from 300 to 1900 Da Full range
Label all possible matches ○ Label matches used for scoring ◉
Show Y-axis ☐

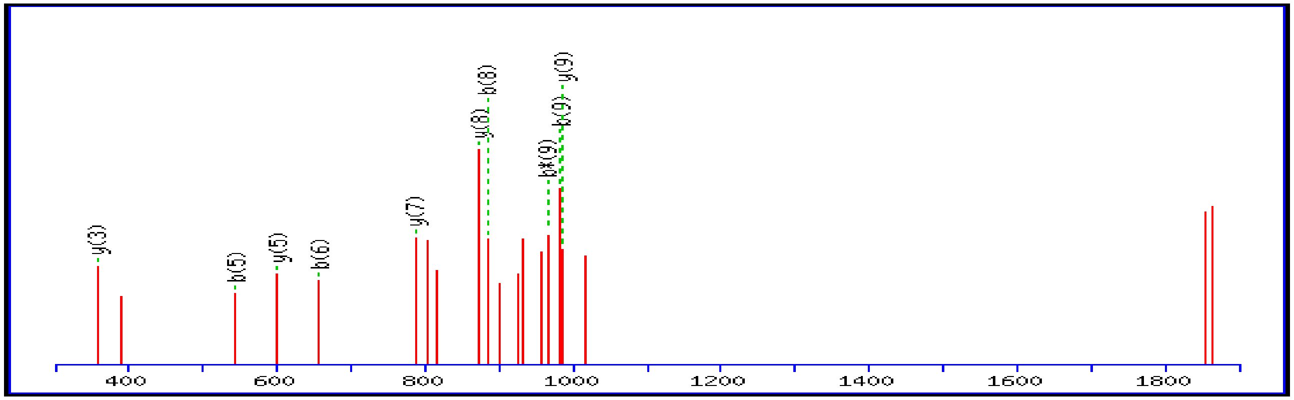

Monoisotopic mass of neutral peptide Mr(calc): 2782.4310
Fixed modifications: Carbamidomethyl (C) (apply to specified residues or termini only)
Ions Score: 30 Expect: 0.07
Matches : 10/276 fragment ions using 17 most intense peaks (help)

#	b	b++	b*	b*++	b0	b0++	Seq.	y	y++	y*	y*++	y0	y0++	#
1	115.0502	58.0287	98.0237	49.5155			N							25
2	228.1343	114.5708	211.1077	106.0575			L	2669.3954	1335.2013	2652.3688	1326.6881	2651.3848	1326.1960	24
3	315.1663	158.0868	298.1397	149.5735	297.1557	149.0815	S	2556.3113	1278.6593	2539.2848	1270.1460	2538.3007	1269.6540	23
4	430.1932	215.6003	413.1667	207.0870	412.1827	206.5950	D	2469.2793	1235.1433	2452.2527	1226.6300	2451.2687	1226.1380	22
5	543.2773	272.1423	526.2508	263.6290	525.2667	263.1370	L	2354.2523	1177.6298	2337.2258	1169.1165	2336.2418	1168.6245	21
6	656.3614	328.6843	639.3348	320.1710	638.3508	319.6790	I	2241.1683	1121.0878	2224.1417	1112.5745	2223.1577	1112.0825	20
7	771.3883	386.1978	754.3618	377.6845	753.3777	377.1925	D	2128.0842	1064.5457	2111.0577	1056.0325	2110.0736	1055.5405	19
8	884.4724	442.7398	867.4458	434.2266	866.4618	433.7345	L	2013.0573	1007.0323	1996.0307	998.5190	1995.0467	998.0270	18
9	983.5408	492.2740	966.5142	483.7608	965.5302	483.2687	V	1899.9732	950.4902	1882.9467	941.9770	1881.9626	941.4850	17
10	1080.5936	540.8004	1063.5670	532.2871	1062.5830	531.7951	P	1800.9048	900.9560	1783.8782	892.4428	1782.8942	891.9508	16
11	1167.6256	584.3164	1150.5990	575.8032	1149.6150	575.3111	S	1703.8520	852.4297	1686.8255	843.9164	1685.8415	843.4244	15
12	1280.7096	640.8585	1263.6831	632.3452	1262.6991	631.8532	L	1616.8200	808.9136	1599.7935	800.4004	1598.8094	799.9084	14
13	1440.7403	720.8738	1423.7137	712.3605	1422.7297	711.8685	C	1503.7359	752.3716	1486.7094	743.8583	1485.7254	743.3663	13
14	1569.7829	785.3951	1552.7563	776.8818	1551.7723	776.3898	E	1343.7053	672.3563	1326.6787	663.8430	1325.6947	663.3510	12
15	1684.8098	842.9086	1667.7833	834.3953	1666.7993	833.9033	D	1214.6627	607.8350	1197.6361	599.3217	1196.6521	598.8297	11
16	1797.8939	899.4506	1780.8673	890.9373	1779.8833	890.4453	L	1099.6358	550.3215	1082.6092	541.8082	1081.6252	541.3162	10
17	1910.9780	955.9926	1893.9514	947.4793	1892.9674	946.9873	L	986.5517	493.7795	969.5251	485.2662	968.5411	484.7742	9
18	1998.0100	999.5086	1980.9834	990.9954	1979.9994	990.5033	S	873.4676	437.2374	856.4411	428.7242	855.4571	428.2322	8
19	2085.0420	1043.0246	2068.0155	1034.5114	2067.0314	1034.0194	S	786.4356	393.7214	769.4090	385.2082	768.4250	384.7162	7
20	2184.1104	1092.5589	2167.0839	1084.0456	2166.0999	1083.5536	V	699.4036	350.2054	682.3770	341.6921	681.3930	341.2001	6
21	2299.1374	1150.0723	2282.1108	1141.5590	2281.1268	1141.0670	D	600.3352	300.6712	583.3086	292.1579	582.3246	291.6659	5
22	2427.1959	1214.1016	2410.1694	1205.5883	2409.1854	1205.0963	Q	485.3082	243.1577	468.2817	234.6445			4
23	2524.2487	1262.6280	2507.2222	1254.1147	2506.2381	1253.6227	P	357.2496	179.1285	340.2231	170.6152			3
24	2637.3328	1319.1700	2620.3062	1310.6568	2619.3222	1310.1647	L	260.1969	130.6021	243.1703	122.0888			2
25							K	147.1128	74.0600	130.0863	65.5468			1

MS/MS Fragmentation of **MADVPGAQRAVPGDGPEPR**
Found in **TMM88_HUMAN** in **SwissProt**, Transmembrane protein 88 OS=Homo sapiens GN=TMEM88 PE=2 SV=1

Match to Query 361: 1918.541472 from(640.521100,3+) intensity(2821.0000) index(273)
Data file IS_111911_23a.txt

Click mouse within plot area to zoom in by factor of two about that point
Or, Plot from 200 to 1300 Da Full range
Label all possible matches ○ Label matches used for scoring ◉
Show Y-axis ☐

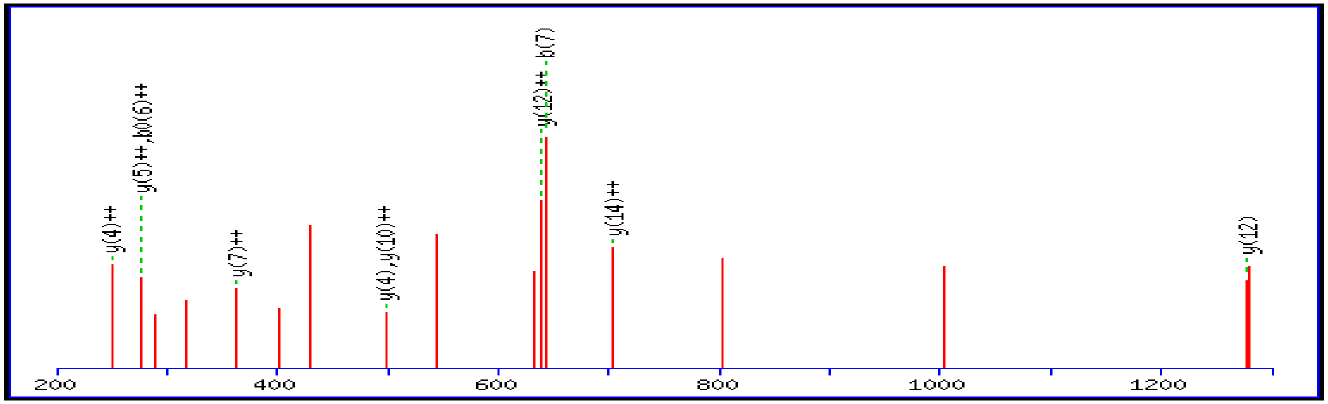

Monoisotopic mass of neutral peptide Mr(calc): 1918.9319
Fixed modifications: Carbamidomethyl (C) (apply to specified residues or termini only)
Ions Score: 30 Expect: 0.097
Matches : 10/194 fragment ions using 14 most intense peaks (help)

#	b	b^{++}	b^{*}	b^{*++}	b^{0}	b^{0++}	Seq.	y	y^{++}	y^{*}	y^{*++}	y^{0}	y^{0++}	#
1	132.0478	66.5275					M							19
2	203.0849	102.0461					A	1788.8987	894.9530	1771.8722	886.4397	1770.8882	885.9477	18
3	318.1118	159.5595			300.1013	150.5543	D	1717.8616	859.4345	1700.8351	850.9212	1699.8511	850.4292	17
4	417.1802	209.0938			399.1697	200.0885	V	1602.8347	801.9210	1585.8081	793.4077	1584.8241	792.9157	16
5	514.2330	257.6201			496.2224	248.6149	P	1503.7663	752.3868	1486.7397	743.8735	1485.7557	743.3815	15
6	571.2545	286.1309			553.2439	277.1256	G	1406.7135	703.8604	1389.6870	695.3471	1388.7029	694.8551	14
7	642.2916	321.6494			624.2810	312.6441	A	1349.6920	675.3497	1332.6655	666.8364	1331.6815	666.3444	13
8	770.3502	385.6787	753.3236	377.1654	752.3396	376.6734	Q	**1278.6549**	639.8311	1261.6284	631.3178	1260.6444	630.8258	12
9	926.4513	463.7293	909.4247	455.2160	908.4407	454.7240	R	1150.5963	575.8018	1133.5698	567.2885	1132.5858	566.7965	11
10	997.4884	499.2478	980.4618	490.7346	979.4778	490.2425	A	994.4952	497.7513	977.4687	489.2380	976.4847	488.7460	10
11	1096.5568	548.7820	1079.5302	540.2688	1078.5462	539.7768	V	923.4581	462.2327	906.4316	453.7194	905.4476	453.2274	9
12	1193.6096	597.3084	1176.5830	588.7951	1175.5990	588.3031	P	824.3897	412.6985	807.3632	404.1852	806.3791	403.6932	8
13	1250.6310	625.8191	1233.6045	617.3059	1232.6205	616.8139	G	727.3369	364.1721	710.3104	355.6588	709.3264	355.1668	7
14	1365.6580	683.3326	1348.6314	674.8193	1347.6474	674.3273	D	670.3155	335.6614	653.2889	327.1481	652.3049	326.6561	6
15	1422.6794	711.8434	1405.6529	703.3301	1404.6689	702.8381	G	555.2885	278.1479	538.2620	269.6346	537.2780	269.1426	5
16	1519.7322	760.3697	1502.7056	751.8565	1501.7216	751.3645	P	**498.2671**	249.6372	481.2405	241.1239	480.2565	240.6319	4
17	1648.7748	824.8910	1631.7482	816.3778	1630.7642	815.8857	E	401.2143	201.1108	384.1878	192.5975	383.2037	192.1055	3
18	1745.8275	873.4174	1728.8010	864.9041	1727.8170	864.4121	P	272.1717	136.5895	255.1452	128.0762			2
19							R	175.1190	88.0631	158.0924	79.5498			1

MS/MS Fragmentation of DATNVGDEGGFAPNILENNEALELLK
Found in ENOB_HUMAN in SwissProt, Beta-enolase OS=Homo sapiens GN=ENO3 PE=1 SV=5

Match to Query 550: 2742.095772 from(915.039200,3+) intensity(20104.1000) index(109)
Data file IS_111911_23b.txt

Click mouse within plot area to zoom in by factor of two about that point
Or, Plot from 0 to 2800 Da Full range
Label all possible matches ○ Label matches used for scoring ◉
Show Y-axis ☐

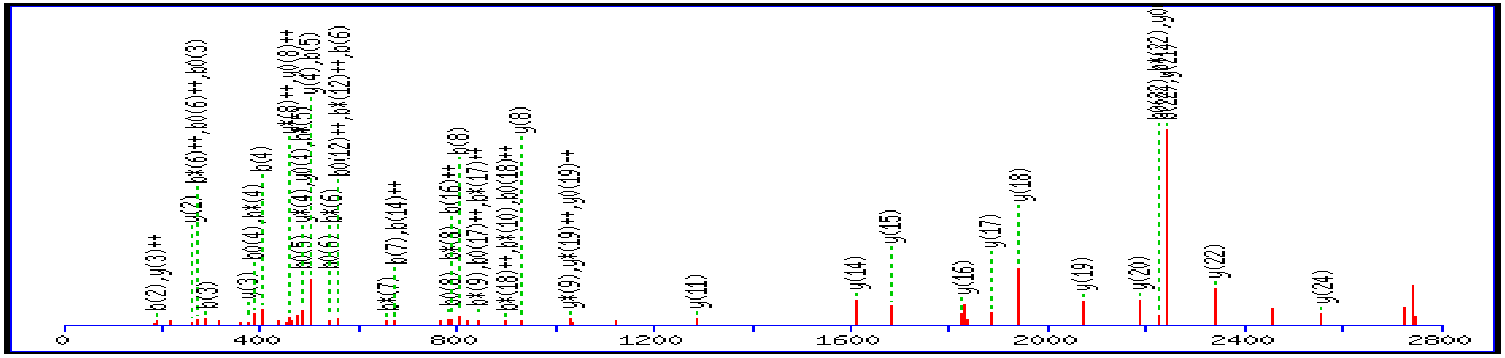


```
Monoisotopic mass of neutral peptide Mr(calc): 2742.3348
Fixed modifications: Carbamidomethyl (C) (apply to specified residues or termini only)
Ions Score: 107   Expect: 1.4e-09
Matches : 56/288 fragment ions using 55 most intense peaks    (help)
```

#	b	b^{++}	b^{*}	b^{*++}	b^{0}	b^{0++}	Seq.	y	y^{++}	y^{*}	y^{*++}	y^{0}	y^{0++}	#
1	116.0342	58.5207			98.0237	49.5155	D							26
2	187.0713	94.0393			169.0608	85.0340	A	2628.3152	1314.6612	2611.2886	1306.1479	2610.3046	1305.6559	25
3	288.1190	144.5631			270.1084	135.5579	T	2557.2780	1279.1427	2540.2515	1270.6294	2539.2675	1270.1374	24
4	402.1619	201.5846	385.1354	193.0713	384.1514	192.5793	N	2456.2304	1228.6188	2439.2038	1220.1055	2438.2198	1219.6135	23
5	501.2304	251.1188	484.2038	242.6055	483.2198	242.1135	V	2342.1874	1171.5974	2325.1609	1163.0841	2324.1769	1162.5921	22
6	558.2518	279.6295	541.2253	271.1163	540.2413	270.6243	G	2243.1190	1122.0631	2226.0925	1113.5499	2225.1085	1113.0579	21
7	673.2788	337.1430	656.2522	328.6297	655.2682	328.1377	D	2186.0976	1093.5524	2169.0710	1085.0391	2168.0870	1084.5471	20
8	802.3214	401.6643	785.2948	393.1510	784.3108	392.6590	E	2071.0706	1036.0389	2054.0441	1027.5257	2053.0600	1027.0337	19
9	859.3428	430.1750	842.3163	421.6618	841.3323	421.1698	G	1942.0280	971.5176	1925.0015	963.0044	1924.0175	962.5124	18
10	916.3643	458.6858	899.3377	450.1725	898.3537	449.6805	G	1885.0066	943.0069	1867.9800	934.4936	1866.9960	934.0016	17
11	1063.4327	532.2200	1046.4061	523.7067	1045.4221	523.2147	F	1827.9851	914.4962	1810.9585	905.9829	1809.9745	905.4909	16
12	1134.4698	567.7385	1117.4433	559.2253	1116.4592	558.7333	A	1680.9167	840.9620	1663.8901	832.4487	1662.9061	831.9567	15
13	1231.5226	616.2649	1214.4960	607.7517	1213.5120	607.2596	P	1609.8796	805.4434	1592.8530	796.9301	1591.8690	796.4381	14
14	1345.5655	673.2864	1328.5390	664.7731	1327.5549	664.2811	N	1512.8268	756.9170	1495.8002	748.4038	1494.8162	747.9118	13
15	1458.6496	729.8284	1441.6230	721.3151	1440.6390	720.8231	I	1398.7839	699.8956	1381.7573	691.3823	1380.7733	690.8903	12
16	1571.7336	786.3705	1554.7071	777.8572	1553.7231	777.3652	L	1285.6998	643.3535	1268.6733	634.8403	1267.6892	634.3483	11
17	1700.7762	850.8917	1683.7497	842.3785	1682.7657	841.8865	E	1172.6157	586.8115	1155.5892	578.2982	1154.6052	577.8062	10
18	1814.8191	907.9132	1797.7926	899.3999	1796.8086	898.9079	N	1043.5732	522.2902	1026.5466	513.7769	1025.5626	513.2849	9
19	1928.8621	964.9347	1911.8355	956.4214	1910.8515	955.9294	N	929.5302	465.2687	912.5037	456.7555	911.5197	456.2635	8
20	2057.9047	1029.4560	2040.8781	1020.9427	2039.8941	1020.4507	E	815.4873	408.2473	798.4607	399.7340	797.4767	399.2420	7
21	2128.9418	1064.9745	2111.9152	1056.4613	2110.9312	1055.9692	A	686.4447	343.7260	669.4182	335.2127	668.4341	334.7207	6
22	2242.0258	1121.5166	2224.9993	1113.0033	2224.0153	1112.5113	L	615.4076	308.2074	598.3810	299.6942	597.3970	299.2022	5
23	2371.0684	1186.0379	2354.0419	1177.5246	2353.0579	1177.0326	E	502.3235	251.6654	485.2970	243.1521	484.3130	242.6601	4
24	2484.1525	1242.5799	2467.1260	1234.0666	2466.1419	1233.5746	L	373.2809	187.1441	356.2544	178.6308			3
25	2597.2366	1299.1219	2580.2100	1290.6086	2579.2260	1290.1166	L	260.1969	130.6021	243.1703	122.0888			2
26							K	147.1128	74.0600	130.0863	65.5468			1

MS/MS Fragmentation of **QSLGELIGTLNAAK**
Found in **TPIS_HUMAN** in **SwissProt**, Triosephosphate isomerase OS=Homo sapiens GN=TPI1 PE=1 SV=3

Match to Query 303: 1414.054448 from(708.034500,2+) intensity(13627.8000) index(396)
Data file IS_111911_23b.txt

Click mouse within plot area to zoom in by factor of two about that point
Or, [Plot from] 100 to 1500 Da [Full range]
Label all possible matches ○ Label matches used for scoring ◉
Show Y-axis ☐

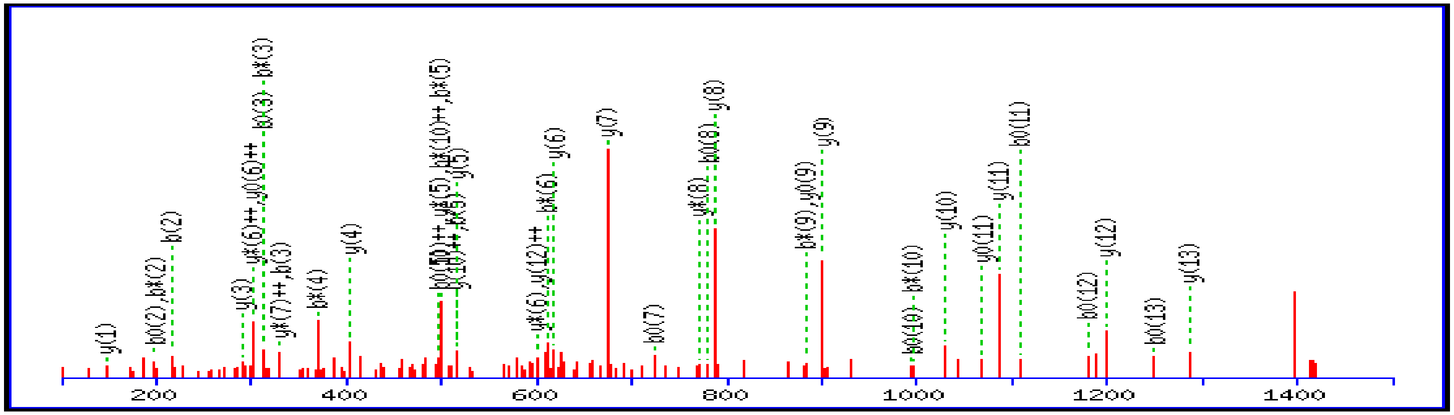


```
Monoisotopic mass of neutral peptide Mr(calc): 1413.7827
Fixed modifications: Carbamidomethyl (C) (apply to specified residues or termini only)
Ions Score: 106  Expect: 3.3e-09
Matches : 43/144 fragment ions using 47 most intense peaks   (help)
```

#	b	b++	b*	b*++	b0	b0++	Seq.	y	y++	y*	y*++	y0	y0++	#
1	129.0659	65.0366	112.0393	56.5233			Q							14
2	216.0979	108.5526	199.0713	100.0393	198.0873	99.5473	S	1286.7314	643.8694	1269.7049	635.3561	1268.7209	634.8641	13
3	329.1819	165.0946	312.1554	156.5813	311.1714	156.0893	L	1199.6994	600.3533	1182.6729	591.8401	1181.6889	591.3481	12
4	386.2034	193.6053	369.1769	185.0921	368.1928	184.6001	G	1086.6154	543.8113	1069.5888	535.2980	1068.6048	534.8060	11
5	515.2460	258.1266	498.2195	249.6134	497.2354	249.1214	E	1029.5939	515.3006	1012.5673	506.7873	1011.5833	506.2953	10
6	628.3301	314.6687	611.3035	306.1554	610.3195	305.6634	L	900.5513	450.7793	883.5247	442.2660	882.5407	441.7740	9
7	741.4141	371.2107	724.3876	362.6974	723.4036	362.2054	I	787.4672	394.2373	770.4407	385.7240	769.4567	385.2320	8
8	798.4356	399.7214	781.4090	391.2082	780.4250	390.7162	G	674.3832	337.6952	657.3566	329.1819	656.3726	328.6899	7
9	899.4833	450.2453	882.4567	441.7320	881.4727	441.2400	T	617.3617	309.1845	600.3352	300.6712	599.3511	300.1792	6
10	1012.5673	506.7873	995.5408	498.2740	994.5568	497.7820	L	516.3140	258.6606	499.2875	250.1474			5
11	1126.6103	563.8088	1109.5837	555.2955	1108.5997	554.8035	N	403.2300	202.1186	386.2034	193.6053			4
12	1197.6474	599.3273	1180.6208	590.8141	1179.6368	590.3220	A	289.1870	145.0972	272.1605	136.5839			3
13	1268.6845	634.8459	1251.6579	626.3326	1250.6739	625.8406	A	218.1499	109.5786	201.1234	101.0653			2
14							K	147.1128	74.0600	130.0863	65.5468			1

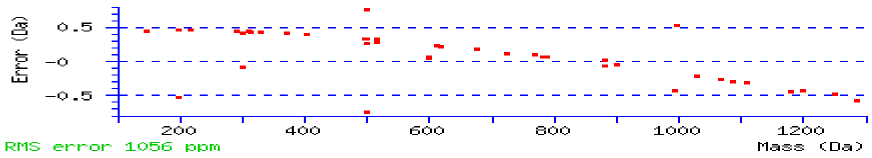

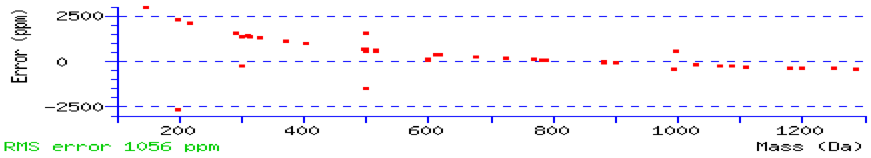

MS/MS Fragmentation of **DMLFQVLALEEPNVR**
Found in **SPRE_HUMAN** in **SwissProt**, Sepiapterin reductase OS=Homo sapiens GN=SPR PE=1 SV=1

Match to Query 397: 1788.779048 from(895.396800,2+) intensity(7350.3000) index(432)
Data file IS_111911_23b.txt

Click mouse within plot area to zoom in by factor of two about that point
Or, [Plot from] 100 to 1800 Da [Full range]
Label all possible matches ○ Label matches used for scoring ◉
Show Y-axis ☐

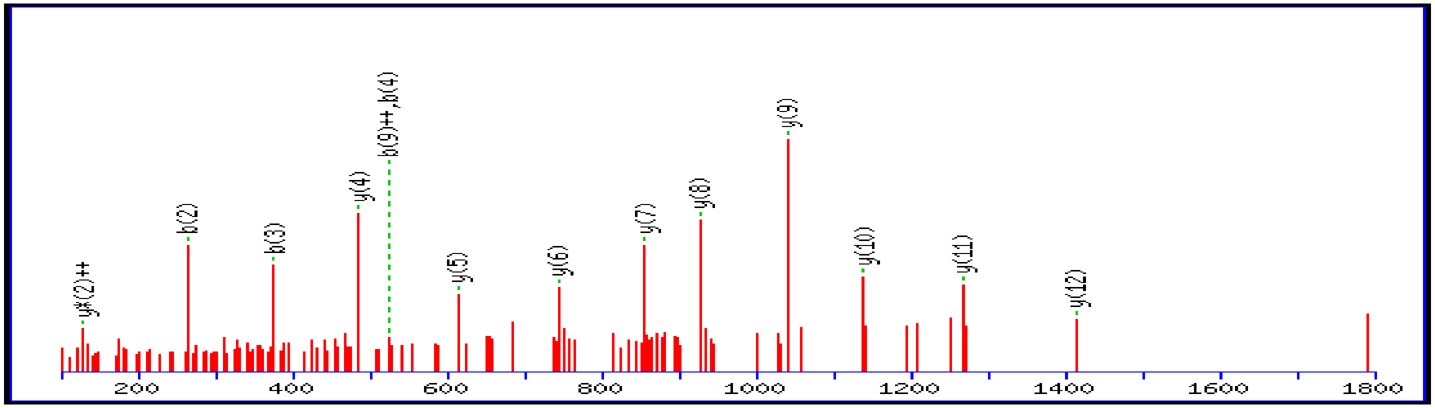

Monoisotopic mass of neutral peptide Mr(calc): 1788.9080
Fixed modifications: Carbamidomethyl (C) (apply to specified residues or termini only)
Variable modifications:
M2 : Oxidation (M), with neutral losses 0.0000(shown in table), 63.9983

Monoisotopic mass of neutral peptide Mr(calc): 1788.9080
Fixed modifications: Carbamidomethyl (C) (apply to specified residues or termini only)
Variable modifications:
M2 : Oxidation (M), with neutral losses 0.0000(shown in table), 63.9983

Ions Score: 90 Expect: 1.5e-07
Matches : 14/230 fragment ions using 13 most intense peaks (help)

#	b	b^{++}	b^*	b^{*++}	b^0	b^{0++}	Seq.	y	y^{++}	y^*	y^{*++}	y^0	y^{0++}	#
1	116.0342	58.5207			98.0237	49.5155	D							15
2	263.0696	132.0384			245.0591	123.0332	M	1674.8884	837.9478	1657.8618	829.4345	1656.8778	828.9425	14
3	376.1537	188.5805			358.1431	179.5752	L	1527.8530	764.4301	1510.8264	755.9168	1509.8424	755.4248	13
4	523.2221	262.1147			505.2115	253.1094	F	*1414.7689*	707.8881	1397.7423	699.3748	1396.7583	698.8828	12
5	651.2807	326.1440	634.2541	317.6307	633.2701	317.1387	Q	*1267.7005*	634.3539	1250.6739	625.8406	1249.6899	625.3486	11
6	750.3491	375.6782	733.3225	367.1649	732.3385	366.6729	V	*1139.6419*	570.3246	1122.6154	561.8113	1121.6313	561.3193	10
7	863.4332	432.2202	846.4066	423.7069	845.4226	423.2149	L	*1040.5735*	520.7904	1023.5469	512.2771	1022.5629	511.7851	9
8	934.4703	467.7388	917.4437	459.2255	916.4597	458.7335	A	*927.4894*	464.2483	910.4629	455.7351	909.4789	455.2431	8
9	1047.5543	524.2808	1030.5278	515.7675	1029.5438	515.2755	L	*856.4523*	428.7298	839.4258	420.2165	838.4417	419.7245	7
10	1176.5969	588.8021	1159.5704	580.2888	1158.5864	579.7968	E	*743.3682*	372.1878	726.3417	363.6745	725.3577	363.1825	6
11	1305.6395	653.3234	1288.6130	644.8101	1287.6290	644.3181	E	*614.3257*	307.6665	597.2991	299.1532	596.3151	298.6612	5
12	1402.6923	701.8498	1385.6657	693.3365	1384.6817	692.8445	P	*485.2831*	243.1452	468.2565	234.6319			4
13	1516.7352	758.8712	1499.7087	750.3580	1498.7246	749.8660	N	388.2303	194.6188	371.2037	186.1055			3
14	1615.8036	808.4054	1598.7771	799.8922	1597.7931	799.4002	V	274.1874	137.5973	257.1608	129.0840			2
15							R	175.1190	88.0631	158.0924	79.5498			1

MS/MS Fragmentation of LYIGLAGLATDVQTVAQR
Found in PSB3_HUMAN in SwissProt, Proteasome subunit beta type-3 OS=Homo sapiens GN=PSMB3 PE=1 SV=2

Match to Query 418: 1887.801248 from(944.907900,2+) intensity(3847.3000) index(441)
Data file IS_111911_23b.txt

Click mouse within plot area to zoom in by factor of two about that point
Or, Plot from 100 to 1900 Da Full range
Label all possible matches ○ Label matches used for scoring ◉
Show Y-axis ☐

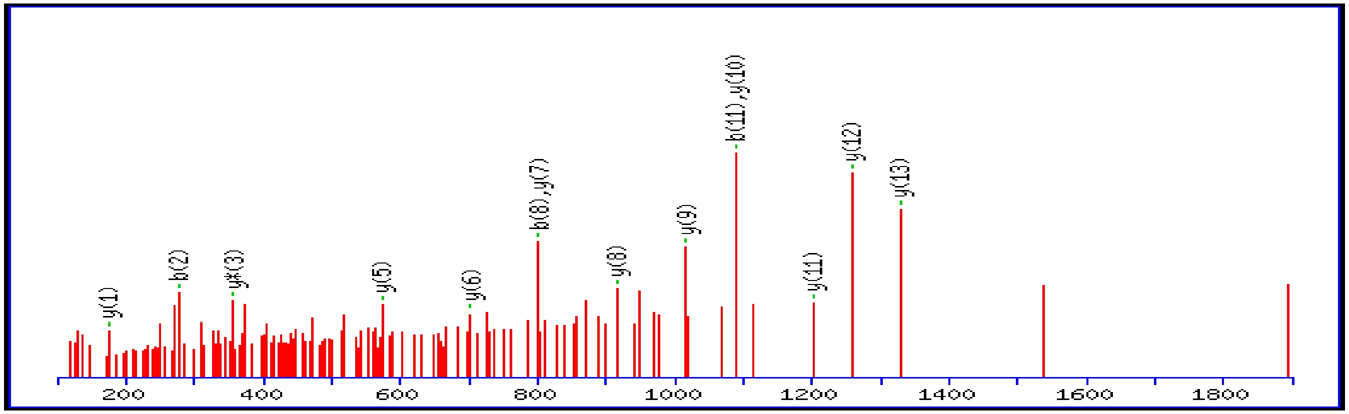

Monoisotopic mass of neutral peptide Mr(calc): 1888.0418
Fixed modifications: Carbamidomethyl (C) (apply to specified residues or termini only)
Ions Score: 88 Expect: 2.1e-07
Matches : 14/154 fragment ions using 14 most intense peaks (help)

#	b	b^{++}	b^*	b^{*++}	b^0	b^{0++}	Seq.	y	y^{++}	y^*	y^{*++}	y^0	y^{0++}	#
1	114.0913	57.5493					L							18
2	277.1547	139.0810					Y	1775.9650	888.4862	1758.9385	879.9729	1757.9545	879.4809	17
3	390.2387	195.6230					I	1612.9017	806.9545	1595.8752	798.4412	1594.8911	797.9492	16
4	447.2602	224.1337					G	1499.8176	750.4125	1482.7911	741.8992	1481.8071	741.4072	15
5	560.3443	280.6758					L	1442.7962	721.9017	1425.7696	713.3884	1424.7856	712.8964	14
6	631.3814	316.1943					A	*1329.7121*	665.3597	1312.6856	656.8464	1311.7015	656.3544	13
7	688.4028	344.7051					G	*1258.6750*	629.8411	1241.6484	621.3279	1240.6644	620.8359	12
8	801.4869	401.2471					L	*1201.6535*	601.3304	1184.6270	592.8171	1183.6430	592.3251	11
9	872.5240	436.7656					A	*1088.5695*	544.7884	1071.5429	536.2751	1070.5589	535.7831	10
10	973.5717	487.2895			955.5611	478.2842	T	*1017.5324*	509.2698	1000.5058	500.7565	999.5218	500.2645	9
11	1088.5986	544.8030			1070.5881	535.7977	D	*916.4847*	458.7460	899.4581	450.2327	898.4741	449.7407	8
12	1187.6671	594.3372			1169.6565	585.3319	V	*801.4577*	401.2325	784.4312	392.7192	783.4472	392.2272	7
13	1315.7256	658.3665	1298.6991	649.8532	1297.7151	649.3612	Q	*702.3893*	351.6983	685.3628	343.1850	684.3787	342.6930	6
14	1416.7733	708.8903	1399.7468	700.3770	1398.7627	699.8850	T	*574.3307*	287.6690	557.3042	279.1557	556.3202	278.6637	5
15	1515.8417	758.4245	1498.8152	749.9112	1497.8312	749.4192	V	473.2831	237.1452	456.2565	228.6319			4
16	1586.8788	793.9431	1569.8523	785.4298	1568.8683	784.9378	A	374.2146	187.6110	357.1881	179.0977			3
17	1714.9374	857.9723	1697.9109	849.4591	1696.9269	848.9671	Q	303.1775	152.0924	286.1510	143.5791			2
18							R	*175.1190*	88.0631	158.0924	79.5498			1

MS/MS Fragmentation of VIGNQSLVNELAFTAR
Found in ECH1_HUMAN in SwissProt, Delta(3,5)-Delta(2,4)-dienoyl-CoA isomerase, mitochondrial OS=Homo sapiens GN=ECH1 PE=1 SV=2

Match to Query 386: 1730.868848 from(866.441700,2+) intensity(5395.8000) index(393)
Data file IS_111911_23b.txt

Click mouse within plot area to zoom in by factor of two about that point
Or, Plot from 100 to 1800 Da Full range
Label all possible matches ○ Label matches used for scoring ◉
Show Y-axis ☐

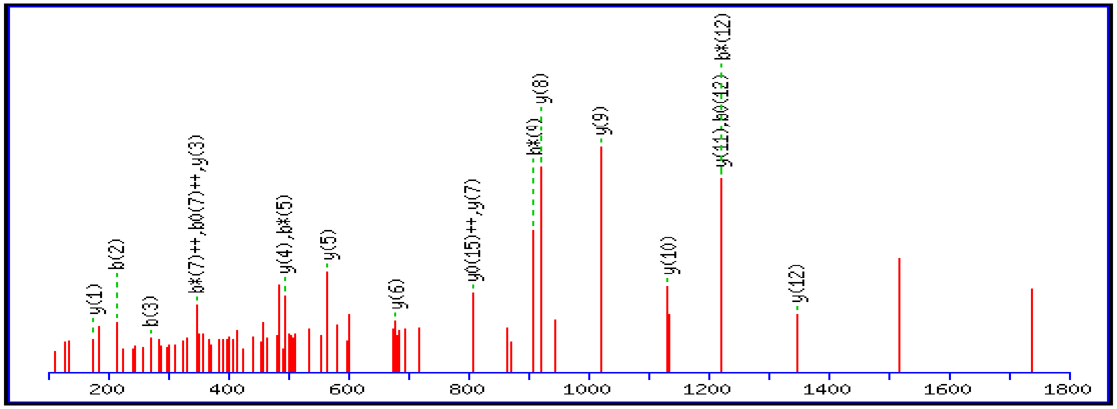


```
Monoisotopic mass of neutral peptide Mr(calc): 1730.9315
Fixed modifications: Carbamidomethyl (C) (apply to specified residues or termini only)
Ions Score: 85  Expect: 5.1e-07
Matches : 20/160 fragment ions using 25 most intense peaks  (help)
```

#	b	b^{++}	b*	b^{*++}	b^0	b^{0++}	Seq.	y	y^{++}	y*	y^{*++}	y^0	y^{0++}	#
1	100.0757	50.5415					V							16
2	213.1598	107.0835					I	1632.8704	816.9388	1615.8438	808.4256	1614.8598	807.9336	15
3	270.1812	135.5942					G	1519.7863	760.3968	1502.7598	751.8835	1501.7758	751.3915	14
4	384.2241	192.6157	367.1976	184.1024			N	1462.7649	731.8861	1445.7383	723.3728	1444.7543	722.8808	13
5	512.2827	256.6450	495.2562	248.1317			Q	1348.7219	674.8646	1331.6954	666.3513	1330.7114	665.8593	12
6	599.3148	300.1610	582.2882	291.6477	581.3042	291.1557	S	1220.6634	610.8353	1203.6368	602.3220	1202.6528	601.8300	11
7	712.3988	356.7030	695.3723	348.1898	694.3883	347.6978	L	1133.6313	567.3193	1116.6048	558.8060	1115.6208	558.3140	10
8	811.4672	406.2373	794.4407	397.7240	793.4567	397.2320	V	1020.5473	510.7773	1003.5207	502.2640	1002.5367	501.7720	9
9	925.5102	463.2587	908.4836	454.7454	907.4996	454.2534	N	921.4789	461.2431	904.4523	452.7298	903.4683	452.2378	8
10	1054.5527	527.7800	1037.5262	519.2667	1036.5422	518.7747	E	807.4359	404.2216	790.4094	395.7083	789.4254	395.2163	7
11	1167.6368	584.3220	1150.6103	575.8088	1149.6262	575.3168	L	678.3933	339.7003	661.3668	331.1870	660.3828	330.6950	6
12	1238.6739	619.8406	1221.6474	611.3273	1220.6634	610.8353	A	565.3093	283.1583	548.2827	274.6450	547.2987	274.1530	5
13	1385.7423	693.3748	1368.7158	684.8615	1367.7318	684.3695	F	494.2722	247.6397	477.2456	239.1264	476.2616	238.6344	4
14	1486.7900	743.8986	1469.7635	735.3854	1468.7795	734.8934	T	347.2037	174.1055	330.1772	165.5922	329.1932	165.1002	3
15	1557.8271	779.4172	1540.8006	770.9039	1539.8166	770.4119	A	246.1561	123.5817	229.1295	115.0684			2
16							R	175.1190	88.0631	158.0924	79.5498			1

MS/MS Fragmentation of **ILTFDQLALDSPK**
Found in **RL18_HUMAN** in **SwissProt**, 60S ribosomal protein L18 OS=Homo sapiens GN=RPL18 PE=1 SV=2

Match to Query 319: 1460.015448 from(731.015000,2+) intensity(11291.0000) index(93)
Data file IS_111911_23b.txt

Click mouse within plot area to zoom in by factor of two about that point
Or, Plot from 100 to 1500 Da Full range
Label all possible matches ○ Label matches used for scoring ◉
Show Y-axis ☐

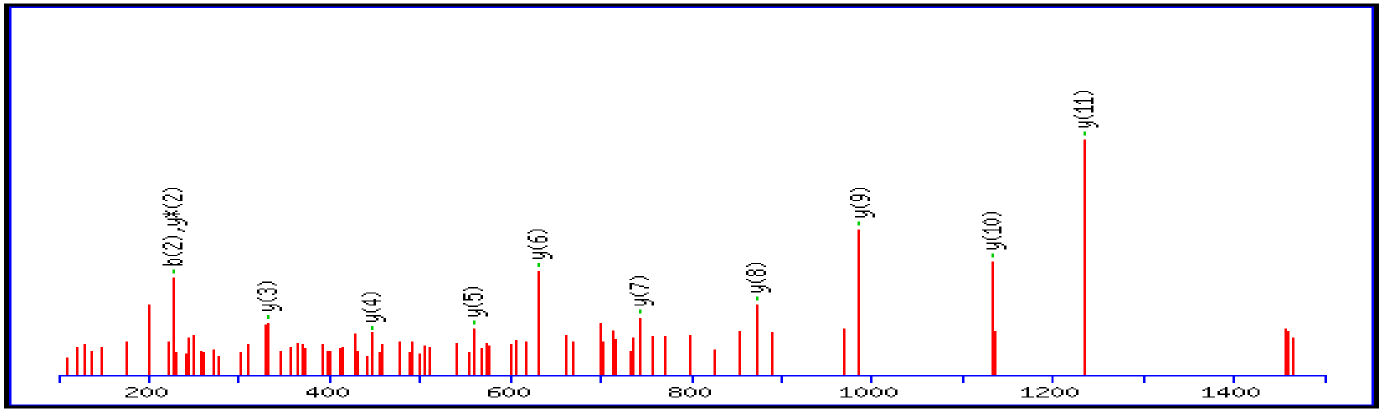

Monoisotopic mass of neutral peptide Mr(calc): 1459.7922
Fixed modifications: Carbamidomethyl (C) (apply to specified residues or termini only)
Ions Score: 83 Expect: 8.8e-07
Matches : 11/126 fragment ions using 12 most intense peaks (help)

#	b	b^{++}	b^{*}	b^{*++}	b^{0}	b^{0++}	Seq.	y	y^{++}	y^{*}	y^{*++}	y^{0}	y^{0++}	#
1	114.0913	57.5493					I							13
2	227.1754	114.0913					L	1347.7155	674.3614	1330.6889	665.8481	1329.7049	665.3561	12
3	328.2231	164.6152			310.2125	155.6099	T	1234.6314	617.8193	1217.6048	609.3061	1216.6208	608.8141	11
4	475.2915	238.1494			457.2809	229.1441	F	1133.5837	567.2955	1116.5572	558.7822	1115.5732	558.2902	10
5	590.3184	295.6629			572.3079	286.6576	D	986.5153	493.7613	969.4888	485.2480	968.5047	484.7560	9
6	718.3770	359.6921	701.3505	351.1789	700.3665	350.6869	Q	871.4884	436.2478	854.4618	427.7345	853.4778	427.2425	8
7	831.4611	416.2342	814.4345	407.7209	813.4505	407.2289	L	743.4298	372.2185	726.4032	363.7053	725.4192	363.2132	7
8	902.4982	451.7527	885.4716	443.2395	884.4876	442.7475	A	630.3457	315.6765	613.3192	307.1632	612.3352	306.6712	6
9	1015.5823	508.2948	998.5557	499.7815	997.5717	499.2895	L	559.3086	280.1579	542.2821	271.6447	541.2980	271.1527	5
10	1130.6092	565.8082	1113.5827	557.2950	1112.5986	556.8030	D	446.2245	223.6159	429.1980	215.1026	428.2140	214.6106	4
11	1217.6412	609.3243	1200.6147	600.8110	1199.6307	600.3190	S	331.1976	166.1024	314.1710	157.5892	313.1870	157.0972	3
12	1314.6940	657.8506	1297.6674	649.3374	1296.6834	648.8454	P	244.1656	122.5864	227.1390	114.0731			2
13							K	147.1128	74.0600	130.0863	65.5468			1

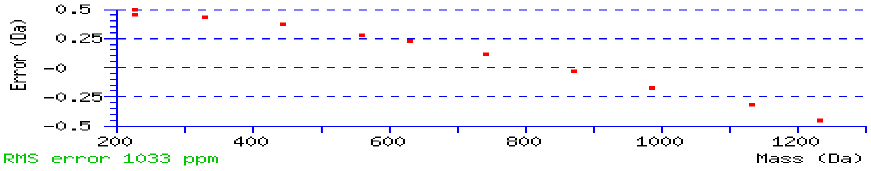

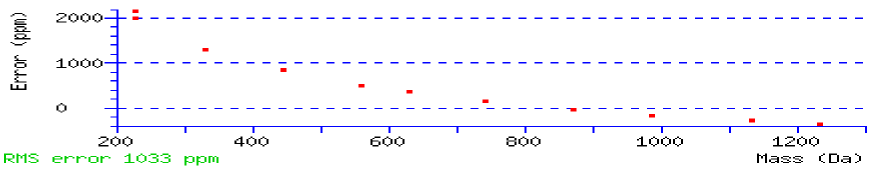

MS/MS Fragmentation of AMGIMNSFVNDIFER
Found in H2B1B_HUMAN in SwissProt, Histone H2B type 1-B OS=Homo sapiens GN=HIST1H2BB PE=1 SV=2

Match to Query 393: 1774.687648 from(888.351100,2+) intensity(8696.5000) index(403)
Data file IS_111911_23b.txt

Click mouse within plot area to zoom in by factor of two about that point
Or, Plot from 100 to 1800 Da Full range
Label all possible matches ○ Label matches used for scoring ◉
Show Y-axis ☐

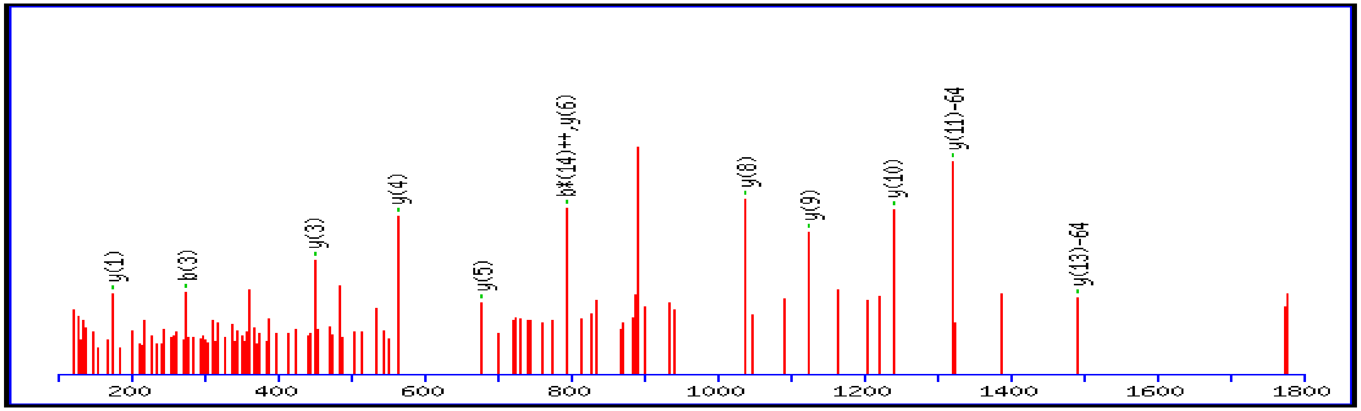

Monoisotopic mass of neutral peptide Mr(calc): 1774.8018
Fixed modifications: Carbamidomethyl (C) (apply to specified residues or termini only)
Variable modifications:
M2 : Oxidation (M), with neutral losses 63.9983(shown in table), 0.0000
M5 : Oxidation (M), with neutral losses 63.9983(shown in table), 0.0000
Ions Score: 82 Expect: 9.4e-07
Matches : 12/228 fragment ions using 14 most intense peaks (help)

#	b	b^{++}	b^{*}	b^{*++}	b^{0}	b^{0++}	Seq.	y	y^{++}	y^{*}	y^{*++}	y^{0}	y^{0++}	#
1	72.0444	36.5258					A							15
2	155.0815	78.0444					M	1576.7754	788.8914	1559.7489	780.3781	1558.7649	779.8861	14
3	212.1030	106.5551					G	*1493.7383*	747.3728	1476.7118	738.8595	1475.7278	738.3675	13
4	325.1870	163.0972					I	1436.7169	718.8621	1419.6903	710.3488	1418.7063	709.8568	12
5	408.2241	204.6157					M	*1323.6328*	662.3200	1306.6062	653.8068	1305.6222	653.3148	11
6	522.2671	261.6372	505.2405	253.1239			N	*1240.5957*	620.8015	1223.5691	612.2882	1222.5851	611.7962	10
7	609.2991	305.1532	592.2726	296.6399	591.2885	296.1479	S	*1126.5527*	563.7800	1109.5262	555.2667	1108.5422	554.7747	9
8	756.3675	378.6874	739.3410	370.1741	738.3570	369.6821	F	*1039.5207*	520.2640	1022.4942	511.7507	1021.5102	511.2587	8
9	855.4359	428.2216	838.4094	419.7083	837.4254	419.2163	V	892.4523	446.7298	875.4258	438.2165	874.4417	437.7245	7
10	969.4789	485.2431	952.4523	476.7298	951.4683	476.2378	N	*793.3839*	397.1956	776.3573	388.6823	775.3733	388.1903	6
11	1084.5058	542.7565	1067.4793	534.2433	1066.4952	533.7513	D	*679.3410*	340.1741	662.3144	331.6608	661.3304	331.1688	5
12	1197.5899	599.2986	1180.5633	590.7853	1179.5793	590.2933	I	*564.3140*	282.6606	547.2875	274.1474	546.3035	273.6554	4
13	1344.6583	672.8328	1327.6317	664.3195	1326.6477	663.8275	F	*451.2300*	226.1186	434.2034	217.6053	433.2194	217.1133	3
14	1473.7009	737.3541	1456.6743	728.8408	1455.6903	728.3488	E	304.1615	152.5844	287.1350	144.0711	286.1510	143.5791	2
15							R	*175.1190*	88.0631	158.0924	79.5498			1

MS/MS Fragmentation of SQGGEPTYNVAVGR
Found in PROF2_HUMAN in SwissProt, Profilin-2 OS=Homo sapiens GN=PFN2 PE=1 SV=3

Match to Query 308: 1433.924448 from(717.969500,2+) intensity(2315.2000) index(323)
Data file IS_111911_23b.txt

Click mouse within plot area to zoom in by factor of two about that point
Or, Plot from 200 to 1500 Da Full range
Label all possible matches ○ Label matches used for scoring ◉
Show Y-axis ☐

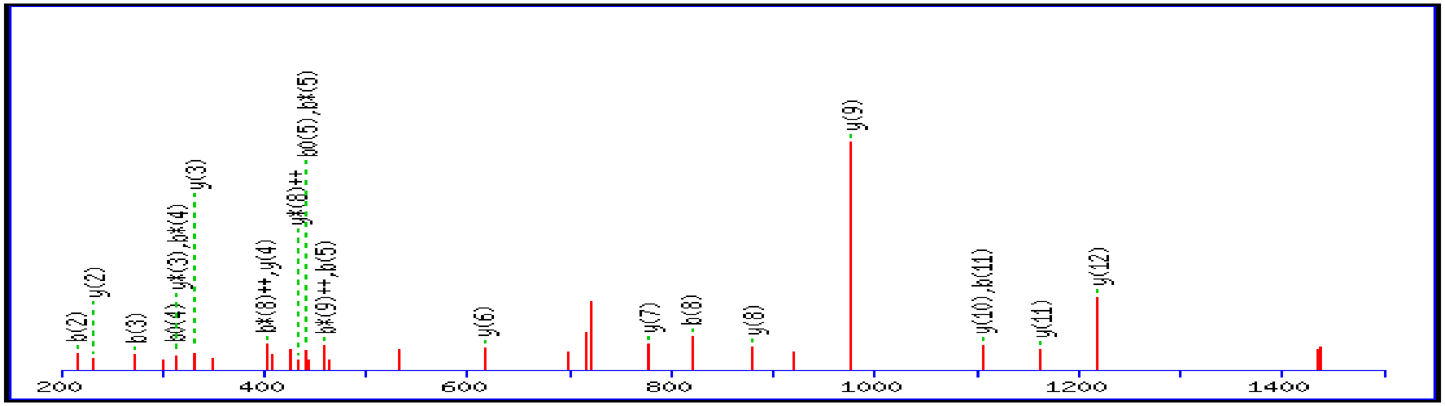

Monoisotopic mass of neutral peptide Mr(calc): 1433.6899
Fixed modifications: Carbamidomethyl (C) (apply to specified residues or termini only)
Ions Score: 82 Expect: 1.2e-06
Matches : 23/140 fragment ions using 31 most intense peaks (help)

#	b	b^{++}	b^{*}	b^{*++}	b^{0}	b^{0++}	Seq.	y	y^{++}	y^{*}	y^{*++}	y^{0}	y^{0++}	#
1	88.0393	44.5233			70.0287	35.5180	S							14
2	216.0979	108.5526	199.0713	100.0393	198.0873	99.5473	Q	1347.6652	674.3362	1330.6386	665.8229	1329.6546	665.3309	13
3	273.1193	137.0633	256.0928	128.5500	255.1088	128.0580	G	1219.6066	610.3069	1202.5800	601.7937	1201.5960	601.3016	12
4	330.1408	165.5740	313.1143	157.0608	312.1302	156.5688	G	1162.5851	581.7962	1145.5586	573.2829	1144.5745	572.7909	11
5	459.1834	230.0953	442.1569	221.5821	441.1728	221.0901	E	1105.5637	553.2855	1088.5371	544.7722	1087.5531	544.2802	10
6	556.2362	278.6217	539.2096	270.1084	538.2256	269.6164	P	976.5211	488.7642	959.4945	480.2509	958.5105	479.7589	9
7	657.2838	329.1456	640.2573	320.6323	639.2733	320.1403	T	879.4683	440.2378	862.4417	431.7245	861.4577	431.2325	8
8	820.3472	410.6772	803.3206	402.1640	802.3366	401.6719	Y	778.4206	389.7139	761.3941	381.2007			7
9	934.3901	467.6987	917.3636	459.1854	916.3795	458.6934	N	615.3573	308.1823	598.3307	299.6690			6
10	1033.4585	517.2329	1016.4320	508.7196	1015.4480	508.2276	V	501.3144	251.1608	484.2878	242.6475			5
11	1104.4956	552.7515	1087.4691	544.2382	1086.4851	543.7462	A	402.2459	201.6266	385.2194	193.1133			4
12	1203.5640	602.2857	1186.5375	593.7724	1185.5535	593.2804	V	331.2088	166.1081	314.1823	157.5948			3
13	1260.5855	630.7964	1243.5590	622.2831	1242.5749	621.7911	G	232.1404	116.5738	215.1139	108.0606			2
14							R	175.1190	88.0631	158.0924	79.5498			1

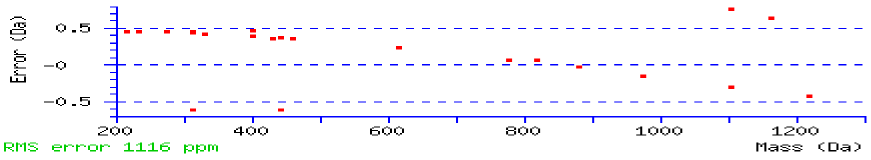

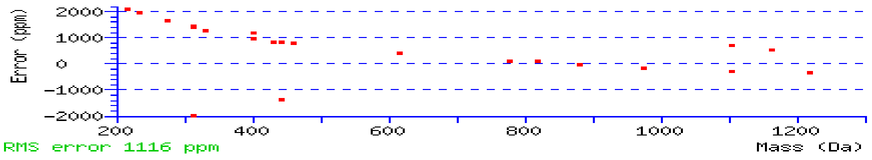

MS/MS Fragmentation of **DYGVLLEGSGLALR**
Found in **PRDX3_HUMAN** in **SwissProt**, Thioredoxin-dependent peroxide reductase, mitochondrial OS=Homo sapiens GN=PRDX3 PE=1 SV=3

Match to Query 323: 1462.011048 from(732.012800,2+) intensity(10740.6000) index(402)
Data file IS_111911_23b.txt

Click mouse within plot area to zoom in by factor of two about that point
Or, Plot from 100 to 1500 Da Full range
Label all possible matches ○ Label matches used for scoring ◉
Show Y-axis ☐

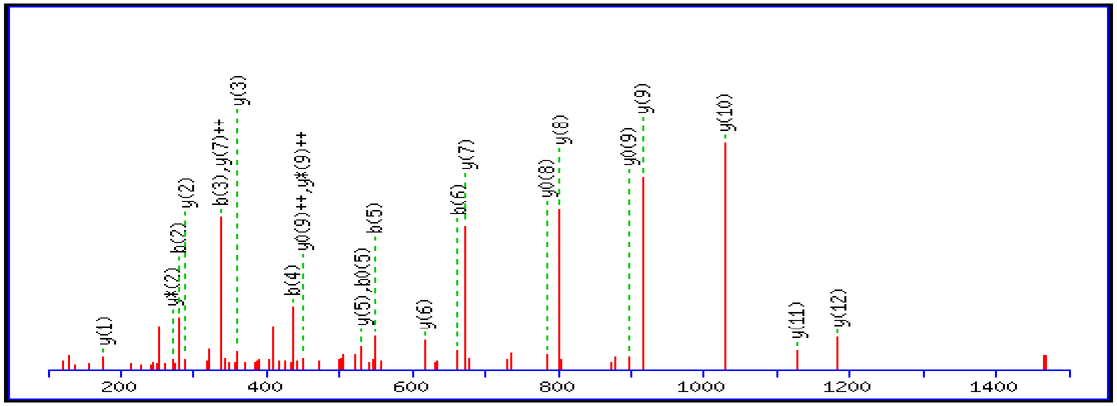

Monoisotopic mass of neutral peptide Mr(calc): 1461.7827
Fixed modifications: Carbamidomethyl (C) (apply to specified residues or termini only)
Ions Score: 82 Expect: 1.2e-06
Matches : 23/120 fragment ions using 35 most intense peaks (help)

#	b	b^{++}	b^{0}	b^{0++}	Seq.	y	y^{++}	y^{*}	y^{*++}	y^{0}	y^{0++}	#
1	116.0342	58.5207	98.0237	49.5155	D							14
2	279.0975	140.0524	261.0870	131.0471	Y	1347.7631	674.3852	1330.7365	665.8719	1329.7525	665.3799	13
3	336.1190	168.5631	318.1084	159.5579	G	1184.6997	592.8535	1167.6732	584.3402	1166.6892	583.8482	12
4	435.1874	218.0974	417.1769	209.0921	V	1127.6783	564.3428	1110.6517	555.8295	1109.6677	555.3375	11
5	548.2715	274.6394	530.2609	265.6341	L	1028.6099	514.8086	1011.5833	506.2953	1010.5993	505.8033	10
6	661.3556	331.1814	643.3450	322.1761	L	915.5258	458.2665	898.4993	449.7533	897.5152	449.2613	9
7	790.3981	395.7027	772.3876	386.6974	E	802.4417	401.7245	785.4152	393.2112	784.4312	392.7192	8
8	847.4196	424.2134	829.4090	415.2082	G	673.3991	337.2032	656.3726	328.6899	655.3886	328.1979	7
9	934.4516	467.7295	916.4411	458.7242	S	616.3777	308.6925	599.3511	300.1792	598.3671	299.6872	6
10	991.4731	496.2402	973.4625	487.2349	G	529.3457	265.1765	512.3191	256.6632			5
11	1104.5572	552.7822	1086.5466	543.7769	L	472.3242	236.6657	455.2976	228.1525			4
12	1175.5943	588.3008	1157.5837	579.2955	A	359.2401	180.1237	342.2136	171.6104			3
13	1288.6783	644.8428	1270.6678	635.8375	L	288.2030	144.6051	271.1765	136.0919			2
14					R	175.1190	88.0631	158.0924	79.5498			1

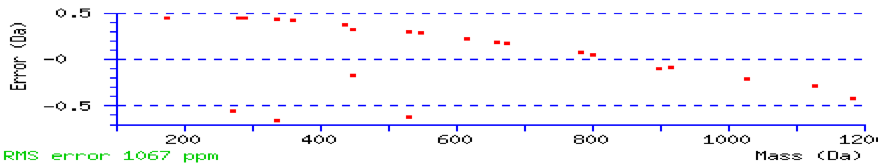

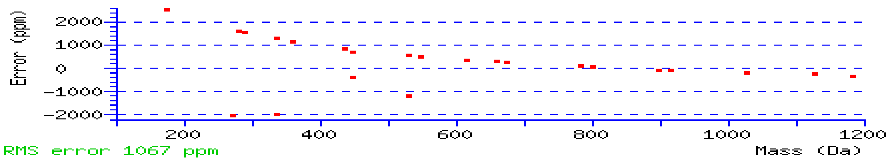

MS/MS Fragmentation of **SLAMEMVLTGDR**
Found in **ECHM_HUMAN** in **SwissProt**, Enoyl-CoA hydratase, mitochondrial OS=Homo sapiens GN=ECHS1 PE=1 SV=4

Match to Query 278: 1353.964248 from(677.989400,2+) intensity(1186.5000) index(334)
Data file IS_111911_23b.txt

Click mouse within plot area to zoom in by factor of two about that point
Or, Plot from 100 to 1400 Da Full range
Label all possible matches ○ Label matches used for scoring ◉
Show Y-axis ☐

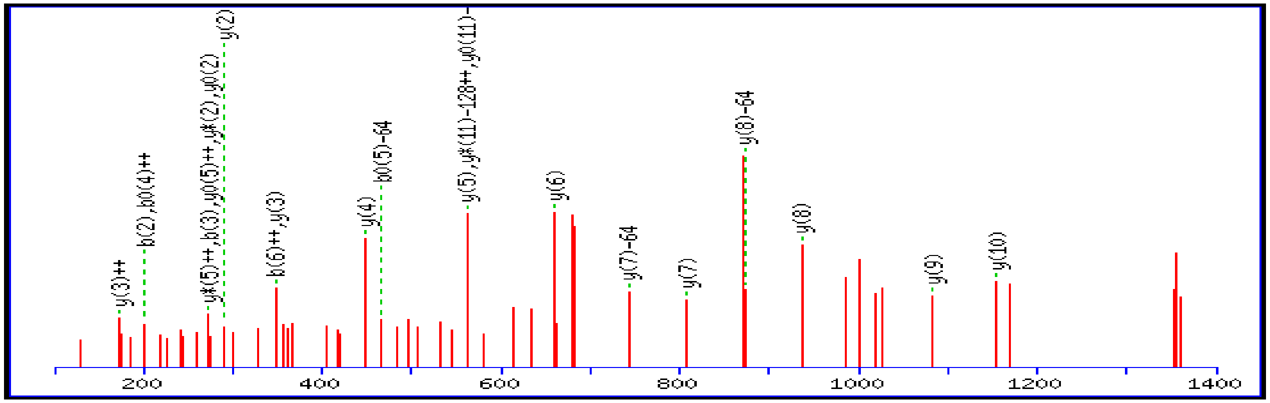

Monoisotopic mass of neutral peptide Mr(calc): 1353.6268
Fixed modifications: Carbamidomethyl (C) (apply to specified residues or termini only)
Variable modifications:
M4 : Oxidation (M), with neutral losses 0.0000(shown in table), 63.9983
M6 : Oxidation (M), with neutral losses 0.0000(shown in table), 63.9983
Ions Score: 70 Expect: 1.5e-05
Matches : 23/170 fragment ions using 21 most intense peaks (help)

#	b	b^{++}	b^{0}	b^{0++}	Seq.	y	y^{++}	y^{*}	y^{*++}	y^{0}	y^{0++}	#
1	88.0393	44.5233	70.0287	35.5180	S							12
2	201.1234	101.0653	183.1128	92.0600	L	1267.6021	634.3047	1250.5755	625.7914	1249.5915	625.2994	11
3	272.1605	136.5839	254.1499	127.5786	A	1154.5180	577.7626	1137.4915	569.2494	1136.5075	568.7574	10
4	419.1959	210.1016	401.1853	201.0963	M	1083.4809	542.2441	1066.4544	533.7308	1065.4703	533.2388	9
5	548.2385	274.6229	530.2279	265.6176	E	936.4455	468.7264	919.4190	460.2131	918.4349	459.7211	8
6	695.2739	348.1406	677.2633	339.1353	M	807.4029	404.2051	790.3764	395.6918	789.3924	395.1998	7
7	794.3423	397.6748	776.3317	388.6695	V	660.3675	330.6874	643.3410	322.1741	642.3570	321.6821	6
8	907.4264	454.2168	889.4158	445.2115	L	561.2991	281.1532	544.2726	272.6399	543.2885	272.1479	5
9	1008.4740	504.7407	990.4635	495.7354	T	448.2150	224.6112	431.1885	216.0979	430.2045	215.6059	4
10	1065.4955	533.2514	1047.4849	524.2461	G	347.1674	174.0873	330.1408	165.5740	329.1568	165.0820	3
11	1180.5224	590.7649	1162.5119	581.7596	D	290.1459	145.5766	273.1193	137.0633	272.1353	136.5713	2
12					R	175.1190	88.0631	158.0924	79.5498			1

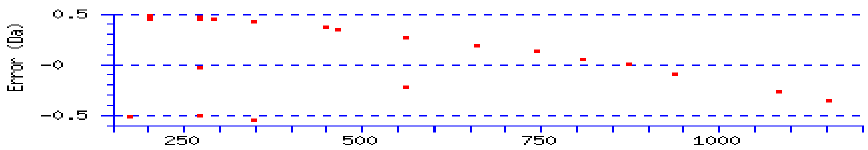

RMS error 1318 ppm

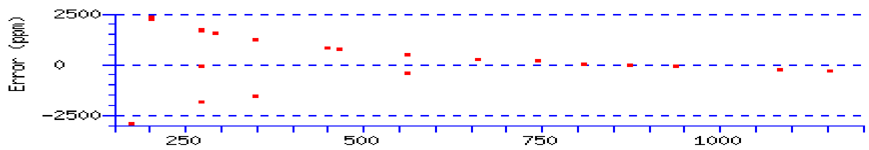

RMS error 1318 ppm

MS/MS Fragmentation of **VLDELTLTK**
Found in **K1C10_HUMAN** in **SwissProt**, Keratin, type I cytoskeletal 10 OS=Homo sapiens GN=KRT10 PE=1 SV=6

Match to Query 142: 1031.201048 from(516.607800,2+) intensity(3361.3000) index(352)
Data file IS_111911_23b.txt

Click mouse within plot area to zoom in by factor of two about that point
Or, Plot from 100 to 950 Da Full range
Label all possible matches ○ Label matches used for scoring ◉
Show Y-axis ☐

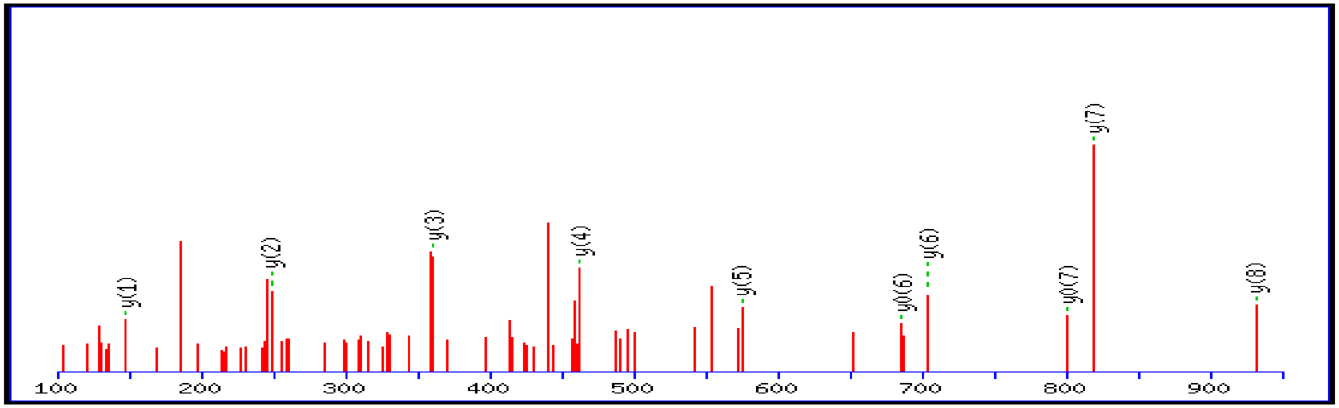

Monoisotopic mass of neutral peptide Mr(calc): 1030.5910
Fixed modifications: Carbamidomethyl (C) (apply to specified residues or termini only)
Ions Score: 65 Expect: 6e-05
Matches : 10/74 fragment ions using 16 most intense peaks (help)

#	b	b^{++}	b^0	b^{0++}	Seq.	y	y^{++}	y^*	y^{*++}	y^0	y^{0++}	#
1	100.0757	50.5415			V							9
2	213.1598	107.0835			L	*932.5299*	466.7686	915.5033	458.2553	914.5193	457.7633	8
3	328.1867	164.5970	310.1761	155.5917	D	*819.4458*	410.2266	802.4193	401.7133	801.4353	401.2213	7
4	457.2293	229.1183	439.2187	220.1130	E	*704.4189*	352.7131	687.3923	344.1998	686.4083	343.7078	6
5	570.3134	285.6603	552.3028	276.6550	L	*575.3763*	288.1918	558.3497	279.6785	557.3657	279.1865	5
6	671.3610	336.1842	653.3505	327.1789	T	*462.2922*	231.6498	445.2657	223.1365	444.2817	222.6445	4
7	784.4451	392.7262	766.4345	383.7209	L	*361.2445*	181.1259	344.2180	172.6126	343.2340	172.1206	3
8	885.4928	443.2500	867.4822	434.2447	T	*248.1605*	124.5839	231.1339	116.0706	230.1499	115.5786	2
9					K	*147.1128*	74.0600	130.0863	65.5468			1

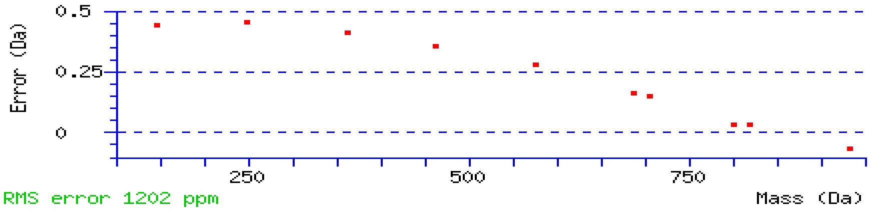

RMS error 1202 ppm

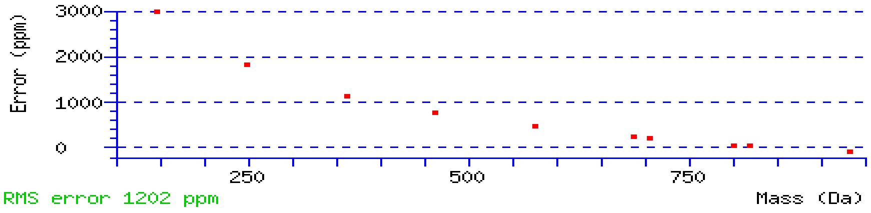

RMS error 1202 ppm

MS/MS Fragmentation of **FLFPFFDSAYQGFASGNLER**
Found in **AATC_HUMAN** in **SwissProt**, Aspartate aminotransferase, cytoplasmic OS=Homo sapiens GN=GOT1 PE=1 SV=3

Match to Query 520: 2311.349448 from(1156.682000,2+) intensity(4194.1000) index(176)
Data file IS_111911_23b.txt

Click mouse within plot area to zoom in by factor of two about that point
Or, Plot from 100 to 1600 Da Full range
Label all possible matches ○ Label matches used for scoring ◉
Show Y-axis ☐

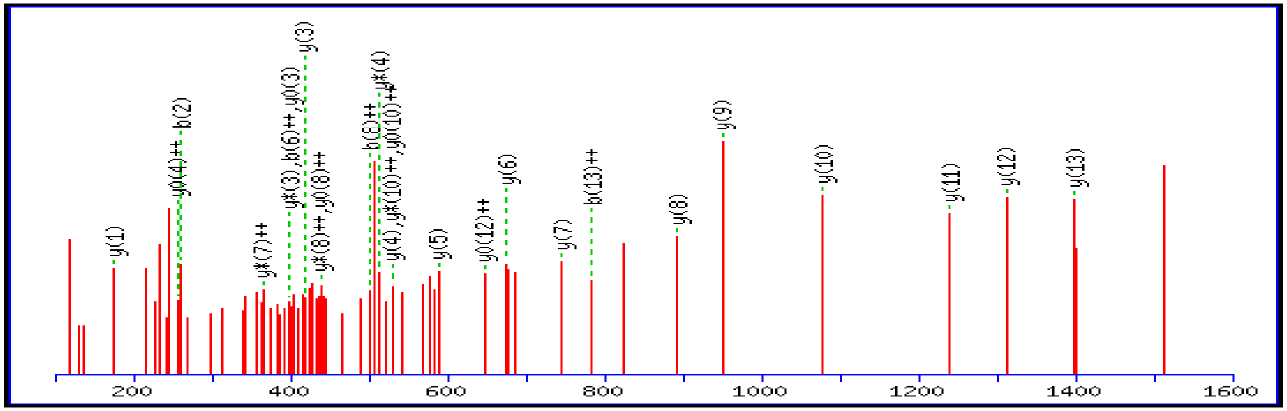


```
Monoisotopic mass of neutral peptide Mr(calc): 2312.0902
Fixed modifications: Carbamidomethyl (C) (apply to specified residues or termini only)
Ions Score: 64  Expect: 3.8e-05
Matches : 26/194 fragment ions using 44 most intense peaks   (help)
```

#	b	b^{++}	b^{*}	b^{*++}	b^{0}	b^{0++}	Seq.	y	y^{++}	y^{*}	y^{*++}	y^{0}	y^{0++}	#
1	148.0757	74.5415					F							20
2	261.1598	131.0835					L	2166.0291	1083.5182	2149.0025	1075.0049	2148.0185	1074.5129	19
3	408.2282	204.6177					F	2052.9450	1026.9761	2035.9185	1018.4629	2034.9345	1017.9709	18
4	505.2809	253.1441					P	1905.8766	953.4419	1888.8501	944.9287	1887.8660	944.4367	17
5	652.3493	326.6783					F	1808.8238	904.9156	1791.7973	896.4023	1790.8133	895.9103	16
6	799.4178	**400.2125**					F	1661.7554	831.3814	1644.7289	822.8681	1643.7449	822.3761	15
7	914.4447	457.7260			896.4341	448.7207	D	1514.6870	757.8471	1497.6605	749.3339	1496.6764	748.8419	14
8	1001.4767	**501.2420**			983.4662	492.2367	S	***1399.6601***	700.3337	1382.6335	691.8204	1381.6495	691.3284	13
9	1072.5138	536.7606			1054.5033	527.7553	A	***1312.6280***	656.8177	1295.6015	648.3044	1294.6175	647.8124	12
10	1235.5772	618.2922			1217.5666	609.2869	Y	***1241.5909***	621.2991	1224.5644	612.7858	1223.5804	612.2938	11
11	1363.6358	682.3215	1346.6092	673.8082	1345.6252	673.3162	Q	***1078.5276***	539.7674	1061.5010	531.2542	1060.5170	530.7622	10
12	1420.6572	710.8322	1403.6307	702.3190	1402.6467	701.8270	G	***950.4690***	475.7381	933.4425	467.2249	932.4585	466.7329	9
13	1567.7256	**784.3665**	1550.6991	775.8532	1549.7151	775.3612	F	***893.4476***	447.2274	876.4210	438.7141	875.4370	438.2221	8
14	1638.7627	819.8850	1621.7362	811.3717	1620.7522	810.8797	A	***746.3791***	373.6932	729.3526	365.1799	728.3686	364.6879	7
15	1725.7948	863.4010	1708.7682	854.8877	1707.7842	854.3957	S	***675.3420***	338.1747	658.3155	329.6614	657.3315	329.1694	6
16	1782.8162	891.9118	1765.7897	883.3985	1764.8057	882.9065	G	***588.3100***	294.6586	571.2835	286.1454	570.2994	285.6534	5
17	1896.8592	948.9332	1879.8326	940.4199	1878.8486	939.9279	N	***531.2885***	266.1479	514.2620	257.6346	513.2780	257.1426	4
18	2009.9432	1005.4753	1992.9167	996.9620	1991.9327	996.4700	L	***417.2456***	209.1264	400.2191	200.6132	399.2350	200.1212	3
19	2138.9858	1069.9965	2121.9593	1061.4833	2120.9753	1060.9913	E	304.1615	152.5844	287.1350	144.0711	286.1510	143.5791	2
20							R	***175.1190***	88.0631	158.0924	79.5498			1

MS/MS Fragmentation of **NVFDEAILAALEPPEPK**
Found in **CDC42_HUMAN** in **SwissProt**, Cell division control protein 42 homolog OS=Homo sapiens GN=CDC42 PE=1 SV=2

Match to Query 415: 1851.754248 from(926.884400,2+) intensity(4681.0000) index(155)
Data file IS_111911_23b.txt

Click mouse within plot area to zoom in by factor of two about that point
Or, Plot from 100 to 1400 Da Full range
Label all possible matches ○ Label matches used for scoring ◉
Show Y-axis ☐

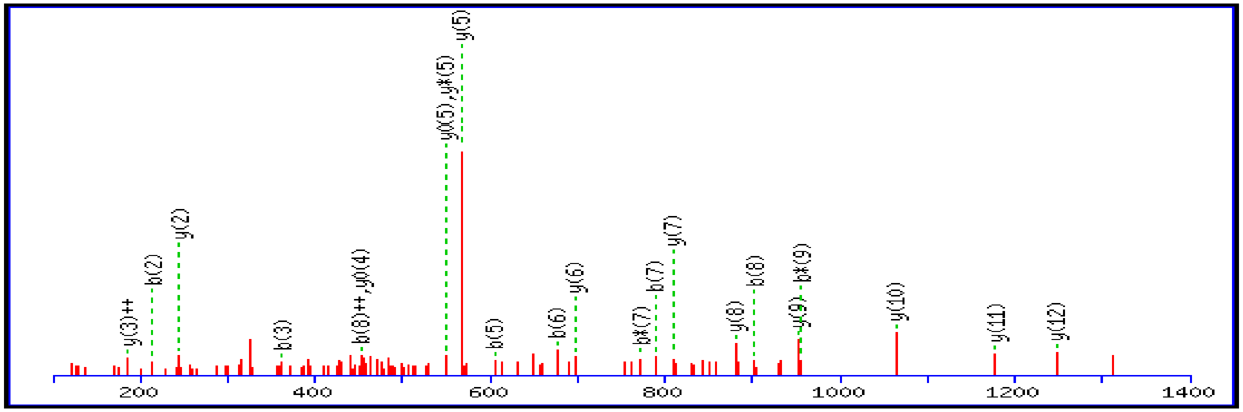

Monoisotopic mass of neutral peptide Mr(calc): 1851.9618
Fixed modifications: Carbamidomethyl (C) (apply to specified residues or termini only)
Ions Score: 57 Expect: 0.0003
Matches : 22/182 fragment ions using 31 most intense peaks (help)

#	b	b^{++}	b^{*}	b^{*++}	b^{0}	b^{0++}	Seq.	y	y^{++}	y^{*}	y^{*++}	y^{0}	y^{0++}	#
1	115.0502	58.0287	98.0237	49.5155			N							17
2	*214.1186*	107.5629	197.0921	99.0497			V	1738.9262	869.9667	1721.8996	861.4535	1720.9156	860.9614	16
3	*361.1870*	181.0972	344.1605	172.5839			F	1639.8578	820.4325	1622.8312	811.9192	1621.8472	811.4272	15
4	476.2140	238.6106	459.1874	230.0974	458.2034	229.6053	D	1492.7894	746.8983	1475.7628	738.3850	1474.7788	737.8930	14
5	*605.2566*	303.1319	588.2300	294.6186	587.2460	294.1266	E	1377.7624	689.3848	1360.7359	680.8716	1359.7518	680.3796	13
6	*676.2937*	338.6505	659.2671	330.1372	658.2831	329.6452	A	*1248.7198*	624.8635	1231.6933	616.3503	1230.7093	615.8583	12
7	*789.3777*	395.1925	772.3512	386.6792	771.3672	386.1872	I	*1177.6827*	589.3450	1160.6562	580.8317	1159.6721	580.3397	11
8	*902.4618*	451.7345	885.4353	443.2213	884.4512	442.7293	L	*1064.5986*	532.8030	1047.5721	524.2897	1046.5881	523.7977	10
9	973.4989	487.2531	956.4724	478.7398	955.4884	478.2478	A	*951.5146*	476.2609	934.4880	467.7477	933.5040	467.2556	9
10	1044.5360	522.7717	1027.5095	514.2584	1026.5255	513.7664	A	*880.4775*	440.7424	863.4509	432.2291	862.4669	431.7371	8
11	1157.6201	579.3137	1140.5936	570.8004	1139.6095	570.3084	L	*809.4403*	405.2238	792.4138	396.7105	791.4298	396.2185	7
12	1286.6627	643.8350	1269.6361	635.3217	1268.6521	634.8297	E	*696.3563*	348.6818	679.3297	340.1685	678.3457	339.6765	6
13	1383.7155	692.3614	1366.6889	683.8481	1365.7049	683.3561	P	*567.3137*	284.1605	550.2871	275.6472	549.3031	275.1552	5
14	1480.7682	740.8877	1463.7417	732.3745	1462.7577	731.8825	P	470.2609	235.6341	453.2344	227.1208	452.2504	226.6288	4
15	1609.8108	805.4090	1592.7843	796.8958	1591.8003	796.4038	E	373.2082	187.1077	356.1816	178.5944	355.1976	178.1024	3
16	1706.8636	853.9354	1689.8370	845.4222	1688.8530	844.9301	P	*244.1656*	122.5864	227.1390	114.0731			2
17							K	147.1128	74.0600	130.0863	65.5468			1

MS/MS Fragmentation of **FALGLSGGSLVSMLAR**
Found in **6PGL_HUMAN** in **SwissProt**, 6-phosphogluconolactonase OS=Homo sapiens GN=PGLS PE=1 SV=2

Match to Query 356: 1593.907648 from(797.961100,2+) intensity(6408.8000) index(424)
Data file IS_111911_23b.txt

Click mouse within plot area to zoom in by factor of two about that point
Or, Plot from 100 to 1600 Da Full range
Label all possible matches ○ Label matches used for scoring ◉
Show Y-axis ☐

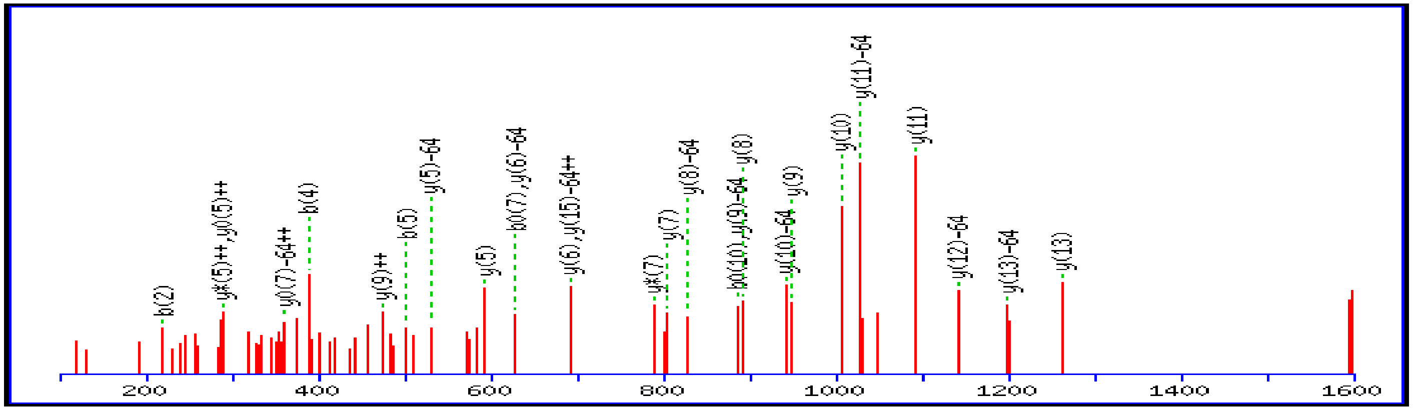


```
Monoisotopic mass of neutral peptide Mr(calc): 1593.8549
Fixed modifications: Carbamidomethyl (C) (apply to specified residues or termini only)
Variable modifications:
M13     : Oxidation (M), with neutral losses 0.0000(shown in table), 63.9983
Ions Score: 53  Expect: 0.0008
Matches : 27/214 fragment ions using 33 most intense peaks   (help)
```

#	b	b++	b0	b0++	Seq.	y	y++	y*	y*++	y0	y0++	#
1	148.0757	74.5415			F							16
2	**219.1128**	110.0600			A	1447.7937	724.4005	1430.7672	715.8872	1429.7832	715.3952	15
3	332.1969	166.6021			L	1376.7566	688.8819	1359.7301	680.3687	1358.7460	679.8767	14
4	**389.2183**	195.1128			G	***1263.6725***	632.3399	1246.6460	623.8266	1245.6620	623.3346	13
5	**502.3024**	251.6548			L	1206.6511	603.8292	1189.6245	595.3159	1188.6405	594.8239	12
6	589.3344	295.1709	571.3239	286.1656	S	***1093.5670***	547.2871	1076.5405	538.7739	1075.5565	538.2819	11
7	646.3559	323.6816	628.3453	314.6763	G	***1006.5350***	503.7711	989.5084	495.2579	988.5244	494.7659	10
8	703.3774	352.1923	685.3668	343.1870	G	***949.5135***	475.2604	932.4870	466.7471	931.5030	466.2551	9
9	790.4094	395.7083	772.3988	386.7030	S	***892.4921***	446.7497	875.4655	438.2364	874.4815	437.7444	8
10	903.4934	452.2504	885.4829	443.2451	L	***805.4600***	403.2337	788.4335	394.7204	787.4495	394.2284	7
11	1002.5619	501.7846	984.5513	492.7793	V	***692.3760***	346.6916	675.3494	338.1783	674.3654	337.6863	6
12	1089.5939	545.3006	1071.5833	536.2953	S	***593.3076***	297.1574	576.2810	288.6441	575.2970	288.1521	5
13	1236.6293	618.8183	1218.6187	609.8130	M	506.2755	253.6414	489.2490	245.1281			4
14	1349.7133	675.3603	1331.7028	666.3550	L	359.2401	180.1237	342.2136	171.6104			3
15	1420.7505	710.8789	1402.7399	701.8736	A	246.1561	123.5817	229.1295	115.0684			2
16					R	175.1190	88.0631	158.0924	79.5498			1

MS/MS Fragmentation of **TLWTVLDAIDQMWLPVVR**
Found in **PGAM1_HUMAN** in **SwissProt**, Phosphoglycerate mutase 1 OS=Homo sapiens GN=PGAM1 PE=1 SV=2

Match to Query 496: 2170.559248 from(1086.286900,2+) intensity(4375.9000) index(521)
Data file IS_111911_23b.txt

Click mouse within plot area to zoom in by factor of two about that point
Or, Plot from 100 to 1600 Da Full range
Label all possible matches ○ Label matches used for scoring ◉
Show Y-axis ☐

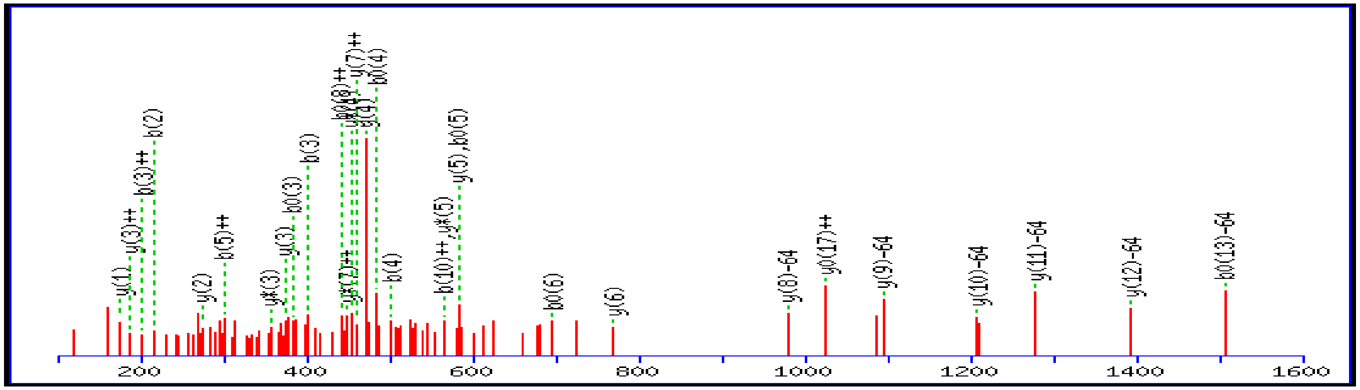

Monoisotopic mass of neutral peptide Mr(calc): 2171.1449
Fixed modifications: Carbamidomethyl (C) (apply to specified residues or termini only)
Variable modifications:
M12 : Oxidation (M), with neutral losses 63.9983(shown in table), 0.0000
Ions Score: 52 Expect: 0.0006
Matches : 30/266 fragment ions using 58 most intense peaks (help)

#	b	b^{++}	b^{*}	b^{*++}	b^{0}	b^{0++}	Seq.	y	y^{++}	y^{*}	y^{*++}	y^{0}	y^{0++}	#
1	102.0550	51.5311			84.0444	42.5258	T							18
2	**215.1390**	108.0731			197.1285	99.0679	L	2007.1062	1004.0567	1990.0797	995.5435	1989.0956	995.0515	17
3	**401.2183**	**201.1128**			383.2078	192.1075	W	1894.0221	947.5147	1876.9956	939.0014	1876.0116	938.5094	16
4	**502.2660**	251.6366			484.2554	242.6314	T	1707.9428	854.4751	1690.9163	845.9618	1689.9323	845.4698	15
5	601.3344	**301.1709**			583.3239	292.1656	V	1606.8952	803.9512	1589.8686	795.4379	1588.8846	794.9459	14
6	714.4185	357.7129			696.4079	348.7076	L	1507.8267	754.4170	1490.8002	745.9037	1489.8162	745.4117	13
7	829.4454	415.2264			811.4349	406.2211	D	*1394.7427*	697.8750	1377.7161	689.3617	1376.7321	688.8697	12
8	900.4825	450.7449			882.4720	441.7396	A	*1279.7157*	640.3615	1262.6892	631.8482	1261.7052	631.3562	11
9	1013.5666	507.2869			995.5560	498.2817	I	*1208.6786*	604.8429	1191.6521	596.3297	1190.6681	595.8377	10
10	1128.5936	**564.8004**			1110.5830	555.7951	D	*1095.5946*	548.3009	1078.5680	539.7876	1077.5840	539.2956	9
11	1256.6521	628.8297	1239.6256	620.3164	1238.6416	619.8244	Q	*980.5676*	490.7874	963.5411	482.2742			8
12	1339.6892	670.3483	1322.6627	661.8350	1321.6787	661.3430	M	852.5090	426.7582	835.4825	418.2449			7
13	1525.7686	763.3879	1508.7420	754.8746	1507.7580	754.3826	W	*769.4719*	385.2396	752.4454	376.7263			6
14	1638.8526	819.9299	1621.8261	811.4167	1620.8421	810.9247	L	*583.3926*	292.1999	566.3661	283.6867			5
15	1735.9054	868.4563	1718.8788	859.9431	1717.8948	859.4510	P	*470.3085*	235.6579	453.2820	227.1446			4
16	1834.9738	917.9905	1817.9473	909.4773	1816.9632	908.9853	V	*373.2558*	187.1315	356.2292	178.6183			3
17	1934.0422	967.5247	1917.0157	959.0115	1916.0317	958.5195	V	*274.1874*	137.5973	257.1608	129.0840			2
18							R	*175.1190*	88.0631	158.0924	79.5498			1

MS/MS Fragmentation of **ALDLFSDNAPPPELLEIINEDIAKR**
Found in **PDIA6_HUMAN** in **SwissProt**, Protein disulfide-isomerase A6 OS=Homo sapiens GN=PDIA6 PE=1 SV=1

Match to Query 551: 2792.113872 from(931.711900,3+) intensity(3810.9000) index(171)
Data file IS_111911_23b.txt

Click mouse within plot area to zoom in by factor of two about that point
Or, Plot from 200 to 2800 Da Full range
Label all possible matches ○ Label matches used for scoring ◉
Show Y-axis ☐

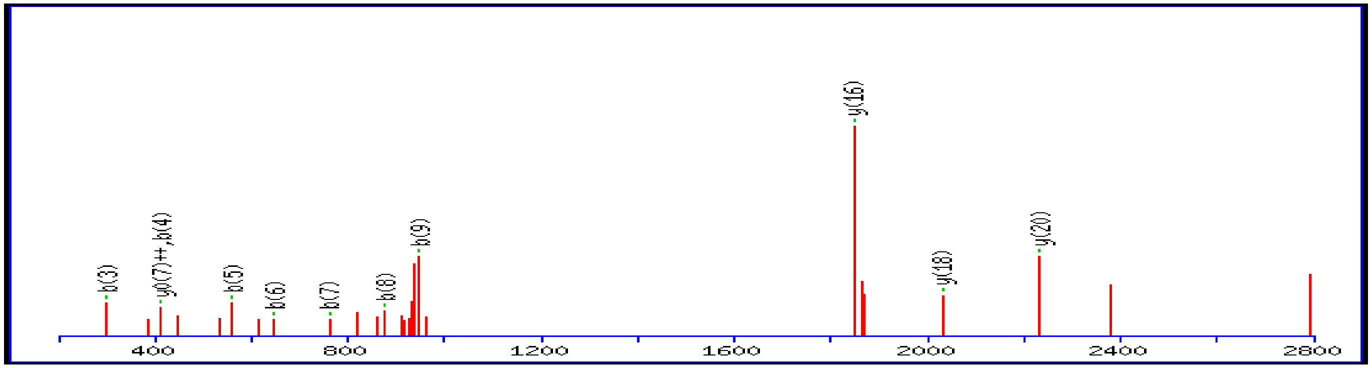

Monoisotopic mass of neutral peptide Mr(calc): 2792.4596
Fixed modifications: Carbamidomethyl (C) (apply to specified residues or termini only)
Ions Score: 50 Expect: 0.0008
Matches : 11/262 fragment ions using 12 most intense peaks (help)

#	b	b^{++}	b*	b^{*++}	b^0	b^{0++}	Seq.	y	y^{++}	y*	y^{*++}	y^0	y^{0++}	#
1	72.0444	36.5258					A							25
2	185.1285	93.0679					L	2722.4298	1361.7185	2705.4032	1353.2053	2704.4192	1352.7132	24
3	*300.1554*	150.5813			282.1448	141.5761	D	2609.3457	1305.1765	2592.3192	1296.6632	2591.3352	1296.1712	23
4	*413.2395*	207.1234			395.2289	198.1181	L	2494.3188	1247.6630	2477.2922	1239.1498	2476.3082	1238.6577	22
5	*560.3079*	280.6576			542.2973	271.6523	F	2381.2347	1191.1210	2364.2082	1182.6077	2363.2242	1182.1157	21
6	*647.3399*	324.1736			629.3293	315.1683	S	**2234.1663**	1117.5868	2217.1398	1109.0735	2216.1557	1108.5815	20
7	*762.3668*	381.6871			744.3563	372.6818	D	2147.1343	1074.0708	2130.1077	1065.5575	2129.1237	1065.0655	19
8	*876.4098*	438.7085	859.3832	430.1952	858.3992	429.7032	N	**2032.1073**	1016.5573	2015.0808	1008.0440	2014.0968	1007.5520	18
9	*947.4469*	474.2271	930.4203	465.7138	929.4363	465.2218	A	1918.0644	959.5358	1901.0379	951.0226	1900.0538	950.5306	17
10	1044.4997	522.7535	1027.4731	514.2402	1026.4891	513.7482	P	**1847.0273**	924.0173	1830.0007	915.5040	1829.0167	915.0120	16
11	1141.5524	571.2798	1124.5259	562.7666	1123.5418	562.2746	P	1749.9745	875.4909	1732.9480	866.9776	1731.9640	866.4856	15
12	1238.6052	619.8062	1221.5786	611.2930	1220.5946	610.8009	P	1652.9218	826.9645	1635.8952	818.4512	1634.9112	817.9592	14
13	1367.6478	684.3275	1350.6212	675.8142	1349.6372	675.3222	E	1555.8690	778.4381	1538.8424	769.9249	1537.8584	769.4329	13
14	1480.7318	740.8696	1463.7053	732.3563	1462.7213	731.8643	L	1426.8264	713.9168	1409.7999	705.4036	1408.8158	704.9116	12
15	1593.8159	797.4116	1576.7894	788.8983	1575.8053	788.4063	L	1313.7423	657.3748	1296.7158	648.8615	1295.7318	648.3695	11
16	1722.8585	861.9329	1705.8319	853.4196	1704.8479	852.9276	E	1200.6583	600.8328	1183.6317	592.3195	1182.6477	591.8275	10
17	1835.9426	918.4749	1818.9160	909.9616	1817.9320	909.4696	I	1071.6157	536.3115	1054.5891	527.7982	1053.6051	527.3062	9
18	1949.0266	975.0169	1932.0001	966.5037	1931.0161	966.0117	I	958.5316	479.7694	941.5051	471.2562	940.5211	470.7642	8
19	2063.0695	1032.0384	2046.0430	1023.5251	2045.0590	1023.0331	N	845.4476	423.2274	828.4210	414.7141	827.4370	414.2221	7
20	2192.1121	1096.5597	2175.0856	1088.0464	2174.1016	1087.5544	E	731.4046	366.2060	714.3781	357.6927	713.3941	357.2007	6
21	2307.1391	1154.0732	2290.1125	1145.5599	2289.1285	1145.0679	D	602.3620	301.6847	585.3355	293.1714	584.3515	292.6794	5
22	2420.2231	1210.6152	2403.1966	1202.1019	2402.2126	1201.6099	I	487.3351	244.1712	470.3085	235.6579			4
23	2491.2603	1246.1338	2474.2337	1237.6205	2473.2497	1237.1285	A	374.2510	187.6292	357.2245	179.1159			3
24	2619.3552	1310.1813	2602.3287	1301.6680	2601.3447	1301.1760	K	303.2139	152.1106	286.1874	143.5973			2
25							R	175.1190	88.0631	158.0924	79.5498			1

MS/MS Fragmentation of **SVYAHFPINVVIQENGSLVEIR**
Found in **RL9_HUMAN** in **SwissProt**, 60S ribosomal protein L9 OS=Homo sapiens GN=RPL9 PE=1 SV=1

Match to Query 528: 2483.323272 from(828.781700,3+) intensity(2117.8000) index(427)
Data file IS_111911_23b.txt

Click mouse within plot area to zoom in by factor of two about that point
Or, Plot from 0 to 2600 Da Full range
Label all possible matches ○ Label matches used for scoring ◉
Show Y-axis ☐

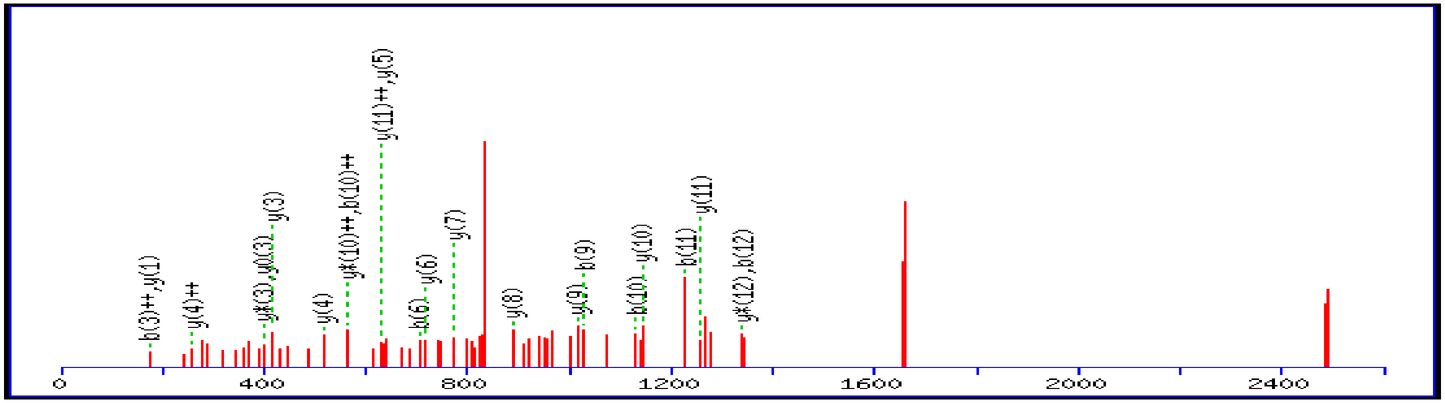


```
Monoisotopic mass of neutral peptide Mr(calc): 2483.3172
Fixed modifications: Carbamidomethyl (C) (apply to specified residues or termini only)
Ions Score: 44 Expect: 0.0035
Matches : 23/232 fragment ions using 39 most intense peaks (help)
```

#	b	b^{++}	b^{*}	b^{*++}	b^{0}	b^{0++}	Seq.	y	y^{++}	y^{*}	y^{*++}	y^{0}	y^{0++}	#
1	88.0393	44.5233			70.0287	35.5180	S							22
2	187.1077	94.0575			169.0972	85.0522	V	2397.2925	1199.1499	2380.2660	1190.6366	2379.2819	1190.1446	21
3	350.1710	175.5892			332.1605	166.5839	Y	2298.2241	1149.6157	2281.1975	1141.1024	2280.2135	1140.6104	20
4	421.2082	211.1077			403.1976	202.1024	A	2135.1608	1068.0840	2118.1342	1059.5707	2117.1502	1059.0787	19
5	558.2671	279.6372			540.2565	270.6319	H	2064.1236	1032.5655	2047.0971	1024.0522	2046.1131	1023.5602	18
6	705.3355	353.1714			687.3249	344.1661	F	1927.0647	964.0360	1910.0382	955.5227	1909.0542	955.0307	17
7	802.3883	401.6978			784.3777	392.6925	P	1779.9963	890.5018	1762.9698	881.9885	1761.9858	881.4965	16
8	915.4723	458.2398			897.4617	449.2345	I	1682.9436	841.9754	1665.9170	833.4621	1664.9330	832.9701	15
9	1029.5152	515.2613	1012.4887	506.7480	1011.5047	506.2560	N	1569.8595	785.4334	1552.8329	776.9201	1551.8489	776.4281	14
10	1128.5837	564.7955	1111.5571	556.2822	1110.5731	555.7902	V	1455.8166	728.4119	1438.7900	719.8986	1437.8060	719.4066	13
11	1227.6521	614.3297	1210.6255	605.8164	1209.6415	605.3244	V	1356.7482	678.8777	1339.7216	670.3644	1338.7376	669.8724	12
12	1340.7361	670.8717	1323.7096	662.3584	1322.7256	661.8664	I	*1257.6797*	629.3435	1240.6532	620.8302	1239.6692	620.3382	11
13	1468.7947	734.9010	1451.7682	726.3877	1450.7841	725.8957	Q	*1144.5957*	572.8015	1127.5691	564.2882	1126.5851	563.7962	10
14	1597.8373	799.4223	1580.8108	790.9090	1579.8267	790.4170	E	*1016.5371*	508.7722	999.5106	500.2589	998.5265	499.7669	9
15	1711.8802	856.4438	1694.8537	847.9305	1693.8697	847.4385	N	*887.4945*	444.2509	870.4680	435.7376	869.4839	435.2456	8
16	1768.9017	884.9545	1751.8751	876.4412	1750.8911	875.9492	G	*773.4516*	387.2294	756.4250	378.7162	755.4410	378.2241	7
17	1855.9337	928.4705	1838.9072	919.9572	1837.9232	919.4652	S	*716.4301*	358.7187	699.4036	350.2054	698.4196	349.7134	6
18	1969.0178	985.0125	1951.9912	976.4993	1951.0072	976.0072	L	*629.3981*	315.2027	612.3715	306.6894	611.3875	306.1974	5
19	2068.0862	1034.5467	2051.0597	1026.0335	2050.0756	1025.5415	V	*516.3140*	258.6606	499.2875	250.1474	498.3035	249.6554	4
20	2197.1288	1099.0680	2180.1022	1090.5548	2179.1182	1090.0628	E	*417.2456*	209.1264	400.2191	200.6132	399.2350	200.1212	3
21	2310.2129	1155.6101	2293.1863	1147.0968	2292.2023	1146.6048	I	288.2030	144.6051	271.1765	136.0919			2
22							R	*175.1190*	88.0631	158.0924	79.5498			1

MS/MS Fragmentation of **LFQVEYAIEAIK**
Found in **PSA5_HUMAN** in **SwissProt**, Proteasome subunit alpha type-5 OS=Homo sapiens GN=PSMA5 PE=1 SV=3

Match to Query 307: 1423.027048 from(712.520800,2+) intensity(4840.0000) index(110)
Data file IS_111911_23b.txt

Click mouse within plot area to zoom in by factor of two about that point
Or, [Plot from] 100 to 1500 Da [Full range]
Label all possible matches ○ Label matches used for scoring ◉
Show Y-axis ☐

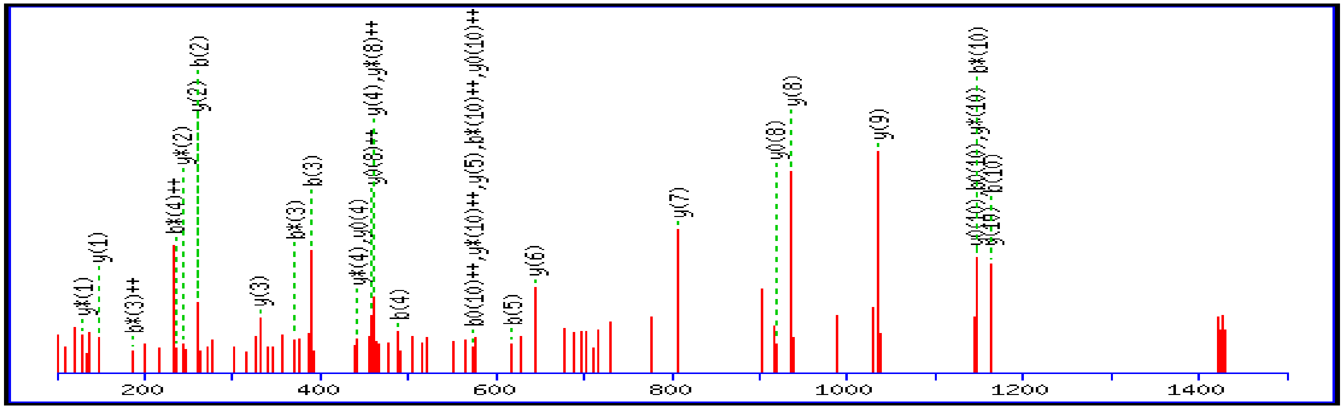

Monoisotopic mass of neutral peptide Mr(calc): 1422.7758
Fixed modifications: Carbamidomethyl (C) (apply to specified residues or termini only)
Ions Score: 43 Expect: 0.0082
Matches : 33/114 fragment ions using 79 most intense peaks (help)

#	b	b^{++}	b^{*}	b^{*++}	b^{0}	b^{0++}	Seq.	y	y^{++}	y^{*}	y^{*++}	y^{0}	y^{0++}	#
1	114.0913	57.5493					L							12
2	**261.1598**	131.0835					F	1310.6991	655.8532	1293.6725	647.3399	1292.6885	646.8479	11
3	**389.2183**	195.1128	372.1918	186.5995			Q	***1163.6307***	582.3190	1146.6041	573.8057	1145.6201	573.3137	10
4	**488.2867**	244.6470	471.2602	236.1337			V	***1035.5721***	518.2897	1018.5455	509.7764	1017.5615	509.2844	9
5	**617.3293**	309.1683	600.3028	300.6550	599.3188	300.1630	E	***936.5037***	468.7555	919.4771	460.2422	918.4931	459.7502	8
6	780.3927	390.7000	763.3661	382.1867	762.3821	381.6947	Y	***807.4611***	404.2342	790.4345	395.7209	789.4505	395.2289	7
7	851.4298	426.2185	834.4032	417.7053	833.4192	417.2132	A	***644.3978***	322.7025	627.3712	314.1892	626.3872	313.6972	6
8	964.5138	482.7606	947.4873	474.2473	946.5033	473.7553	I	***573.3606***	287.1840	556.3341	278.6707	555.3501	278.1787	5
9	1093.5564	547.2819	1076.5299	538.7686	1075.5459	538.2766	E	***460.2766***	230.6419	443.2500	222.1287	442.2660	221.6366	4
10	**1164.5936**	582.8004	1147.5670	574.2871	1146.5830	573.7951	A	***331.2340***	166.1206	314.2074	157.6074			3
11	1277.6776	639.3424	1260.6511	630.8292	1259.6671	630.3372	I	***260.1969***	130.6021	243.1703	122.0888			2
12							K	***147.1128***	74.0600	130.0863	65.5468			1

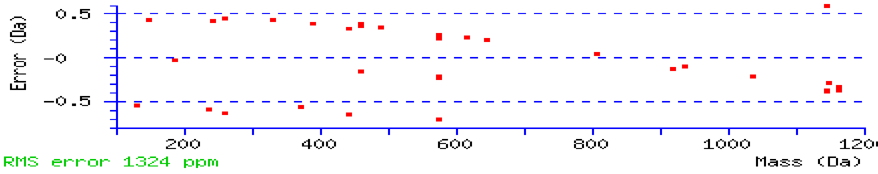

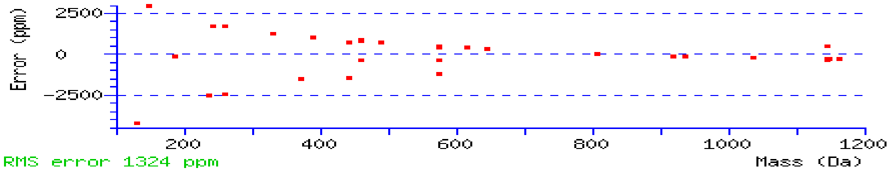

MS/MS Fragmentation of **LLCGLLAER**
Found in **MIF_HUMAN** in **SwissProt**, Macrophage migration inhibitory factor OS=Homo sapiens GN=MIF PE=1 SV=4

Match to Query 147: 1044.186448 from(523.100500,2+) intensity(4021.9000) index(66)
Data file IS_111911_23b.txt

Click mouse within plot area to zoom in by factor of two about that point
Or, Plot from 100 to 850 Da Full range
Label all possible matches ○ Label matches used for scoring ◉
Show Y-axis ☐

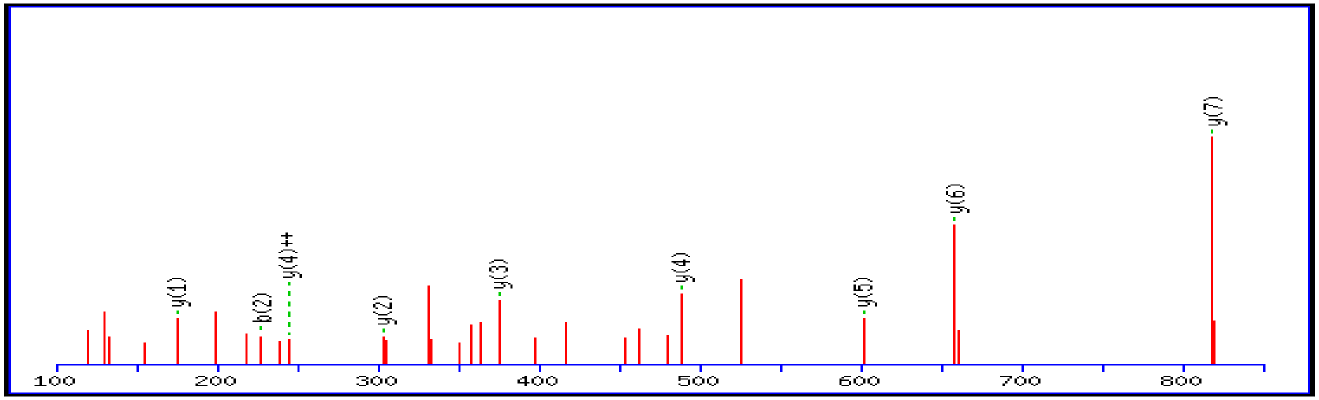


```
Monoisotopic mass of neutral peptide Mr(calc): 1043.5797
Fixed modifications: Carbamidomethyl (C) (apply to specified residues or termini only)
Ions Score: 41  Expect: 0.013
Matches : 9/64 fragment ions using 18 most intense peaks  (help)
```

#	b	b^{++}	b^0	b^{0++}	Seq.	y	y^{++}	y^*	y^{*++}	y^0	y^{0++}	#
1	114.0913	57.5493			L							9
2	227.1754	114.0913			L	931.5030	466.2551	914.4764	457.7418	913.4924	457.2498	8
3	387.2061	194.1067			C	*818.4189*	409.7131	801.3924	401.1998	800.4083	400.7078	7
4	444.2275	222.6174			G	*658.3883*	329.6978	641.3617	321.1845	640.3777	320.6925	6
5	557.3116	279.1594			L	*601.3668*	301.1870	584.3402	292.6738	583.3562	292.1817	5
6	670.3956	335.7015			L	*488.2827*	244.6450	471.2562	236.1317	470.2722	235.6397	4
7	741.4328	371.2200			A	*375.1987*	188.1030	358.1721	179.5897	357.1881	179.0977	3
8	870.4754	435.7413	852.4648	426.7360	E	*304.1615*	152.5844	287.1350	144.0711	286.1510	143.5791	2
9					R	*175.1190*	88.0631	158.0924	79.5498			1

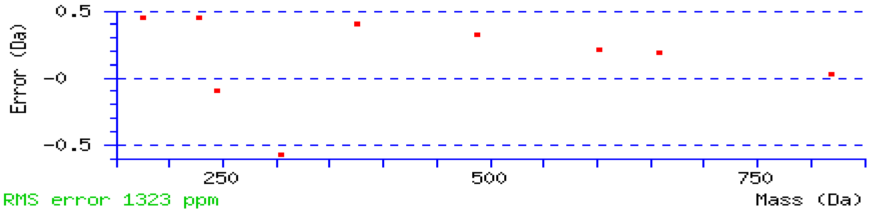

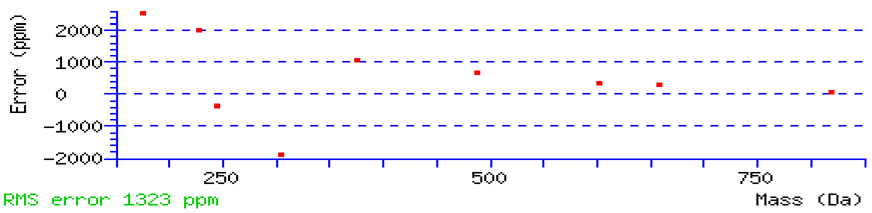

MS/MS Fragmentation of VKPFMTGAAEQIK
Found in TCTP_HUMAN in SwissProt, Translationally-controlled tumor protein OS=Homo sapiens GN=TPT1 PE=1 SV=1

Match to Query 309: 1434.998048 from(718.506300,2+) intensity(3126.8000) index(24)
Data file IS_111911_23b.txt

Click mouse within plot area to zoom in by factor of two about that point
Or, Plot from 100 to 1500 Da Full range
Label all possible matches ○ Label matches used for scoring ◉
Show Y-axis ☐

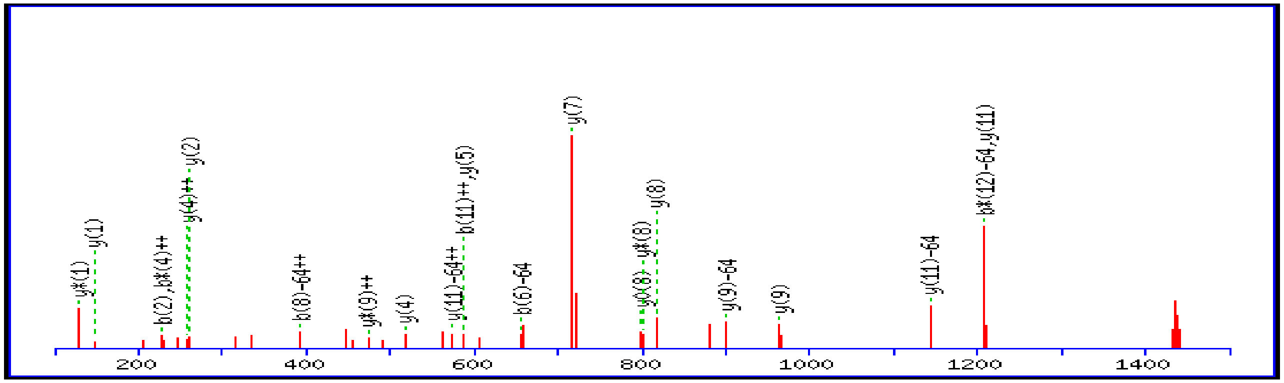

Monoisotopic mass of neutral peptide Mr(calc): 1434.7541
Fixed modifications: Carbamidomethyl (C) (apply to specified residues or termini only)
Variable modifications:
M5 : Oxidation (M), with neutral losses 0.0000(shown in table), 63.9983
Ions Score: 41 Expect: 0.014
Matches : 22/196 fragment ions using 38 most intense peaks (help)

#	b	b^{++}	b^{*}	b^{*++}	b^{0}	b^{0++}	Seq.	y	y^{++}	y^{*}	y^{*++}	y^{0}	y^{0++}	#
1	100.0757	50.5415					V							13
2	228.1707	114.5890	211.1441	106.0757			K	1336.6930	668.8501	1319.6664	660.3368	1318.6824	659.8448	12
3	325.2234	163.1153	308.1969	154.6021			P	1208.5980	604.8026	1191.5714	596.2894	1190.5874	595.7973	11
4	472.2918	236.6496	455.2653	228.1363			F	1111.5452	556.2762	1094.5187	547.7630	1093.5347	547.2710	10
5	619.3272	310.1673	602.3007	301.6540			M	964.4768	482.7420	947.4503	474.2288	946.4662	473.7368	9
6	720.3749	360.6911	703.3484	352.1778	702.3643	351.6858	T	817.4414	409.2243	800.4149	400.7111	799.4308	400.2191	8
7	777.3964	389.2018	760.3698	380.6886	759.3858	380.1965	G	716.3937	358.7005	699.3672	350.1872	698.3832	349.6952	7
8	848.4335	424.7204	831.4069	416.2071	830.4229	415.7151	A	659.3723	330.1898	642.3457	321.6765	641.3617	321.1845	6
9	919.4706	460.2389	902.4441	451.7257	901.4600	451.2337	A	588.3352	294.6712	571.3086	286.1579	570.3246	285.6659	5
10	1048.5132	524.7602	1031.4866	516.2470	1030.5026	515.7550	E	517.2980	259.1527	500.2715	250.6394	499.2875	250.1474	4
11	1176.5718	588.7895	1159.5452	580.2763	1158.5612	579.7842	Q	388.2554	194.6314	371.2289	186.1181			3
12	1289.6558	645.3316	1272.6293	636.8183	1271.6453	636.3263	I	260.1969	130.6021	243.1703	122.0888			2
13							K	147.1128	74.0600	130.0863	65.5468			1

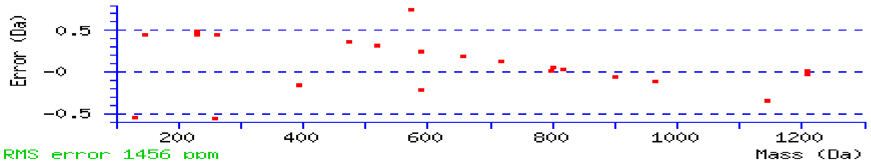

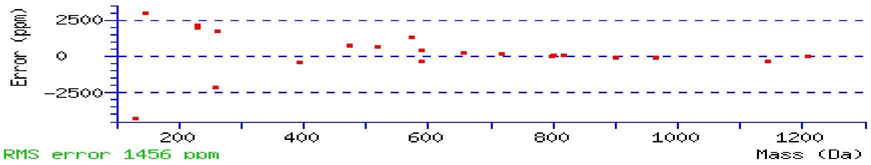

MS/MS Fragmentation of NGPVEGAFSVYSDFLLYK
Found in CATB_HUMAN in SwissProt, Cathepsin B OS=Homo sapiens GN=CTSB PE=1 SV=3

Match to Query 455: 2004.602648 from(1003.308600,2+) intensity(7756.4000) index(464)
Data file IS_111911_23b.txt

Click mouse within plot area to zoom in by factor of two about that point
Or, Plot from 100 to 1800 Da Full range
Label all possible matches ○ Label matches used for scoring ◉
Show Y-axis ☐

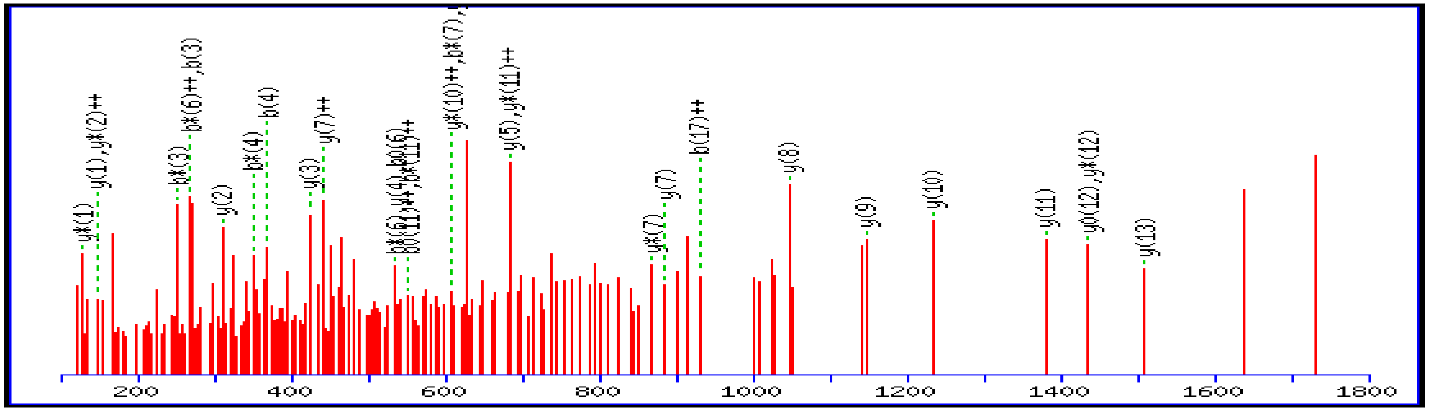

Monoisotopic mass of neutral peptide Mr(calc): 2004.9833
Fixed modifications: Carbamidomethyl (C) (apply to specified residues or termini only)
Ions Score: 38 Expect: 0.017
Matches : 31/186 fragment ions using 59 most intense peaks (help)

#	b	b++	b*	b*++	b0	b0++	Seq.	y	y++	y*	y*++	y0	y0++	#
1	115.0502	58.0287	98.0237	49.5155			N							18
2	172.0717	86.5395	155.0451	78.0262			G	1891.9476	946.4775	1874.9211	937.9642	1873.9371	937.4722	17
3	269.1244	135.0659	252.0979	126.5526			P	1834.9262	917.9667	1817.8996	909.4535	1816.9156	908.9614	16
4	368.1928	184.6001	351.1663	176.0868			V	1737.8734	869.4403	1720.8469	860.9271	1719.8629	860.4351	15
5	497.2354	249.1214	480.2089	240.6081	479.2249	240.1161	E	1638.8050	819.9061	1621.7785	811.3929	1620.7944	810.9009	14
6	554.2569	277.6321	537.2304	269.1188	536.2463	268.6268	G	1509.7624	755.3848	1492.7359	746.8716	1491.7518	746.3796	13
7	625.2940	313.1506	608.2675	304.6374	607.2835	304.1454	A	1452.7409	726.8741	1435.7144	718.3608	1434.7304	717.8688	12
8	772.3624	386.6849	755.3359	378.1716	754.3519	377.6796	F	1381.7038	691.3556	1364.6773	682.8423	1363.6933	682.3503	11
9	859.3945	430.2009	842.3679	421.6876	841.3839	421.1956	S	1234.6354	617.8213	1217.6089	609.3081	1216.6249	608.8161	10
10	958.4629	479.7351	941.4363	471.2218	940.4523	470.7298	V	1147.6034	574.3053	1130.5768	565.7921	1129.5928	565.3001	9
11	1121.5262	561.2667	1104.4997	552.7535	1103.5156	552.2615	Y	1048.5350	524.7711	1031.5084	516.2579	1030.5244	515.7658	8
12	1208.5582	604.7828	1191.5317	596.2695	1190.5477	595.7775	S	885.4716	443.2395	868.4451	434.7262	867.4611	434.2342	7
13	1323.5852	662.2962	1306.5586	653.7829	1305.5746	653.2909	D	798.4396	399.7234	781.4131	391.2102	780.4291	390.7182	6
14	1470.6536	735.8304	1453.6270	727.3172	1452.6430	726.8251	F	683.4127	342.2100	666.3861	333.6967			5
15	1583.7377	792.3725	1566.7111	783.8592	1565.7271	783.3672	L	536.3443	268.6758	519.3177	260.1625			4
16	1696.8217	848.9145	1679.7952	840.4012	1678.8111	839.9092	L	423.2602	212.1337	406.2336	203.6205			3
17	1859.8850	930.4462	1842.8585	921.9329	1841.8745	921.4409	Y	310.1761	155.5917	293.1496	147.0784			2
18							K	147.1128	74.0600	130.0863	65.5468			1

MS/MS Fragmentation of SGSGTMNLGGSLTR
Found in CAPZB_HUMAN in SwissProt, F-actin-capping protein subunit beta OS=Homo sapiens GN=CAPZB PE=1 SV=4

Match to Query 273: 1352.955448 from(677.485000,2+) intensity(3438.9000) index(22)
Data file IS_111911_23b.txt

Click mouse within plot area to zoom in by factor of two about that point
Or, Plot from 500 to 1400 Da Full range
Label all possible matches ○ Label matches used for scoring ◉
Show Y-axis ☐

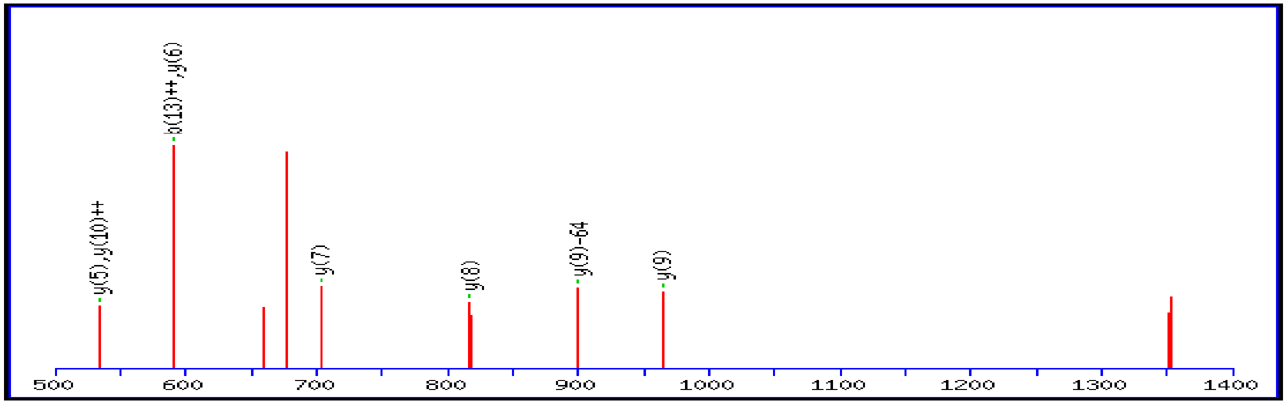


```
Monoisotopic mass of neutral peptide Mr(calc): 1352.6354
Fixed modifications: Carbamidomethyl (C) (apply to specified residues or termini only)
Variable modifications:
M6      : Oxidation (M), with neutral losses 0.0000(shown in table), 63.9983
Ions Score: 36  Expect: 0.046
Matches : 8/218 fragment ions using 9 most intense peaks  (help)
```

#	b	b^{++}	b^{*}	b^{*++}	b^{0}	b^{0++}	Seq.	y	y^{++}	y^{*}	y^{*++}	y^{0}	y^{0++}	#
1	88.0393	44.5233			70.0287	35.5180	S							14
2	145.0608	73.0340			127.0502	64.0287	G	1266.6107	633.8090	1249.5841	625.2957	1248.6001	624.8037	13
3	232.0928	116.5500			214.0822	107.5448	S	1209.5892	605.2982	1192.5627	596.7850	1191.5786	596.2930	12
4	289.1143	145.0608			271.1037	136.0555	G	1122.5572	561.7822	1105.5306	553.2690	1104.5466	552.7769	11
5	390.1619	195.5846			372.1514	186.5793	T	1065.5357	533.2715	1048.5092	524.7582	1047.5252	524.2662	10
6	537.1973	269.1023			519.1868	260.0970	M	964.4880	482.7477	947.4615	474.2344	946.4775	473.7424	9
7	651.2403	326.1238	634.2137	317.6105	633.2297	317.1185	N	817.4526	409.2300	800.4261	400.7167	799.4421	400.2247	8
8	764.3243	382.6658	747.2978	374.1525	746.3138	373.6605	L	703.4097	352.2085	686.3832	343.6952	685.3991	343.2032	7
9	821.3458	411.1765	804.3192	402.6633	803.3352	402.1713	G	590.3257	295.6665	573.2991	287.1532	572.3151	286.6612	6
10	878.3673	439.6873	861.3407	431.1740	860.3567	430.6820	G	533.3042	267.1557	516.2776	258.6425	515.2936	258.1504	5
11	965.3993	483.2033	948.3727	474.6900	947.3887	474.1980	S	476.2827	238.6450	459.2562	230.1317	458.2722	229.6397	4
12	1078.4833	539.7453	1061.4568	531.2320	1060.4728	530.7400	L	389.2507	195.1290	372.2241	186.6157	371.2401	186.1237	3
13	1179.5310	590.2692	1162.5045	581.7559	1161.5205	581.2639	T	276.1666	138.5870	259.1401	130.0737	258.1561	129.5817	2
14							R	175.1190	88.0631	158.0924	79.5498			1

MS/MS Fragmentation of **FAAATGATPIAGR**
Found in **RSSA_HUMAN** in **SwissProt**, 40S ribosomal protein SA OS=Homo sapiens GN=RPSA PE=1 SV=4

Match to Query 197: 1203.113448 from(602.564000,2+) intensity(985.1000) index(326)
Data file IS_111911_23b.txt

Click mouse within plot area to zoom in by factor of two about that point
Or, Plot from 100 to 1300 Da Full range
Label all possible matches ○ Label matches used for scoring ◉
Show Y-axis ☐

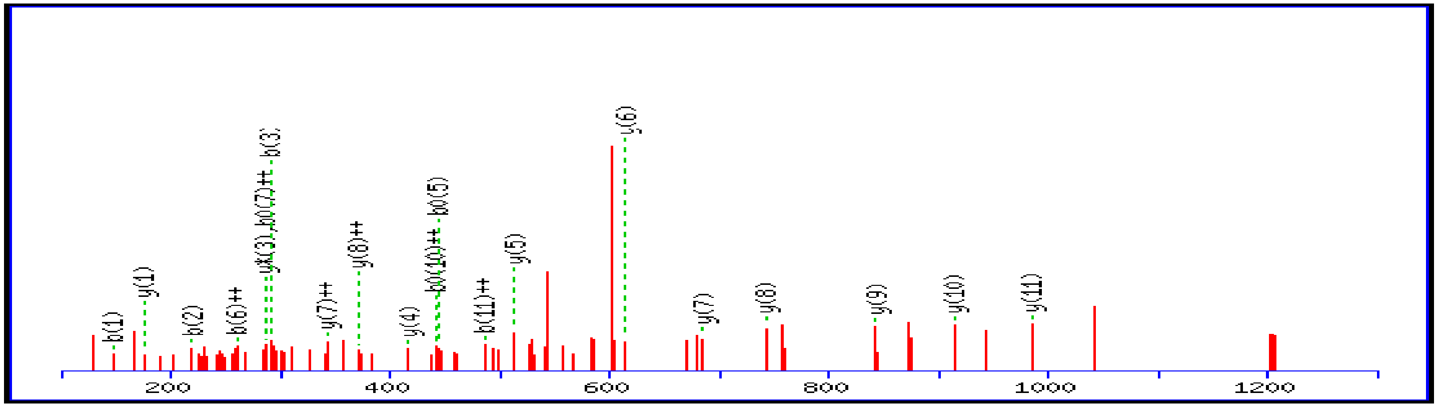

Monoisotopic mass of neutral peptide Mr(calc): 1202.6408
Fixed modifications: Carbamidomethyl (C) (apply to specified residues or termini only)
Ions Score: 35 Expect: 0.045
Matches : 20/102 fragment ions using 54 most intense peaks (help)

#	b	b++	b0	b0++	Seq.	y	y++	y*	y*++	y0	y0++	#
1	**148.0757**	74.5415			F							13
2	**219.1128**	110.0600			A	1056.5796	528.7935	1039.5531	520.2802	1038.5691	519.7882	12
3	**290.1499**	145.5786			A	***985.5425***	493.2749	968.5160	484.7616	967.5320	484.2696	11
4	361.1870	181.0972			A	***914.5054***	457.7563	897.4789	449.2431	896.4948	448.7511	10
5	462.2347	231.6210	444.2241	222.6157	T	***843.4683***	422.2378	826.4417	413.7245	825.4577	413.2325	9
6	519.2562	260.1317	501.2456	251.1264	G	***742.4206***	371.7139	725.3941	363.2007	724.4100	362.7087	8
7	590.2933	295.6503	572.2827	286.6450	A	***685.3992***	343.2032	668.3726	334.6899	667.3886	334.1979	7
8	691.3410	346.1741	673.3304	337.1688	T	***614.3620***	307.6847	597.3355	299.1714	596.3515	298.6794	6
9	788.3937	394.7005	770.3832	385.6952	P	***513.3144***	257.1608	496.2878	248.6475			5
10	901.4778	451.2425	883.4672	442.2373	I	***416.2616***	208.6344	399.2350	200.1212			4
11	972.5149	486.7611	954.5043	477.7558	A	303.1775	152.0924	286.1510	143.5791			3
12	1029.5364	515.2718	1011.5258	506.2665	G	232.1404	116.5738	215.1139	108.0606			2
13					R	***175.1190***	88.0631	158.0924	79.5498			1

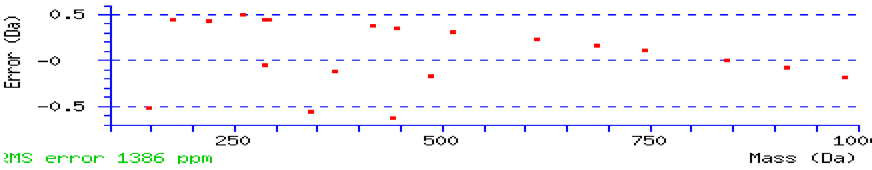

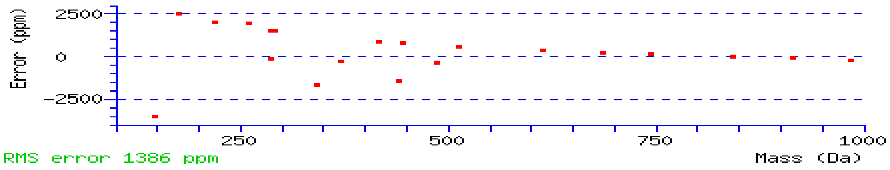

MS/MS Fragmentation of FGAQLAHIQALISGIEAQLGDVR
Found in K1C19_HUMAN in SwissProt, Keratin, type I cytoskeletal 19 OS=Homo sapiens GN=KRT19 PE=1 SV=4

Match to Query 522: 2406.400572 from(803.140800,3+) intensity(1265.7000) index(187)
Data file IS_111911_23b.txt

Click mouse within plot area to zoom in by factor of two about that point
Or, Plot from 100 to 1700 Da Full range
Label all possible matches ○ Label matches used for scoring ◉
Show Y-axis ☐

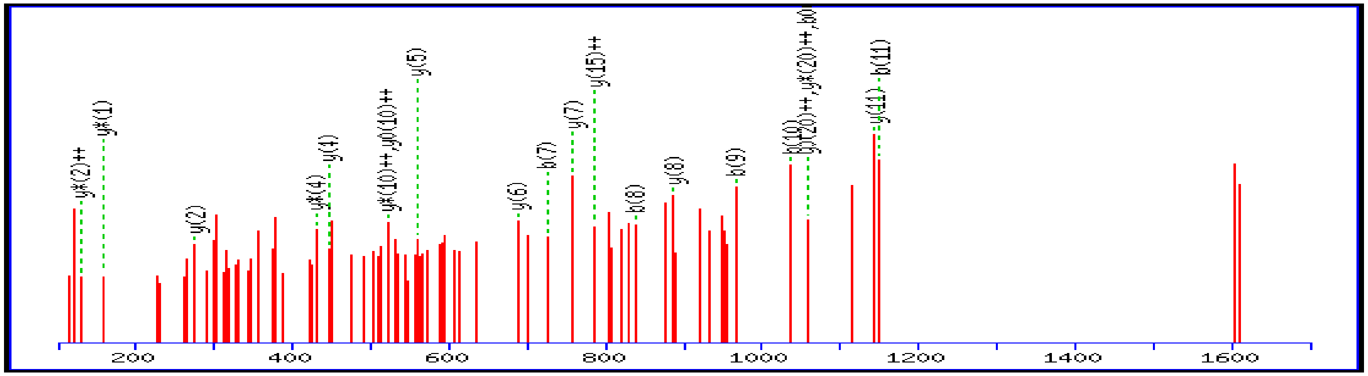

Monoisotopic mass of neutral peptide Mr(calc): 2406.3019
Fixed modifications: Carbamidomethyl (C) (apply to specified residues or termini only)
Ions Score: 34 Expect: 0.036
Matches : 23/230 fragment ions using 43 most intense peaks (help)

#	b	b++	b*	b*++	b0	b0++	Seq.	y	y++	y*	y*++	y0	y0++	#
1	148.0757	74.5415					F							23
2	205.0972	103.0522					G	2260.2408	1130.6240	2243.2143	1122.1108	2242.2302	1121.6188	22
3	276.1343	138.5708					A	2203.2193	1102.1133	2186.1928	1093.6000	2185.2088	1093.1080	21
4	404.1928	202.6001	387.1663	194.0868			Q	2132.1822	1066.5948	2115.1557	1058.0815	2114.1717	1057.5895	20
5	517.2769	259.1421	500.2504	250.6288			L	2004.1237	1002.5655	1987.0971	994.0522	1986.1131	993.5602	19
6	588.3140	294.6607	571.2875	286.1474			A	1891.0396	946.0234	1874.0130	937.5102	1873.0290	937.0181	18
7	725.3729	363.1901	708.3464	354.6768			H	1820.0025	910.5049	1802.9759	901.9916	1801.9919	901.4996	17
8	838.4570	419.7321	821.4305	411.2189			I	1682.9436	841.9754	1665.9170	833.4621	1664.9330	832.9701	16
9	966.5156	483.7614	949.4890	475.2482			Q	1569.8595	785.4334	1552.8329	776.9201	1551.8489	776.4281	15
10	1037.5527	519.2800	1020.5261	510.7667			A	1441.8009	721.4041	1424.7744	712.8908	1423.7904	712.3988	14
11	1150.6368	575.8220	1133.6102	567.3087			L	1370.7638	685.8855	1353.7373	677.3723	1352.7532	676.8803	13
12	1263.7208	632.3640	1246.6943	623.8508			I	1257.6797	629.3435	1240.6532	620.8302	1239.6692	620.3382	12
13	1350.7528	675.8801	1333.7263	667.3668	1332.7423	666.8748	S	1144.5957	572.8015	1127.5691	564.2882	1126.5851	563.7962	11
14	1407.7743	704.3908	1390.7478	695.8775	1389.7637	695.3855	G	1057.5636	529.2855	1040.5371	520.7722	1039.5531	520.2802	10
15	1520.8584	760.9328	1503.8318	752.4196	1502.8478	751.9275	I	1000.5422	500.7747	983.5156	492.2615	982.5316	491.7694	9
16	1649.9010	825.4541	1632.8744	816.9408	1631.8904	816.4488	E	887.4581	444.2327	870.4316	435.7194	869.4476	435.2274	8
17	1720.9381	860.9727	1703.9115	852.4594	1702.9275	851.9674	A	758.4155	379.7114	741.3890	371.1981	740.4050	370.7061	7
18	1848.9967	925.0020	1831.9701	916.4887	1830.9861	915.9967	Q	687.3784	344.1928	670.3519	335.6796	669.3678	335.1876	6
19	1962.0807	981.5440	1945.0542	973.0307	1944.0702	972.5387	L	559.3198	280.1636	542.2933	271.6503	541.3093	271.1583	5
20	2019.1022	1010.0547	2002.0756	1001.5415	2001.0916	1001.0494	G	446.2358	223.6215	429.2092	215.1082	428.2252	214.6162	4
21	2134.1291	1067.5682	2117.1026	1059.0549	2116.1186	1058.5629	D	389.2143	195.1108	372.1878	186.5975	371.2037	186.1055	3
22	2233.1975	1117.1024	2216.1710	1108.5891	2215.1870	1108.0971	V	274.1874	137.5973	257.1608	129.0840			2
23							R	175.1190	88.0631	158.0924	79.5498			1

MS/MS Fragmentation of YALYDATYETK
Found in COF1_HUMAN in SwissProt, Cofilin-1 OS=Homo sapiens GN=CFL1 PE=1 SV=3

Match to Query 262: 1336.970048 from(669.492300,2+) intensity(2116.3000) index(346)
Data file IS_111911_23b.txt

Click mouse within plot area to zoom in by factor of two about that point
Or, Plot from 100 to 1400 Da Full range
Label all possible matches ○ Label matches used for scoring ◉
Show Y-axis ☐

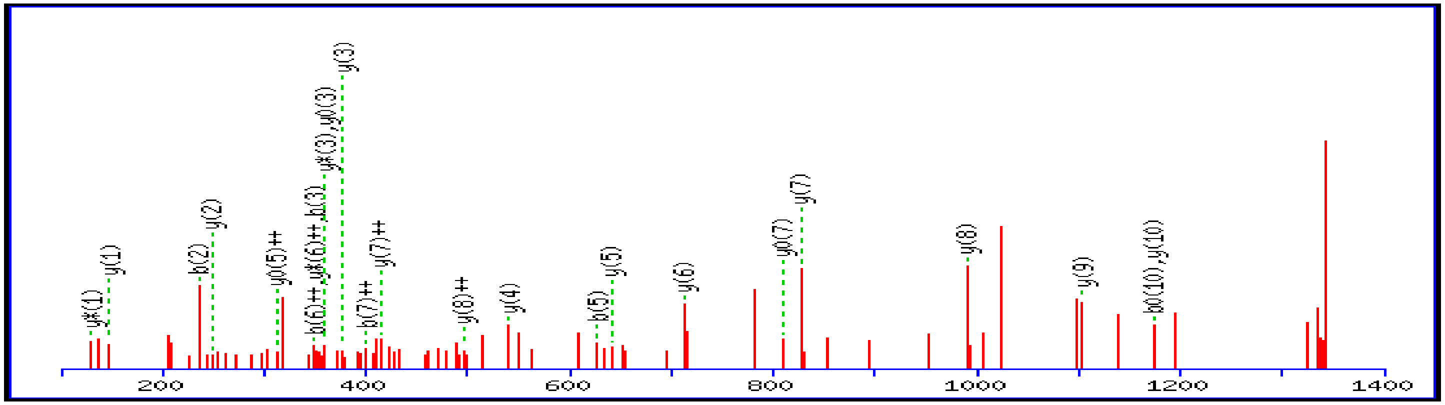

Monoisotopic mass of neutral peptide Mr(calc): 1336.6187
Fixed modifications: Carbamidomethyl (C) (apply to specified residues or termini only)
Ions Score: 33 Expect: 0.087
Matches : 24/90 fragment ions using 68 most intense peaks (help)

#	b	b^{++}	b^{0}	b^{0++}	Seq.	y	y^{++}	y^{*}	y^{*++}	y^{0}	y^{0++}	#
1	164.0706	82.5389			Y							11
2	235.1077	118.0575			A	1174.5626	587.7850	1157.5361	579.2717	1156.5521	578.7797	10
3	348.1918	174.5995			L	1103.5255	552.2664	1086.4990	543.7531	1085.5150	543.2611	9
4	511.2551	256.1312			Y	990.4415	495.7244	973.4149	487.2111	972.4309	486.7191	8
5	626.2821	313.6447	608.2715	304.6394	D	827.3781	414.1927	810.3516	405.6794	809.3676	405.1874	7
6	697.3192	349.1632	679.3086	340.1579	A	712.3512	356.6792	695.3246	348.1660	694.3406	347.6740	6
7	798.3668	399.6871	780.3563	390.6818	T	641.3141	321.1607	624.2875	312.6474	623.3035	312.1554	5
8	961.4302	481.2187	943.4196	472.2134	Y	540.2664	270.6368	523.2399	262.1236	522.2558	261.6316	4
9	1090.4728	545.7400	1072.4622	536.7347	E	377.2031	189.1052	360.1765	180.5919	359.1925	180.0999	3
10	1191.5204	596.2639	1173.5099	587.2586	T	248.1605	124.5839	231.1339	116.0706	230.1499	115.5786	2
11					K	147.1128	74.0600	130.0863	65.5468			1

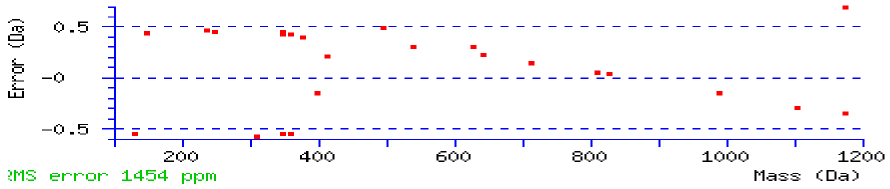

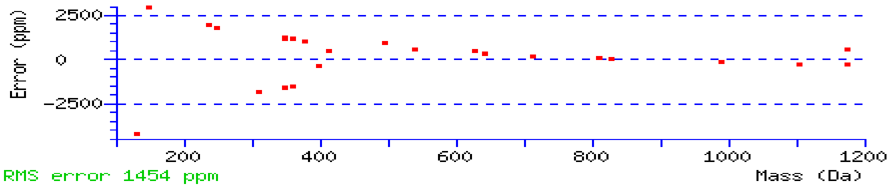

MS/MS Fragmentation of IVKPNGEKPDEFESGISQALLELEMNSDLK
Found in RS7_HUMAN in SwissProt, 40S ribosomal protein S7 OS=Homo sapiens GN=RPS7 PE=1 SV=1

Match to Query 599: 3346.622096 from(837.662800,4+) intensity(6379.0000) index(457)
Data file IS_111911_23b.txt

Click mouse within plot area to zoom in by factor of two about that point
Or, Plot from 100 to 2100 Da Full range
Label all possible matches ○ Label matches used for scoring ◉
Show Y-axis ☐

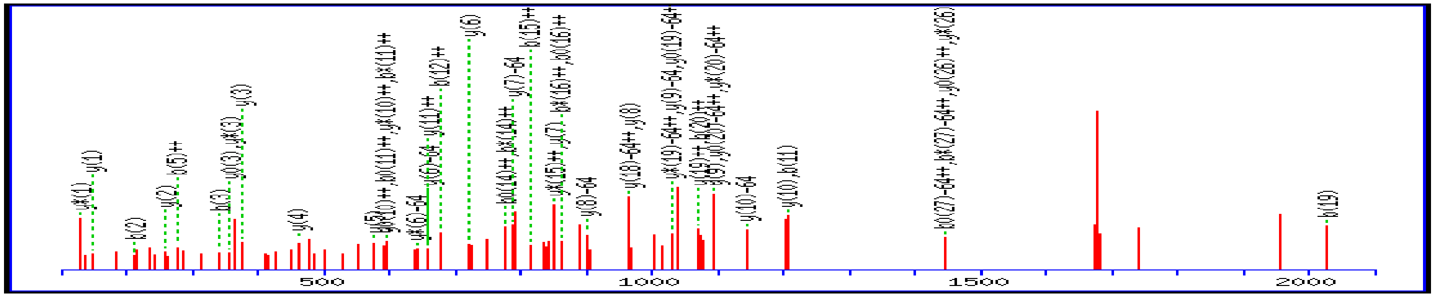

Monoisotopic mass of neutral peptide Mr(calc): 3345.6649
Fixed modifications: Carbamidomethyl (C) (apply to specified residues or termini only)
Variable modifications:
M25 : Oxidation (M), with neutral losses 0.0000(shown in table), 63.9983
Ions Score: 32 Expect: 0.04
Matches : 47/502 fragment ions using 67 most intense peaks (help)

#	b	b^{++}	b^{*}	b^{*++}	b^{0}	b^{0++}	Seq.	y	y^{++}	y^{*}	y^{*++}	y^{0}	y^{0++}	#
1	114.0913	57.5493					I							30
2	213.1598	107.0835					V	3233.5882	1617.2977	3216.5617	1608.7845	3215.5776	1608.2925	29
3	341.2547	171.1310	324.2282	162.6177			K	3134.5198	1567.7635	3117.4933	1559.2503	3116.5092	1558.7583	28
4	438.3075	219.6574	421.2809	211.1441			P	3006.4248	1503.7161	2989.3983	1495.2028	2988.4143	1494.7108	27
5	552.3504	276.6788	535.3239	268.1656			N	2909.3721	1455.1897	2892.3455	1446.6764	2891.3615	1446.1844	26
6	609.3719	305.1896	592.3453	296.6763			G	2795.3291	1398.1682	2778.3026	1389.6549	2777.3186	1389.1629	25
7	738.4145	369.7109	721.3879	361.1976	720.4039	360.7056	E	2738.3077	1369.6575	2721.2811	1361.1442	2720.2971	1360.6522	24
8	866.5094	433.7584	849.4829	425.2451	848.4989	424.7531	K	2609.2651	1305.1362	2592.2385	1296.6229	2591.2545	1296.1309	23
9	963.5622	482.2847	946.5356	473.7715	945.5516	473.2795	P	2481.1701	1241.0887	2464.1436	1232.5754	2463.1596	1232.0834	22
10	1078.5891	539.7982	1061.5626	531.2849	1060.5786	530.7929	D	2384.1174	1192.5623	2367.0908	1184.0490	2366.1068	1183.5570	21
11	1207.6317	604.3195	1190.6052	595.8062	1189.6212	595.3142	E	2269.0904	1135.0488	2252.0639	1126.5356	2251.0799	1126.0436	20
12	1354.7001	677.8537	1337.6736	669.3404	1336.6896	668.8484	F	2140.0478	1070.5276	2123.0213	1062.0143	2122.0373	1061.5223	19
13	1483.7427	742.3750	1466.7162	733.8617	1465.7322	733.3697	E	1992.9794	996.9933	1975.9529	988.4801	1974.9688	987.9881	18
14	1570.7748	785.8910	1553.7482	777.3777	1552.7642	776.8857	S	1863.9368	932.4720	1846.9103	923.9588	1845.9263	923.4668	17
15	1627.7962	814.4018	1610.7697	805.8885	1609.7857	805.3965	G	1776.9048	888.9560	1759.8782	880.4428	1758.8942	879.9508	16
16	1740.8803	870.9438	1723.8537	862.4305	1722.8697	861.9385	I	1719.8833	860.4453	1702.8568	851.9320	1701.8728	851.4400	15
17	1827.9123	914.4598	1810.8858	905.9465	1809.9018	905.4545	S	1606.7993	803.9033	1589.7727	795.3900	1588.7887	794.8980	14
18	1955.9709	978.4891	1938.9443	969.9758	1937.9603	969.4838	Q	1519.7672	760.3873	1502.7407	751.8740	1501.7567	751.3820	13
19	2027.0080	1014.0076	2009.9815	1005.4944	2008.9974	1005.0024	A	1391.7087	696.3580	1374.6821	687.8447	1373.6981	687.3527	12
20	2140.0921	1070.5497	2123.0655	1062.0364	2122.0815	1061.5444	L	1320.6715	660.8394	1303.6450	652.3261	1302.6610	651.8341	11
21	2253.1761	1127.0917	2236.1496	1118.5784	2235.1656	1118.0864	L	1207.5875	604.2974	1190.5609	595.7841	1189.5769	595.2921	10
22	2382.2187	1191.6130	2365.1922	1183.0997	2364.2082	1182.6077	E	1094.5034	547.7553	1077.4769	539.2421	1076.4929	538.7501	9
23	2495.3028	1248.1550	2478.2762	1239.6418	2477.2922	1239.1498	L	965.4608	483.2340	948.4343	474.7208	947.4503	474.2288	8
24	2624.3454	1312.6763	2607.3188	1304.1631	2606.3348	1303.6710	E	852.3768	426.6920	835.3502	418.1787	834.3662	417.6867	7
25	2771.3808	1386.1940	2754.3542	1377.6808	2753.3702	1377.1888	M	723.3342	362.1707	706.3076	353.6574	705.3236	353.1654	6
26	2885.4237	1443.2155	2868.3972	1434.7022	2867.4132	1434.2102	N	576.2988	288.6530	559.2722	280.1397	558.2882	279.6477	5
27	2972.4557	1486.7315	2955.4292	1478.2182	2954.4452	1477.7262	S	462.2558	231.6316	445.2293	223.1183	444.2453	222.6263	4
28	3087.4827	1544.2450	3070.4561	1535.7317	3069.4721	1535.2397	D	375.2238	188.1155	358.1973	179.6023	357.2132	179.1103	3
29	3200.5668	1600.7870	3183.5402	1592.2737	3182.5562	1591.7817	L	260.1969	130.6021	243.1703	122.0888			2
30							K	147.1128	74.0600	130.0863	65.5468			1

MS/MS Fragmentation of YSVDIPLDK
Found in RL27_HUMAN in SwissProt, 60S ribosomal protein L27 OS=Homo sapiens GN=RPL27 PE=1 SV=2

Match to Query 148: 1049.160648 from(525.587600,2+) intensity(3442.5000) index(44)
Data file IS_111911_23b.txt

Click mouse within plot area to zoom in by factor of two about that point
Or, Plot from 100 to 1050 Da Full range
Label all possible matches ○ Label matches used for scoring ◉
Show Y-axis ☐

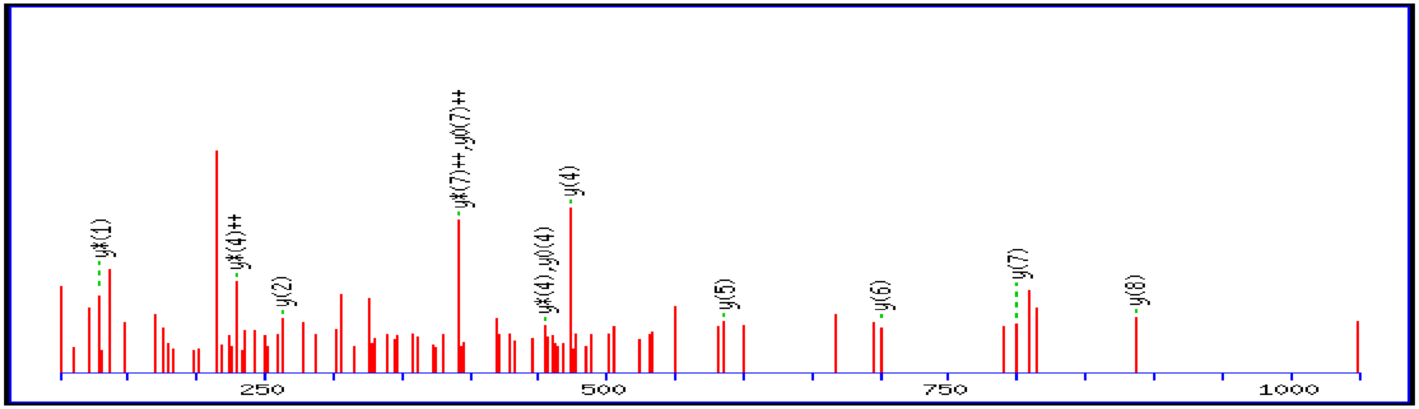

Monoisotopic mass of neutral peptide Mr(calc): 1048.5441
Fixed modifications: Carbamidomethyl (C) (apply to specified residues or termini only)
Ions Score: 28 Expect: 0.3
Matches : 12/76 fragment ions using 24 most intense peaks (help)

#	b	b++	b0	b0++	Seq.	y	y++	y*	y*++	y0	y0++	#
1	164.0706	82.5389			Y							9
2	251.1026	126.0550	233.0921	117.0497	S	886.4880	443.7477	869.4615	435.2344	868.4775	434.7424	8
3	350.1710	175.5892	332.1605	166.5839	V	799.4560	400.2316	782.4294	391.7184	781.4454	391.2264	7
4	465.1980	233.1026	447.1874	224.0974	D	700.3876	350.6974	683.3610	342.1842	682.3770	341.6921	6
5	578.2821	289.6447	560.2715	280.6394	I	585.3606	293.1840	568.3341	284.6707	567.3501	284.1787	5
6	675.3348	338.1710	657.3243	329.1658	P	472.2766	236.6419	455.2500	228.1287	454.2660	227.6366	4
7	788.4189	394.7131	770.4083	385.7078	L	375.2238	188.1155	358.1973	179.6023	357.2132	179.1103	3
8	903.4458	452.2266	885.4353	443.2213	D	262.1397	131.5735	245.1132	123.0602	244.1292	122.5682	2
9					K	147.1128	74.0600	130.0863	65.5468			1

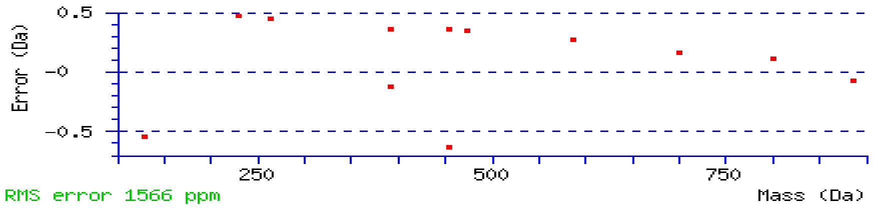

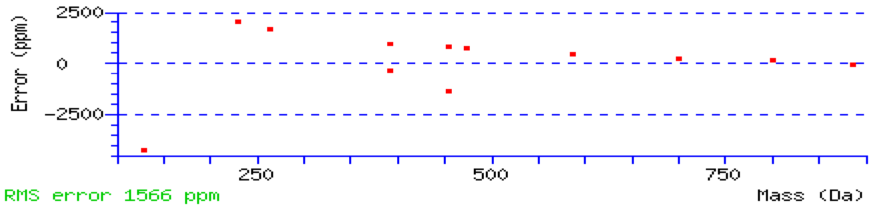

MS/MS Fragmentation of **LENMWQERFLSPLLGR**
Found in **CELR3_HUMAN** in **SwissProt**, Cadherin EGF LAG seven-pass G-type receptor 3 OS=Homo sapiens GN=CELSR3 PE=1 SV=2

Match to Query 454: 2003.616448 from(1002.815500,2+) intensity(12325.0000) index(389)
Data file IS_111911_23b.txt

Click mouse within plot area to zoom in by factor of two about that point
Or, Plot from 100 to 1600 Da Full range
Label all possible matches ○ Label matches used for scoring ◉
Show Y-axis ☐

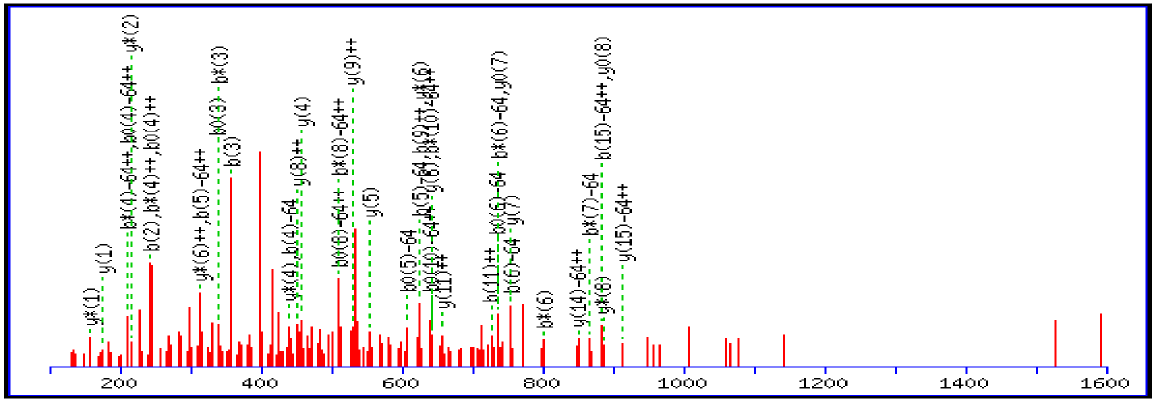


```
Monoisotopic mass of neutral peptide Mr(calc): 2004.0251
Fixed modifications: Carbamidomethyl (C) (apply to specified residues or termini only)
Variable modifications:
M4      : Oxidation (M), with neutral losses 63.9983(shown in table), 0.0000
Ions Score: 28  Expect: 0.18
Matches : 42/254 fragment ions using 67 most intense peaks   (help)
```

#	b	b^{++}	b^{*}	b^{*++}	b^{0}	b^{0++}	Seq.	y	y^{++}	y^{*}	y^{*++}	y^{0}	y^{0++}	#
1	114.0913	57.5493					L							16
2	***243.1339***	122.0706			225.1234	113.0653	E	1827.9500	914.4787	1810.9235	905.9654	1809.9395	905.4734	15
3	***357.1769***	179.0921	340.1503	170.5788	339.1663	170.0868	N	1698.9075	849.9574	1681.8809	841.4441	1680.8969	840.9521	14
4	***440.2140***	220.6106	423.1874	212.0974	422.2034	211.6053	M	1584.8645	792.9359	1567.8380	784.4226	1566.8540	783.9306	13
5	***626.2933***	313.6503	609.2667	305.1370	608.2827	304.6450	W	1501.8274	751.4173	1484.8009	742.9041	1483.8168	742.4121	12
6	***754.3519***	377.6796	737.3253	369.1663	736.3413	368.6743	Q	1315.7481	658.3777	1298.7215	649.8644	1297.7375	649.3724	11
7	883.3945	442.2009	866.3679	433.6876	865.3839	433.1956	E	1187.6895	594.3484	1170.6630	585.8351	1169.6790	585.3431	10
8	1039.4956	520.2514	1022.4690	511.7381	1021.4850	511.2461	R	1058.6469	529.8271	1041.6204	521.3138	1040.6364	520.8218	9
9	1186.5640	593.7856	1169.5374	585.2724	1168.5534	584.7803	F	902.5458	451.7765	885.5193	443.2633	884.5352	442.7713	8
10	1299.6480	650.3277	1282.6215	641.8144	1281.6375	641.3224	L	***755.4774***	378.2423	738.4509	369.7291	737.4668	369.2371	7
11	1386.6801	693.8437	1369.6535	685.3304	1368.6695	684.8384	S	***642.3933***	321.7003	625.3668	313.1870	624.3828	312.6950	6
12	1483.7328	742.3701	1466.7063	733.8568	1465.7223	733.3648	P	***555.3613***	278.1843	538.3348	269.6710			5
13	1596.8169	798.9121	1579.7904	790.3988	1578.8063	789.9068	L	***458.3085***	229.6579	441.2820	221.1446			4
14	1709.9010	855.4541	1692.8744	846.9408	1691.8904	846.4488	L	345.2245	173.1159	328.1979	164.6026			3
15	1766.9224	883.9649	1749.8959	875.4516	1748.9119	874.9596	G	232.1404	116.5738	215.1139	108.0606			2
16							R	***175.1190***	88.0631	158.0924	79.5498			1
