# Supplementary material for: Comparative Proteomics Reveals Novel Components at the Plasma Membrane of Differentiated HepaRG Cells and Different Distribution in Hepatocyte- and Biliary-Like Cells
Source: PLoS One. 2013 Aug 20;8(8):e71859. doi: 10.1371/journal.pone.0071859 (PMC3748114; doi:10.1371/journal.pone.0071859)
Supplement: Table S3 — Proteins with a role in viral reproduction. This classification resulted from the analysis of the Mascot results shown in Tables S1 and S2, using the Scaffold software-version 4.0.4. (DOCX) [file pone.0071859.s009.docx]

| # | Identified Proteins (45) | Accession Number |
| --- | --- | --- |
| 1 | Coatomer subunit beta OS=Homo sapiens GN=COPB1 PE=1 SV=3 | COPB_HUMAN |
| 2 | Keratin, type I cytoskeletal 19 OS=Homo sapiens GN=KRT19 PE=1 SV=4 | K1C19_HUMAN |
| 3 | K2C8_HUMAN | K2C8_HUMAN |
| 4 | Heat shock cognate 71 kDa protein OS=Homo sapiens GN=HSPA8 PE=1 SV=1 | HSP7C_HUMAN |
| 5 | IF4A1_HUMAN | IF4A1_HUMAN |
| 6 | Keratin, type I cytoskeletal 18 OS=Homo sapiens GN=KRT18 PE=1 SV=2 | K1C18_HUMAN |
| 7 | 60 kDa heat shock protein, mitochondrial OS=Homo sapiens GN=HSPD1 PE=1 SV=2 | CH60_HUMAN |
| 8 | Importin subunit beta-1 OS=Homo sapiens GN=KPNB1 PE=1 SV=2 | IMB1_HUMAN |
| 9 | 14-3-3 protein epsilon OS=Homo sapiens GN=YWHAE PE=1 SV=1 | 1433E_HUMAN |
| 10 | Guanine nucleotide-binding protein subunit beta-2-like 1 OS=Homo sapiens GN=GNB2L1 PE=1 SV=3 | GBLP_HUMAN |
| 11 | Nucleophosmin OS=Homo sapiens GN=NPM1 PE=1 SV=2 | NPM_HUMAN |
| 12 | Apoptosis regulator BAX OS=Homo sapiens GN=BAX PE=1 SV=1 | BAX_HUMAN |
| 13 | Staphylococcal nuclease domain-containing protein 1 OS=Homo sapiens GN=SND1 PE=1 SV=1 | SND1_HUMAN |
| 14 | ADP-ribosylation factor 1 OS=Homo sapiens GN=ARF1 PE=1 SV=2 | ARF1_HUMAN (+1) |
| 15 | Peptidyl-prolyl cis-trans isomerase A OS=Homo sapiens GN=PPIA PE=1 SV=2 | PPIA_HUMAN |
| 16 | Ubiquitin-60S ribosomal protein L40 OS=Homo sapiens GN=UBA52 PE=1 SV=2 | RL40_HUMAN (+3) |
| 17 | 60S acidic ribosomal protein P0 OS=Homo sapiens GN=RPLP0 PE=1 SV=1 | RLA0_HUMAN |
| 18 | 40S ribosomal protein SA OS=Homo sapiens GN=RPSA PE=1 SV=4 | RSSA_HUMAN |
| 19 | 40S ribosomal protein S16 OS=Homo sapiens GN=RPS16 PE=1 SV=2 | RS16_HUMAN |
| 20 | Proteasome activator complex subunit 2 OS=Homo sapiens GN=PSME2 PE=1 SV=4 | PSME2_HUMAN |
| 21 | 60S ribosomal protein L10a OS=Homo sapiens GN=RPL10A PE=1 SV=2 | RL10A_HUMAN |
| 22 | X-ray repair cross-complementing protein 5 OS=Homo sapiens GN=XRCC5 PE=1 SV=3 | XRCC5_HUMAN |
| 23 | Proteasome subunit beta type-1 OS=Homo sapiens GN=PSMB1 PE=1 SV=2 | PSB1_HUMAN |
| 24 | X-ray repair cross-complementing protein 6 OS=Homo sapiens GN=XRCC6 PE=1 SV=2 | XRCC6_HUMAN |
| 25 | Proteasome subunit alpha type-2 OS=Homo sapiens GN=PSMA2 PE=1 SV=2 | PSA2_HUMAN |
| 26 | 60S ribosomal protein L11 OS=Homo sapiens GN=RPL11 PE=1 SV=2 | RL11_HUMAN |
| 27 | 60S ribosomal protein L18 OS=Homo sapiens GN=RPL18 PE=1 SV=2 | RL18_HUMAN |
| 28 | 60S ribosomal protein L23a OS=Homo sapiens GN=RPL23A PE=1 SV=1 | RL23A_HUMAN |
| 29 | 60S acidic ribosomal protein P1 OS=Homo sapiens GN=RPLP1 PE=1 SV=1 | RLA1_HUMAN |
| 30 | 60S ribosomal protein L27 OS=Homo sapiens GN=RPL27 PE=1 SV=2 | RL27_HUMAN |
| 31 | 60S ribosomal protein L7 OS=Homo sapiens GN=RPL7 PE=1 SV=1 | RL7_HUMAN |
| 32 | 40S ribosomal protein S8 OS=Homo sapiens GN=RPS8 PE=1 SV=2 | RS8_HUMAN |
| 33 | 60S ribosomal protein L31 OS=Homo sapiens GN=RPL31 PE=1 SV=1 | RL31_HUMAN |
| 34 | 40S ribosomal protein S7 OS=Homo sapiens GN=RPS7 PE=1 SV=1 | RS7_HUMAN |
| 35 | Proteasome activator complex subunit 1 OS=Homo sapiens GN=PSME1 PE=1 SV=1 | PSME1_HUMAN |
| 36 | 40S ribosomal protein S15 OS=Homo sapiens GN=RPS15 PE=1 SV=2 | RS15_HUMAN |
| 37 | 40S ribosomal protein S24 OS=Homo sapiens GN=RPS24 PE=1 SV=1 | RS24_HUMAN |
| 38 | PSA5_HUMAN | PSA5_HUMAN |
| 39 | 40S ribosomal protein S9 OS=Homo sapiens GN=RPS9 PE=1 SV=3 | RS9_HUMAN |
| 40 | Proteasome subunit beta type-3 OS=Homo sapiens GN=PSMB3 PE=1 SV=2 | PSB3_HUMAN |
| 41 | 60S ribosomal protein L27a OS=Homo sapiens GN=RPL27A PE=1 SV=2 | RL27A_HUMAN |
| 42 | 60S ribosomal protein L30 OS=Homo sapiens GN=RPL30 PE=1 SV=2 | RL30_HUMAN |
| 43 | 40S ribosomal protein S13 OS=Homo sapiens GN=RPS13 PE=1 SV=2 | RS13_HUMAN |
| 44 | 40S ribosomal protein S5 OS=Homo sapiens GN=RPS5 PE=1 SV=4 | RS5_HUMAN |
| 45 | GTP-binding nuclear protein Ran OS=Homo sapiens GN=RAN PE=1 SV=3 | RAN_HUMAN |
